# Mechanochemical Solid Form Screening of Zeolitic Imidazolate Frameworks Using Structure-Directing Liquid Additives

**DOI:** 10.1021/jacs.5c04043

**Published:** 2025-07-24

**Authors:** Ivana Brekalo, Katarina Lisac, Joseph R. Ramirez, Petra Pongrac, Andreas Puškarić, Srećko Valić, Yizhi Xu, Michael Ferguson, Joseph M. Marrett, Mihails Arhangelskis, Tomislav Friščić, K. Travis Holman

**Affiliations:** † Division of Physical Chemistry, 54583 Ruđer Bošković Institute, Zagreb 10000, Croatia; ‡ Department of Chemistry, 8368 Georgetown University, Washington, D.C 20057, United States; § Department of Chemistry, McGill University, Montréal H3A 0B8, Canada; ∥ Faculty of Chemical Engineering and Technology, University of Zagreb, Zagreb 10000, Croatia; ⊥ Division of Materials Chemistry, Ruđer Bošković Institute, Zagreb 10000, Croatia; # Faculty of Medicine, University of Rijeka, Rijeka 51000, Croatia; ∇ Faculty of Chemistry, 49605 University of Warsaw, Warsaw 02-093, Poland; ○ School of Chemistry, 1724 University of Birmingham, Birmingham B15 2TT, U.K.

## Abstract

We demonstrate a systematic application of the mechanochemical liquid-assisted grinding (LAG) methodology to screen for forms of zinc imidazolate (Zn**Im**_2_), of fundamental importance as the simplest member of the zeolitic imidazolate framework materials family. The exploration of 45 different liquid additives, selected based on their molecular structure and physicochemical properties has resulted in eight different Zn**Im**_2_ topological forms, appearing in 13 crystallographically distinct solid forms (including two previously unknown forms of the **crb** (BCT) topology), amorphous phases, and the interrupted **moc**-Zn_4_ **Im**_8_H**Im**. All prepared topological forms were also explored computationally, using dispersion-corrected periodic density functional theory (DFT) calculations, enabling the rationalization of screening outcomes, and setting the stage for future prediction of additive-directed metal–organic framework (MOF) synthesis. This first systematic exploration of LAG in screening for three-dimensional MOFs demonstrates the potential of the liquid additive to not only accelerate materials synthesis, but also to direct it toward topologically different MOFs. The discovery of novel forms of a material that already exhibits at least 21 crystallographically and functionally different forms provides a strong testimony on the power of mechanochemistry in metal–organic materials discovery.

## Graphical abstract

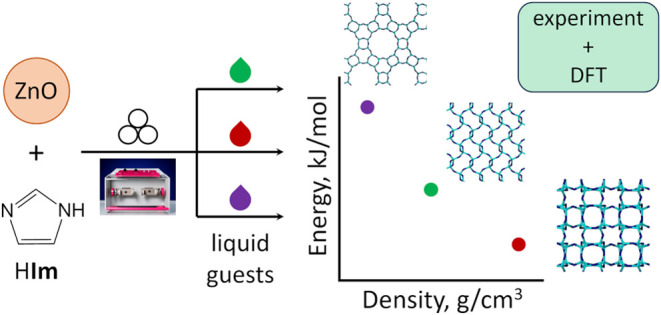

## 1 Introduction

The physicochemical properties of materials are intimately linked with their structure, so form screening is a crucial aspect of solid-state materials science. For example, solid form screening in the pharmaceutical industry is prevalent, as it is well recognized that different solid forms of active pharmaceutical ingredients can offer different performance properties, such as solubility, stability, dissolution rates or bioavailability.^1–4^ Similarly, the properties of porous materials are highly dependent on the size, shape, and chemical compositions of their pores and pore windows.^5–8^ Significant effort has thus been dedicated to the rational design of metal–organic frameworks (MOFs) with advanced properties (such as microporosity, conductivity, or adsorbent selectivity) by engineering their structures.^9–16^ A particular challenge is the control of polymorphism, where the same starting materials can give many different products, the most porous of which are inherently metastable with respect to their less porous or nonporous polymorphs, often resulting in MOF flexibility^17–20^ and form conversion.^21–23^ Recent years have thus seen increasing focus on the fundamental understanding of structure-directing effects and stability in MOFs.^13,24–26^

Zeolitic imidazolate frameworks (ZIFs)^27–31^ – a class of MOFs built from tetrahedral metal centers (e.g., Zn^2+^, Cd^2+^, Co^2+^) and imidazolate ligands – are particularly susceptible to polymorphism and form diversity.

Like zeolites, ZIFs can exist in many topological forms. For example, the sterically unhindered zinc imidazolate (Zn**Im**_2_), is known to exist in at least 18 topologies^32^ (Scheme 1), some of which can take several distinct crystallographic/conformational forms. Furthermore, **crb**-Zn**Im**_2_ ^33,34^ exists in three different forms^27,35^ with different space groups, unit cell volumes, and predicted pore properties (Tables 1 and S9). Approaches to synthesize Zn**Im**_2_ often yield (pseudo)­polymorphic mixtures, and some of the topologically distinct forms (e.g., **mer**, **gis**) have, to the best of our knowledge, only ever been reported in one experiment each, isolated as single crystals.^27^ Amorphous forms of Zn**Im**_2_ have also been prepared,^36–38^ as has an interrupted dense framework of the **moc** topology (**moc**-Zn_4_ **Im**_8_H**Im**).^39,40^ Overall, ZIF syntheses broadly, and Zn**Im**_2_ synthesis in particular, pose a significant challenge in terms of solid form control and the stabilization of certain porous forms.

**Scheme 1:**
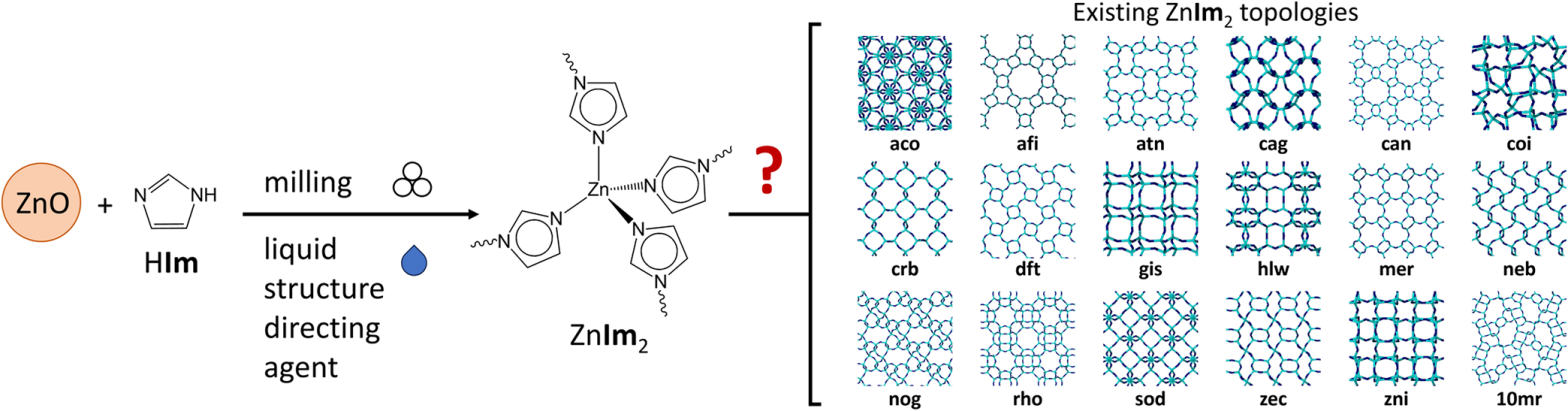
Use of Liquid-directed Mechanochemical Screening in the Preparation of Zinc Imidazolate Topologies

**Table 1:** Calculated Structural and Porosity Parameters of Different Forms of **crb**-Zn**Im**_2_

	CSD code	space group	V_UC_, Å^3^	V_UC_ per Zn, Å^3^	T/V, nm^–3^	SSA, m^2^/g	*d*_max_^pore^, Å	*d*_lim_^pore^, Å	void fraction, %
**crb1**	VEJYIT	*Pbca*	5706.6	356.7	2.80	1495	6.01	5.04	23.4
**crb2**	GITTEJ	*P*2/*n*	4414.4	275.9	3.62	465	6.80	1.70	11.9
**crb3**	VEJYEP	*P*2_1_/*n*	2195.9	274.5	3.64	428	6.12	1.87	11.0
**crbA**	this work	*P*2_1_/*c*	4503.7	281.5	3.55	511	6.40	1.96	12.0
**crbT**	this work	*Pnnm*	2571.4	321.4	3.11	321	4.75	3.66	14.7

The predicted specific surface area (SSA, N_2_ at 77 K), maximum pore diameter (*d*_max_^pore^), pore limiting diameter (*d*_lim_^pore^) and void fraction were all calculated using the Pore Analyzer function in Mercury. V_UC_ designates the unit cell volume, and T/V designates the density of metal atoms per unit volume.

Solid form control strategies used on ZIFs derived from substituted imidazoles, such as the use of specific linkers,^31,41^ mixed linkers,^35^ and the steric index approach^42^ unfortunately cannot be applied to unsubstituted M**Im**_2_ ZIFs. Instead, solid form control is often achieved using putative templates, such as amide solvents,^43–46^ structure-directing agents,^47^ or macrocycles.^48^ This often involves time- and energy-intensive solvothermal screening, which inherently limits the template scope to molecules that are miscible with/soluble in the solvents used. In that context, the development of methodologies for sustainable, efficient MOF solid form screening is very important, but has remained largely unexplored, especially in comparison with the wide, continuously growing set of methodologies being deployed in pharmaceutical materials science. In particular, mechanochemical ball milling techniques,^49^ especially those involving a liquid additive (liquid-assisted grinding, LAG) have been demonstrated as highly efficient for rapid discovery of new forms of organic solids, such as polymorphs, cocrystals, salts and more,^50–52^ including sustainably^53^ at a large scale.^54,55^

Here we demonstrate the systematic exploration of liquid additives for the mechanochemical solid form screening of the simplest and most polymorphic ZIF representative, zinc imidazolate. We show that this fast, effective and environmentally friendly screening approach can provide 14 different crystalline ZIF frameworks, including two new forms of the **crb** (BCT) topology, as well as amorphous solid forms. Application of previously validated periodic density functional theory (DFT) computational methods^56^ to the solvated Zn**Im**_2_ systems reveals that, while kinetic effects can play a significant role, the topological outcomes of LAG Zn**Im**_2_ syntheses are ultimately governed by the thermodynamic stabilities of the specific solvated Zn**Im**_2_ structures. We anticipate that this solid form screening method can be widely applied to discover and control the polymorphism of different classes of host–guest materials.

## 2 Results and Discussion

### 2.1 General Outcomes of LAG Screening

Mechanochemistry has been shown to be a highly versatile approach to synthesize a diverse range of coordination compounds, encompassing discrete complexes, as well as coordination polymers of different dimensionality, including MOFs.^57–62^ Moreover, mechanochemical synthesis of ZIFs has been explored, including neat grinding (NG) syntheses,^63^ LAG/ILAG (ion-and-liquid assisted grinding)^64–66^ and aging syntheses,^67–69^ *in situ* and *ex situ* monitoring,^70–74^ discovery of new ZIF topologies through mechanochemistry,^75^ scale-up,^54,76,77^ expanding the ligand scope of certain ZIF topologies,^78^ and encapsulation of functional guests such as fullerenes, catalysts, MRI agents or enzymes into ZIFs, creating hybrid materials.^62,79–81^ Furthermore, periodic density functional theory (DFT) calculations have been used to rationalize mechanochemical reactivity,^56,82^ and even predict the crystal structures of the products of milling syntheses.^83,84^ Despite this extensive body of research, the role of liquid additives in the mechanochemical syntheses of MOFs, and their potential utility as stabilizers, structure-directing agents, and even presumptive templates in MOF solid form screening has, to the best of our knowledge, not been systematically studied.

Inspired by the zeolite community’s use of cationic templates in the controlled synthesis of desired zeolite topologies, and our own work on the solvothermal templation of **mer**-Zn**Im**_2_,^48^ we have previously used the macrocyclic Cram’s cavitands^85^ for targeted mechanochemical synthesis of **rho**-Zn**Im**_2_.^86,87^ Certain cavitands were highly successful at templating the *double-8-ring (d8r)* motif of the **rho** topology via eight (imidazolate) C–H•••O (cavitand) hydrogen bonds, enabling the synthesis of decagram quantities of a highly porous **rho**-Zn**Im**_2_ material in quantitative yield, without bulk solvent. However, the cavitand template molecules themselves are not ubiquitous, require solvothermal organic synthesis and purification, and most importantly, they template only one specific topological motif – the *d8r*. We are now seeking presumptive templates that would be readily available, and able to direct the synthesis of many different solid forms of ZIFs in a fast and efficient mechanochemical screening. One possible avenue is the use of small-molecule liquids as structure-directing agents. The role of liquid additives in mechanochemistry is still not fully resolved, but they are known to assist and accelerate mechanochemical syntheses, while sometimes also directing the synthetic outcome,^88^ including in coordination polymers,^89^ MOFs,^90,91^ and ZIFs.^64^ While we cannot exclude kinetic or nucleation effects related to the presence of different liquid phases, we propose that small molecule liquid additives can be used as presumptive templates and stabilizing pore-fillers, and will provide an avenue for mechanochemical screening of different solid forms of ZIFs.

We selected 45 different liquids to serve as additives in the mechanochemical reactions of ZnO and imidazole (H**Im**). The liquids were chosen to sample a range of properties, including polarity, aromaticity, proticity, functional groups, molecular shapes and sizes, etc. The use of mechanochemistry enabled a very broad additive scope, as solubility of the reagents is not necessarily a limiting factor. For example, it is possible to use highly nonpolar liquids such as cyclohexane (cHANE) in conjunction with the ionic ZnO and the highly polar and protic imidazole. The full list of liquid additives explored can be found in SI-1.7. (Table S1). The screening was conducted by adding a set amount of a liquid (100 μL, unless otherwise noted; η = 0.5 μL/mg) into a milling jar containing two milling balls and a mixture of ZnO and H**Im** in the respective 1:2 stoichiometric ratio (total mass = 200 mg), and then ball-milling the mixture at a frequency of 30 Hz for 15, 30, 60, or 90 min. One larger (*d* = 9 mm, *m* = 3.5 g) and one smaller (*d* = 7 mm, *m* = 1.4 g) stainless steel milling ball were used to provide both sufficient impact power (larger, heavier ball) as well as increase the number of collisions and improve shear and mixing (smaller ball), while avoiding the balls getting stuck inside the jar.^92,93^ Neat grinding control experiments (without the addition of liquid additives) were also performed. The milled product mixtures were then analyzed by powder X-ray diffraction (PXRD), and the topological outcome of the synthesis determined by comparison to an internal database of simulated PXRD patterns of different ZIF forms, whose structures were extracted from the Cambridge Structural Database (CSD).^94^ For selected liquid additives, different experimental conditions were tested (using steel or Teflon milling jars, different amounts of liquid, different milling times, aging the reaction mixture in air at room temperature).

The screening results (section SI-2.1, summarized in section SI-2.1.47, Tables S3 and S4, and Schemes S1–S3) can be classified into three main outcomes (Figure 1): case (1) the liquid additive yields a pure Zn**Im**_2_ product of a single topology; case (2) the liquid additive yields a mixture of different Zn**Im**_2_ topologies; or case (3) the topological outcome changes depending on other experimental conditions. In most cases, the resulting framework structures presumably encapsulate the used additives as guests, and the resulting materials will be named *x*guest@**top**-Zn**Im**_2_, where **top** designates the topology and form of the product, and *x* the number of included guest molecules per Zn atom. For example, reactions with added cyclohexanone (cHONE) always yielded 0.5cHONE@**cag**-Zn**Im**_2_; the cHONE solvate of the **cag**-topology zinc imidazolate^95^ (compares to 0.5DMF@cag-Zn**Im**_2_, CSD code VEJYUF01,^47^ DMF = *N*,*N*-dimethylformamide), regardless of milling time or milling vessel material (Figures 1b, S10). Conversely, milling with acetonitrile (MeCN) provides a mixture of **coi-**Zn**Im**_2_ (compares to CSD code IMIDZB07 ^1,23^ ) and **zni-**Zn**Im**_2_ (compares to CSD code IMIDZB02 ^1,23^ ) in varying ratios depending on the reaction conditions (Figures 1c, S27), but never as a single pure phase. For ease of future referencing, all reactions reliably providing phase-pure products regardless of the conditions (case 1) have been summarized in Scheme S1 of the SI, representing a blueprint for the quick and efficient synthesis of eight different Zn**Im**_2_ solid forms. The most common case is case number 3, where the templation outcome partially depends on the reaction conditions. Different conditions can result in different pure phases for the same liquid additive. For example, using cHANE as the structure-directing agent gives 0.5cHANE@**cag-**Zn**Im**_2_ (compares to 0.5DMF@**cag-**Zn**Im**_2_, CSD code VEJYUF01 ^47^) after 15 min of milling in a Teflon jar, but yields 0.5cHANE@**neb1-**Zn**Im**_2_ the cHANE solvate of form 1 of the **neb**-topology zinc imidazolate (hereafter named **neb1**, compares to 0.5MORPH@**neb1**-Zn**Im**_2_, CSD code KUDJOK,^95^ where MORPH = morpholine) upon longer milling (≥30 min) in a Teflon jar, or milling in a steel jar (Figure 1d, S7). Such changes in topology upon different milling periods were also previously reported in *in situ* studies of ZIF syntheses using substituted imidazolates.^65,74,75,84^ On the other hand, changing the reaction conditions sometimes switches between pure and mixed phases. A 15 min milling reaction using pyridine (PYR) as the liquid additive results in a mixture of *x*PYR@**crb1-**Zn**Im**_2_ (compares to 1.5DMF@**crb1-**Zn**Im**_2_, CSD code VEJYIT^27^) and 0.5PYR@**neb2-**Zn**Im**_2_ (CSD code KEVLEE^96^) phases, while milling for an hour provides the pure 0.5PYR@**neb2-**Zn**Im**_2_ phase (Figures S48, S49). All time-dependent transformations of solid forms in this work have been summarized in Scheme S2 in the SI, while reactions where the solid form outcome depends on the amount of liquid added are summarized in Scheme S3 in the SI.

**Figure 1:**
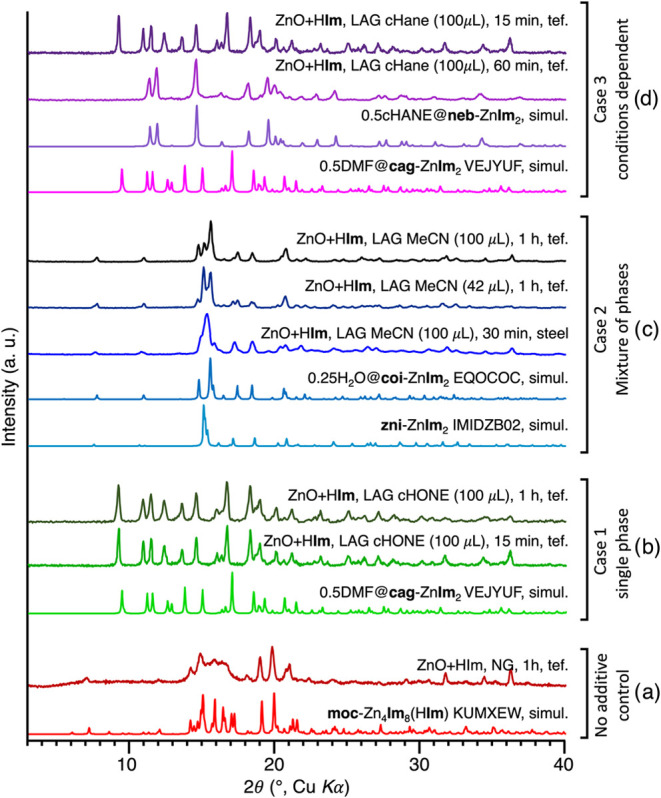
PXRD patterns representing the results of milling ZnO and imidazole (a) without additive, (b) with added cyclohexanone (cHONE), (c) with added acetonitrile (MeCN), (d) with added cyclohexane (cHANE). Tef = Teflon jar, simul.= simulated.

Overall, the presented fast and accessible screening method resulted in pure samples of eight different Zn**Im**_2_ topologies – namely **zni**,^1,23^ **coi**,^1,23^ **crb**,^27,35^ **cag**,^47^ **neb**,^95,96^ **nog**,^47^ **10mr** ^43^ and **afi** ^45^ – out of the 18 known Zn**Im**_2_ topologies, as well as the **moc**-Zn_4_ **Im**_8_H**Im** ^39^ interrupted framework, and amorphous phases (Figure 2). Importantly, this screening also revealed two new, crystallographically distinct forms of **crb**-Zn**Im**_2_, and provides the three already known **crb**-Zn**Im**_2_ phases (pure or in mixture), as well as the two known phases of **neb**-Zn**Im**_2_. Altogether, 14 distinct crystalline frameworks based on zinc imidazolate have thus been found via our screening method (Figure 1). Many of these exist in several solvated forms, encapsulating different guest molecules, providing even more distinct materials. The PXRD analyses and summaries of the topological outcomes of all reactions can be found in the Supporting Information (SI-2.1.), while herein we discuss only the observed trends, new forms, and selected interesting cases.

**Figure 2:**
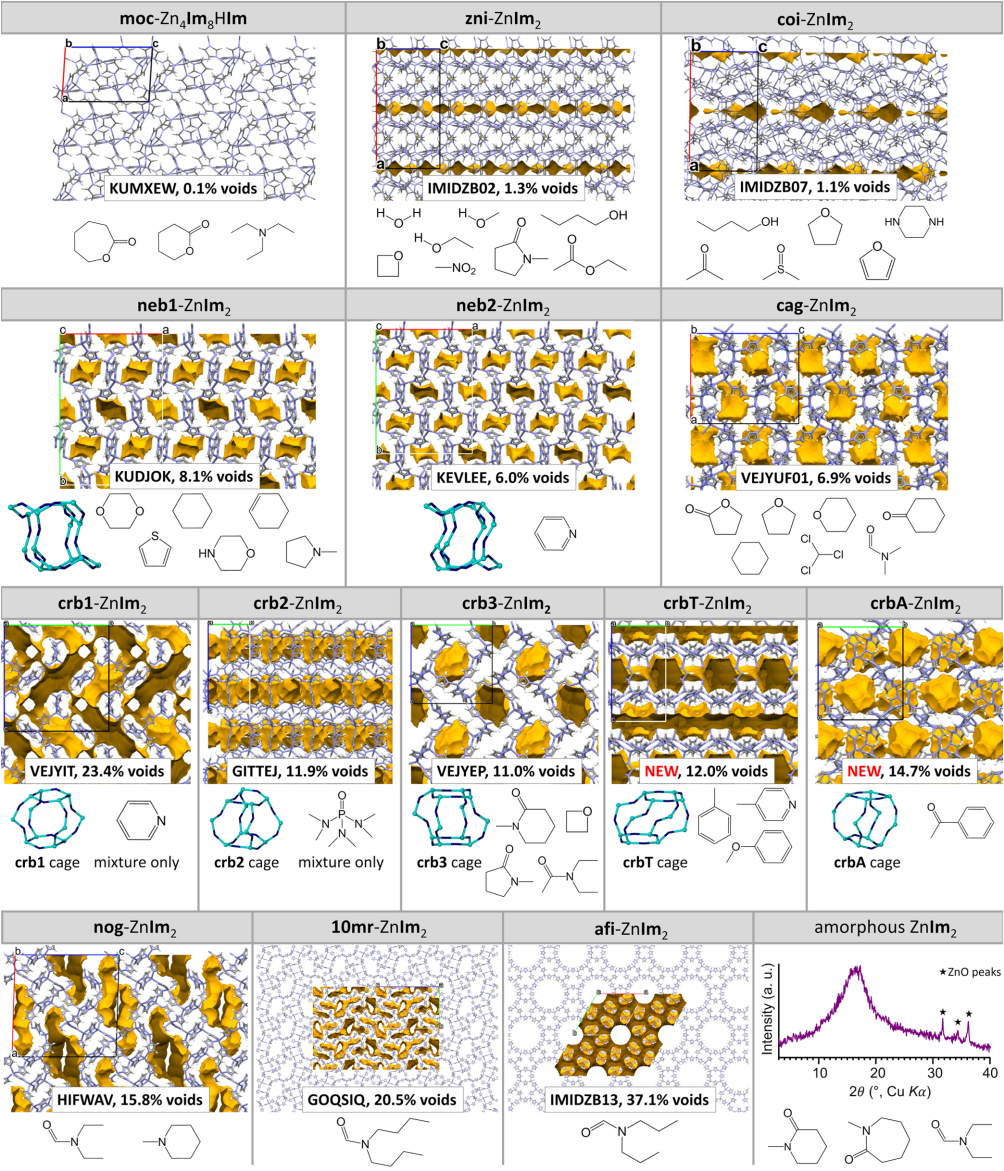
Overview of the topological results of LAG screening. The crystal structures of different topological forms obtained by LAG mechanochemical screening are shown in the capped sticks representations, along with their CSD code (if applicable), void percentage (calculated using the solvent accessible surface in Mercury (v. 2024.1.0, MacOS), 1.2 Å probe, grid spacing 0.3 Å), and the additive used to synthesize them as pure phases. Node-and-linker representations of the building block cages for the **crb** and **neb** forms are also shown (light blue = Zn, dark blue = Im^–^ centroid). Solvent accessible surfaces are shown in yellow contour.

### 2.2 Preparation of Dense Forms

The most commonly observed phases in the screening were, expectedly, the highest density forms (densities between 1.5 and 1.6 g/cm^3^): **moc**-Zn_4_ **Im**_8_H**Im** (3/45 liquids + NG, void fraction 0.1%), **zni**-Zn**Im**_2_ (10/45 liquids, void fraction 1.3%) and **coi**-Zn**Im**_2_ (10/45 liquids, void fraction 1.1%). The **moc**-Zn_4_ **Im**_8_H**Im** material, an interrupted dense framework of the **moc** topology, where one-quarter of the **Im** ^–^ linkers are replaced with an [**Im**···H···**Im**]^−^ unit, is also the predominant product of the control NG reactions, as has recently been shown during an *in situ* Raman monitoring study.^97^ Here we show that it appears as a product in both steel and Teflon jars, after 15, 30, or 60 min of milling (Figure S1). Only the 60 min milling reaction in a steel jar provides **zni**-Zn**Im**_2_ alongside **moc**-Zn_4_ **Im**_8_H**Im**. The **moc**, **zni** and **coi** phases also appear as products of heating of the less dense phases (Figure S42), or after long periods of room temperature (RT) aging (Figures S14, S50), hinting at their higher thermodynamic stability compared to the more open phases. Previously, the **moc**, **zni** and **coi** phases have mostly been synthesized from ionic liquids,^39^ thermally in the solid state,^40^ or by conversion from more open forms.^96^

Overall, these observations lead us to conclude that these are the thermodynamically preferred structures when no effective pore-filling guest is available to stabilize a more open Zn**Im**_2_ topology. Any selectivity toward generating these structures in the presence of liquid additives is therefore likely due to surface processes, or transient templation effects in the early stages of ZIF nucleation, where the liquid preorganizes the reagents but is not encapsulated in the product. In the LAG screening protocols, the protic liquids (H_2_O, MeOH, EtOH) seem to steer the reaction toward the **zni**-Zn**Im**_2_ phase, while polar aprotic liquids (acetone (AcMe), dimethyl sulfoxide (DMSO), tetrahydrofuran (THF)) favor the **coi**-Zn**Im**_2_ phase. These two phases also appear concomitantly, in mixtures upon using certain liquid additives (ethylene glycol (EtGly), H_2_O, MeCN), which is unsurprising since they have been otherwise shown to interconvert under different pressures and temperatures.^23,96^

### 2.3 Lower Density Forms of ZnIm_2_

The next most prevalent Zn**Im**_2_ topologies to appear in the LAG screening are **cag** (7/45 liquids, void fraction 6.9%) and **neb** (7/45, void fraction 8.1% for **neb1** and 6.0% for **neb2**), which have theoretical activated densities between 1.0 and 1.3 g/cm^3^. These both predominantly appear using aliphatic cyclic molecules with 5- or 6-membered rings as additives and structure-directing agents. The framework **cag-**Zn**Im**_2_ appears to be preferred with more polar molecules (e.g., tetrahydropyran (THP), cHONE, THF), while **neb-**Zn**Im**_2_ materials appear mostly using nonpolar and nitrogen-containing additives (e.g., 1,4-dioxane (DIOX), MORPH, cHANE). Both topologies comprise zero-dimensional (0D) pores (cavities) well suited by shape for cyclic small molecules, such as those yielding these materials in the LAG screening (bottom panel of Figure 3 shows the **crb** 0D cages). As mentioned before, using cHANE as the liquid additive leads to both the **cag** and the **neb** form depending on the reaction conditions, with cHANE@**cag-**Zn**Im**_2_ being favored in Teflon milling jars and with shorter milling times. Based on literature precedent,^56^ this implies that cHANE@**cag-**Zn**Im**_2_ is less thermodynamically stable than cHANE@**neb1-**Zn**Im**_2_ and thus transforms into it upon prolonged milling.

**Figure 3:**
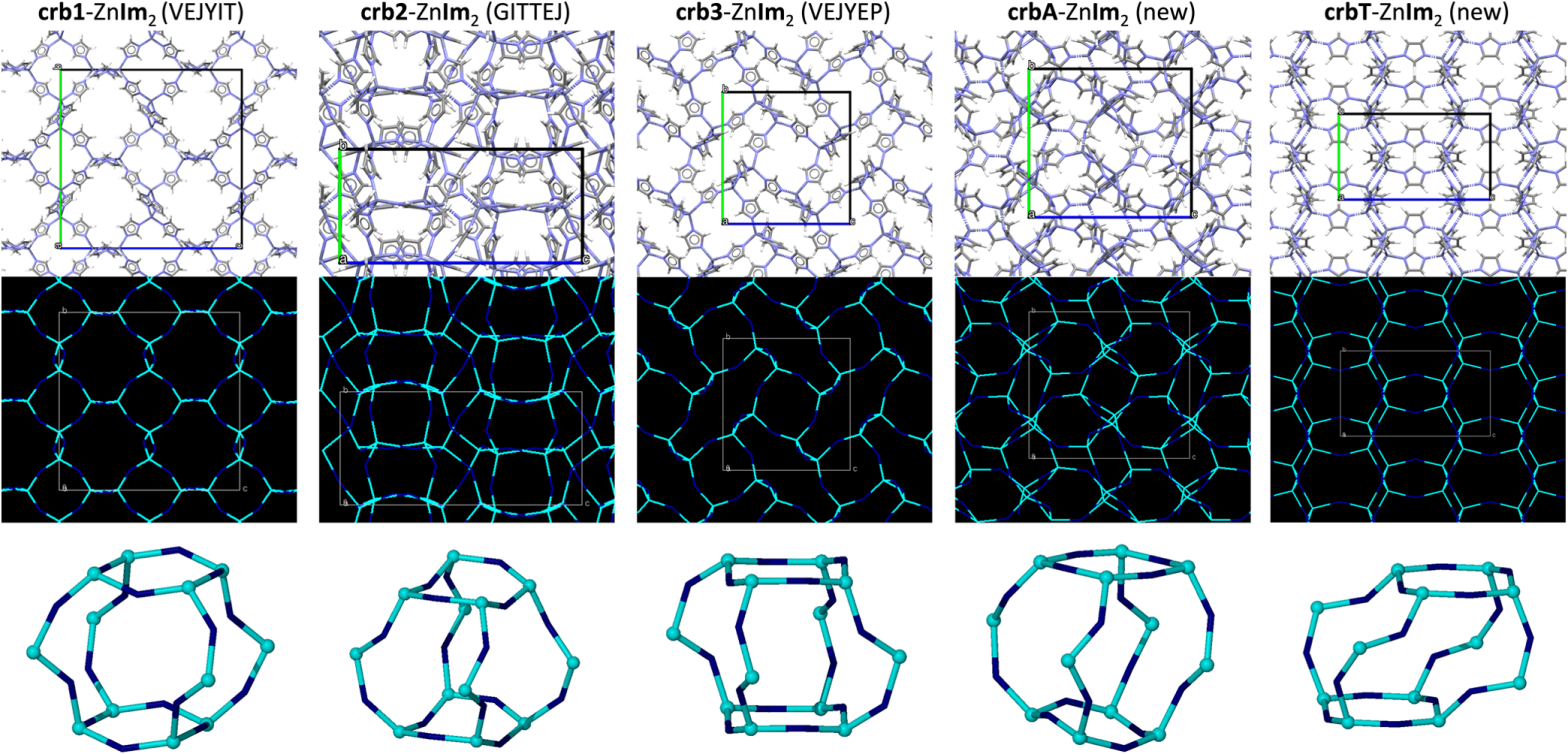
Graphical representations of the **crb1**, **crb2**, **crb3**, **crbA** and **crbT** forms of Zn**Im**_2_. Top row: The crystal structures viewed along the *a* axis and presented through capped sticks representations. The unit cells are drawn in black, and any present guests have been removed from the structure. Middle row: The crystal packing represented in a reduced node-and-linker image, where zinc ions are depicted in light blue, whereas the centroids of imidazolate ions are depicted in dark blue. Bottom row: The **crb** building cages of each structure depicted through the reduced node-and-linker image.

The **neb-**Zn**Im**_2_ topology appears in two forms, the previously mentioned **neb1**, and the **neb2** form. These two forms exhibit the same metal–ligand–metal connectivity, but due to the flexibility of the Zn**Im**_2_ framework (resulting from changes in the relative orientations of **Im** ^–^ ligands) they have different pore sizes and shapes (Figure 6), which leads to a difference in porous properties. For example, the **neb** cage building block of **neb1** is larger than that in **neb2**, resulting in a higher void fraction (8.1% in **neb1**, vs 6.0% in **neb2**, Table S9), and higher calculated surface area (237 m^2^/g in **neb1**, vs 1 m^2^/g in **neb2**, Table S9).

Of the liquid additives studied, only PYR leads to the appearance of a **neb2**-Zn**Im**_2_ solvate, which is notable since PYR (in a mixture with ethanol) is also the solvent originally used for the solvothermal synthesis of this form.^96^ While this suggests a templating effect that is transferable from solution to the solid state, it might also indicate a particularly strong stabilization of this framework form upon encapsulation of PYR. Conversely, **neb1**-Zn**Im**_2_ solvates are found using a wide range of liquid additives, including thiophene (TPH), *N*-methylpyrrolidine (NMPl), and the six-membered aliphatic liquids (cHANE, DIOX and cyclohexene (cHENE)), including MORPH – the solvent used (in a mixture with ethanol) for the original solvothermal preparation of 0.5MORPH@**neb1**-Zn**Im**_2_.^95^ As mentioned, the **neb** cage building block of **neb1** is larger than that in **neb2**, which could explain the greater flexibility with regard to guest encapsulation, as it can potentially fit more sterically demanding guests than **neb2**. On the other hand, the **neb** cage in **neb2** is narrower (maximum pore diameter, *d*_max_^pore^ = 4.05 Å, Figure 6, Table S9) than in **neb1** (*d*_max_^pore^ = 4.97 Å), possibly explaining the need for planar aromatic pyridine. Interestingly, other than the mentioned polar cyclic molecules, **cag**-Zn**Im**_2_ is additionally templated by *N*,*N*-dimethylformamide (DMF), the solvent used (in a mixture with propylamine) for its original solvothermal synthesis^47^ and chloroform (CHCl_3_). Both the **neb** and **cag** topologies thus demonstrate not only the possibility of transferring knowledge gained from solvothermal syntheses into mechanochemical synthesis, but also showcase the much broader toolbox of presumptive templates at hand when employing mechanochemistry, where reagent solubility and/or liquid miscibility are not limiting factors for experimental design.

Following the **neb** and **cag** topologies, the **crb** family of frameworks appears when using 11 out of the 45 herein explored presumptive liquid templates. So far, three forms of **crb**-Zn**Im**_2_ have been reported in the literature: ZIF-2 (1.5DMF@**crb1**-Zn**Im**_2_, CSD code VEJYIT^27^), ZIF-64 (0.55DMF@**crb2**-Zn**Im**_2_, CSD code GITTEJ^35^), and ZIF-1 (0.5DMA@**crb3**-Zn**Im**_2_, CSD code VEJYEP,^27^ DMA = *N*,*N*-dimethylacetamide). These frameworks were all prepared solvothermally from DMF under different conditions (see Table S2). Despite possessing the same Zn-**Im-**Zn connectivity and thus the same topology, these three **crb** forms adopt different space groups, unit cell parameters, predicted surface areas and pore sizes (Table 1), revealing there is considerable flexibility of the **crb**-Zn**Im**_2_ framework. This flexibility is a direct result of the conformational flexibility of the metal–ligand–metal linkages, which allows for large differences in the shapes of the **crb**-cage building blocks for the different **crb** forms (Figure 3). This makes **crb1**-, **crb2**- and **crb3**-Zn**Im**_2_ essentially different materials for potential porosity application purposes.

During LAG screening, pure samples of **crb3-**Zn**Im**_2_ were successfully prepared by addition of DMA, oxetane (OXT), *N*-methyl-2-piperidone (NMPd) and *N*-methyl-2-pyrrolidone (NMP). Interestingly, the crystal structure of **crb3-**Zn**Im**_2_ deposited in the CSD (VEJYEP) contains DMA, despite the nominal crystallization solvent being DMF, indicating a potential preference of the framework for this guest. The **crb1**-Zn**Im**_2_ and **crb2**-Zn**Im**_2_ materials were prepared in our screening only as components of mixtures, using PYR (mixture with **neb2**-Zn**Im**_2_, SI 2.1.42.) and hexamethylphosphoramide (HMPA, mixture with **crb3**-Zn**Im**_2_, SI 2.1.24.), respectively, as liquid additives. Since **crb1-**Zn**Im**_2_ and **crb2-**Zn**Im**_2_ have only been prepared as single crystals in high-throughput solvothermal experiments, it is not surprising that the mechanochemical approach is also only partially successful in their synthesis, providing them solely in mixtures with other forms. These two phases are also the least dense **crb** phases published to date (Table 1; void fractions: **crb1** – 23.4%, **crb2** – 11.9%, **crb3** – 11.0%), and may in the future be prepared pure using larger guests, especially in the case of **crb1**.

### 2.4 New Forms of crb-ZnIm_2_

In further screening, mechanochemical reactions with added toluene (PhMe) and acetophenone (AcPhe) reproducibly gave phase-pure products whose PXRD patterns did not match any of the already known ZIF forms. Attempts at solution-based syntheses of single crystals of these phases (SI-1.4.) were unsuccessful, potentially due to solubility constraints. We therefore resorted to crystal structure solution from PXRD data, assisted by periodic DFT calculations (see Materials and Methods, and SI-1.5., 1.6.). The new phases were found by ToposPro^98^ and TopCryst^99^ to both have the **crb** topology and were designated **crbT-**Zn**Im**_2_ (from PhMe, **T**oluene) and **crbA-**Zn**Im**_2_ (from AcPhe, **A**cetophenone). Their guest content was found from Rietveld refinement (0.88 PhMe and 0.49 AcPhe, respectively), by thermogravimetric analysis (TGA; 0.68 PhMe and 0.49 AcPhe, respectively), and by solution NMR (0.62 PhMe and 0.52 AcPhe, respectively). Heavy guest disorder makes determination of guest amount by Rietveld refinement less reliable in this case, so the average values of the NMR and TGA quantifications were used to give the final compositions of 0.65(3)­PhMe@**crbT-**Zn**Im**_2_ and 0.51(2)­AcPhe@**crbA-**Zn**Im**_2_. Both 0.65PhMe@**crbT-**Zn**Im**_2_ and 0.51AcPhe@**crbA-**Zn**Im**_2_ are crystallographically distinct from the three previously reported **crb** phases and have different calculated pore properties (Table 1). For example, **crbT** has the second highest calculated void fraction (14.7%) and per-zinc unit cell volume (321.4 Å^3^), while **crbA** has the second highest calculated maximum pore diameter (6.40 Å) of the known **crb** phases.

The physicochemical properties of these new solid forms are also different; 0.51AcPhe@**crbA-**Zn**Im**_2_ conserves its structure upon extensive washing with acetone (Figure S3) without guest exchange (based on NMR, Figure S61), while exposing 0.65PhMe@**crbT-**Zn**Im**_2_ to acetone for even short periods of time results in a transformation into an unknown phase (Figure S42), presumably due to framework collapse upon guest exchange. Heating 0.65PhMe@**crbT-**Zn**Im**_2_ at mild temperatures (40 or 60 °C) results in the slow transformation to yet another unknown phase (Figure S43) with simultaneous loss of toluene (Figure S60a), and heating 0.65PhMe@**crbT-**Zn**Im**_2_ at 120 °C results in collapse into a mixture of the dense **coi-** and **zni-**Zn**Im**_2_ phases (Figure S42). Conversely, heating 0.51AcPhe@**crbA-**Zn**Im**_2_ shows no observable loss of guest on the TGA until at least 100 °C (Figure S60b), yet heating the same material at temperatures as low as 40 °C results in at least partial transformation to the **crb3-**Zn**Im**_2_ phase based on PXRD (Figures S3, S4). The transformation of 0.51AcPhe@**crbA-**Zn**Im**_2_ to the **crb3-**Zn**Im**_2_ phase is very slow at lower temperatures, but is finished within 3 h at 105 °C (Figure S3). Heating 0.51AcPhe@**crbA-**Zn**Im**_2_ at 150 °C results in a significant mass loss visible on the TGA (Figure S60b) and the appearance of a novel phase (presumably unknown-Zn**Im**_2_) characterized by low-angle peaks in the PXRD pattern, indicating a large unit cell (Figure S3). Preliminary N_2_ sorption studies at 77 K (Figure S67a,c,e) show that unknown-Zn**Im**_2_ is porous (Brunauer–Emmett–Teller surface area, SA_BET_ = 585 m^2^/g). Structure-determination studies are underway.

The thermally induced transformations of 0.51AcPhe@**crbA**-Zn**Im**_2_ and 0.65PhMe@**crbT**-Zn**Im**_2_ make porosity analysis challenging, leaving room temperature (RT) activation as one of the few potential avenues. Unfortunately, even long exposure to high vacuum at RT was not sufficient to adequately activate these materials. In the case of 0.51AcPhe@**crbA**-Zn**Im**_2_, the sample shows partial conversion into a **crb3**-Zn**Im**_2_ phase after only 18 h of RT vacuum treatment (Figure S68). In the case of 0.65PhMe@**crbT**-Zn**Im**_2_ the sample remains intact even after 60 h under high vacuum at RT, but gives an isotherm characteristic of nonporous materials (SA_BET_ = 27 m^2^/g, Figure S67b,d). The challenge of activating the new **crb**-Zn**Im**_2_ phases is likely due to the zero dimensional nature of the pores in the **crb** family, as lack of proper 1D or 2D channels induces a significant kinetic barrier to the desolvation of these frameworks, leaving them occupied with guest even after a long RT activation. It is however encouraging that 0.65PhMe@**crbT**-Zn**Im**_2_ remains intact after vacuum treatment, and that 0.51AcPhe@**crbA**-Zn**Im**_2_ transforms into a porous phase, so we plan on further exploring these systems in detail.

It is important to note that low-intensity reflections characteristic of unreacted ZnO (∼32.1, 34.6, and 36.5° 2θ) were observed in the PXRD patterns of the synthesized **crbT** material. This is likely due to the formation of a core–shell system where the Zn**Im**_2_ grows on a ZnO core, as seen by Tanaka et al.^63^ In principle, it should be possible to convert the remaining ZnO to the ZIF product by adding ionic catalysts,^100^ additional milling, or by using nanoparticulate ZnO as a reagent.^63^ Indeed, adding 5 wt % (compared to ZnO) of zinc acetate dihydrate to the reaction mixture allows full conversion into 0.65PhMe@**crbT**-Zn**Im**_2_ after 1 h of milling (Figure S44). Conversely, while the use of nanoparticulate zinc oxide as a reagent does slightly diminish the amount of leftover zinc oxide (3.8(3)% using regular ZnO and 1.7(2)% using nanoparticulate ZnO, Section S2.10.), it does not lead to full conversion (Figure S44).

The **crbA** phase was also found through LAG with 4-acetylpyridine (4AcPyr, Figure S5), and the **crbT** phase was also produced by using anisole (PhOMe, Figure S45) and 4-methylpyridine (4-MePyr, Figure S30) as additives in the LAG reactions. It appears therefore that aromatic compounds with a single unbranched substituent favor the **crbT** phase, while a larger substituent on the aromatic core is needed to produce the **crbA** phase. This matches well with the larger calculated maximum pore diameter in 0.51AcPhe@**crbA-**Zn**Im**_2_ (6.40 Å, compared to 4.75 Å in 0.65PhMe@**crbT-**Zn**Im**_2_), despite the larger void fraction in 0.65PhMe@**crbT-**Zn**Im**_2_ (14.7% compared to 12.0% in **crbA**). With the addition of these new forms, the **crb** topology now has an unprecedented five crystallographically unique forms with different physical properties. Further research on the **crbA** and **crbT** phases, and especially their thermal transformations and the new phases thus produced is needed, but it is already evident that they have new and potentially interesting properties.

### 2.5 Preparation of Lowest-Density ZnIm_2_ Phases

Finally, the topologies that appeared least often in the LAG screening were also the least dense ones (theoretical activated densities between 0.85 and 1.15 g/cm^3^), namely **nog-**Zn**Im**_2_ (4/45 liquids, void fraction 15.8%), **10mr-**Zn**Im**_2_ (1/45 liquids, void fraction 20.5%) and **afi**-Zn**Im**_2_ (1/45 liquids, void fraction 37.1%). Low-intensity X-ray reflections of leftover ZnO were present in the PXRD patterns of all three phases. This challenge could likely in the future be addressed similarly to the case of **crbT**, by adding a catalytic amount of zinc acetate. Since these materials are all reported to be highly porous, it is also possible that part of the imidazole reagent was absorbed into the ZIF channels and thus was unavailable for the reaction, lowering the yield. If so, using a larger amount of the pore-filling liquid, or an excess of imidazole might also provide a higher yield.^72^

The **nog-**Zn**Im**_2_ phase appeared immediately, without side products, only using *N*-methylpiperidine (NMPP, Figure S38), but was also observed to appear from the amorphous phase produced by milling with *N*,*N*-diethylformamide (DEF, Figure S13) after being aged for 12 days in air at room temperature. The **10mr** phase was only found by LAG with DBF, the solvent from which it was originally prepared,^43^ with larger amounts of solvent (either 167 or 200 μL, compared to the standard 100 μL) needed to avoid formation of the **moc**-Zn_4_ **Im**_8_H**Im** side-product. Even then, in some repetitions of the synthesis, mixtures of products were obtained (Figure S12), potentially influenced by ambient conditions such as temperature, humidity, or other factors, so further work on this system is needed in the future. Similarly, the **afi** phase was prepared by LAG with *N*,*N*-dipropylformamide (DPF), the solvent from which it was originally prepared,^45^ with 200 or 300 μL of DPF (compared to the standard 100 μL) needed to avoid formation of the dense **moc** phase (Figure S14). It is important to note that, while the original solvothermal reactions to produce **10mr**-Zn**Im**_2_ and **afi**-Zn**Im**_2_ required 3 days of heating the reagents in DBF at 50 °C or DPF at 60 °C, respectively, we were able to prepare 200 mg of phase-pure product using only 167 μL of DBF or 200–300 μL of DPF in 1 h of ball-milling. This demonstrates that our LAG screening is not only highly successful at synthesizing different Zn**Im**_2_ phases, but can also provide faster, inexpensive, more efficient, and more environmentally friendly methods of MOF synthesis, once fully optimized.

### 2.6 Direct Preparation of Amorphous ZnIm_2_

In addition to the presented crystalline phases, we also observed several cases of amorphous phases being directly synthesized by LAG. Specifically, 30 min of milling with *N*-methylcaprolactam (NMC, Figure S33) or *N*-methyl-2-piperidone (NMPd, Figure S35), as well as 60 min of milling with DEF (Figure S13) or NMPd gave amorphous products, presumably *a*-Zn**Im**_2_, with small amounts of residual ZnO detectable by PXRD analysis. The reaction in the presence of NMC as the liquid additive was reproducible, with the amorphous phase persisting for a minimum of 7 days, but ultimately found to convert into unidentifiable crystalline phases after longer standing in air at room temperature. The amorphous products from DEF converted into the *x*DEF@**nog**-Zn**Im**_2_ phase in less than 14 days of standing under ambient conditions. The stability of amorphous phases resulting from the reactions with NMPd varies, with some phases being stable for at least 20 days, while others started converting within 4 days. The variability is potentially due to subtle changes in the laboratory environment, or even the presence of seeds of different ZIFs. We attempted to remove NMPd from the amorphous product of the LAG reaction by washing the material in acetone, followed by heating in a vacuum oven, to see if the removal of the directing agent would affect the stability of the amorphous phase (Figure S36). Heating for 1 h at 100 °C under vacuum immediately resulted in crystallization of the sample into what appears to be a mixture of **zni**-Zn**Im**_2_ and **crb2**-Zn**Im**_2_ phases according to PXRD analysis. Washing the sample with acetone preserved the amorphous phase at first, but then resulted in crystallization to the **coi**-Zn**Im**_2_ phase within 1 day. In contrast, the parent amorphous phase was found to start crystallizing into the **crb2**-Zn**Im**_2_ phase only after 4 days of standing at room temperature. These experiments indicate some degree of stabilization being provided to the amorphous phase by the presence of the liquid additive, perhaps similar to the stabilization that bulkier ligands can sometimes provide to amorphous ZIFs, ostensibly by preventing their crystallization into thermodynamically more stable dense phases.^101,102^

Most syntheses of amorphous ZIFs involve first making a crystalline ZIF material, and then amorphizing it via heating, pressure or mechanochemistry,^38^ wherein postsynthetic mechanochemical treatment can allow for the preparation of glassy ZIFs, even in some cases where quench melting fails due to ZIF decomposition.^103^ It was recently also shown that mechanochemically synthesized ZIFs can exhibit lower glass transition temperatures than those prepared solvothermally, presumably due to the introduction of defects, enabling the synthesis of otherwise inaccessible ZIF glasses.^104^ Direct syntheses of amorphous ZIFs, however, are much less common. To the best of our knowledge, there are at least two other such syntheses reported to date. One used 2-methylimidazole (**HMeIm**) and included encapsulation of the glucose oxidase enzyme from solution,^105^ while the other produced amorphous ZIF-62 using a combination of mechanochemical synthesis and increased ratio of bulky benzimidazolate ligand in the ZIF.^102^ As amorphous ZIFs have been the target of extensive studies and have potential applications,^36–38,105–108^ their direct, rapid synthesis is of interest.

### 2.7 Periodic DFT Calculations on Empty Frameworks

While LAG screening enabled a quick and efficient preparation of 14 different crystalline ZIFs as well as amorphous Zn**Im**_2_ phases, and greatly expanded the scope of potential structure-directing agents in ZIF syntheses, the underpinnings of the presumptive templation effect remain unclear. We propose that the use of periodic DFT calculations will provide insight into the structure-directing process, and hopefully enable us to later conduct targeted LAG templation of specific desired MOF topologies. Periodic DFT has already been employed to elucidate the mechanochemistry of ZIFs, showing that polymorphic transformations of Zn­(**MeIm**)_2_ and Zn­(**EtIm**)_2_ (**EtIm** = 2-ethylimidazole) inside the mill follow the Ostwald rule of ripening, proceeding from the more open, thermodynamically less stable phases, toward the more stable, denser forms.^56^ Furthermore, periodic DFT correctly surveyed the topological landscape of experimentally unknown ZIFs, in both a ligand-replacement experiment^83^ and in the true crystal structure prediction of hypergolic ZIFs,^84^ allowing for their later mechanochemical synthesis. It has also been broadly used in assessing the relative stability of Zn**Im**_2_ polymorphs,^23,96,109–112^ including generating hypothetical future ZIFs based on zeolite topologies.^111–113^ Some of these have then been synthesized, including the AFI and CAN topologies predicted in 2009 ^111^ and synthesized in 2016,^45^ and the ATN topology predicted in 2009 ^111^ and not synthesized until 2021.^44^ All three topologies were prepared only by use of appropriate structure-directing agents (DPF for AFI and CAN, and DBF with *N*-butylamine for the ATN phase), emphasizing their importance. Despite this, the vast majority of Zn**Im**_2_ DFT modeling has only considered empty frameworks, discounting interactions with guests/presumptive templates. We therefore decided to pursue periodic DFT modeling of the frameworks obtained using LAG screening in their empty forms, and select guest-filled frameworks.

Several of the prepared Zn**Im**_2_ frameworks, namely the **cag**,^109,111,112^ **crb1**,^111^ **crb3**,^111^ **neb2**,^112^ **coi**,^96^ and **zni** ^96,111,112^ frameworks, had previously been modeled by periodic DFT calculations. It was shown that dispersion correction can have a crucial effect on the accuracy of energy rankings of ZIF polymorphs, and the Perdew, Burke and Ernzerhof (PBE) functional^114^ combined with Grimme D2 ^115^ or D3 ^116^ semiempirical dispersion correction (PBE-D3) was found to best match the experimental crystallographic parameters.^109^ D2 and D3 corrections appeared to perform similarly, though it was noted that the D2 correction tends to overestimate the interaction energies, an effect known as overbinding.^116^ In ZIFs made from different ligands (such as H**MeIm** and H**EtIm)**, PBE with Grimme D2 and, later, many-body dispersion (MBD*) correction also reproduced well the relative experimental energies of several ZIF topological forms.^56,82–84^ Furthermore, the performance of the PBE functional with TS, D3 and MBD* corrections was evaluated for the transformation of ZIF polymorphs to a ZIF carbonate phase upon exposure to carbon dioxide, and MBD* and D3 corrections showed best overall agreement with experimental energies determined from dissolution calorimetry.^117^ We have ultimately decided to use the PBE-D3 method over PBE-MBD*, given their similar accuracy, but lower computational cost of the PBE-D3 method. All periodic DFT calculations were performed in the CASTEP^118^ plane-wave DFT code.

We first performed geometry optimizations for the guest-free versions of all 14 zinc imidazolate frameworks obtained through LAG screening (Figure 4).

**Figure 4:**
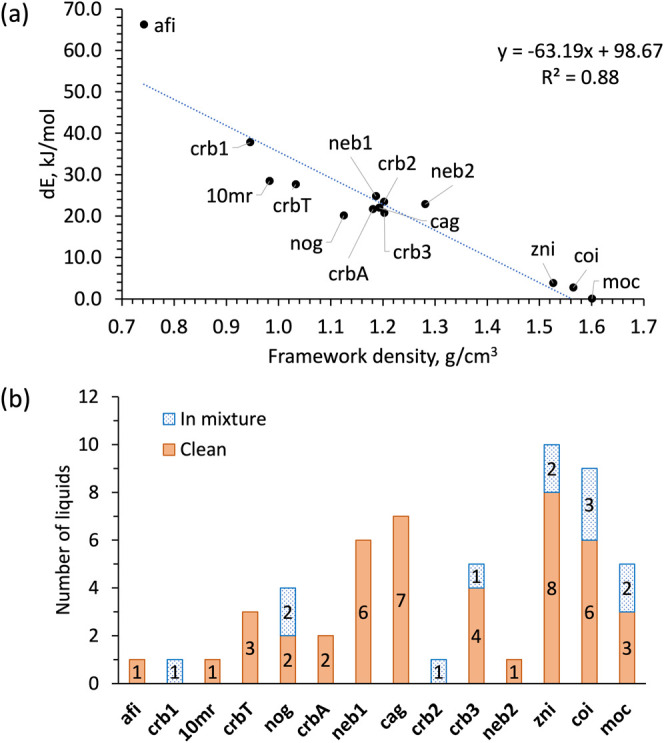
(a) Comparison of the calculated energies of different topology empty Zn**Im**_2_ frameworks and their densities. The reference point is the adjusted energy of **moc**-Zn_4_ **Im**_8_H**Im**, and all energies are scaled per zinc atom. (b) Number of liquids providing each topology in LAG screening, phase pure (orange bar) or as part of a mixture (blue bar). If a liquid provides the same topology both phase pure (“clean”) and in a mixture, it is only added to the “clean” number. The topological forms are listed in order of descending relative calculated energy.

The starting atom coordinates were obtained from the CSD, or via structure solution from PXRD data (**crbA** and **crbT** phases). All guests (if present) were removed from the frameworks, and disorder (if present) was resolved into components, all of which were separately optimized. Full details of all optimizations can be found in SI-1.6. and SI-2.9. The lowest energy structure was found to be the **moc**-Zn_4_ **Im**_8_H**Im** material, which is also the product of the neat mechanochemical reaction between ZnO and H**Im**. Above it in energy are the topological polymorphs of Zn**Im**_2_ and their different forms, whose energies are reported relative to the **moc** phase (after accounting for the additional terminal imidazole molecule in **moc**-Zn_4_ **Im**_8_H**Im**).

As previously reported,^109,111^ the calculated energies of the optimized empty phases are roughly linearly proportional to their densities (Figure 4a.). Thus, **moc**-Zn_4_ **Im**_8_H**Im** is the global minimum, followed closely by **coi-**Zn**Im**_2_ (Δ*E*_moc_ = 2.66 kJ/mol) and **zni-**Zn**Im**_2_ (Δ*E*_moc_ = 3.76 kJ/mol). The very small calculated difference in energy for the **coi** and **zni** phases (Δ*E*_zni‑coi_ = 1.10 kJ/mol) likely explains the prevalence of their concomitant appearance in the LAG screening, and the reported ease of interconversion among them.^23,96^ The Δ*E*_zni‑coi_ value also matches fairly well with the experimentally obtained value of the enthalpy of polymorphic transition between these two phases at 360 °C (Δ*H*_zni‑coi_ = 2.9(1) kJ/mol).^96^ Not surprisingly, these three most stable phases also appear most often in the LAG screening (Figure 4b).

The next most stable phases belong to the **cag**, **neb** and **crb** topologies, almost all clustering in the 20–25 kJ/mol range of relative energies. Together, these phases are produced as phase pure products by 20 out of the 45 liquids used. Notable outliers from the observed relative energies in the **crb** topology are the **crb1** and **crbT** phases. The **crb1** phase has the second highest calculated relative energy (Δ*E*_moc_ = 37.77 kJ/mol) of all obtained phases, and it is therefore unsurprising that it only shows up once in the LAG screening (with PYR), and only as part of a mixture (with the **neb2** phase). The newly found **crbT** form also has a high relative energy (Δ*E*_moc_ = 27.63 kJ/mol), significantly larger than the **crbA** form (Δ*E*_moc_ = 21.61 kJ/mol). On the other hand, **crbA-**Zn**Im**_2_ is very close in energy (Δ*E*_crbA‑crb3_ = 0.90 kJ/mol) to **crb3-**Zn**Im**_2_ (Δ_moc_ = 20.71 kJ/mol), which may explain their aforementioned low-temperature (as low as 40 °C) interconversion.

The highest relative energies of the ZIFs found through mechanochemical screening belong to the most porous phases, **afi**-Zn**Im**_2_ (Δ*E*_moc_ = 66.18 kJ/mol) and **10mr**-Zn**Im**_2_ (Δ*E*_moc_ = 28.42 kJ/mol). That these frameworks can be experimentally observed during mechanochemical templation, even with an energy difference of more than 65 kJ/mol above the most stable phases (for **afi**) is quite extraordinary. It indicates a significant kinetic effect and/or a high degree of stabilization of the low density framework by the encapsulated guest. For reference, the observed energy difference between the highest and lowest energy forms in a mechanochemical reaction for ZIF frameworks based on other ligands is 10.6 kJ/mol for H**MeIm**,^56^ 17.6 kJ/mol for H**EtIm** ^56^ and 15.6 kJ/mol for 2-trifluoromethylimidazole.^83^ Moreover, the experimentally achievable Δ*E*_moc_ value could be even higher, as we have previously achieved the mechanochemical synthesis of RHO-Zn**Im**_2_ (density = 0.63 g/cm^3^ compared to 0.74 g/cm^3^ in **afi**-Zn**Im**_2_) by employing a designer, shape-persistent macrocyclic template.^86^ Similarly, it has been shown that low density hydrogen bonded organic frameworks (HOFs) more than 50 kJ/mol in energy above the global minimum structure are experimentally accessible due to their stabilization by pore solvation.^119^

### 2.8 Periodic DFT Calculations on Guest-Occupied Frameworks

We therefore decided to further explore the energetics of the LAG reactions by selecting several of the prepared frameworks and inserting additive molecules into their pores and cavities. Guest-filled structures were found or adapted from the CSD, in-house single crystal X-ray diffraction data, or solved PXRD crystal structures (see SI-2.9.3.). Then, the *x*guest@*y*Zn**Im**_2_ composites were geometry optimized and their energies (*E*(*x*guest@*y*Zn**Im**_2_)) were determined, taking into account the guest:framework stoichiometric ratio (*N.B.* these are nondynamic 0K calculations). Separately, the guest molecules were geometry optimized in a large (*Lx* = *Ly* = *Lz* = 25 Å) box simulating the gas phase, and their energies (*E*(guest)) were obtained. Full details can be found in SI-1.6 and SI-2.9. Subtracting the energies of the guest molecules (adjusted for stoichiometry) provides the energies of the Zn**Im**_2_ framework when it is occupied by guest (*E*_occup_(Zn**Im**_2_)), according to eq 1.

1 $$E_{\mathrm{occup}}\left(\mathrm{Zn}Im_{2}\right)=\left[E\left(x\mathrm{guest}@y\mathrm{Zn}Im_{2}\right)-\mathrm{xE}\left(\mathrm{guest}\right)\right]/y$$

Comparison with the energies of the empty Zn**Im**_2_ frameworks (*E*_empty_) provides the stabilization energy Δ*E*_gas_ that can be attributed to the guest being absorbed from the gas-phase (eq 2).

2 $$\Delta E_{\mathrm{gas}}=E_{\mathrm{occup}}-E_{\mathrm{empty}}$$

To account for the fact that the used liquid additives are not in the gas phase, the obtained energies were adjusted for experimental enthalpies of evaporation according to eq 3, providing the stabilization energy Δ*E*_liquid_ that can be attributed to the guest being absorbed from the liquid phase

3 $$\Delta E_{\mathrm{liquid}}=\Delta E_{\mathrm{gas}}+x/yE_{\mathrm{vap}}\left(\mathrm{guest}\right)$$

Arguably, the true stabilization energies are somewhere between Δ*E*_gas_ and Δ*E*_liquid_, as many used liquids are volatile, and would be partially in the gas phase, especially under the highly dynamic milling conditions. In this case, Δ*E*_liquid_ is likely to be more representative of the experimental reality, and we will refer to those values throughout the discussion. Figure 5 shows the dependence of the calculated energies for the empty frameworks of topologies found in our LAG screen, as well as the selected guest-occupied frameworks, on their density.

**Figure 5:**
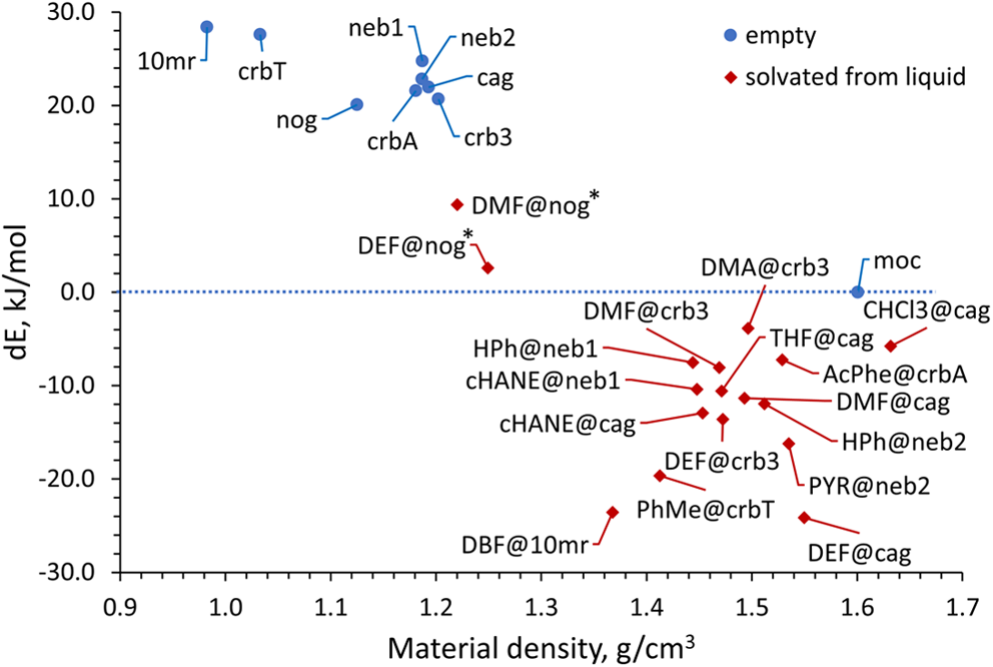
Dependence of the calculated relative energy for different topology empty Zn**Im**_2_ frameworks (*E*_empty_, blue circles) and guest-occupied Zn**Im**_2_ frameworks (*E*_occup_, red rhombuses) on their density. The reference point is the adjusted energy of **moc** topology framework (blue dotted line), and all enthalpies are scaled per zinc atom. The two **nog** topologies are marked with an asterisk due to potential issues with the starting structure.

The first observation is that almost all of the guest-occupied Zn**Im**_2_ frameworks investigated by periodic DFT calculations exhibit relative energies lower than the most stable empty framework, **moc-**Zn_4_ **Im**_8_H**Im**. The only exceptions are the two calculated **nog** topology solvates, 0.2DMF@**nog**-Zn**Im**_2_ (DMF@**nog**, Δ*E*_moc, DMF_ = 9.38 kJ/mol) and 0.2DEF@**nog**-Zn**Im**_2_ (DEF@**nog**, Δ*E*_moc, DEF_ = 2.59 kJ/mol). In our screening, the *x*DMF@**nog**-Zn**Im**_2_ phase has not yet been observed, while the *x*DEF@**nog**-Zn**Im**_2_ phase appears, but only by slow transformation from one of the amorphous phases. The DEF@**nog** phase is significantly lower in energy than the corresponding DMF phase (Δ*E*_DMF‑DEF_ = 6.79 kJ/mol), and much closer to **moc-**Zn_4_ **Im**_8_H**Im**, so it is unsurprising that of the two, the DEF@**nog** phase is the only one experimentally observed. Even though both **nog** phases are significantly stabilized compared to the empty framework (by 17.47 and 10.68 kJ/mol for DEF and DMF, respectively), visual inspection of the optimized structures indicates that a significant volume of unoccupied space is still present (10.5% solvent accessible void space, or 613.6 Å^3^ per unit cell). It is possible that the single crystal from which the crystal structure of 0.2DEF@**nog**-Zn**Im**_2_ was originally determined was only partially solvated, and that the maximum amount of DEF that can be absorbed into **nog**-Zn**Im**_2_ is actually higher. If the leftover void space were filled with more DEF, the stabilization would no doubt be greater, and it is likely that the energy of the *x*DEF@**nog**-Zn**Im**_2_ structure would also fall below that of the **moc** phase global minimum. In the future we aim to obtain the fully occupied DEF@**nog** structure and, more generally, investigate the dynamical effects of the absorption of small-molecule guests into ZIF voids, therefore diminishing our reliance on published crystal structures.

For all the other optimized solvated forms of Zn**Im**_2_, the energy stabilization effects of the templates are even more pronounced. For **10mr-**Zn**Im**_2_, the stabilization upon inclusion of DBF, compared to the empty framework, is 51.96 kJ/mol, bringing the 0.4DBF@**10mr**-Zn**Im**_2_ (DBF@**10mr**) material to 23.58 kJ/mol below **moc-**Zn_4_ **Im**_8_H**Im**. Looking at the geometry optimized crystal structure of DBF@**10mr**, not only is the formerly empty space within the framework now completely filled (void fraction 0.1%), but the DBF molecules are connected to the framework imidazolates by a series of (imidazolate) C–H•••O (amide) hydrogen bonds (Figure S91). This abundance of intermolecular van der Waals interactions and C–H•••O hydrogen bonds not only stabilizes the framework, but also potentially indicates a true templation effect of DBF molecules in the synthesis of DBF@**10mr**. Namely, the directionality of the C–H•••O hydrogen bonds could serve as a way to preorganize imidazol­(at)­e molecules around the DBF structure-directing molecules in the early stages of the reaction, so that the **10mr** topology is highly favored as a product.

A similar degree of stabilization is achieved for the **crbT** phase when it is occupied by toluene. The 0.65PhMe@**crbT**-Zn**Im**_2_ (PhMe@**crbT**) phase is 47.24 kJ/mol lower in energy than the empty **crbT**-Zn**Im**_2_ phase, and 19.64 kJ/mol more stable than **moc-**Zn_4_ **Im**_8_H**Im**. In this case, the stabilization is almost exclusively due to space-filling (void fraction is 0.0%) and van der Waals interactions, as toluene is unable to form hydrogen bonds with the framework. In comparison, the stabilization of the **crbA** phase is significantly smaller. 0.51AcPhe@**crbA**-Zn**Im**_2_ (AcPhe@**crbA**) is 20.24 kJ/mol more stable than the empty framework, and only 7.22 kJ/mol more stable than **moc-**Zn_4_ **Im**_8_H**Im**.

Analysis of the **neb** topology Zn**Im**_2_ materials reveals that the 0.5PYR@**neb2**-Zn**Im**_2_ (PYR@**neb2**) material is stabilized by inclusion of pyridine (Δ*E* = −39.03 kJ/mol), placing it at 16.23 kJ/mol below the **moc-**Zn_4_ **Im**_8_H**Im** phase. The 0.5cHANE@**neb1**-Zn**Im**_2_ (cHANE@**neb1**) phase is slightly less effectively stabilized by cyclohexane (Δ*E* = −35.15 kJ/mol), yet remains at 10.39 kJ/mol below **moc-**Zn_4_ **Im**_8_H**Im**. Since the two **neb** guest@Zn**Im**_2_ phases are both densely packed (solvent accessible void fraction is 0.0% for both), the extra stabilization in the **neb2** phase is likely attributable to weak C–H•••N interactions connecting the pyridine nitrogen to the framework imidazolates (Figure S92). Indeed, if the PYR guest in **neb2-**Zn**Im**_2_ is replaced by benzene (HPh) *in silico*, the stabilization is lowered by 4.28 kJ/mol (Δ*E*_empty_ = −34.75 kJ/mol, Δ*E*_moc_ = −11.95 kJ/mol), and is very close to the stabilization of **neb1-**Zn**Im**_2_ by the cHANE template. It is possible that the described C–H•••N interactions could act as a pathway to true templation and ligand preorganization, providing a potential explanation why the PYR liquid additive is uniquely suited to producing the **neb2-**Zn**Im**_2_ phase. Similarly, if cHANE in **neb1** is replaced by HPh, the degree of stabilization is lessened (Δ*E*_empty_ = −32.30 kJ/mol, Δ*E*_moc_ = −7.53 kJ/mol), demonstrating the importance of molecular shape for the stabilization of **neb1-**Zn**Im**_2_. Namely, bulkier, nonplanar aliphatic 6-membered rings in the chair conformation fit the wider **neb1** cage much better than flat aromatic rings (Figure 6).

**Figure 6:**
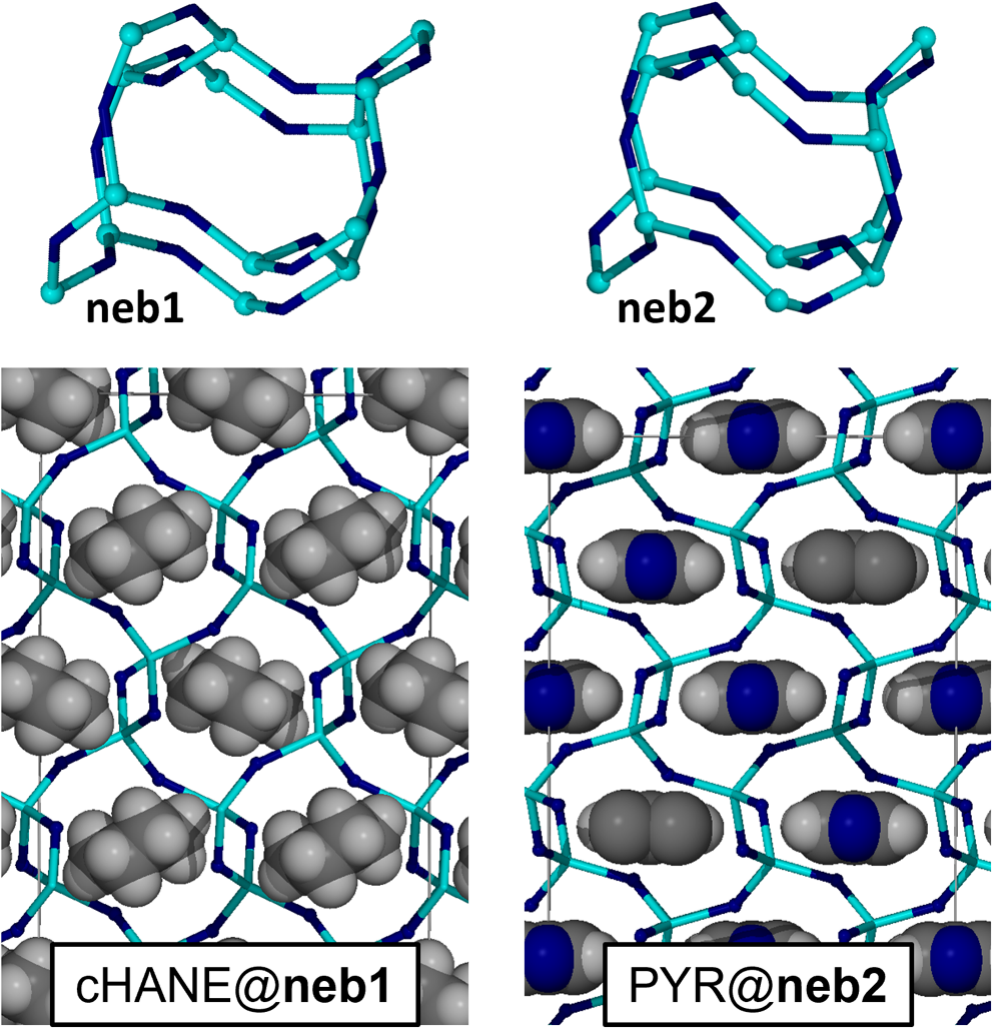
Comparison of the crystal structures of the DFT-optimized cHANE@**neb1** and PYR@**neb2** phases, and their **neb** cages. The frameworks and cages are shown in the node-and-linker representations (light blue = Zn, dark blue = **Im** ^**–**^ centroid), while the template molecules are shown in spacefill and CPK color.

For **cag**-Zn**Im**_2_, we tested five different template molecules, chloroform (CHCl_3_), DMF, DEF, tetrahydrofuran (THF) and cHANE. All five additives provide solvated 0.5guest@**cag**-Zn**Im**_2_ (guest@**cag**) phases calculated to be more thermodynamically stable than **moc-**Zn_4_ **Im**_8_H**Im**, with relative energies in the following order: DEF@**cag**, −24.13 kJ/mol < cHANE@**cag**, −12.94 kJ/mol < DMF@**cag**, −11.33 kJ/mol < THF@**cag**, −10.17 kJ/mol< CHCl_3_@**cag**, −5.76 kJ/mol. Unexpectedly, the most stable of the guest@**cag-**Zn**Im**_2_ phases according to our calculations – DEF@**cag** – is the only one we do not observe experimentally. The DEF additive instead provides the aforementioned amorphous phase that transforms into the DEF@**nog** phase, which is significantly less stable by our calculations than the DEF@**cag** phase (Δ*E*_cag‑nog_ = 26.72 kJ/mol). However, insertion of more DEF molecules into the DEF@**nog** phase may drastically change the relative thermodynamics of these phases and shed more light on the current discrepancies between calculation and experiment.

Another interesting case is that of the cHANE@**cag** phase. As previously mentioned, shorter milling of ZnO and H**Im** with cHANE results in the cHANE@**cag** phase, while longer milling provides the cHANE@**neb1** phase (Figure 7a). Ostwald’s rule of stages, which is considered generally applicable in mechanochemistry,^56,74^ would imply that cHANE@**cag** should be the thermodynamically less stable phase. Our calculations, however, suggest the opposite: cHANE@**cag** is calculated to be more stable than cHANE@**neb1** by Δ*E*_cag‑neb_ = 2.55 kJ/mol; a small but non-negligible amount. Comparison of the optimized crystal structures shows that cHANE@**neb1** is densely packed, but cHANE@**cag** still has a small amount of accessible space (4.1% void space), enough to ensure some mobility of the cHANE molecules in an otherwise inaccessible 0D cavity (Figure 7b). We hypothesize that in the highly dynamic ball milling environment, where particles are constantly being comminuted and new surfaces are opening, cHANE molecules have the opportunity to escape cHANE@**cag** and nucleate cHANE@**neb1**. cHANE molecules appear to then be kinetically locked inside cHANE@**neb1**, and the material is stabilized enough to withstand further milling. Kinetic and entropic effects such as these are impossible to assess via periodic DFT calculations, again emphasizing the need for dynamic modeling.

**Figure 7:**
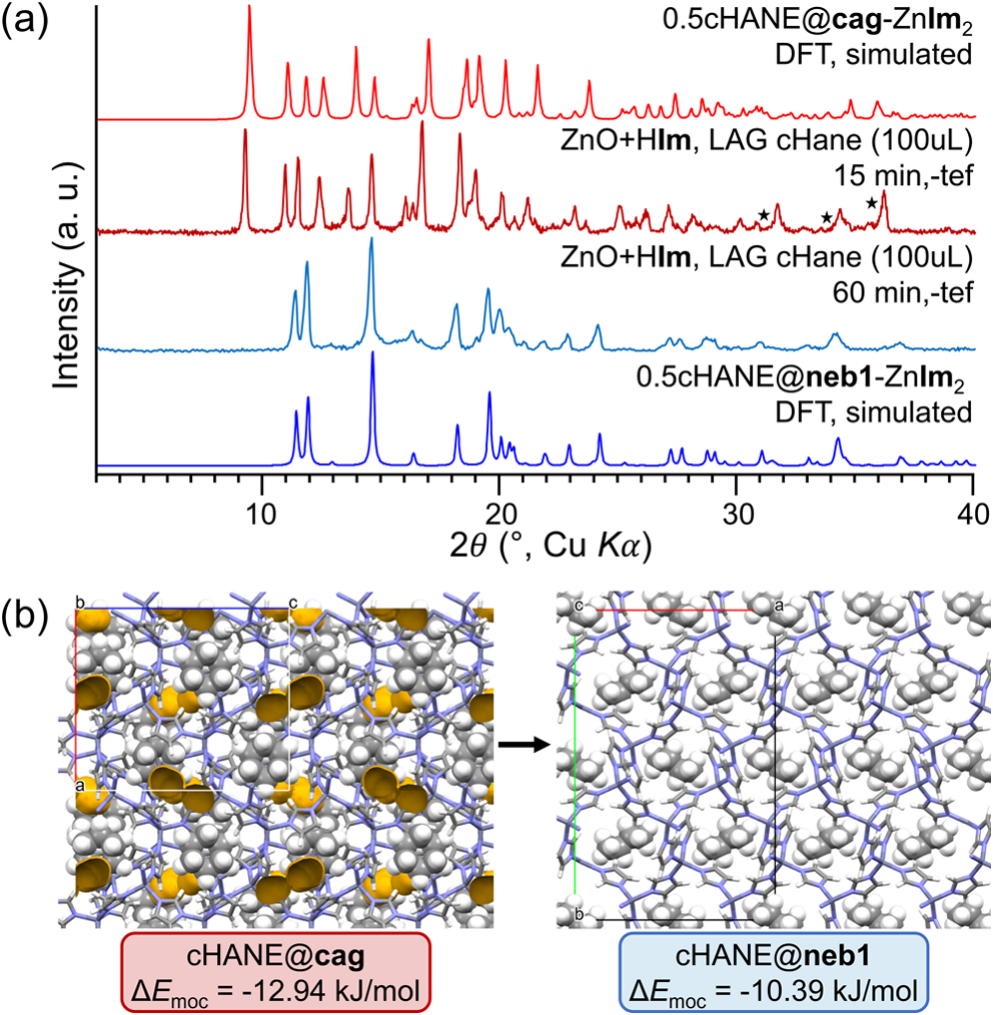
(a) Comparison of experimental PXRD patterns of products of milling ZnO and H**Im** with cyclohexane (cHANE) for 15 and 60 min with simulated PXRD patterns of the DFT-optimized cHANE@**cag** and cHANE@**neb1** phases. Black stars denote peaks of leftover ZnO reagent. (b) Comparison of the crystal structures of the DFT-optimized cHANE@**cag** and cHANE@**neb1** phases, and their relative energies compared to **moc-**Zn_4_ **Im**_8_H**Im**. Voids in the crystal structures are shown in yellow contour.

Similar to cHANE@**cag**, the structure of THF@**cag** contains a small amount of void space (1.8% void space), which is reflected in the experimental behavior. Namely, despite the stabilization of THF@**cag** compared to the empty framework (Δ*E*_empty_ = −32.12 kJ/mol), longer milling times or longer standing in air at room temperature are observed to lead to the collapse of the framework into the denser **coi-**Zn**Im**_2_ phase (Figure S50). Presumably, the voids facilitate the escape of THF molecules from the framework, allowing it to collapse into **coi**-Zn**Im**_2_. Conversely, CHCl_3_@**cag** phase is found to persist upon 60 min of ball-milling, and appears to be the sole thermodynamic product of mechanochemical synthesis, despite the presence of 1.5% void space in the structure. Such behavior could potentially be attributed to a slightly higher vapor pressure of THF compared to CHCl_3_, but could also be due to other factors, such as particle size, surface energy, etc. For example, it is well-known that stability of different polymorphs can vary widely depending on their particle size^120^ which in turn varies depending on the liquid additive or milling conditions.

The third most stable **cag-**Zn**Im**_2_ solvate studied herein is 0.5DMF@**cag**-Zn**Im**_2_ (DMF@**cag**, Δ*E*_moc‑cag_ = −11.33 kJ/mol). Other than DMF@**cag**, we optimized DMF as the guest in two other frameworks, namely 0.2DMF@**nog**-Zn**Im**_2_ (DMF@**nog**) and 0.5DMF@**crb3**-Zn**Im**_2_ (DMF@**crb3**). Of the three solvates, DMF@**cag** has the lowest energy (Δ*E*_moc‑nog_ = +9.38 kJ/mol for DMF@**nog** and Δ*E*_moc‑crb3_ = −8.05 kJ/mol for DMF@**crb3**), and it is the phase experimentally obtained during LAG screening (Figure 8). This agreement between the calculations and experiment provides additional validation of the chosen computational method.

**Figure 8:**
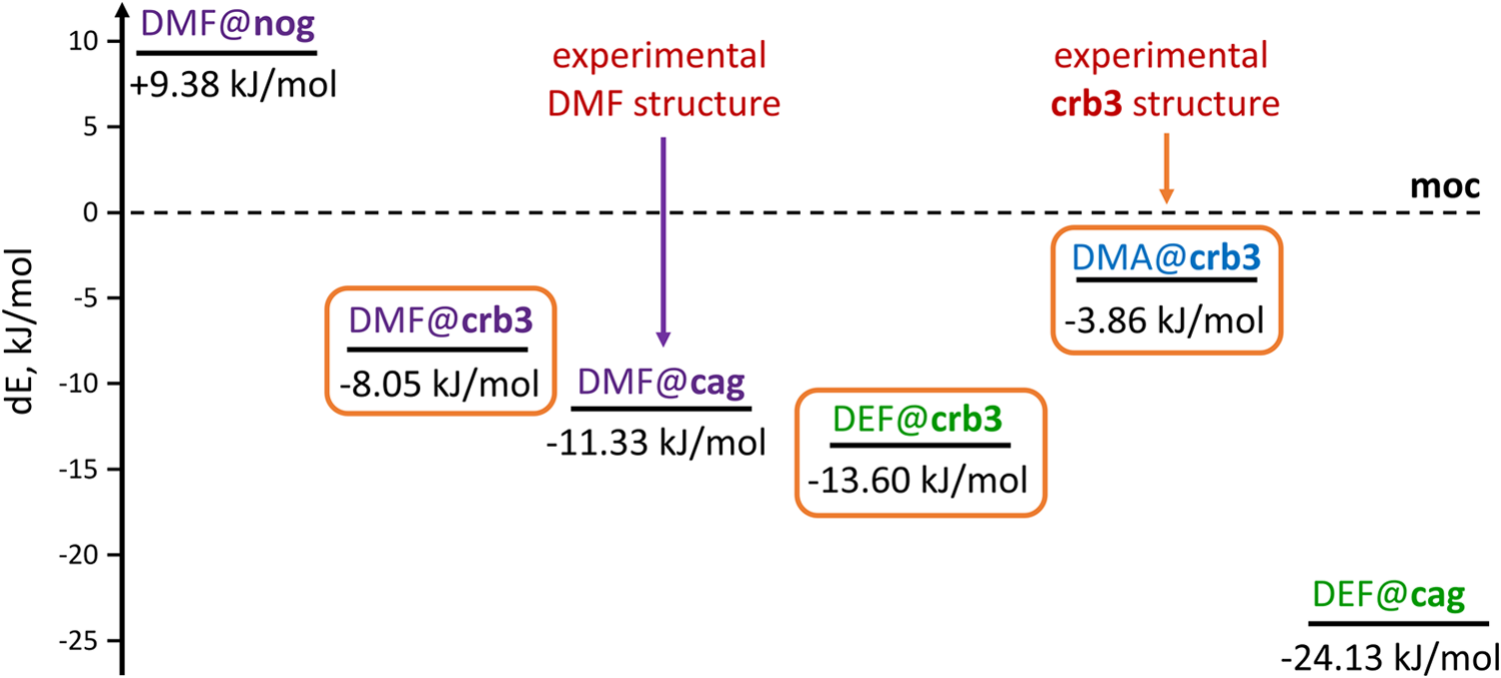
Comparison of energies for different DMF, DEF and **crb3** phases. Materials containing DMF, DEF and DMA are marked in purple, green and blue, respectively, while the three **crb3** phases are circled in orange.

Other than DMF@**crb3**, we also modeled other guest@c**rb3** phases, namely the 0.5DMA@**crb3**-Zn**Im**_2_ (DMA@**crb3**) and the 0.5DEF@**crb3**-Zn**Im**_2_ (DEF@**crb3**) phases. The lowest in energy appeared to be the DEF@**crb3** phase (Δ*E*_DEF_ = −13.60 kJ/mol). However, the DEF@**cag** phase was even lower in energy, and as mentioned before, it is difficult to predict what the energy of the DEF@**nog** phase would be when fully occupied. Logically, DEF ought not yield **crb3**-Zn**Im**_2_, when at least one lower energy phase is available as product. The next lowest in energy would be the DMF@**crb3** phase, but again, there exists a lower energy phase encapsulating DMF, DMF@**cag**. The remaining guest@c**rb3** phase, DMA@**crb3**, while highest in energy of the three **crb3** phases we calculated, still achieves significant stabilization compared to the empty **crb3**-Zn**Im**_2_ (Δ*E*_empty_= −24.54 kJ/mol) and falls below **moc-**Zn_4_ **Im**_8_H**Im** (Δ*E*_moc_ = −3.86 kJ/mol). It is therefore unsurprising that the addition of DMA in LAG screening results in the **crb3** phase (Figure 8).

## 3 Conclusions

Overall, we have demonstrated a novel, fast, highly effective method to screen for ZIF topologies by using small-molecule liquid additives as structure-directing agents in mechanochemical synthesis. We tested 45 different potential additives and prepared 8 different Zn**Im**_2_ topologies in 13 different crystalline forms, including two new forms of **crb**-Zn**Im**_2_, as well as the **moc**-Zn_4_ **Im**_8_H**Im** material and amorphous phases. The results show that strategies and putative templates used in solvothermal ZIF syntheses can be loosely translated to mechanochemical liquid-assisted screening protocols, as well as that using mechanochemical methods significantly expands the scope of the structure-directing agents that can be explored, since reagent solubility is no longer a constraint.

Periodic DFT calculations performed on guest-free and solvated structures of the obtained zinc imidazolate solid forms strongly suggest that these systems are ultimately governed by thermodynamics, with structure stabilization achieved by effective pore solvation. In addition, we have shown that by comparing the energetics of different frameworks containing the same additive, it was possible to anticipate which ones will be experimentally favored. Consequently, this synergistic experimental and theoretical work illustrates the potential predictive power of combining periodic DFT calculations with mechanochemical screening for MOF syntheses. In the future, it ought to be possible to conduct additive screenings *in silico*, saving precious experimental resources, as well as conduct the improved experimental discovery of new phases through real-time monitoring techniques based on synchrotron X-ray powder diffraction, or even Raman spectroscopy, including terahertz-frequency Raman spectroscopy (THz-Raman).^74,75,97,121^ However, the success of DFT modeling depends on the starting model used, so combining DFT with dynamical methods to obtain better starting models and probe the effects of different structure-directing agents might offer many benefits for the future. In addition, we are currently unable to model the kinetics of these syntheses, which is sorely needed to successfully make predictions about the outcomes of mechanochemical templation of ZIFs. However, we believe this work presents significant step toward that end goal.

## 4 Materials and Methods

Full details of all methods and experimental procedures can be found in the Supporting Information (SI). All chemicals were purchased (SI-1.1) and used without further purification.

### 4.1 Powder X-ray Diffraction (PXRD) Patterns

were collected using either a Bruker APEX II DUO CCD area-detector diffractometer operating in transmission mode (DUO), a Bruker D2 powder X-ray diffractometer in Bragg–Brentano mode (D2), or a Panalytical Aeris powder X-ray diffractometer in Bragg–Brentano mode (Aeris). The PXRD patterns were collected in the 3–45° 2θ range using Cu–Kα radiation (λ = 1.5418 Å), with the sample mounted on a silicon (Aeris) or plastic plate (D2), or in a Kapton capillary (DUO). Capillary PXRD data for structure solution were collected at room temperature (RT) on a Malvern Panalytical Empyrean diffractometer using Cu–Kα radiation (λ = 1.5418 Å), collecting from 2 to 70° 2θ with a step size of 0.0077° and 80 s exposure time with the sample mounted in a Kapton capillary. High resolution synchrotron PXRD data for structure solution were collected using beamline 11-BM at the Advanced Photon Source (APS), Argonne National Laboratory using an average wavelength of 0.412602 Å, with the sample mounted in a Kapton capillary.

### 4.2 Single Crystals of the CHCl_3_, THF and DEF Solvates of cag-ZnIm_2_

were all prepared by soaking single crystals of 0.5DMF@**cag**-Zn**Im**_2_ (synthesized according to Park et al.^27^) in the corresponding solvent over several weeks, with periodic exchanges of the soaking solvent.

### 4.3 Single Crystal X-ray Diffraction (SCXRD) Data

were collected on a Bruker-AXS APEX II DUO single crystal diffractometer equipped with an Oxford Cryosystems 700 Cryostream, using Mo Kα radiation (0.71073 Ǻ). The crystal structures were solved by direct methods using SHELXS^122^ and all structural refinements were conducted using SHELXL-2014-7.^123^ All hydrogen atoms were placed in calculated positions and were refined using a riding model with coordinates and isotropic displacement parameters depending upon the atom to which they are attached.

### 4.4 Thermogravimetric Analyses (TGA)

were conducted on a Simultaneous Thermal Analyzer (STA) 6000 (PerkinElmer, Inc.) in alumina crucibles, heated at a rate of 7 °C/min from 35 to 700 °C under dynamic atmosphere of oxygen gas with a flow rate of 30 mL/min.

### 4.5 Differential Scanning Calorimetry (DSC)

measurements were conducted on a TA DSC 25 instrument (TA Instruments Inc., New Castle, DE) in a temperature range from 35 to 250 °C in a dynamic nitrogen atmosphere (50 mL/min) using TZero aluminum pans (40 μL). The heating rate was set at 5 °C/min.

### 4.6 Fourier Transform Infrared (FTIR)

spectroscopy measurements were performed on a PerkinElmer FTIR spectrometer Spectrum Two using Spectrum10 software in transmittance mode and FTIR-ATR technique in the range of 400–4000 cm^–1^ using 4 averaged scans with a resolution of 4 cm^–1^.

### 4.7 Nuclear Magnetic Resonance (NMR)

spectra were recorded on a Bruker Avance 600 MHz spectrometer. The temperature was kept constant at 25 °C and chemical shifts are reported in ppm and referenced to residual solvent signals. Samples were prepared by dissolving 0.5–1 mg of sample in a mixture of 0.5 mL *d6*-DMSO and 25 μL DCl.

### 4.8 Gas Adsorption Analyses

were conducted on a Micromeritics ASAP 2020 porosimeter. All samples were analyzed in a 6 mm bulb cell, at 77 K, with N_2_ as the analysis gas. Outgas was performed under vacuum at: (a) room temperature for 0.65PhMe@**crbT**-Zn**Im**_2_, (60h); (b) 150 °C for 0.51AcPhe@**crbA**-Zn**Im**_2_ (12 h); and (c) room temperature for 0.51AcPhe@**crbA**-Zn**Im**_2_ (18 h).

### 4.9 Ball-Milling Experiments

were conducted in a 14 mL Teflon (obtained commercially from Form-Tech Scientific or InSolido Technologies) or stainless steel (obtained commercially from InSolido Technologies) jar with one 7 mm (1.4 g) and one 9 mm (3.5 g) diameter stainless steel ball bearing. In a typical liquid assisted grinding (LAG) experiment, 100 μL (or 200 μL, 300 μL, or an equimolar amount compared to zinc, if so noted) of a given liquid was added into a milling jar containing the ball bearings, zinc oxide (75.0 mg, 0.92 mmol) and imidazole (125.5 mg, 1.84 mmol). The samples were milled at 30 Hz for 15–90 min using a Retsch MM400 ball mill or an InSolido Technologies IST-500 mixer mill. The products were collected by scraping with a spatula and analyzed without washing or further purification. To avoid cross-contamination, the milling balls and jars were cleaned by milling a mixture of sodium hydrogen carbonate and laboratory solid detergent (Sparkleen or Vim) with a few drops of added ethanol or water for 15 min at 30 Hz frequency after every use, and then washed with soap and water, and rinsed with DI water and ethanol.

### 4.10 Structure Solution from PXRD Data

The PXRD patterns of 0.88PhMe@**crbT**-Zn**Im**_2_ and 0.49AcPhe@**crbA**-Zn**Im**_2_ were indexed using DICVOL06 ^124^ and NTREOR^125^ algorithms, as implemented in the program EXPO2014 ^126^ followed by Le Bail pattern decomposition^127^ and space group determination. Direct methods structure solution was then performed in the same program, determining the positions of Zn centers. The imidazolate ligand positions were either found from electron density (for 0.88PhMe@**crbT**-Zn**Im**_2_) or inserted manually (for 0.49AcPhe@**crbA**-Zn**Im**_2_). Pawley refinement, simulated annealing structure solution and Rietveld refinement were performed using TOPAS v7.^128^ Peaks of ZnO impurity (in the **crbT** structure, COD code 1011258 ^129^) were explicitly modeled in all procedures, using the known ZnO structure and unit cell parameters. Positions of guest molecules (toluene and acetophenone) were found through the Simulated Annealing (SA) algorithm, where only the positions, orientations and occupancies of guest molecule fragments were allowed to vary. Both structures were then subjected to Rietveld refinement. Cycles of periodic DFT optimization (with unit cell parameters fixed to their experimental values) and subsequent Rietveld refinement using DFT-optimized rigid bodies were then performed for both materials until a satisfactory final structure was achieved. Full details of the employed procedures can be found in Sections SI-1.5, SI-1.6 and SI-2.3.2.

A preliminary structure for 0.5cHANE@**neb1**-Zn**Im**_2_ was prepared from the isostructural cyclohexanol solvate of Co**Im**_2_ (CSD code EQOCES^29^). Rietveld refinement for 0.5cHANE@**neb1**-Zn**Im**_2_ was then performed using TOPAS v7 ^128^ by refining the position of the Zn atom, while positions and orientations of imidazolate and cyclohexane fragments were refined with rigid body constraints. Peaks of ZnO (COD code 1011258 ^129^) and **moc**-Zn_4_ **Im**_8_H**Im** (CSD code KUMXEW^39^) impurities were explicitly modeled and a mixed-phase refinement performed. The resulting structural model was then subjected to periodic DFT geometry optimization with unit cell parameters fixed at their experimental values. The DFT-optimized structure was then used to define the rigid body for the final refinement cycle. Full details of the employed procedures can be found in Sections SI-1.5, SI-1.6 and SI-2.3.1.

### 4.11 Periodic DFT Calculations

were performed with the plane-wave DFT code CASTEP 19.1 or 20.1.^118^ The input files were prepared from crystal structures solved from SCXRD and PXRD data, or obtained from the CSD. In each crystal structure disorder was resolved into components which were individually optimized and the lowest energy structure was taken into consideration. Prior to geometry optimization, C–H bond lengths were normalized to a value of 1.088 Å in Mercury, in order to speed up the optimization toward the energy minimum geometry.

For the empty ZIF structures, all guests were deleted from the parent framework, and for the guest-filled structures, guests were either taken directly from a solved or published crystal structure, or a preliminary guest structure was generated inside the framework pores using X-Seed^130^ (see SI-2.9.3). CASTEP-compatible .cell files were then generated for empty ZIFs, gas-phase guests and the ZIF-guest complexes using the cif2cell^131^ program. All structures with I-, C- or F-centered lattices were transformed to the corresponding primitive structure with the aim of reducing the cell volume and, thus, the computational cost of the DFT calculation. This transformation preserved all the symmetry operations of the original structure. An optimization of DFT parameters was performed using **moc**-Zn_4_ **Im**_8_H**Im** (CSD code KUMXEW) as the model structure (Section SI-2.9.1). The plane wave basis set was truncated at 800 eV cutoff and ultrasoft on-the-fly generated pseudopotentials were used to attenuate Coulomb potential in the core regions. Electronic calculations were performed with PBE functional^114^ combined with Grimme D3 ^116^ semiempirical dispersion correction. The electronic Brillouin zone was sampled with a 0.06 Å^–1^ k-point spacing. Crystal structures were optimized with respect to unit cell parameters and atom positions, subject to space group symmetry constraints. The geometry convergence criteria were set as follows: maximum energy change: 1 × 10^–10^ eV atom^–1^; maximum atom displacement: 0.001 Å; maximum atomic force: 0.05 eV Å^–1^; maximum value of stress tensor parameters: 0.05 GPa. Example input files can be seen in the SI-2.9.2.

Gas-phase energies of guest molecules were also calculated. by placing each guest molecule in a large cubic cell (Lx = Ly = Lz = 25 Å) and optimizing the geometry. The unit cell dimensions were kept fixed to prevent contraction of the simulation box and aggregation of the molecules located in the periodic images of the simulation cell. The electronic Brillouin zone was sampled with the Γ k-point, and all the other calculation parameters were set the same as for the geometry optimization of the ZIF crystal structures.

## Supplementary Material



## Data Availability

Original raw data related to this publication is available from the Zenodo data repository: https://doi.org/10.5281/zenodo.13602596.

## Notes

Details of synthetic procedures, periodic DFT calculations, crystal structure solution and characterization methods; PXRD analyses of all reactions; sorption, TGA, IR, NMR and DSC data for **crbT** and **crbA** materials; graphs and tables summarizing the computational results, including optimizations of parameters; example input files used for calculations; images of crystal structures; tables summarizing the templates used, the reaction outcomes, and materials properties (PDF)
