# Supplementary material for: Mechanochemical Solid Form Screening of Zeolitic Imidazolate Frameworks Using Structure-Directing Liquid Additives
Source: J Am Chem Soc. 2025 Jul 24;147(31):27413–30. doi: 10.1021/jacs.5c04043 (PMC12333376; doi:10.1021/jacs.5c04043)
Supplement: Supplementary file 1 [file ja5c04043_si_001.pdf]

Supporting Information for

Mechanochemical Solid Form Screening of Zeolitic Imidazolate Frameworks using Structure-Directing Liquid Additives

Ivana Brekalo,[a,b,c]* Katarina Lisac,[a] Joseph R. Ramirez,[b] Petra Pongrac,[a,d] Andreas Puškarić,[e] Srećko Valić,[a,f] Yizhi Xu,[g] Michael Ferguson,[h] Joseph M. Marrett,[h] Mihails Arhangelskis,[g,]* Tomislav Friščić,[c,h,]* K. Travis Holman[b,]*

[a]Division of Physical Chemistry, Ruđer Bošković Institute, Zagreb 10000, Croatia;

[b]Department of Chemistry, Georgetown University, Washington, D.C. 20057, USA;

[c]Department of Chemistry, McGill University, Montréal H3A 0B8, Canada;

[d]Faculty of Chemical Engineering and Technology, University of Zagreb, Zagreb 10000, Croatia;

[e]Division of Materials Chemistry Chemistry, Ruđer Bošković Institute, Zagreb 10000, Croatia;

[f]Faculty of Medicine, University of Rijeka, Rijeka 51000, Croatia;

[g]Faculty of Chemistry, University of Warsaw, Warsaw 02-093, Poland;

[h]School of Chemistry, University of Birmingham, Birmingham B15 2TT, UK

KEYWORDS: Templation, mechanochemistry, Zeolitic Imidazolate Frameworks, periodic DFT, additives

TABLE OF CONTENTS

1. EXPERIMENTAL DETAILS 4
1.1. General Details 4
1.2. Instrumental Details 4
1.3. Mechanochemical syntheses 6
1.4. Single crystal syntheses 7
1.5. Structure solution from PXRD data 8
1.6. Periodic DFT calculations 9
1.7. List of additives used for mechanochemical screening, their acronyms and molecular diagrams 10
2. RESULTS 13
2.1. Powder X-Ray diffractograms for each liquid screening reaction 13
2.1.1. Untemplated control – neat grinding without additives 13
2.1.2. Acetone (AcMe) 14
2.1.3. Acetophenone (AcPhe) 14
2.1.4. 4-Acetylpyridine (4AcPyr) 15
2.1.5. Butyrolactone (BLAC) 16
2.1.6. Cyclohexane (cHANE) 17
2.1.7. Chloroform ($CHCl_3$) 17
2.1.8. Cyclohexene (cHENE) 18
2.1.9. Cyclohexanone (cHONE) 18
2.1.10. ε-Caprolactone (CLAC) 19
2.1.11. N,N-dibutylformamide (DBF) 19
2.1.12. N,N-diethylformamide (DEF) 20
2.1.13. N,N-dipropylformamide (DPF) 20
2.1.14. 1,4-dioxane (DIOX) 21
2.1.15. N,N-dimethylacetamide (DMA) 21
2.1.16. N,N-dimethylformamide (DMF) 22
2.1.17. Dimethylsulfoxide (DMSO) 22
2.1.18. Triethylamine (Et_3N) 23
2.1.19. Ethylene glycol (EtGly) 23
2.1.20. Ethyl acetate (EtOAc) 24
2.1.21. Ethanol (EtOH) 24
2.1.22. Furan (FUR) 25
2.1.23. Water (H_2O) 25
2.1.24. Hexamethylphosphoramide (HMPA) 26
2.1.25. Acetonitrile (MeCN) 27
2.1.26. Nitromethane ($MeNO_2$) 27
2.1.27. Methanol (MeOH) 28
2.1.28. 4-methylpyridine (4-MePyr) 28

2.1.29. Morpholine (MORPH) ... 29
2.1.30. Butan-1-ol (nBuOH) ... 29
2.1.31. N-methylcaprolactam (NMC) ... 30
2.1.32. N-Methyl-2-pyrrolidone (NMP) ... 31
2.1.33. N-methyl-2-piperidone (NMPd) ... 32
2.1.34. N-Methylpyrrolidine (NMPl) ... 34
2.1.35. N-methylpiperidine (NMPP) ... 35
2.1.36. Propan-1-ol (nPrOH) ... 36
2.1.37. Oxetane (OXT) ... 36
2.1.38. Toluene (PhMe) ... 37
2.1.39. Anisole (PhOMe) ... 40
2.1.40. Piperidine (PP) ... 40
2.1.41. Piperazine (PPZ) ... 41
2.1.42. Pyridine (PYR) ... 41
2.1.43. Tetrahydrofuran (THF) ... 43
2.1.44. Tetrahydropyran (THP) ... 43
2.1.45. Thiophene (TPH) ... 44
2.1.46. Valerolactone (VLAC) ... 44
2.1.47. Summary of PXRD experimental results and previous literature ... 45
2.2. Crystal structures solved from Single Crystal X-Ray Diffraction (SCXRD) ... 52
2.3. Crystal structures solved from Powder X-Ray Diffraction (PXRD) ... 55
2.3.1. Crystal structure of 0.5cHANE@**neb1**-Zn**Im**$_2$ ... 55
2.3.2. Crystal structures of 0.88PhMe@**crbT**-Zn**Im**$_2$ and 0.49AcPhe@**crbA**-Zn**Im**$_2$ ... 56
2.4. Thermogravimetric studies ... 63
2.5. NMR results ... 64
2.6. IR results ... 66
2.7. DSC results ... 67
2.8. N_2 adsorption results ... 68
2.9. DFT results ... 70
2.9.1. DFT parameter optimization ... 70
2.9.2. Periodic DFT optimization of experimentally obtained zinc imidazolate forms ... 73
2.9.3. Periodic DFT optimization of selected guest-filled zinc imidazolate forms ... 80
2.9.4. Comparison of DFT and experimental structures for different topologies of Zn**Im**$_2$ ... 82
2.9.5. Images of selected geometry optimized crystal structures ... 91
2.10. Quantitative Rietveld refinement of nano-ZnO experiments ... 92
3. REFERENCES ... 92

1. EXPERIMENTAL DETAILS

1.1. General Details

Zinc oxide (ZnO) was purchased from Alfa Aesar and Lach-Ner, imidazole (H**Im**) was purchased from Alfa Aesar and Thermo Scientific. N,N-dimethylformamide (DMF), butyrolactone (BLAC), chloroform ($CHCl_3$), acetonitrile (MeCN), methanol (MeOH), Butan-1-ol (nBuOH) and tetrahydrofuran (THF) were purchased from Lach-Ner. Ethanol (EtOH) was purchased from Honeywell. Acetone (AcMe) was purchased from Kemika. Toluene (PhMe) and dimethylsulfoxide (DMSO) were purchased from Gram-Mol. Ethyl acetate (EtOAc) and N-Methyl-2-pyrrolidone (NMP) were purchased from VWR chemicals. Anisole (methoxybenzene, PhOMe) was purchased from Fluka. 4-acetylpyridine was purchased from Acros Organics. Nanoparticulate zinc oxide (NanoTek, 40-100 nm) was purchased from Alfa Aesar. N,N-dipropylformamide was purchased from Angene. Zinc acetate dihydrate was purchased from Merck. DCl was purchased from Cambridge Isotope Laboratories. *d6*-DMSO was purchased from Eurisotop. Acetophenone (AcPhe), hexamethylphosphoramide (HMPA), 1,4-dioxane (DIOX), cyclohexane (cHANE), cyclohexene (cHENE), cyclohexanone (cHONE), caprolactone (CLAC), N,N-dibutylformamide (DBF), N,N-dimethylacetamide (DMA), N,N-diethylformamide (DEF), triethylamine (Et_3N), furan (FUR), benzene (HPh), morpholine (MORPH), N-methylcaprolactam (NMC), N-methyl-2-piperidone (NMPd), N-Methylpyrrolidine (NMPl), N-methylpiperidine (NMPP), Propan-1-ol (nPrOH), Oxetane (OXT), Piperidine (PP), Piperazine (PPZ), Pyridine (PYR), tetrahydropyran (THP), Thiophene (TPH) and valerolactone (VLC) were purchased from Sigma-Aldrich.
All chemicals were used without further purification.

1.2. Instrumental Details

Powder X-ray diffraction (PXRD) patterns were collected using either a Bruker APEX II DUO CCD area-detector diffractometer operating in transmission mode (DUO), a Bruker D2 powder X-ray diffractometer in Bragg-Brentano mode (D2), or a Panalytical Aeris powder X-ray diffractometer in Bragg-Brentano mode (Aeris).
The *DUO diffractometer* was equipped with a CCD detector using graphite monochromated Cu-$K\alpha$ radiation (λ = 1.54060 Å) from an Incoatec IμS source. DUO samples were mounted in a 0.5 mm capillary tube (Kapton® ®) and were irradiated for 90 seconds per scan while rotating about the φ axis with three scans in the orientations of 2θ = -12, -24, and -36°, and ω = 174, 168, and 162°, respectively. The diffraction data were integrated using Apex3 v.2016.5-0 software (3-40° 2θ, with a 0.02° step size).
The *D2 diffractometer* was equipped with a Cu-Kα (λ=1.5418 Å) source and Lynxeye detector set at a discriminant range of 0.110 V to 0.250 V. The patterns were collected in the range of 3° to 40°, using a PMMA (poly(methylmethacrylate)) plate with a sample well, made in-house.

The *Aeris diffractometer* PXRD patterns were collected in the 3-45° 2θ range using a 0.003° step size with an overall scan time of 6 minutes, and Bragg-Brentano geometry, using Cu-Kα radiation (λ=1.5418 Å, 40 kV, 7.5 mA) with the sample mounted on a zero-background silicon plate.
Analysis of PXRD patterns was conducted using Panalytical X'Pert Highscore Plus[1] software, and raw data was converted into a suitable format using the PowDLL[2] program. Experimental patterns were compared to simulated patterns calculated from single crystal structures using Mercury[3] crystal structure viewing software. Crystallographic Information Files containing published crystal structures were obtained from the Cambridge Structural Database (CSD)[4,5] or the Crystallography Open Database (COD)[6].

Capillary PXRD data for structure solution (0.65PhMe@**crbT**-Zn**Im**$_2$ and 0.51AcPhe@**crbA**-Zn**Im**$_2$)were collected at room temperature (RT) on a Malvern Panalytical Empyrean diffractometer, equipped with a long fine focus X-ray tube with copper anode (45 kV, 40 mA, λ=1.5418 Å), a W/Si multilayer elliptical focusing mirror (0.84 angle) and a PIXcel3d detector in scanning line mode. The sample was mounted in a 0.5 mm Kapton® capillary, and the diffractogram collected from 2 to 70 °2θ with a step size of 0.0077 °2θ, and 80 s exposure time.

High resolution synchrotron PXRD data for structure solution (0.5cHANE@**neb1**-Zn**Im**$_2$)were collected using beamline 11-BM at the Advanced Photon Source (APS), Argonne National Laboratory using an average wavelength of 0.412602 Å. The diffractogram was collected over a 0.5-50 °2θ range, with data points collected every 0.001° 2θ and scan speed of 0.01°/s. The sample was mounted in a Kapton® capillary. Structure solution and refinement details can be found in section 2.6.3.

CCDC depository numbers 2381143, 2381139, and 2381142 contain the powder X-ray structure data for 0.5cHANE@**neb1**-Zn**Im**$_2$, 0.65PhMe@**crbT**-Zn**Im**$_2$ and 0.51AcPhe@**crbA**-Zn**Im**$_2$, respectively. The data can be obtained free of charge via www.ccdc.cam.ac.uk/data_request/cif, by e-mailing data_request@ccdc.cam.ac.uk, or by contacting The Cambridge Crystallographic Data Centre, 12 Union Road, Cambridge CB2 1EZ, UK; Fax: + 44-1223-336033. CCDC.

Single crystal X-ray diffraction (SCXRD) data were collected on a Bruker-AXS APEX II DUO single crystal diffractometer equipped with an Oxford Cryosystems 700 Cryostream, using Mo *K*α radiation (0.71073 Å). The crystal structures were solved by direct methods using SHELXS[7], and all structural refinements were conducted using SHELXL-2014-7[8]. All hydrogen atoms were placed in calculated positions and were refined using a riding model with coordinates and isotropic displacement parameters depending upon the atom to which they are attached.

The program X-Seed[9] was used as a graphical interface for the SHELX software suite and for the generation of the figures. CCDC depository numbers 2381140, 2381141 and 2423969 contain the single-crystal X-ray structure data for 0.5$CHCl_3$@**cag**-Zn**Im**$_2$, 0.5THF@**cag**-Zn**Im**$_2$ and 0.5DEF@**cag**-Zn**Im**$_2$, respectively. The data can be obtained free of charge via www.ccdc.cam.ac.uk/data_request/cif,

by e-mailing data_request@ccdc.cam.ac.uk, or by contacting The Cambridge Crystallographic Data Centre, 12 Union Road, Cambridge CB2 1EZ, UK; Fax: + 44-1223-336033. CCDC.

Thermogravimetric analyses (TGA) were conducted on a Simultaneous Thermal Analyzer (STA) 6000 (PerkinElmer, Inc.) in alumina crucibles at heated at a rate of 7 °C/min from 35 °C to 700 °C under dynamic atmosphere of oxygen gas, with a flow rate of 30 mL/min. TGA curves were analyzed using TA Universal Analysis 2000 v. 3.9A software, and Microsoft Excel (for figure creation).

Differential scanning calorimetry (DSC) measurements were conducted on a TA DSC 25 instrument (TA Instruments Inc., New Castle, DE, USA) in a temperature range from 35 to 250 °C in a dynamic nitrogen atmosphere (50 mL/min) using TZero aluminium pans (40 μL). The heating rate was set at 5 °C/min.

Fourier transform infrared (FTIR) spectroscopy measurements were performed on a PerkinElmer FTIR spectrometer Spectrum Two using Spectrum10 software (PerkinElmer, Inc.) in transmittance mode and FTIR-ATR technique in the range of 400-4000 cm^{-1} using 4 averaged scans with a resolution of 4cm^{-1}.

Nuclear Magnetic Resonance (NMR) spectra were recorded on a Bruker Avance 600 MHz spectrometer. The temperature was kept constant at 25 °C and chemical shifts are reported in ppm and referenced to residual solvent signals. Samples were prepared by dissolving 1-2 mg of sample in a mixture of 0.5 mL *d6*-DMSO and 50 μL DCl.

Gas adsorption analyses were conducted on a Micromeritics ASAP 2020 porosimeter. All samples were analyzed in a 6 mm bulb cell, at 77 K, with N_2 as the analysis gas. Outgas was performed under vacuum at: a) room temperature for 0.65PhMe@**crbT**-Zn**Im**$_2$, (60h); b) 150 °C for **crbA**-Zn**Im**$_2$ (12 h); and c) room temperature for **crbA**-Zn**Im**$_2$ (18 h). For a full isotherm 40 points were collected in adsorption mode, with P/P_0 ranging from 0.00001 to 0.9975, and 20 points were collected in desorption mode, with P/P_0 ranging from 0.9975 to 0.05. Data were analyzed using the AS1 v. 1.55B software package and Microsoft Excel (for figure preparation). BET surface areas were calculated using adsorption points at P/P_0 = 0.005, 0.008, 0.01, 0.03, and 0.05. Total pore volume and average pore size were determined from adsorption at P/P_0 = 0.955. Pore size distribution was calculated using the Saito-Foley (SF) method using all adsorption points below P/P_0 = 0.02.

Milling reactions were conducted in a 14 mL Teflon™ (Form-Tech Scientific or InSolido Technologies) or stainless steel (hereafter referred to as steel, InSolido Technologies) jar with one 7 mm (1.4 g) diameter and one 9 mm (3.5 g) diameter stainless steel ball bearing. Exceptionally, reactions involving N,N-dibutylformamide used two 10 mm (3.2 g) zirconia ball bearings.

1.3. Mechanochemical syntheses

Milling syntheses In the neat grinding (NG) reactions zinc oxide (75.0 mg, 0.92 mmol) and imidazole (125.5 mg, 1.84 mmol) were added into a milling jar containing two ball bearings. In the liquid assisted

grinding (LAG) reactions, 100 μL (or 200 μL, 300 μL, or an equimolar amount compared to zinc, if so noted) of a given liquid was also added into the milling jar. The samples were milled at 30 Hz for up to 90 min (milling time noted on an individual basis) using a Retsch MM400 ball mill or an InSolido Technologies IST-500 mixer mill. The products were collected by scraping with a spatula and analyzed without washing or further purification. To avoid cross-contamination, the milling balls and jars were cleaned by milling a mixture of sodium hydrogencarbonate and laboratory solid detergent (Sparkleen or Vim) with a few drops of added ethanol or water for 15 min at 30 Hz frequency after every use, and then washed with soap and water and rinsed with DI water and ethanol.

1.4. Single crystal syntheses

0.5DMF@cag-ZnIm$_2$ The synthesis was performed similar to that reported by Park *et al.*[10] In a 20 mL vial, $Zn(NO_3)_2$•$4H_2O$ (9.8 mg, 0.038 mmol) and imidazole (31 mg, 0.46 mmol) were dissolved in 3 mL of *N*,*N*-dimethylformamide (DMF). The resulting mixture was filtered through a cotton plug into a clean 20 mL vial. The vial was placed in an oven at 120 °C for three days to yield colorless crystals. The crystals were separated from the mother liquor *via* vacuum filtration. The as-synthesized crystals were then washed in DMF and stored in fresh DMF for later use.

0.5CHCl$_3$@cag-ZnIm$_2$ A single crystal of 0.5DMF@**cag**-Zn**Im**$_2$ was placed in a 7 mL screwcap vial containing chloroform. The solvent-exchange process was allowed to take place for 7 days, and the crystal was then analyzed *via* SCXRD.

0.5THF@cag-ZnIm$_2$ and **0.5DEF@cag-ZnIm$_2$** Suitable as-synthesized single crystals of 0.5DMF@**cag**-Zn**Im**$_2$ were placed in 7 mL screwcap vials containing 3-5 mL of tetrahydrofuran (THF) and N,N-diethylformamide, respectively. The vials were then capped and set aside at room temperature for three months with periodic decanting of the vial and replacing with fresh guest solvent. The crystals were then analyzed *via* SCXRD.

Crystallization attempts of crbT and crbA

0.2 M stock solutions of H**Im** and $Zn(NO_3)_2$ were prepared in *N*,*N*-diethylformamide (DEF).

a) RT syntheses

0.5 mL of H**Im** solution, 0.25 mL of $Zn(NO_3)_2$ solution, 0.25 mL of triethylamine and 1 mL of acetophenone (AcPhe) or toluene (PhMe) were added in a vial, sonicated, and left to stand at room temperature. No single crystals were formed.

b) 90 °C syntheses

Reactions were performed at three sets of reagent concentrations:

- 1 mL H**Im** solution, 0.5 mL $Zn(NO_3)_2$ solution, 1 mL AcPhe or PhMe
- 0.5 mL H**Im** solution, 0.25 mL $Zn(NO_3)_2$ solution, 1.5 mL DEF, 1 mL AcPhe or PhMe
- 0.75 mL H**Im** solution, 0.25 mL $Zn(NO_3)_2$ solution, 1.5 mL DEF, 1 mL AcPhe or PhMe

All vials were sonicated, capped, and put in a preheated oven at 90 °C for 4 days (96 hours), when the oven was turned off and the vials left to cool slowly. No crystals were formed in any of the reactions.

1.5. Structure solution from PXRD data

The powder patterns of 0.65PhMe@**crbT**-Zn**Im**$_2$ and 0.51AcPhe@**crbA**-Zn**Im**$_2$ were indexed using DICVOL06[11] and NTREOR[12] algorithms, as implemented in the program EXPO2014[13], followed by Le Bail pattern decomposition[14] and space group determination. Direct methods structure solution was then performed in the same program using a presumed composition of Zn**Im**$_2$, with the appropriate number of toluene and acetophenone guest molecules presumed (based on TGA analyses, section SI-2.4). The positions of Zn centers were thus determined, and imidazolate ligand positions were either found from electron density (for 0.65PhMe@**crbT**-Zn**Im**$_2$) or inserted manually (for 0.51AcPhe@**crbA-ZnIm**$_2$). The positions of guest molecules could not be determined during this process.

Pawley refinement, simulated annealing structure solution, and Rietveld refinement for 0.65PhMe@**crbT**-Zn**Im**$_2$ and 0.51AcPhe@**crbA**-Zn**Im**$_2$ were performed using TOPAS v7[15]. First, Pawley refinement was used to refine unit cell parameters, Lorentzian polarization and background Chebyshev polynomial terms, zero error, Simple Axial parameters and peak shape parameters (pseudo-Voigt function). Peaks of ZnO impurity (in the crbT structure, COD code 1011258[16]) were explicitly modeled in all procedures, using the known ZnO structure and unit cell parameters. Positions of guest molecules (toluene and acetophenone) were then found through the Simulated Annealing (SA) algorithm. During the SA procedure, the unit cell parameters, host framework atom positions, pseudo-Voigt peak shapes and Chebyshev background polynomials were kept fixed, only the positions, orientations and occupancies of guest molecule fragments were allowed to vary. The occupancies were constrained to remain within the limits of 0 to 1. Both structures were then subjected to Rietveld refinement, where positions of all Zn atoms, as well as positions and orientations of framework imidazolate and guest molecules were refined. Cycles of periodic density-functional theory (DFT) optimization (with unit cell parameters fixed to their experimental values) and subsequent Rietveld refinement using DFT-optimized rigid bodies were then performed for both materials until a satisfactory final structure was achieved. Full details of the employed procedures can be found in section SI-2.3.

The network topologies of these structures were determined using two different methods: standalone software ToposPro[17] and web-based software TopCryst[18]. Both methods were in agreement and assigned the structures as forms of the **crb** (BCT) topology.

A preliminary structure for 0.5cHANE@**neb1**-Zn**Im**$_2$ was prepared from the isostructural cyclohexanol solvate of Co**Im**$_2$ (CSD code EQOCES[19]). Rietveld refinement for 0.5cHANE@**neb1**-Zn**Im**$_2$ was then performed using TOPAS v7[15], first refining the zero point, unit cell parameter, Chebyshev polynomial background function and pseudo-Voigt peak shape function. In the next step, the position of Zn atom was refined, while positions and orientations of imidazolate and cyclohexane fragments were refined with rigid body constraints. Finally, atomic thermal motion was described with a single isotropic Debye-Waller factor. Peaks of ZnO (COD code 1011258[16]) and **moc**-Zn_4Im_8(H**Im**) (CSD code

KUMXEW[20]) impurities were then explicitly modeled and a mixed-phase refinement performed. The resulting structural model was then subjected to periodic DFT geometry optimization with unit cell parameters fixed at their experimental values (for details of the periodic DFT procedure see SI-1.6). The DFT-optimized structure was then used to define the rigid body for the final refinement cycle.

1.6. Periodic DFT calculations

Periodic DFT calculations were performed with the plane-wave DFT code CASTEP 19.1 or 20.1.[21] The input files were prepared from crystal structures solved from SCXRD and PXRD data, or obtained from the CSD. In each crystal structure disorder was resolved into components which were individually optimized and the lowest energy structure was taken into consideration. Prior to geometry optimization, C-H bond lengths were normalized to a value of 1.088 Å in Mercury, to speed up the optimization towards the energy minimum geometry. However, no constraints were applied to hydrogen positions during the geometry optimization calculations.

For the empty ZIF structures, all guests were deleted from the parent framework, and for the guest-filled structures, guests were either taken directly from a solved or published crystal structure, or a preliminary guest structure was generated inside the framework pores using X-Seed. CASTEP-compatible .cell files were then generated for empty ZIFs, gas-phase guests and the ZIF-guest complexes using the cif2cell[22] program. All structures with I-, C- or F-centered lattices were transformed to the corresponding primitive structure with the aim of reducing the cell volume and, thus, the computational cost of the DFT calculation. This transformation preserved all the symmetry operations of the original structure. An optimization of DFT parameters was performed using **moc**-Zn_4**Im**$_8$(H**Im**) (CSD code KUMXEW[20]) as the model structure (section SI-2.5.1). The plane wave basis set was truncated at 800 eV cutoff and ultrasoft on-the-fly generated pseudopotentials were used to attenuate Coulomb potential in the core regions. Electronic calculations were performed with PBE functional,[23] combined with Grimme D3[24] semiempirical dispersion correction. The electronic Brillouin zone was sampled with a 0.06 $Å^{-1}$ k-point spacing.[25] Crystal structures were optimized with respect to unit cell parameters and atom positions, subject to space group symmetry constraints. The geometry convergence criteria were set as follows: maximum energy change: $1x10^{-10}$ eV atom-^{1}; maximum atom displacement: 0.001 Å; maximum atomic force: 0.05 eV $Å^{-1}$; maximum value of stress tensor parameters: 0.05 GPa.

In addition to the optimization of the crystal structures of the ZIF polymorphs, gas-phase energies of guest molecules were also calculated. This was achieved by placing each guest molecule in a cubic simulation cell of side length equal to 25 Å and optimizing the geometry. In this case, unlike for crystal structures, the unit cell dimensions were kept fixed, in order to prevent contraction of the simulation box and aggregation of the molecules located in the periodic images of the simulation cell. The electronic Brillouin zone in this case was sampled only at the Γ k-point, all the other calculation parameters were set the same as for the geometry optimization of the ZIF crystal structures.

1.7. List of additives used for mechanochemical screening, their acronyms and molecular diagrams

Table S1. List of additives used for mechanochemical screening, their acronyms and molecular diagrams

Liquid additive	Acronym	Molecular diagram
Acetone	AcMe	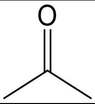
Acetophenone	AcPhe	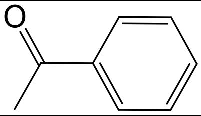
4-Acetylpyridine	4AcPyr	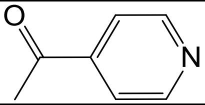
Butyrolactone	BLAC	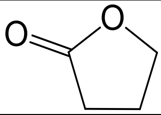
Cyclohexane	cHANE	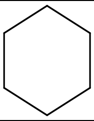
Chloroform	$CHCl_3$	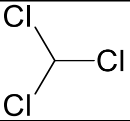
Cyclohexene	cHENE	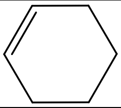
Cyclohexanone	cHONE	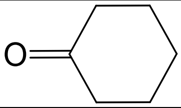
Caprolactone	CLAC	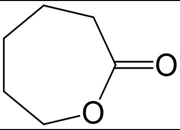
N,*N*-diethylformamide	DEF	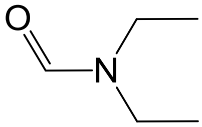
N,N-dipropylformamide	DPF	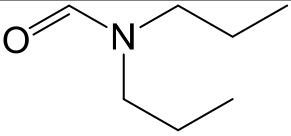
N,*N*-dibutylformamide	DBF	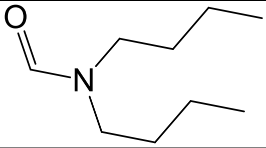
1,4-dioxane	DIOX	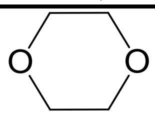

N,*N*-dimethylylacetamide	DMA	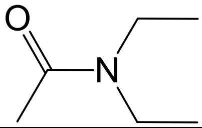
N,*N*-dimethylylformamide	DMF	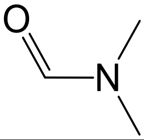
Dimethylsulfoxide	DMSO	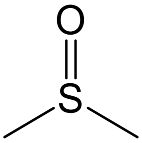
Triethylamine	Et_3N	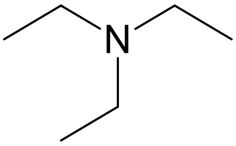
Ethylene glycol	EtGly	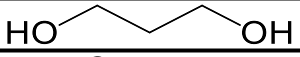
Ethyl acetate	EtOAc	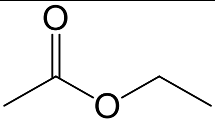
Ethanol	EtOH	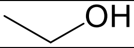
Furan	FUR	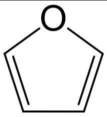
Water	H_2O	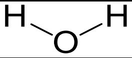
Hexamethylphosphoramide	HMPA	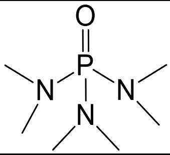
Acetonitrile	MeCN	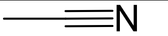
Nitromethane	$MeNO_2$	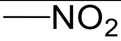
Methanol	MeOH	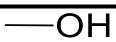
4-methylpyridine	4-MePyr	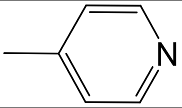
Morpholine	MORPH	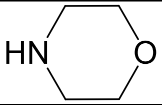
Butan-1-ol	nBuOH	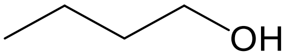
N-methylcaprolactam	NMC	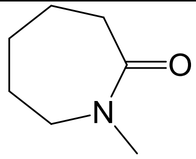

N-methyl-2-pyrrolidone	NMP	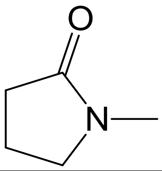
N-methyl-2-piperidone	NMPd	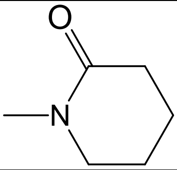
N-methylpyrrolidine	NMPl	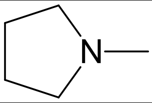
N-methylpiperidine	NMPP	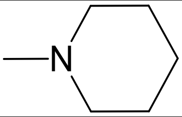
Propan-1-ol	nPrOH	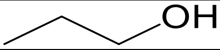
Oxetane	OXT	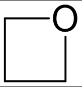
Toluene	PhMe	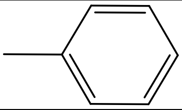
Anisole	PhOMe	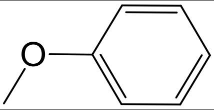
Piperidine	PP	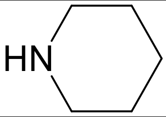
Piperazine	PPZ	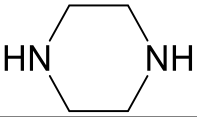
Pyridine	PYR	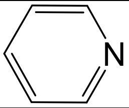
Tetrahydrofuran	THF	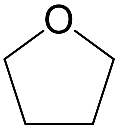
Tetrahydropyran	THP	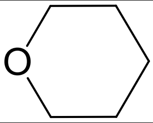
Thiophene	TPH	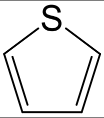
Valerolactone	VLAC	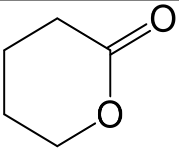

2. RESULTS

2.1. Powder X-Ray diffractograms for each liquid screening reaction

N.B. Black stars will be used to denote peaks of ZnO reagent in all PXRD figures (ZnO diffractogram displaying the original peaks is shown in figure S1). Labels "-st" and "-tef" denote the use of steel and Teflon™ jars, respectively

2.1.1. Untemplated control – neat grinding without additives

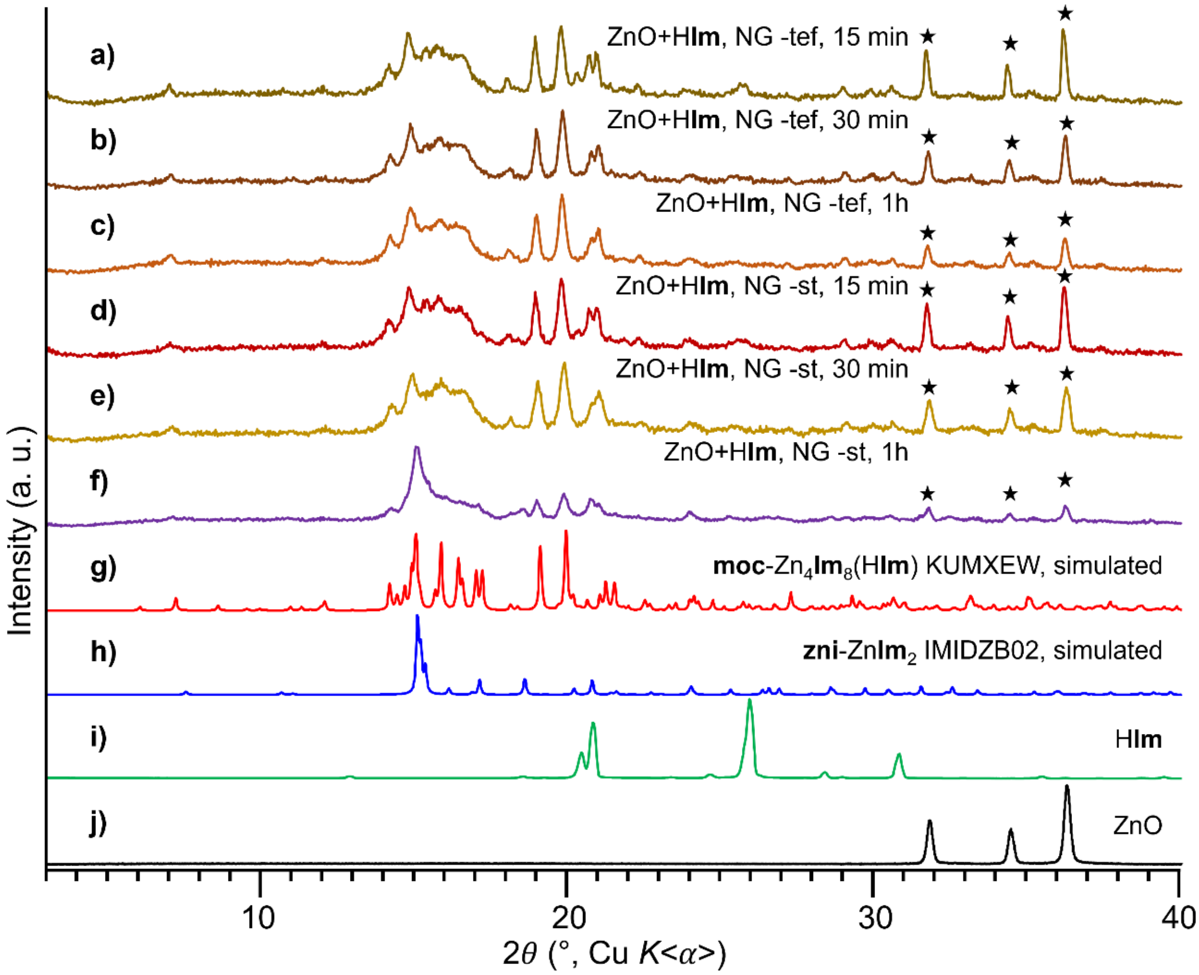

Figure S1. PXRD patterns of the products of neat milling ZnO and H**Im** without additives in a) a Teflon™ jar for 15 min; b) a Teflon™ jar for 30 min; c) a Teflon™ jar for 60 min; d) a steel jar for 15 min; e) a steel jar for 30 min; f) a steel jar for 60 min. Simulated PXRD patterns of g) **moc**-Zn_4**Im**$_8$H**Im** (CSD code KUMXEW) and h) **zni**-Zn**Im**$_2$ IMIDZB02. PXRD patterns of the starting materials i) imidazole (H**Im**) and j) zinc oxide. Black stars denote peaks of leftover ZnO reagent.

2.1.2. Acetone (AcMe)

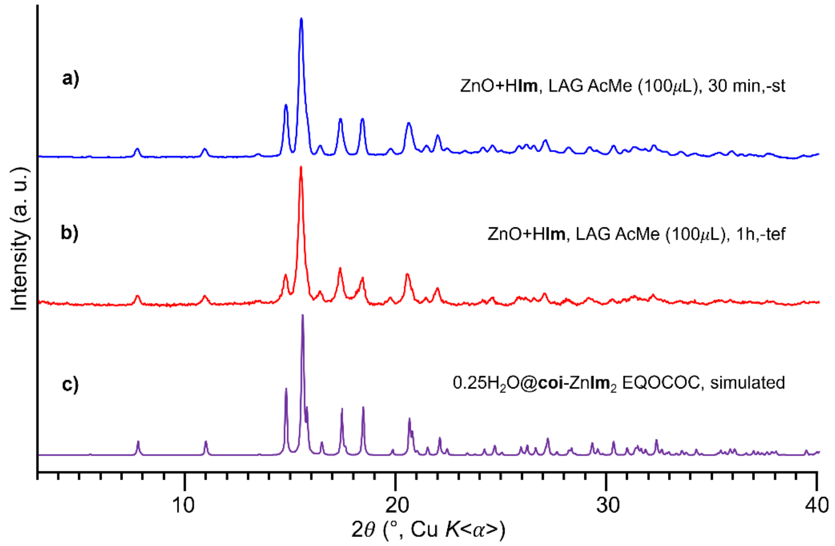

Figure S2. PXRD patterns of the products of milling ZnO and HIm with acetone for a) 30 min in a steel jar and b) 60 min in a Teflon™ jar. c) Simulated PXRD pattern of 0.25H_2O@**coi**-Zn**Im**$_2$ (CSD code EQOCOC).

2.1.3. Acetophenone (AcPhe)

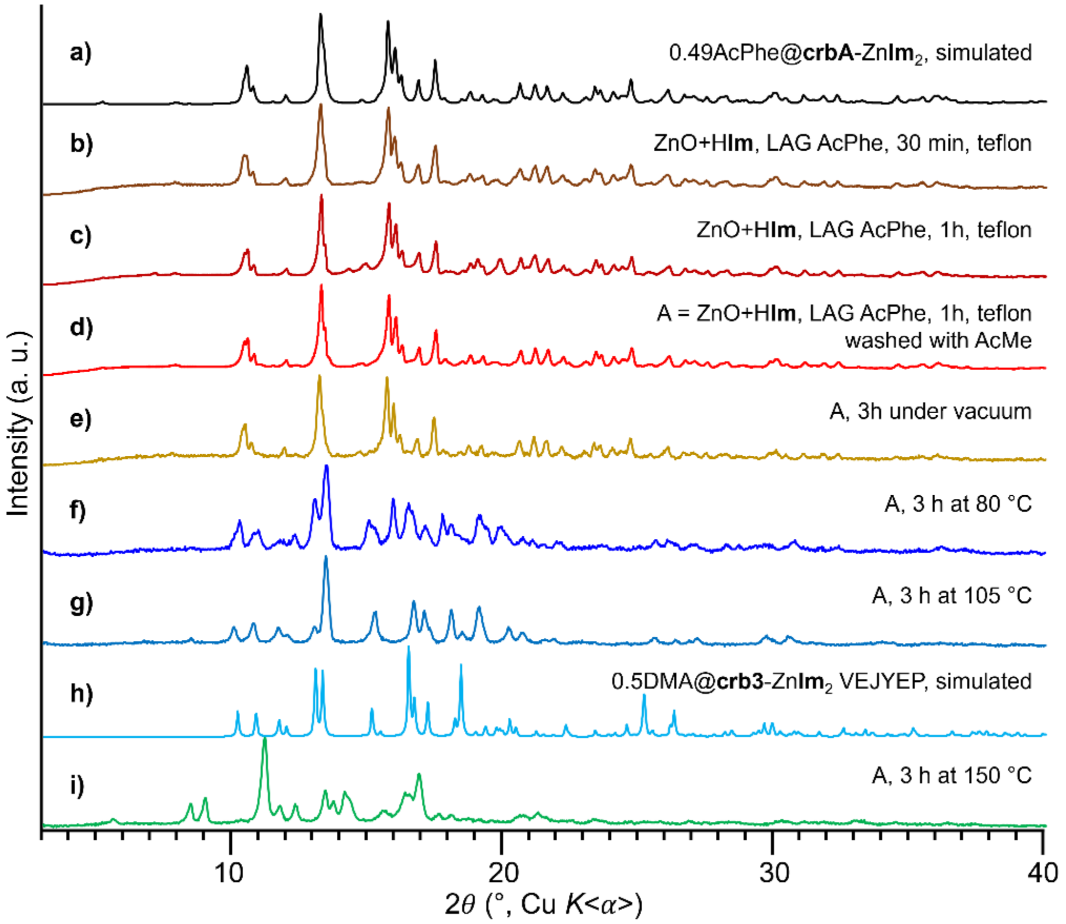

Figure S3. Simulated PXRD patterns of a) 0.49AcPhe@**crbA**-Zn**Im**$_2$ (solved from PXRD data), and h) 0.5DMA@**crb3**-Zn**Im**$_2$ (CSD code VEJYEP). PXRD patterns of b) the product of milling ZnO and H**Im** with AcPhe in a Teflon™ jar for 30 min; c) the product of milling ZnO and H**Im** with AcPhe in a Teflon™ jar for 1 h; d) A = the product of milling ZnO and H**Im** with AcPhe in a Teflon™ jar for 1 h, washed with acetone; e) A, under vacuum for 3 h; f) A, heated at 80 °C for 3 h; g) A, heated at 105 °C for 3 h; i) A, heated at 150 °C for 3 h.

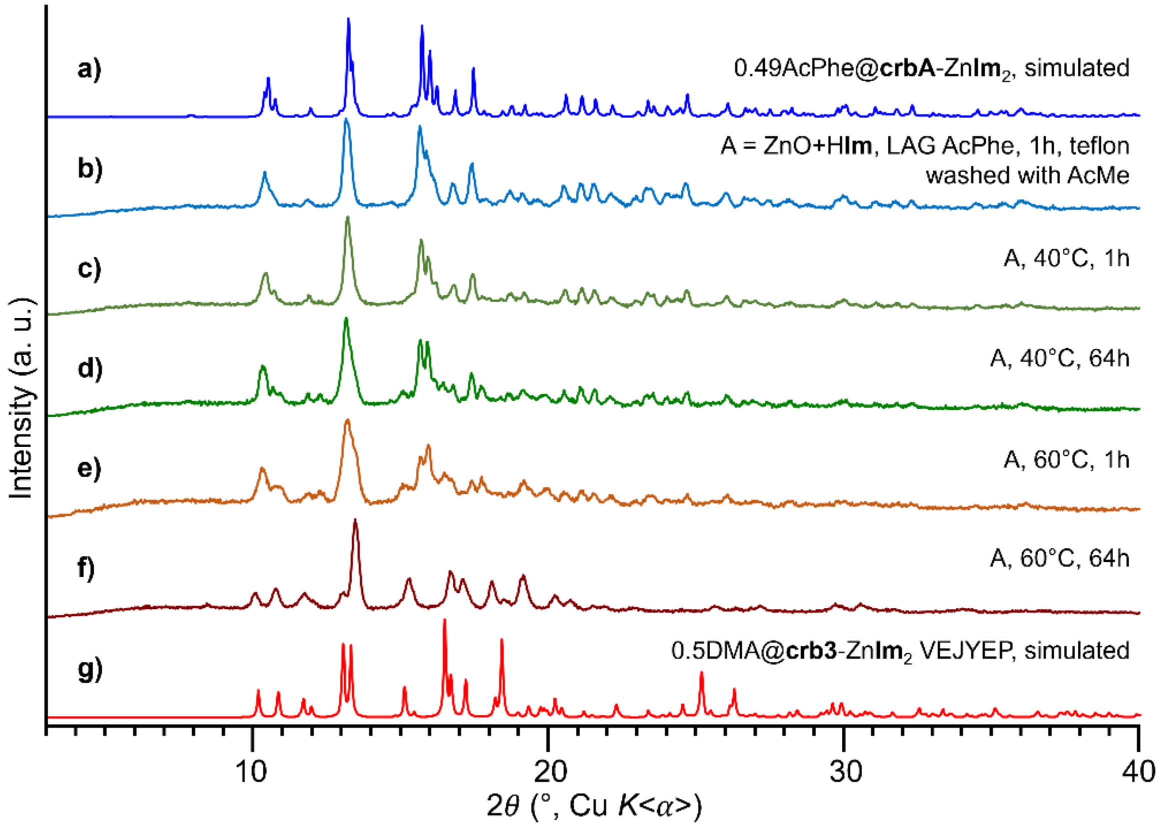

Figure S4. Simulated PXRD patterns of a) 0.49AcPhe@**crbA**-Zn**Im**$_2$ (solved from PXRD data), and g) 0.5DMA@**crb3**-Zn**Im**$_2$ (CSD code VEJYEP). PXRD patterns of b) A = the product of milling ZnO and H**Im** with AcPhe in a Teflon™ jar for 1 h, washed with acetone; c) A, heated at 40 °C for 1 h; d) A, heated at 40 °C for 64 h; e) A, heated at 60 °C for 1 h; f) A, heated at 60 °C for 64 h;

2.1.4.4-Acetylpyridine (4AcPyr)

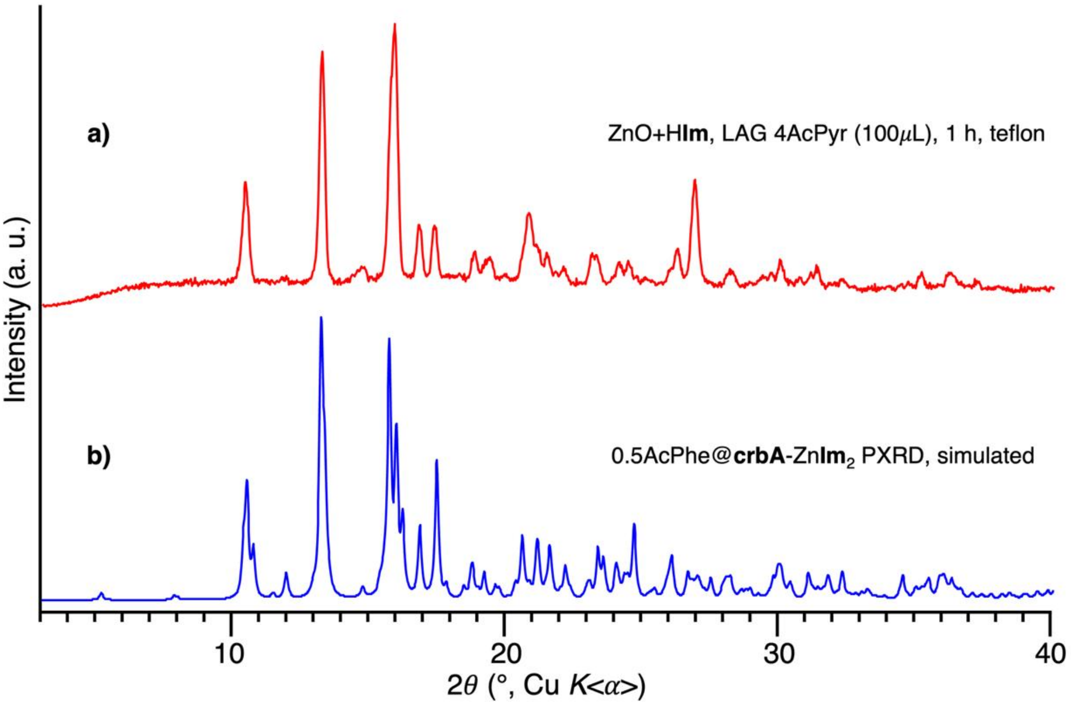

Figure S5. a) PXRD pattern of the product of milling ZnO and H**Im** with 4AcPyr in a Teflon™ jar for 1 h. b) Simulated PXRD pattern of 0.51AcPhe@**crbA**-Zn**Im**$_2$ (solved from PXRD data).

2.1.5. Butyrolactone (BLAC)

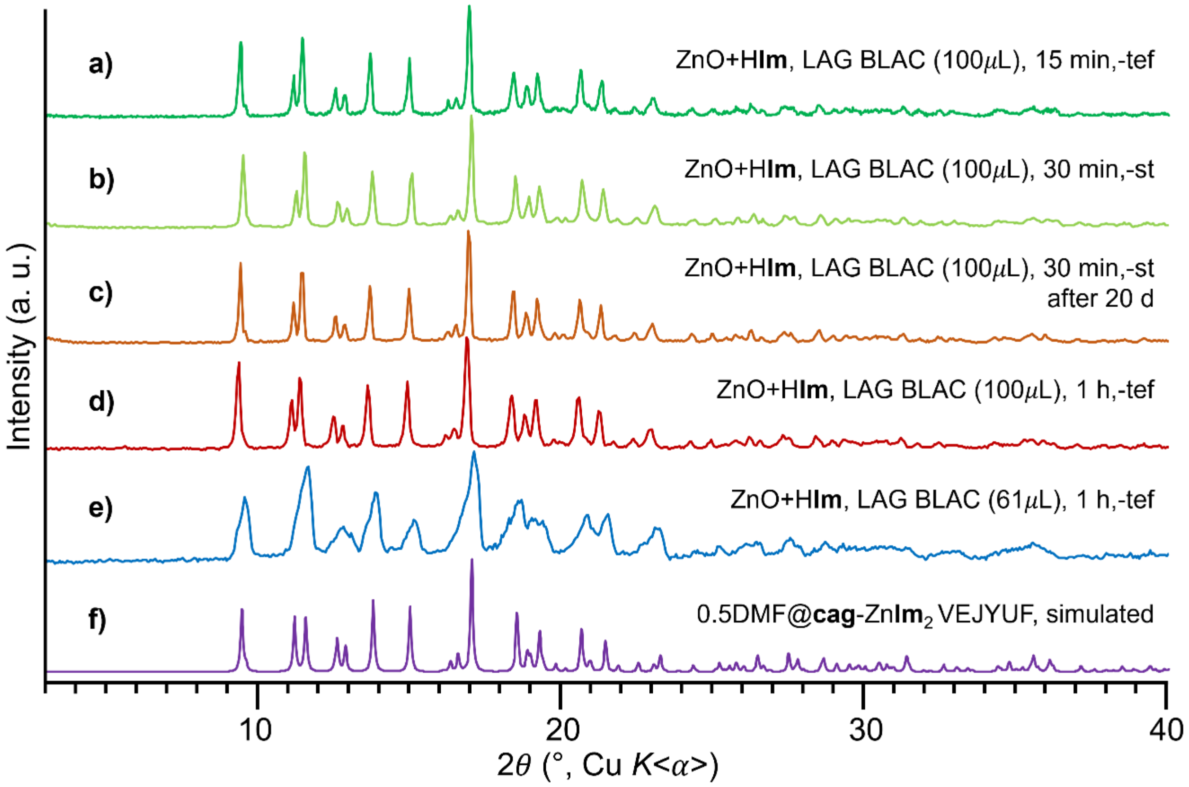

Figure S6. PXRD patterns of the products of milling ZnO and H**Im** with BLAC a) in a Teflon™ jar for 15 min, b) in a steel jar for 30 min, c) in a steel jar for 30 min, left standing in ambient conditions for 20 days, d) in a Teflon™ jar for 60 min, and e) in a Teflon™ jar for 60 min, using an equimolar amount of BLAC (61 μL). f) Simulated PXRD pattern of 0.5DMF@**cag**-Zn**Im**$_2$ (CSD code VEJYUF).

2.1.6. Cyclohexane (cHANE)

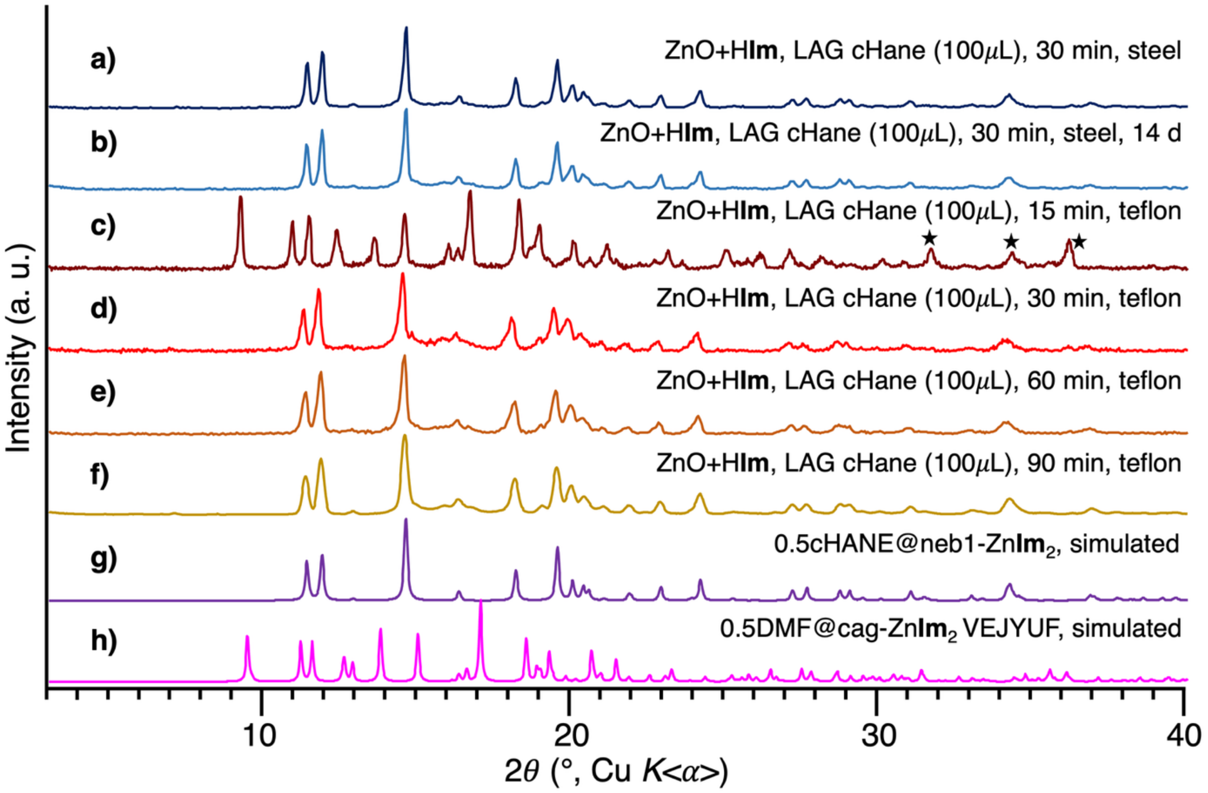

Figure S7. PXRD patterns of the products of milling ZnO and H**Im** with cHANE a) in a steel jar for 30 min, b) in a steel jar for 30 min, left standing in ambient conditions for 14 days, c) in a Teflon™ jar for 15 min, d) in a Teflon™ jar for 30 min, e) in a Teflon™ jar for 60 min, f) in a Teflon™ jar for 90 min. Simulated PXRD patterns of g) 0.5cHANE@**neb1**-Zn**Im**$_2$, and h) 0.5DMF@**cag**-Zn**Im**$_2$ (CSD code VEJYUF). Black stars denote peaks of leftover ZnO reagent.

2.1.7. Chloroform ($CHCl_3$)

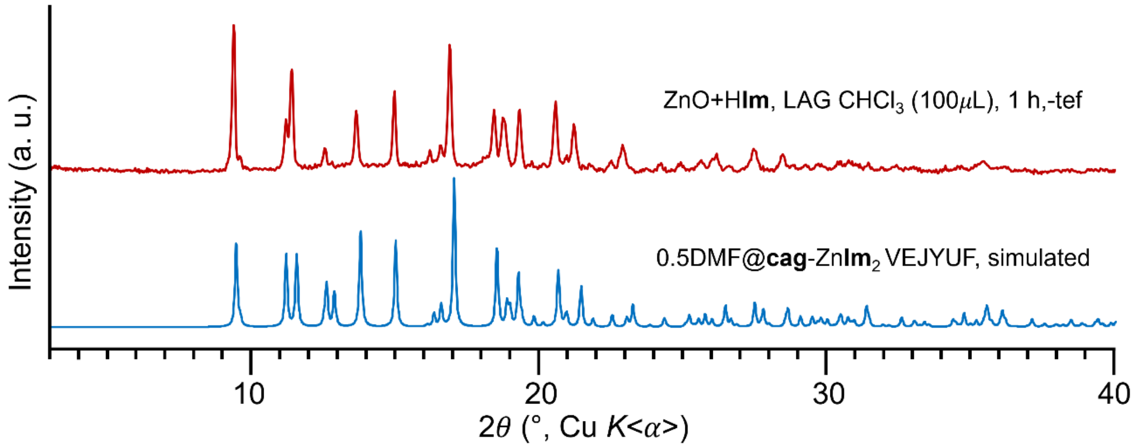

Figure S8. PXRD pattern of the producs of milling ZnO and H**Im** with $CHCl_3$ in a Teflon™ jar for 60 min (top). Simulated PXRD pattern of 0.5$CHCl_3$@**cag**-Zn**Im**$_2$ (from SCXRD data, bottom).

2.1.8. Cyclohexene (cHENE)

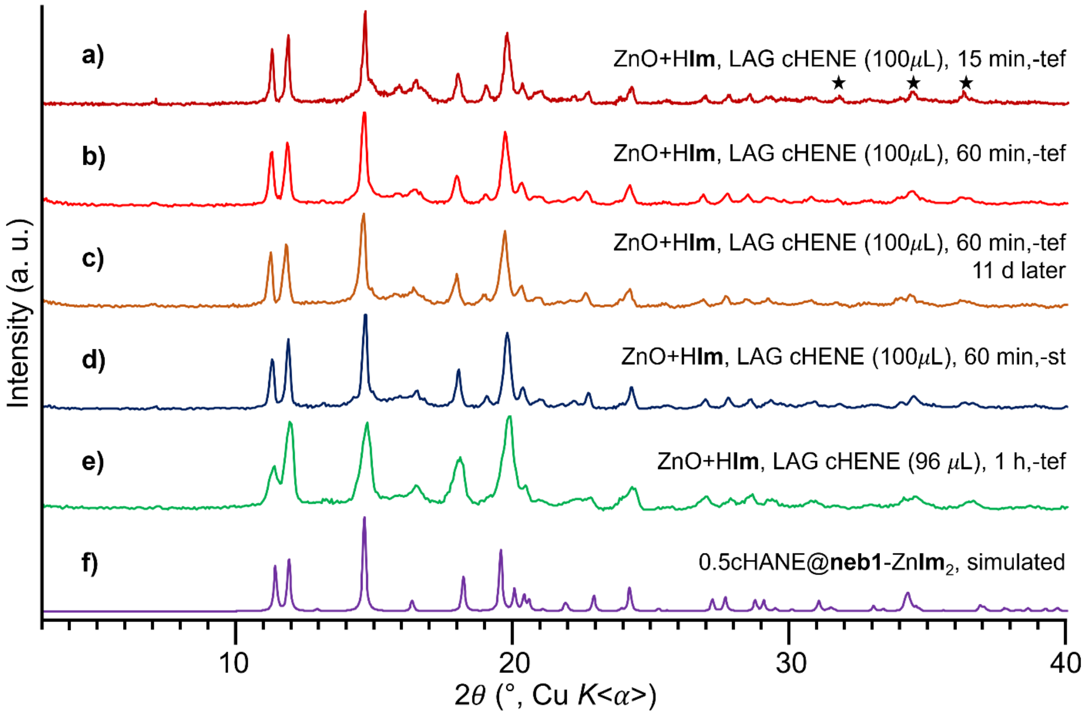

Figure S9. PXRD patterns of the products of milling ZnO and H**Im** with cHENE a) in a Teflon™ jar for 15 min; b) in a Teflon™ jar for 60 min; c) in a Teflon™ jar for 60 min, left standing in ambient conditions for 11 days; d) in a steel jar for 60 min; e) in a Teflon™ jar for 60 min, using an equimolar amount of cHENE (96 µL). f) Simulated PXRD pattern of 0.5cHANE@**neb1**-Zn**Im**$_2$. Black stars denote peaks of leftover ZnO reagent.

2.1.9. Cyclohexanone (cHONE)

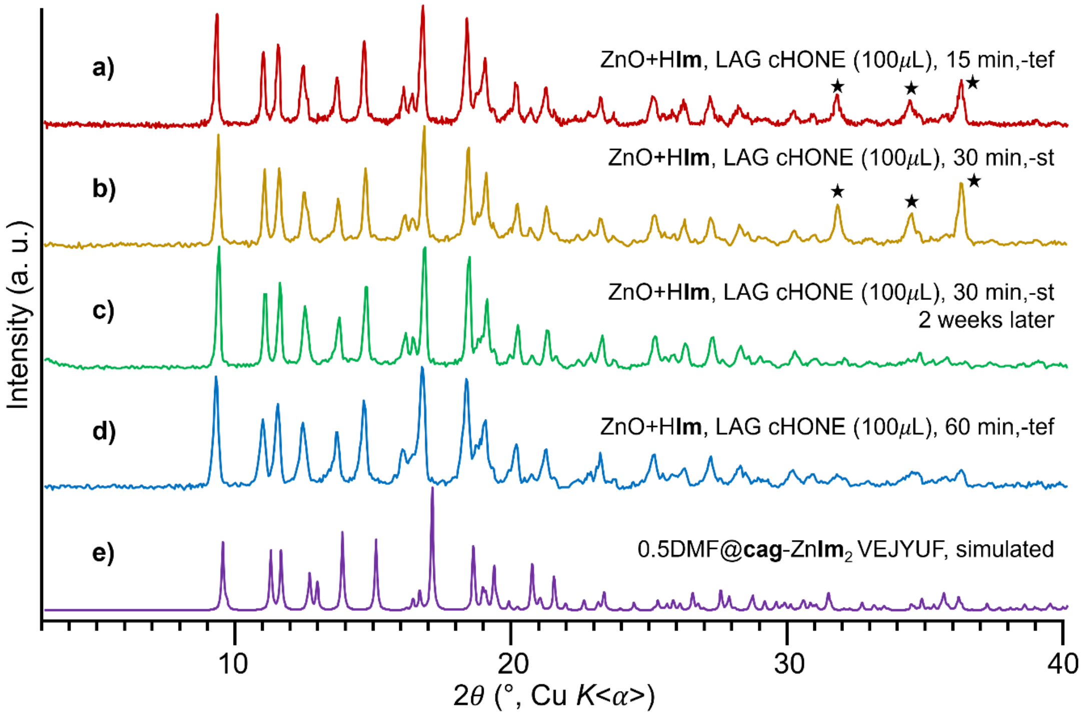

Figure S10. PXRD patterns of the products of milling ZnO and H**Im** with cHONE a) in a Teflon™ jar for 15 min; b) in a steel jar for 30 min; c) in a steel jar for 30 min, left standing in ambient conditions for 14 days; d) in a Teflon™ jar for 60 min. e) Simulated PXRD pattern of 0.5DMF@**cag**-Zn**Im**$_2$ (CSD code VEJYUF). Black stars denote peaks of leftover ZnO reagent.

2.1.10. ε-Caprolactone (CLAC)

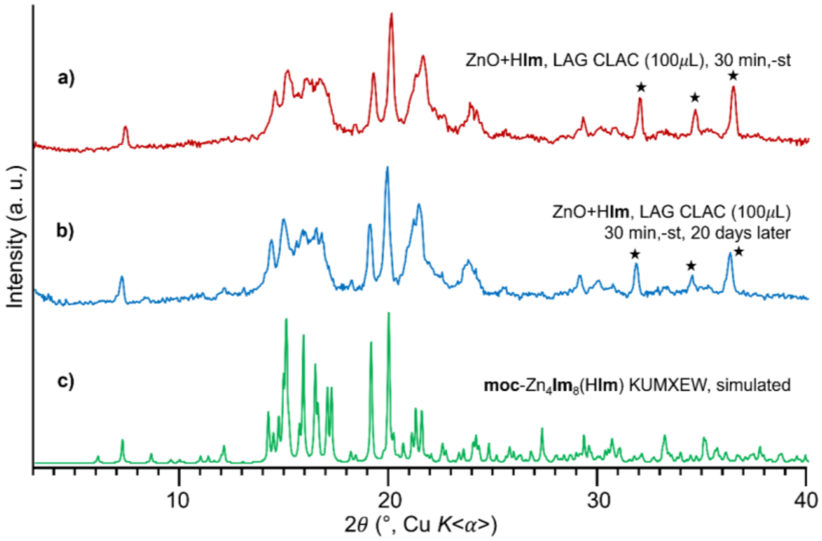

Figure S11. PXRD patterns of the products of milling ZnO and H**Im** with CLAC a) in a steel jar for 30 min; b) in a steel jar for 30 min, left standing in ambient conditions for 20 days. c) Simulated PXRD pattern of **moc**-Zn_4**Im**$_8$H**Im** (CSD code KUMXEW). Black stars denote peaks of leftover ZnO reagent.

2.1.11. N,N-dibutylformamide (DBF)

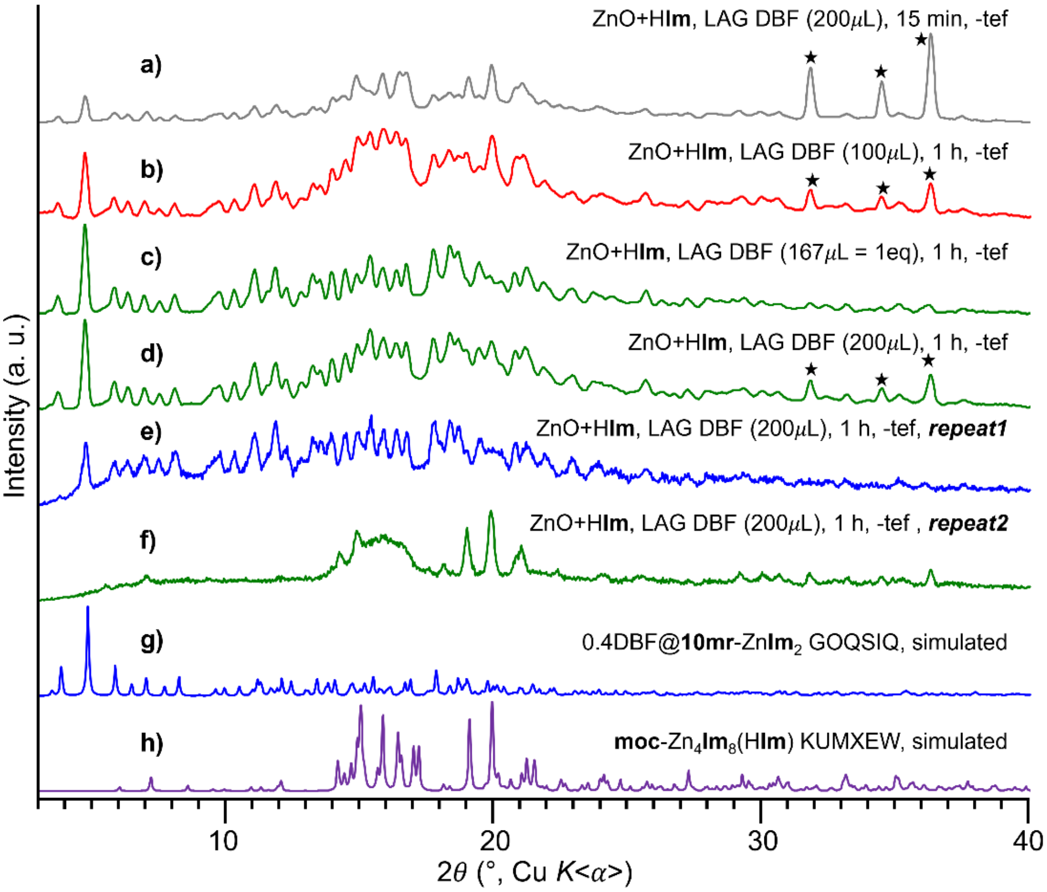

Figure S12. PXRD patterns of the products of milling ZnO and H**Im** with DBF in a Teflon jar a) for 15 min with 200 μL of DBF; b) for 60 min with 100 μL of DBF; c) for 60 min with 167 μL of DBF; d) for 60 min with 200 μL of DBF; e) for 60 min with 200 μL of DBF, repeat experiment 1; f) for 60 min with 200 μL of DBF, repeat experiment 2. Simulated PXRD patterns of g) 0.4DBF@**10mr**-Zn**Im**$_2$ (CSD code GOQSIQ) and h) **moc**-Zn_4**Im**$_8$H**Im** (CSD code KUMXEW). Black stars denote peaks of leftover ZnO reagent.

2.1.12. *N,N-diethylformamide (DEF)*

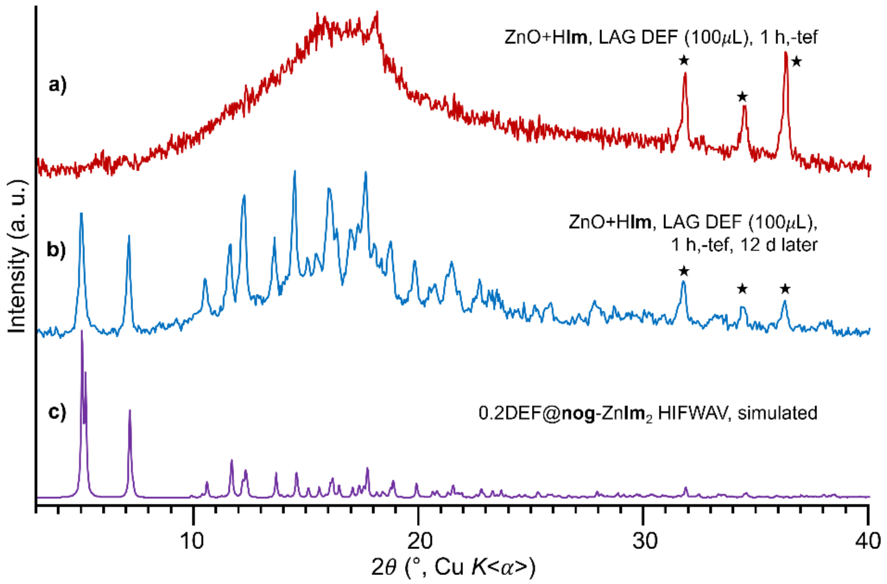

Figure S13. PXRD patterns of the products of milling ZnO and H**Im** with DEF in a Teflon™™ jar a) for 60 min; b) for 60 min, left standing in ambient conditions for 12 days. C) Simulated PXRD patterns of 0.2DEF@**nog**-Zn**Im**$_2$ (CSD code HIFWAV). Black stars denote peaks of leftover ZnO reagent.

2.1.13. *N,N-dipropylformamide (DPF)*

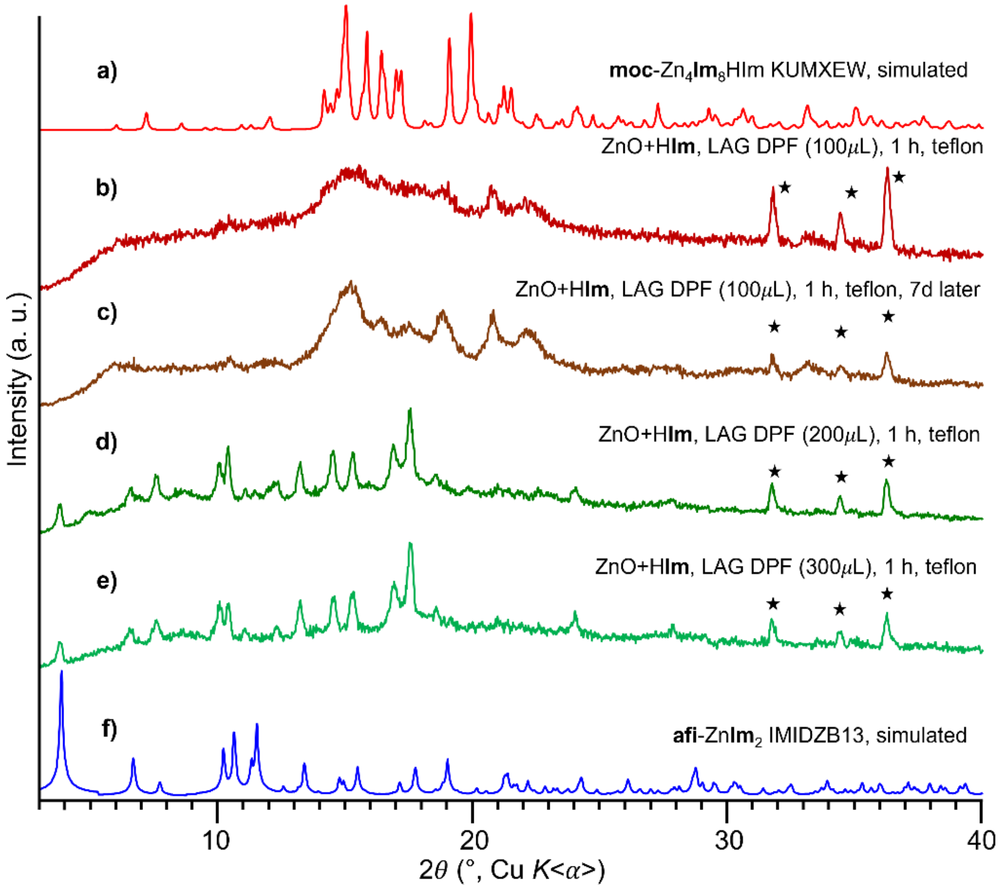

Figure S14. Simulated PXRD patterns of a) **moc**-Zn$_4$**Im**$_8$H**Im** (CSD code KUMXEW) and f) **afi**-Zn**Im**$_2$ (CSD code IMIDZB13), plotted as the square root of intensity on the y axis, for easier peak identification. PXRD patterns of the products of milling ZnO and H**Im** in a Teflon™ jar for 60 min with: b) 100 µL of DPF; d) 200 µL of DPF; e) 300 µL of DPF. c) product of b after 7d. Black stars denote peaks of leftover ZnO reagent.

2.1.14. *1,4-dioxane (DIOX)*

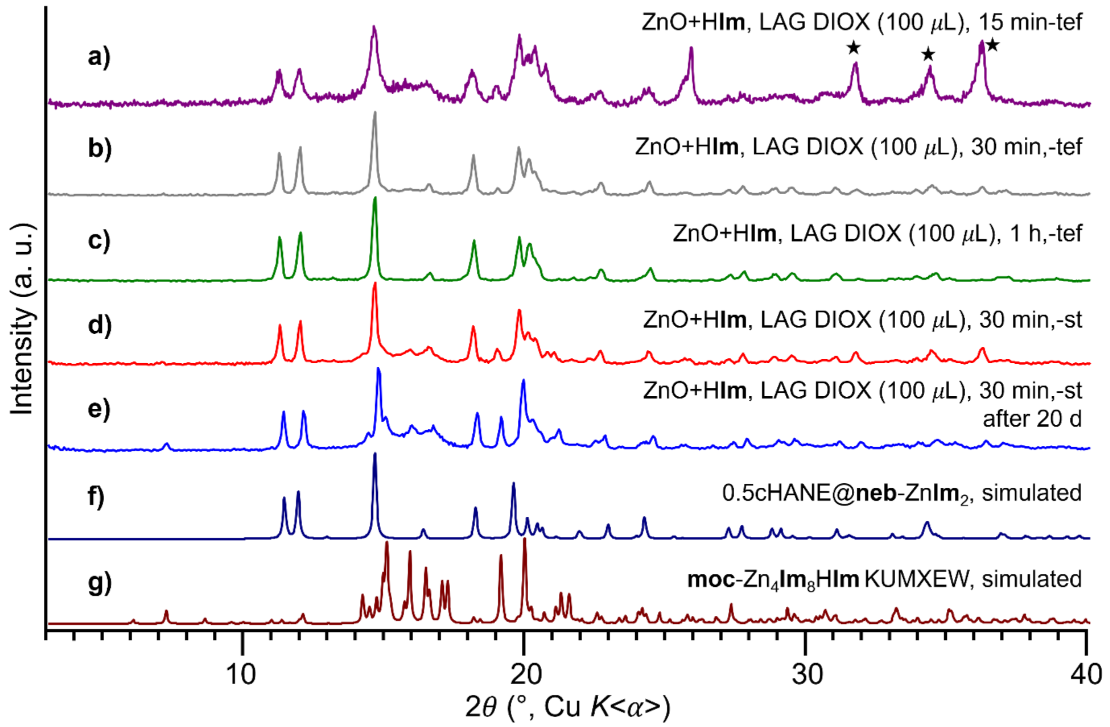

Figure S15. PXRD patterns of the products of milling ZnO and H**Im** with DIOX a) in a Teflon™ jar for 15 min, b) in a Teflon™ jar for 30 min, c) in a Teflon™ jar for 60 min, d) in a steel jar for 30 min, e) in a steel jar for 30 min, left standing in ambient conditions for 20 days. Simulated PXRD patterns of f) 0.5cHANE@**neb1**-Zn**Im**$_2$, and g) **moc**-Zn$_4$**Im**$_8$H**Im** (CSD code KUMXEW). Black stars denote peaks of leftover ZnO reagent.

2.1.15. *N,N-dimethylacetamide (DMA)*

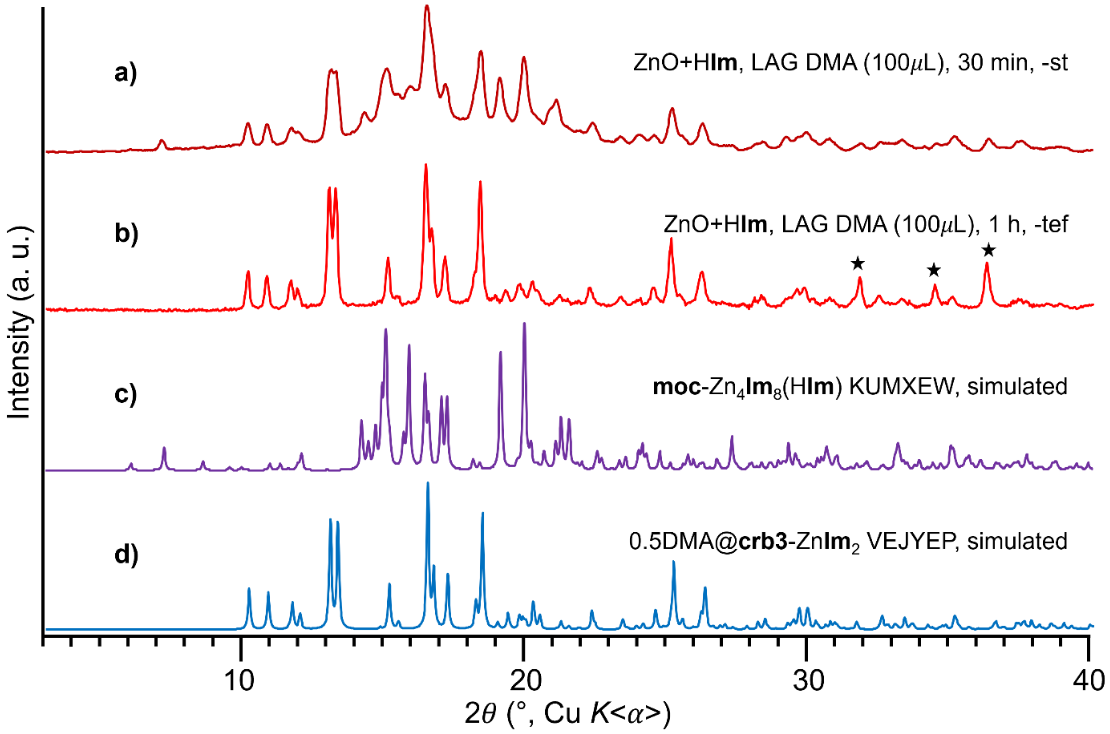

Figure S16. PXRD patterns of the products of milling ZnO and H**Im** with DMA in a) a steel jar for 30 min; b) a Teflon™ jar for 60 min. Simulated PXRD patterns of c) **moc**-Zn$_4$**Im**$_8$H**Im** (CSD code KUMXEW) and d) 0.5DMA@**crb3**-Zn**Im**$_2$ (CSD code VEJYEP). Black stars denote peaks of leftover ZnO reagent.

2.1.16. N,N-dimethylformamide (DMF)

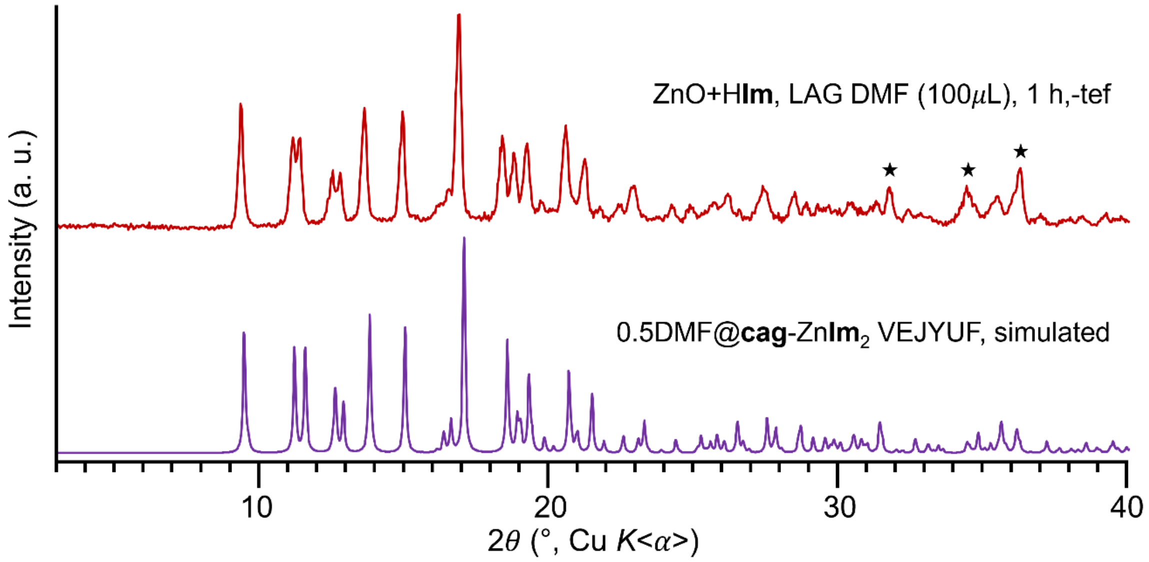

Figure S17. PXRD pattern of the product of milling ZnO and H**Im** with DMF in a Teflon™ jar for 60 min (top). Simulated PXRD pattern of of 0.5DMF@**cag**-Zn**Im**$_2$ (CSD code VEJYUF, bottom). Black stars denote peaks of leftover ZnO reagent.

2.1.17. Dimethylsulfoxide (DMSO)

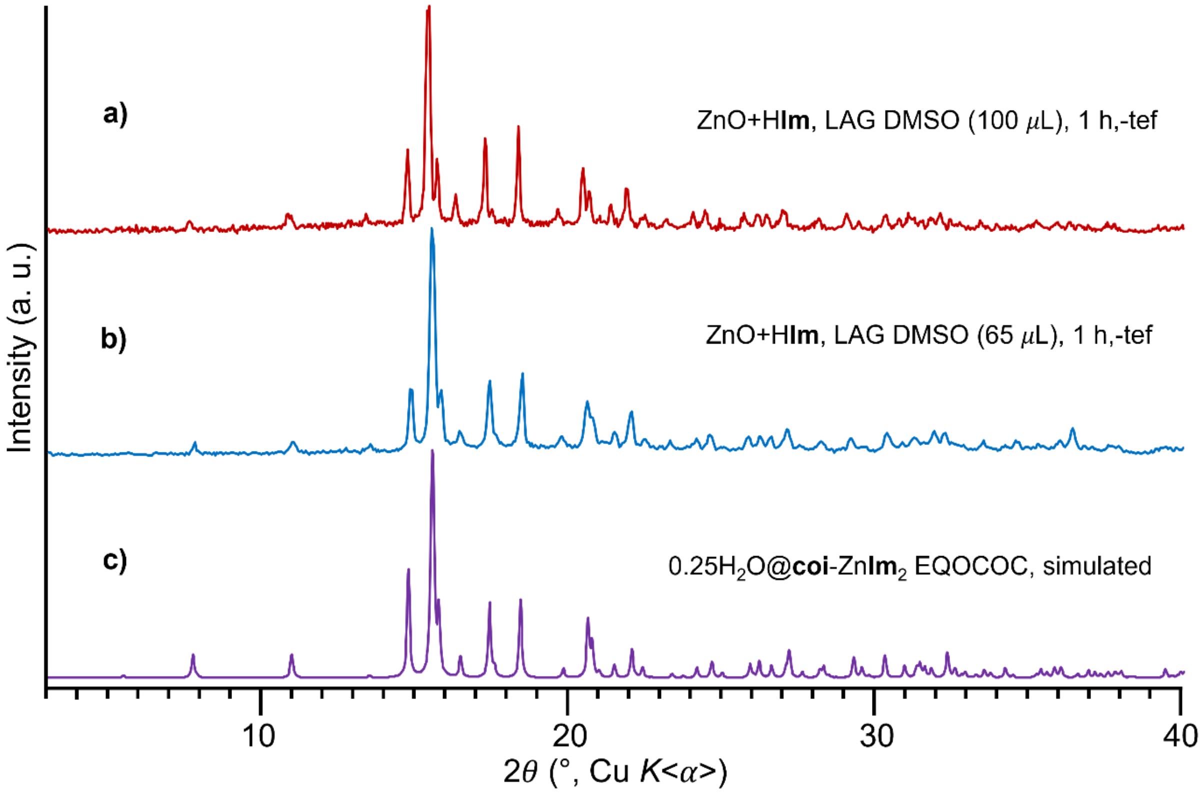

Figure S18. PXRD patterns of the products of milling ZnO and H**Im** with DMSO a) in a Teflon™ jar for 60 min; b) in a Teflon™ jar for 60 min, using an equimolar amount of DMSO (65 μL). c) Simulated PXRD pattern of 0.25H_2O@**coi**-Zn**Im**$_2$ (CSD code EQOCOC).

2.1.18. Triethylamine (Et_3N)

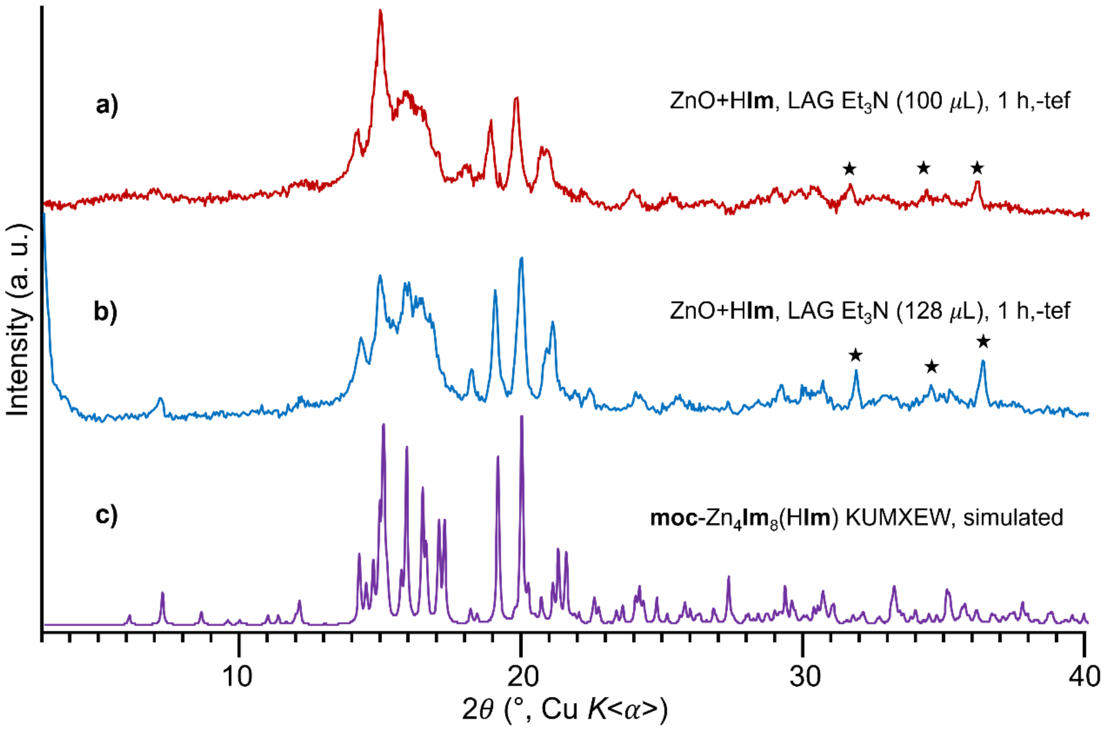

Figure S19. PXRD patterns of the products of milling ZnO and H**Im** with Et_3N a) in a Teflon™ jar for 60 min; b) in a Teflon™ jar for 60 min, using an equimolar amount of Et_3N (128 µL). Simulated PXRD pattern of c) **moc**-Zn_4**Im**$_8$H**Im** (CSD code KUMXEW). Black stars denote peaks of leftover ZnO reagent.

2.1.19. Ethylene glycol (EtGly)

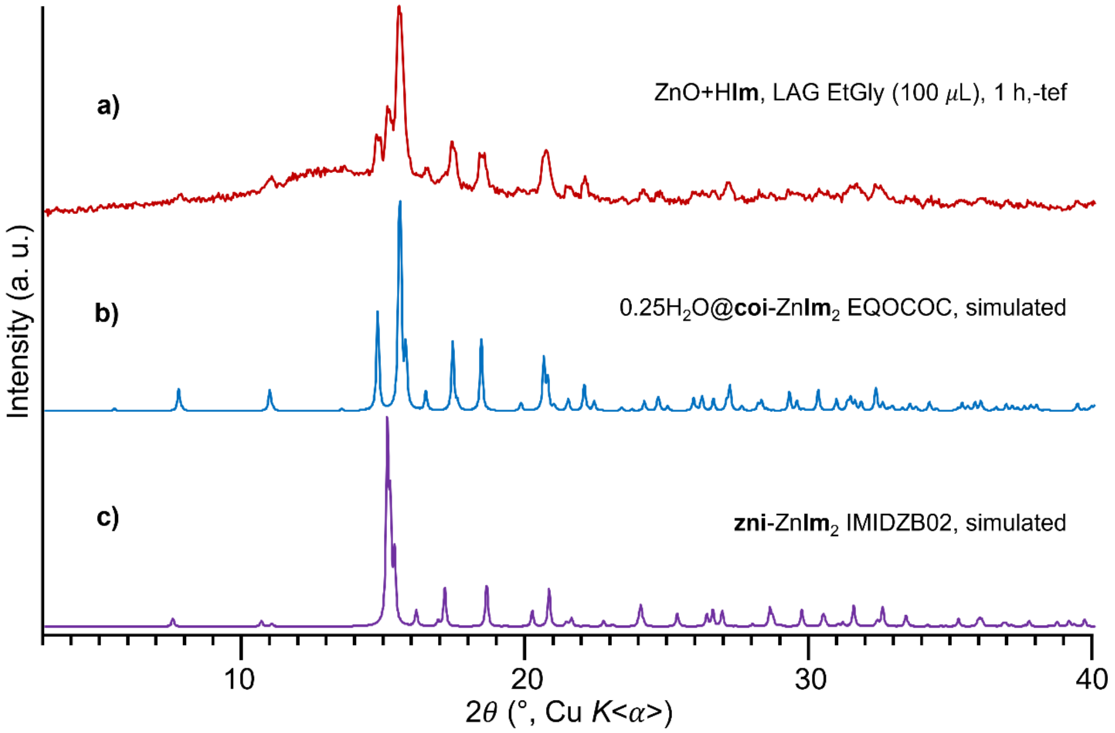

Figure S20. a) PXRD pattern of the product of milling ZnO and H**Im** with EtGly in a Teflon™ jar for 60 min. Simulated PXRD patterns of b) $0.25H_2O$@**coi**-Zn**Im**$_2$ (CSD code EQOCOC) and c) **zni**-Zn**Im**$_2$ (CSD code IMIDZB02).

2.1.20. Ethyl acetate (EtOAc)

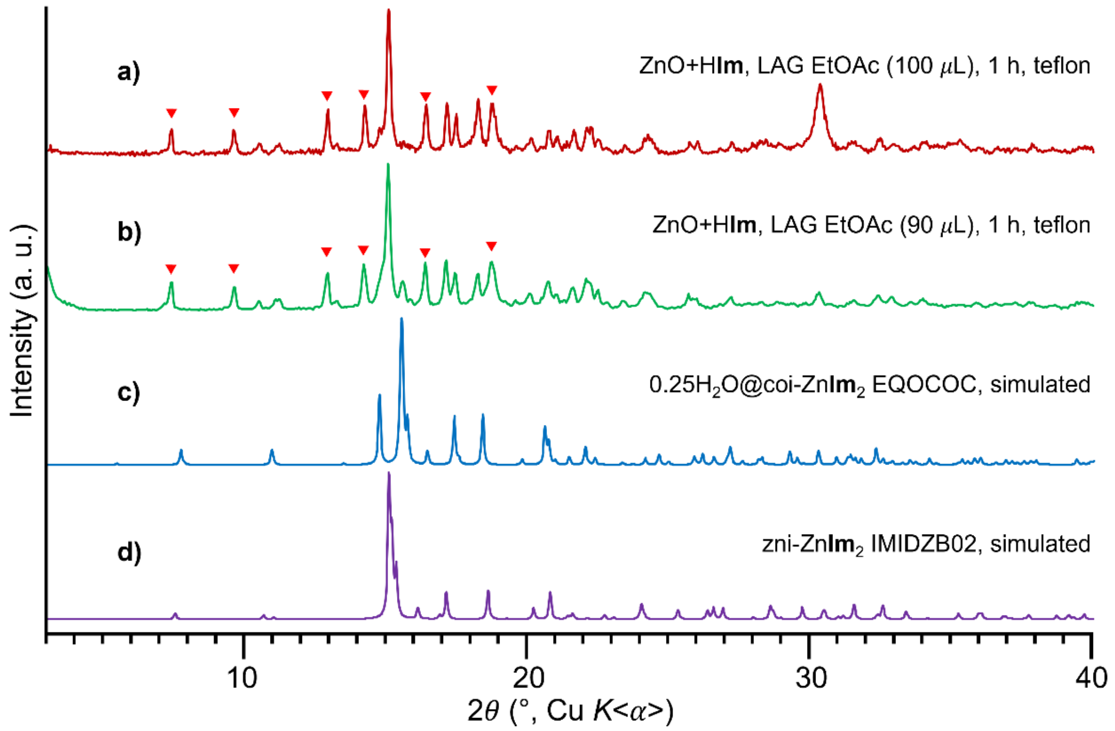

Figure S21. PXRD patterns of the products of milling ZnO and H**Im** with EtOAc a) in a Teflon™ jar for 60 min; b) in a Teflon™ jar for 60 min, using an equimolar amount of EtOAc (90 µL). Simulated PXRD patterns of c) $0.25H_2O$@**coi**-Zn**Im**$_2$ (CSD code EQOCOC) and d) **zni**-Zn**Im**$_2$ (CSD code IMIDZB02). Red triangles indicate peaks belonging to an unknown phase.

2.1.21. Ethanol (EtOH)

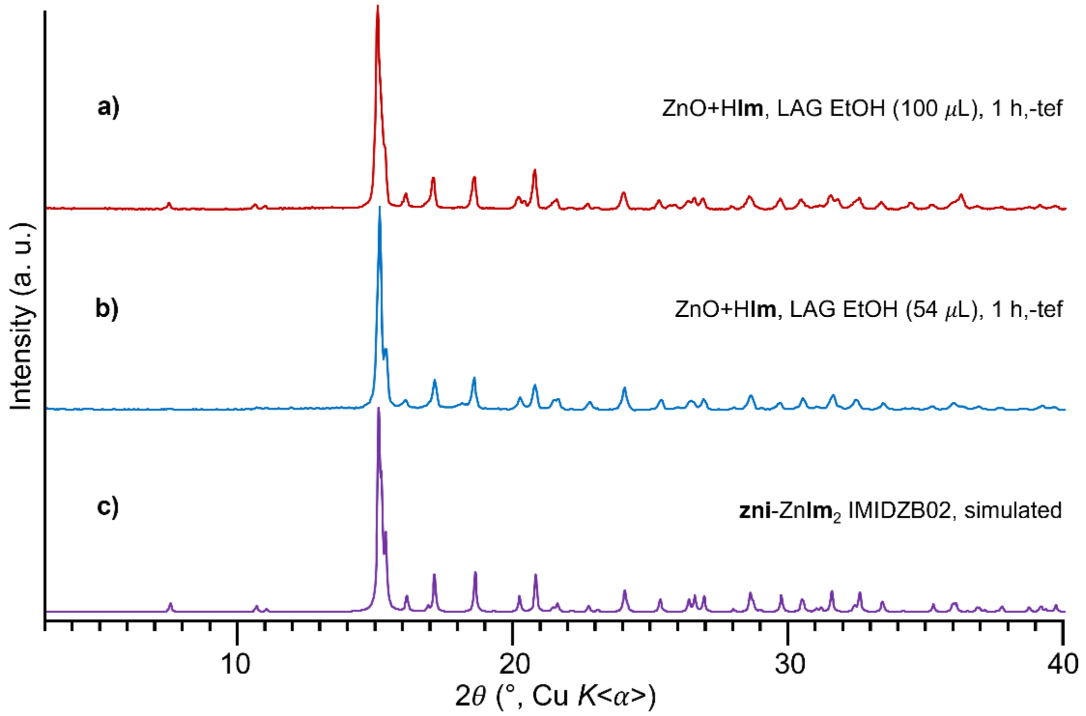

Figure S22. PXRD patterns of the products of milling ZnO and H**Im** with EtOH a) in a Teflon™ jar for 60 min; b) in a Teflon™ jar for 60 min, using an equimolar amount of EtOH (54 µL). Simulated PXRD pattern of c) **zni**-Zn**Im**$_2$ (CSD code IMIDZB02).

2.1.22. Furan (FUR)

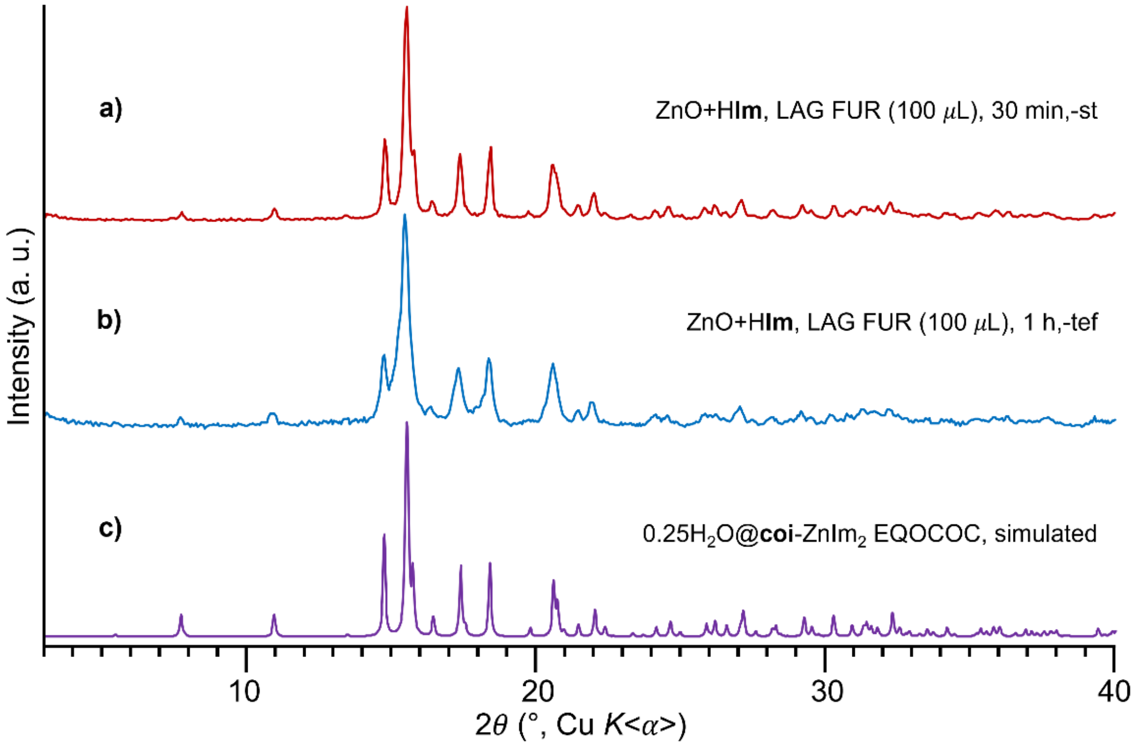

Figure S23. PXRD patterns of the products of milling ZnO and H**Im** with FUR a) in a steel jar for 30 min; b) in a Teflon™ jar for 60 min. Simulated PXRD pattern of c) 0.25H_2O@**coi**-Zn**Im**$_2$ (CSD code EQOCOC).

2.1.23. Water (H_2O)

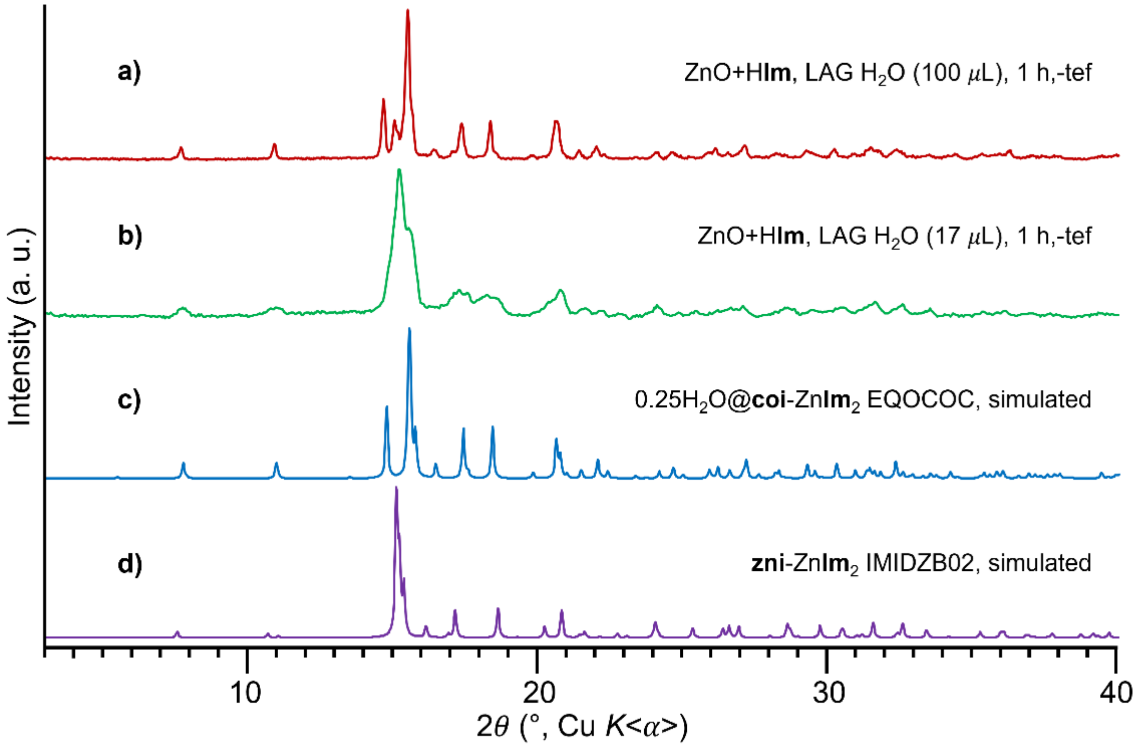

Figure S24. PXRD patterns of the products of milling ZnO and H**Im** with H_2O a) in a Teflon™ jar for 60 min; b) in a Teflon™ jar for 60 min, using an equimolar amount of H_2O (17 μL). Simulated PXRD patterns of c) 0.25H_2O@**coi**-Zn**Im**$_2$ (CSD code EQOCOC) and d) **zni**-Zn**Im**$_2$ (CSD code IMIDZB02).

2.1.24. Hexamethylphosphoramide (HMPA)

CAUTION! HMPA is a possible carcinogen.

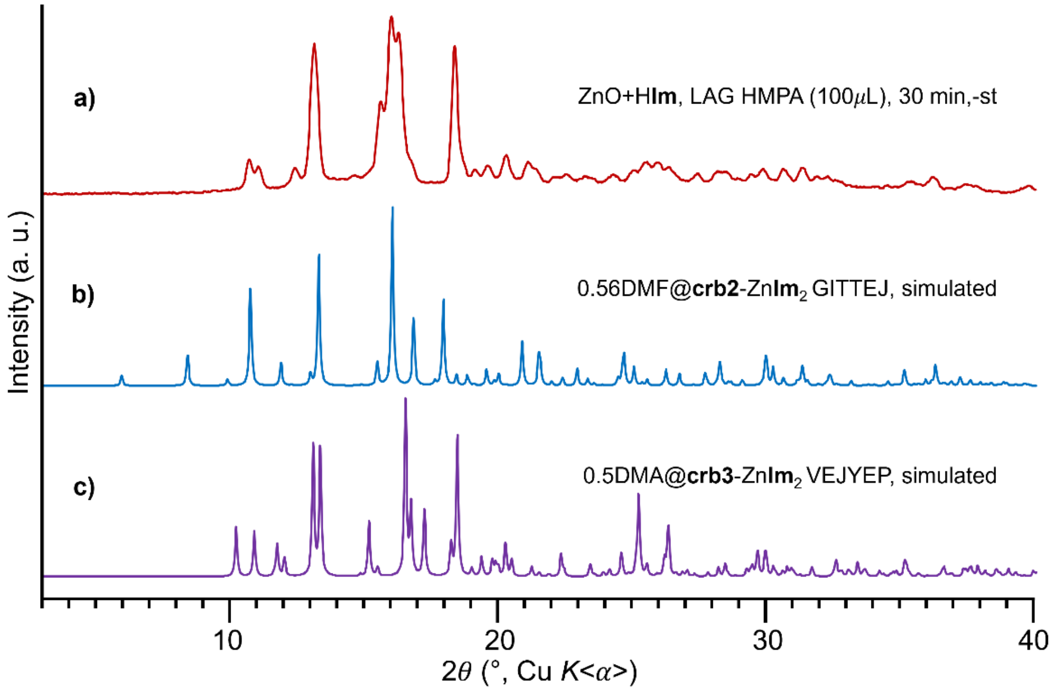

Figure S25. a) PXRD pattern of the product of milling ZnO and H**Im** with HMPA for 30 min in a steel jar. Simulated PXRD patterns of b) 0.56DMF@**crb2**-Zn**Im**$_2$ (CSD code GITTEJ) and c) 0.5DMA@**crb3**-Zn**Im**$_2$ (CSD code VEJYEP).

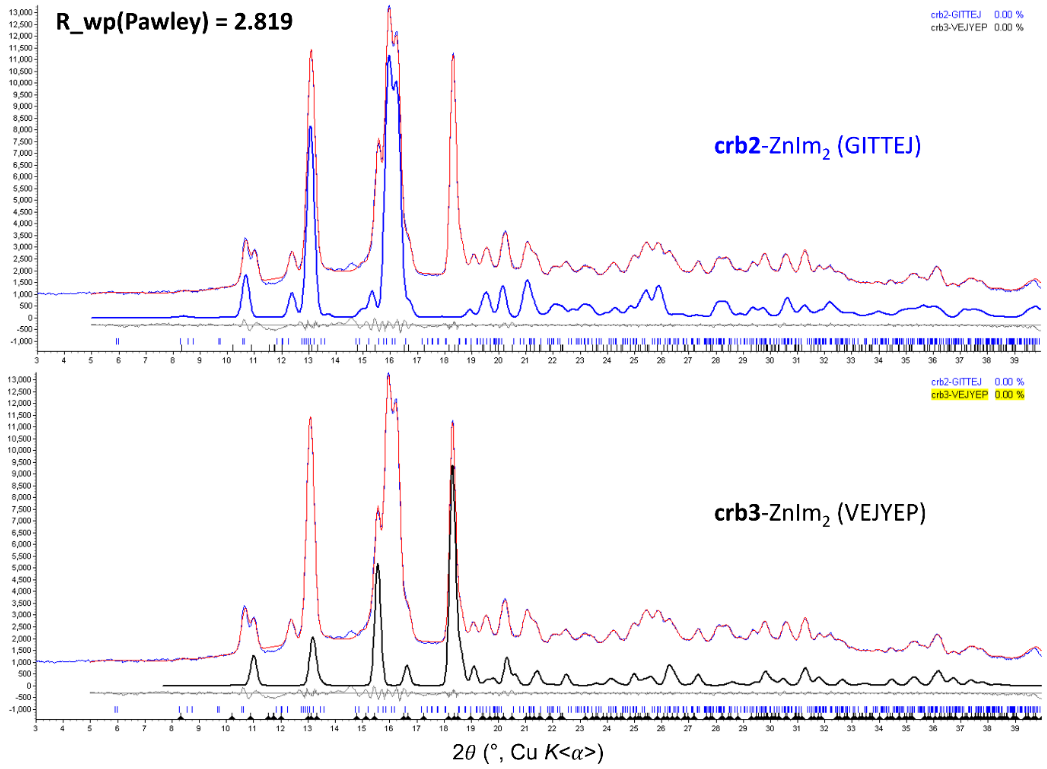

Figure S26. Pawley fitting of 0.56DMF@**crb2**-Zn**Im**$_2$ (CSD code GITTEJ) and 0.5DMA@**crb3**-Zn**Im**$_2$ (CSD code VEJYEP) to the experimental PXRD pattern obtained by milling ZnO and H**Im** with HMPA for 30 min in a steel jar. The experimental pattern is in light blue, the Pawley fit in red, and the difference curve in grey. Top panel displays the **crb2** contribution (dark blue), and the bottom panel shows the **crb3** contribution (black) to the overall fit.

2.1.25. Acetonitrile (MeCN)

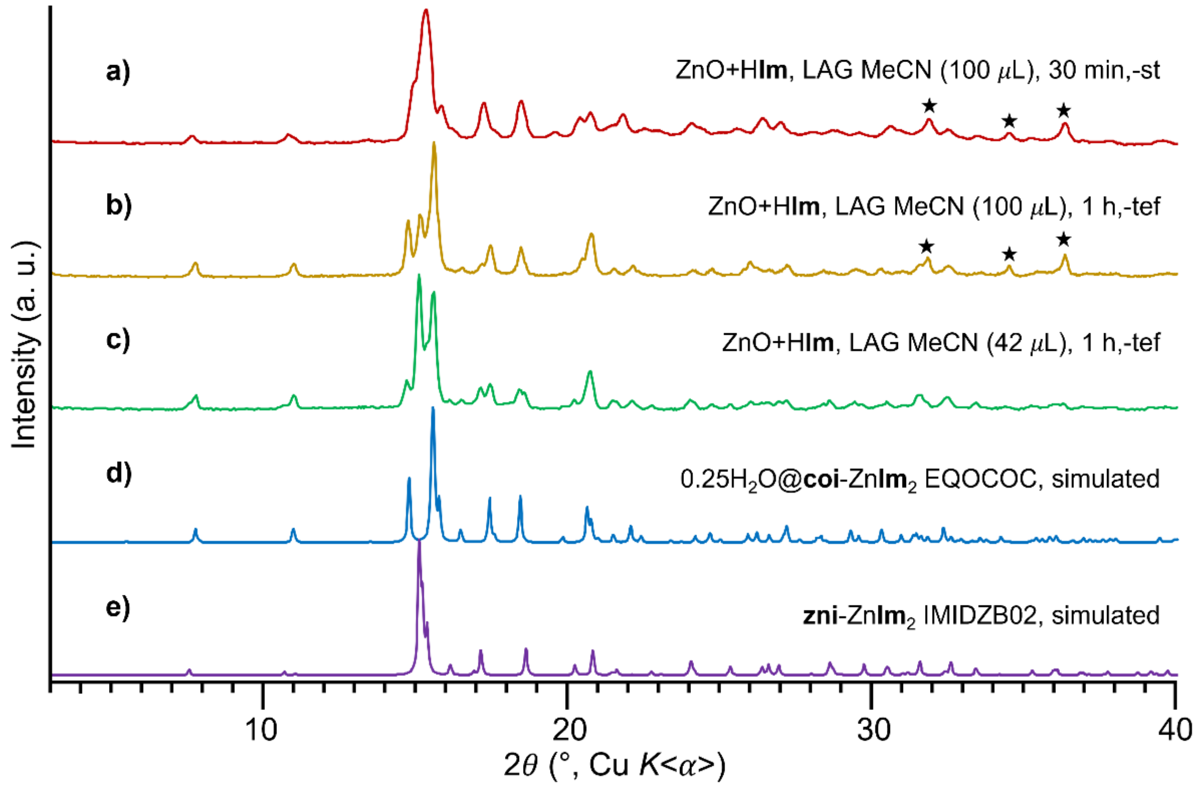

Figure S27. PXRD patterns of the products of milling ZnO and H**Im** with MeCN a) in a steel jar for 30 min; b) in a Teflon™ jar for 60 min; c) in a Teflon™ jar for 60 min, using an equimolar amount of MeCN (42 μL). Simulated PXRD patterns of d) $0.25H_2O$@**coi**-Zn**Im**$_2$ (CSD code EQOCOC) and e) **zni**-Zn**Im**$_2$ (CSD code IMIDZB02). Black stars denote peaks of leftover ZnO reagent.

2.1.26. Nitromethane ($MeNO_2$)

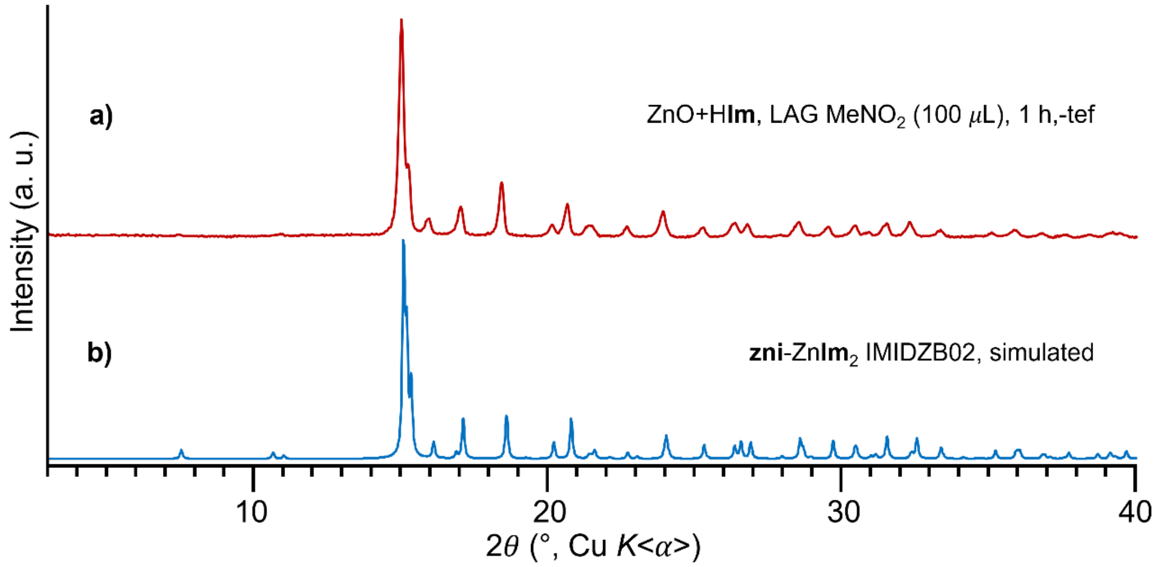

Figure S28. PXRD patterns of the products of milling ZnO and H**Im** with $MeNO_2$ a) in a Teflon™ jar for 60 min. Simulated PXRD pattern of b) **zni**-Zn**Im**$_2$ (CSD code IMIDZB02).

2.1.27. Methanol (MeOH)

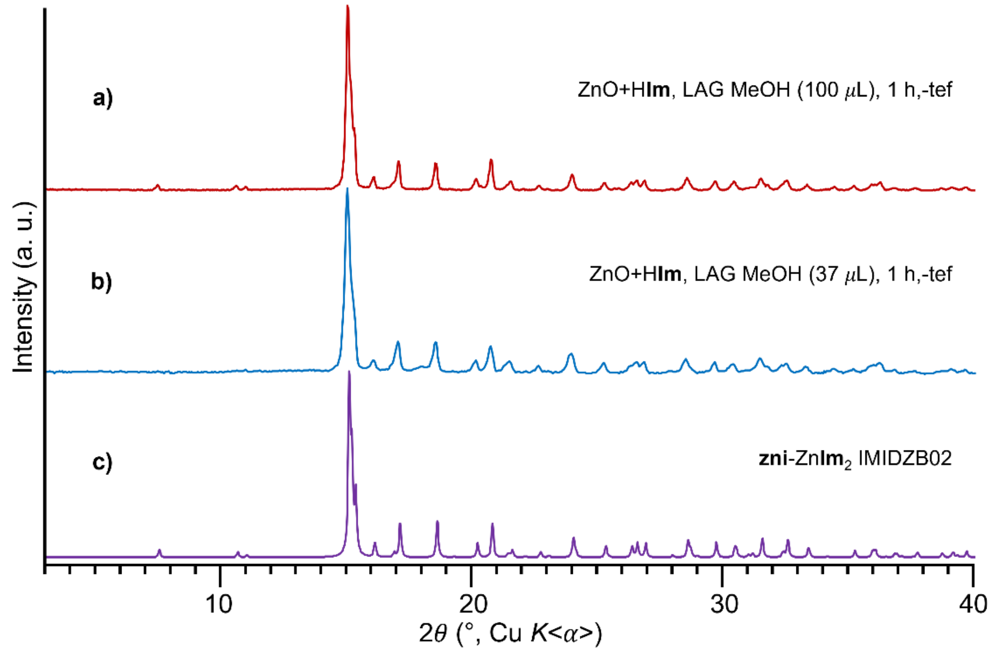

Figure S29. PXRD patterns of the products of milling ZnO and H**Im** with MeOH a) in a Teflon™ jar for 60 min; b) in a Teflon™ jar for 60 min, using an equimolar amount of MeOH (37 µL). Simulated PXRD pattern of c) **zni**-Zn**Im**$_2$ (CSD code IMIDZB02).

2.1.28. 4-methylpyridine (4-MePyr)

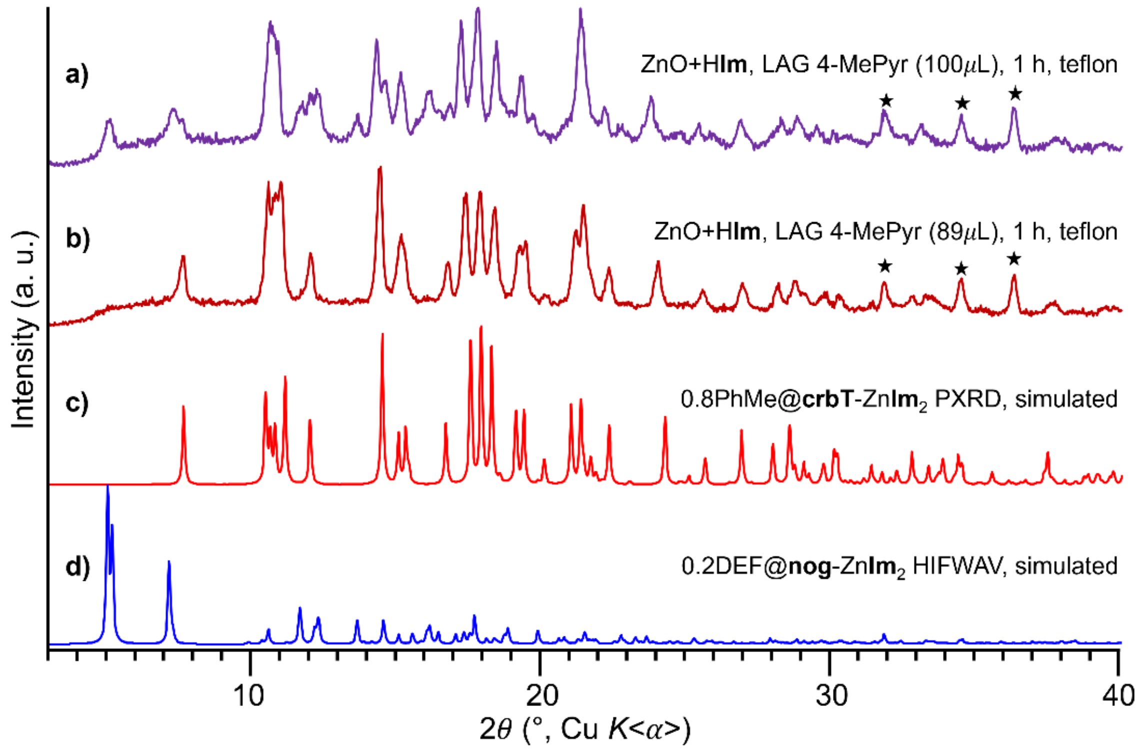

Figure S30. PXRD patterns of the product of milling ZnO and H**Im** with 4-MePyr a) in a Teflon™ jar for 1 h; b) in a Teflon™ jar for 60 min, using an equimolar amount of 4-MePyr (89 µL). Simulated PXRD patterns of c) 0.65PhMe@**crbT**-Zn**Im**$_2$ (solved from PXRD data) and d) 0.2DEF@**nog**-Zn**Im**$_2$ (CSD code HIFWAV). Black stars denote peaks of leftover ZnO reagent.

2.1.29. *Morpholine (MORPH)*

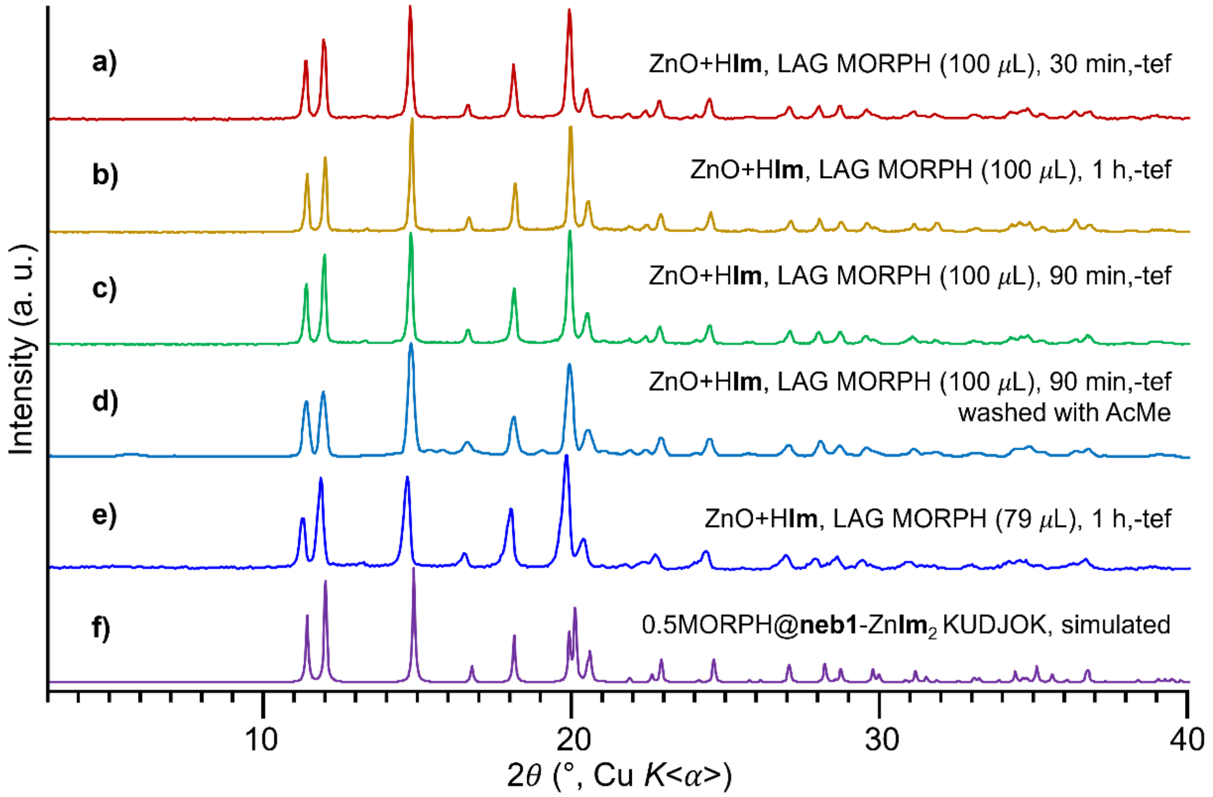

Figure S31. PXRD patterns of the products of milling ZnO and H**Im** with MORPH a) in a Teflon™ jar for 30 min; b) in a Teflon™ jar for 60 min; c) in a Teflon™ jar for 90 min; d) in a Teflon™ jar for 90 min, then washed with acetone; e) in a Teflon™ jar for 60 min, using an equimolar amount of MORPH (79 μL). f) Simulated PXRD pattern of 0.5MORPH@**neb1**-Zn**Im**$_2$ (CSD code KUDJOK).

2.1.30. *Butan-1-ol (nBuOH)*

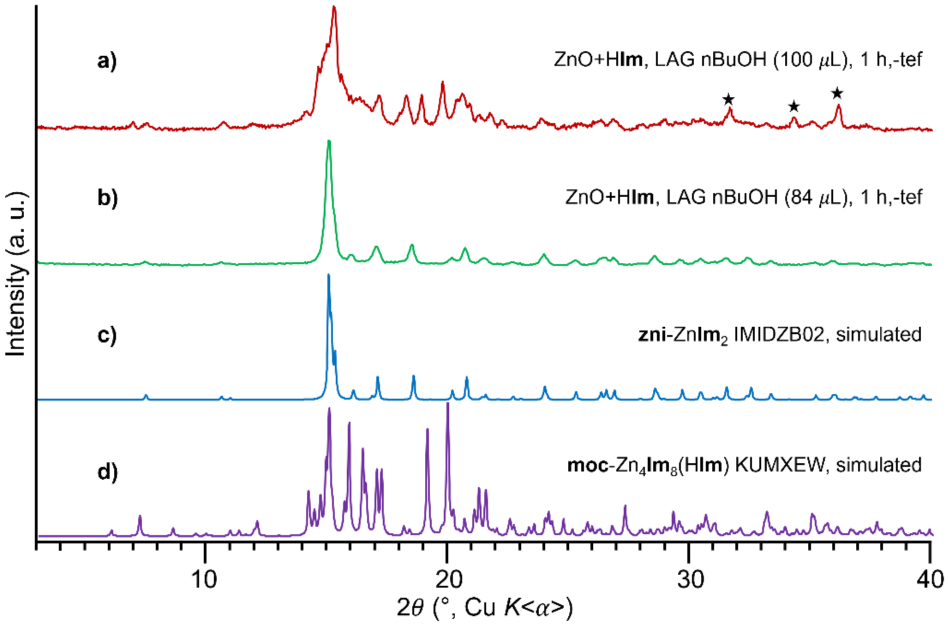

Figure S32. PXRD patterns of the products of milling ZnO and H**Im** with nBuOH a) in a Teflon™ jar for 60 min; b) in a Teflon™ jar for 60 min, using an equimolar amount of nBuOH (84 μL). Simulated PXRD patterns of c) **zni**-Zn**Im**$_2$ (CSD code IMIDZB02) and d) **moc**-Zn$_4$**Im**$_8$H**Im** (CSD code KUMXEW). Black stars denote peaks of leftover ZnO reagent.

2.1.31. N-methylcaprolactam (NMC)

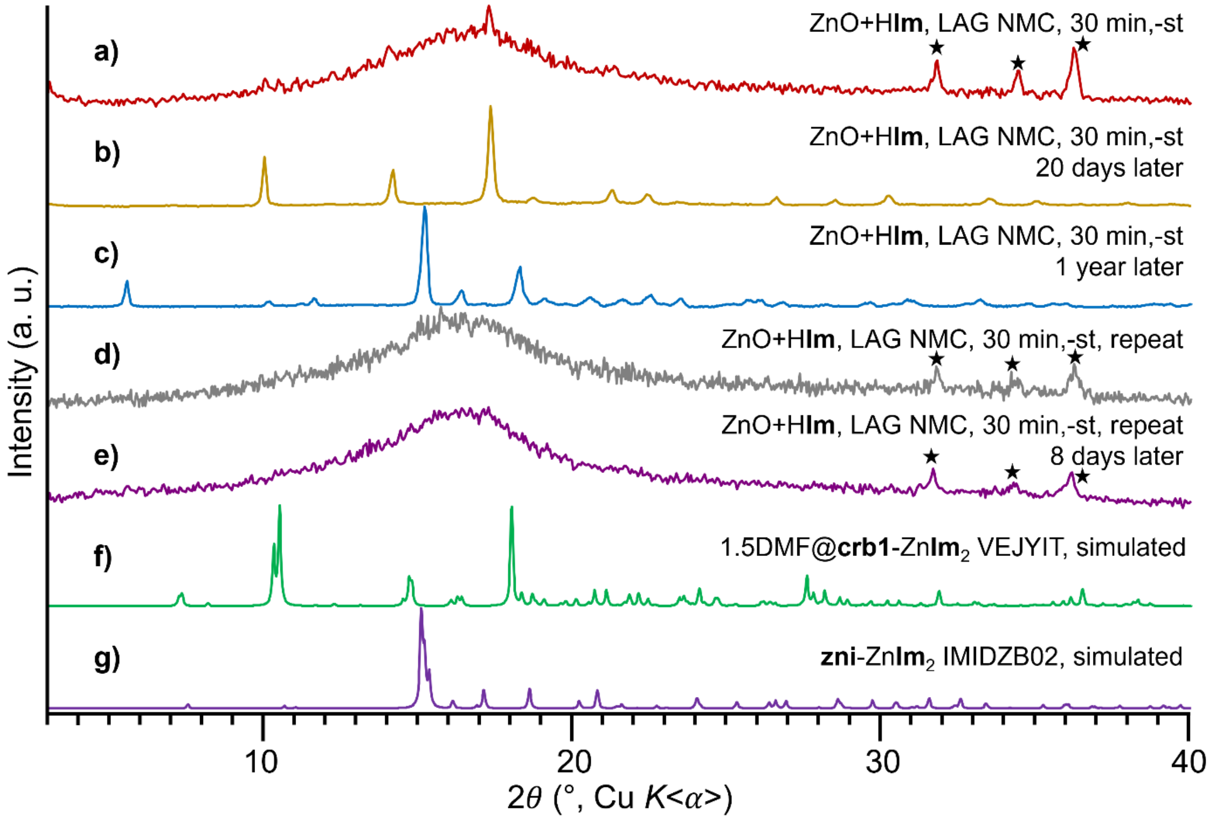

Figure S33. PXRD patterns of the products of milling ZnO and H**Im** with NMC a) in a steel jar for 30 min; b) product of a after 20 days; c) product of a after 1 year; d) in a steel jar for 30 min, repeated experiment; e) product of d after 8 days. g) Simulated PXRD pattern of **zni**-Zn**Im**$_2$ (CSD code IMIDZB02). Black stars denote peaks of leftover ZnO reagent.

2.1.32. N-Methyl-2-pyrrolidone (NMP)

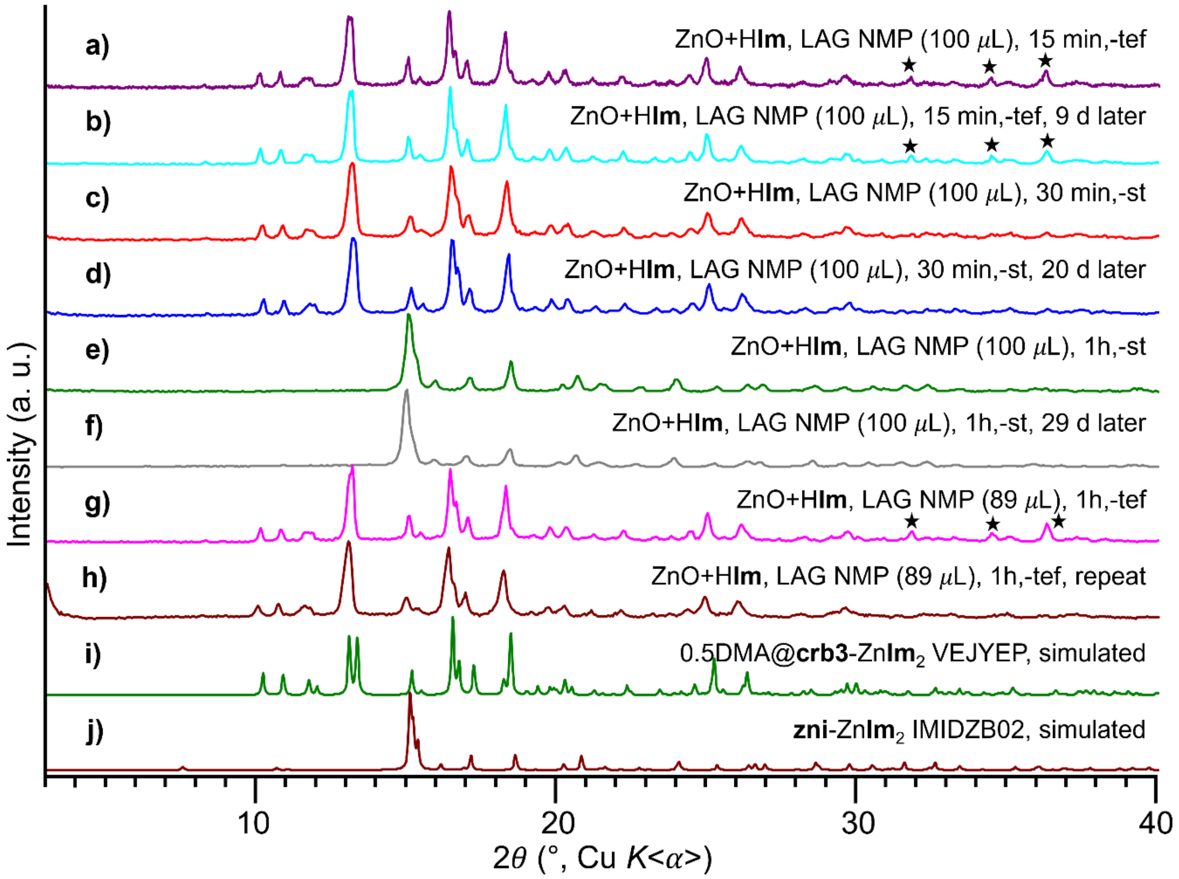

Figure S34. PXRD patterns of the products of milling ZnO and H**Im** with NMP a) in a Teflon™ jar for 15 min; b) product of a after 9 days; c) in a steel jar for 30 min; d) product of c after 20 days; e) in a steel jar for 60 min; f) product of e after 29 days; g) in a Teflon™ jar for 60 min, using an equimolar amount of NMP (89 μL); h) in a Teflon™ jar for 60 min, using an equimolar amount of NMP (89 μL), repeated experiment. Simulated PXRD patterns of i) 0.5DMA@**crb3**-Zn**Im**$_2$ (CSD code VEJYEP) and j) **zni**-Zn**Im**$_2$ (CSD code IMIDZB02). Black stars denote peaks of leftover ZnO reagent.

2.1.33. N-methyl-2-piperidone (NMPd)

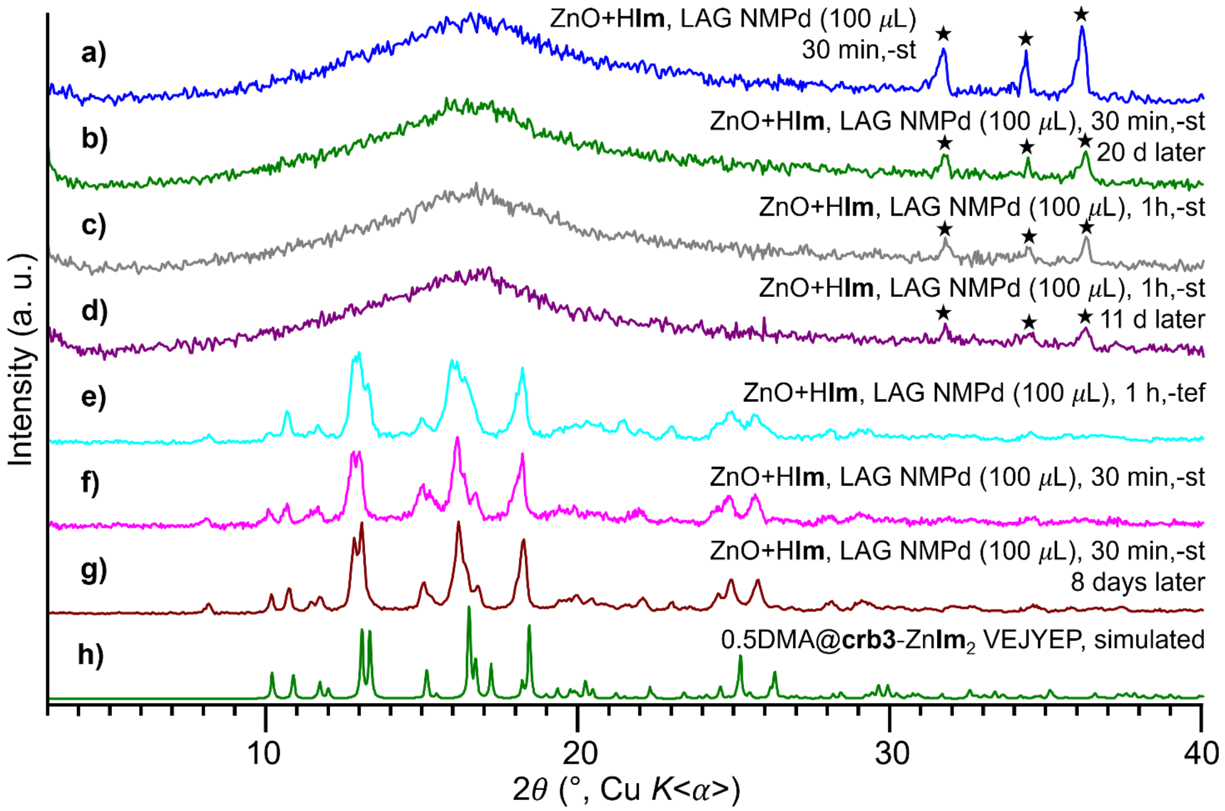

Figure S35. PXRD patterns of the products of milling ZnO and H**Im** with NMP a) in a steel jar for 30 min; b) product of a after 20 days; c) in a steel jar for 60 min; d) product of c after 11 days; e) in a Teflon™ jar for 60 min; f) in a steel jar for 30 min, repeated experiment; g) product of f after 8 days. h) Simulated PXRD pattern of 0.5DMA@**crb3**-Zn**Im**$_2$ (CSD code VEJYEP). Black stars denote peaks of leftover ZnO reagent.

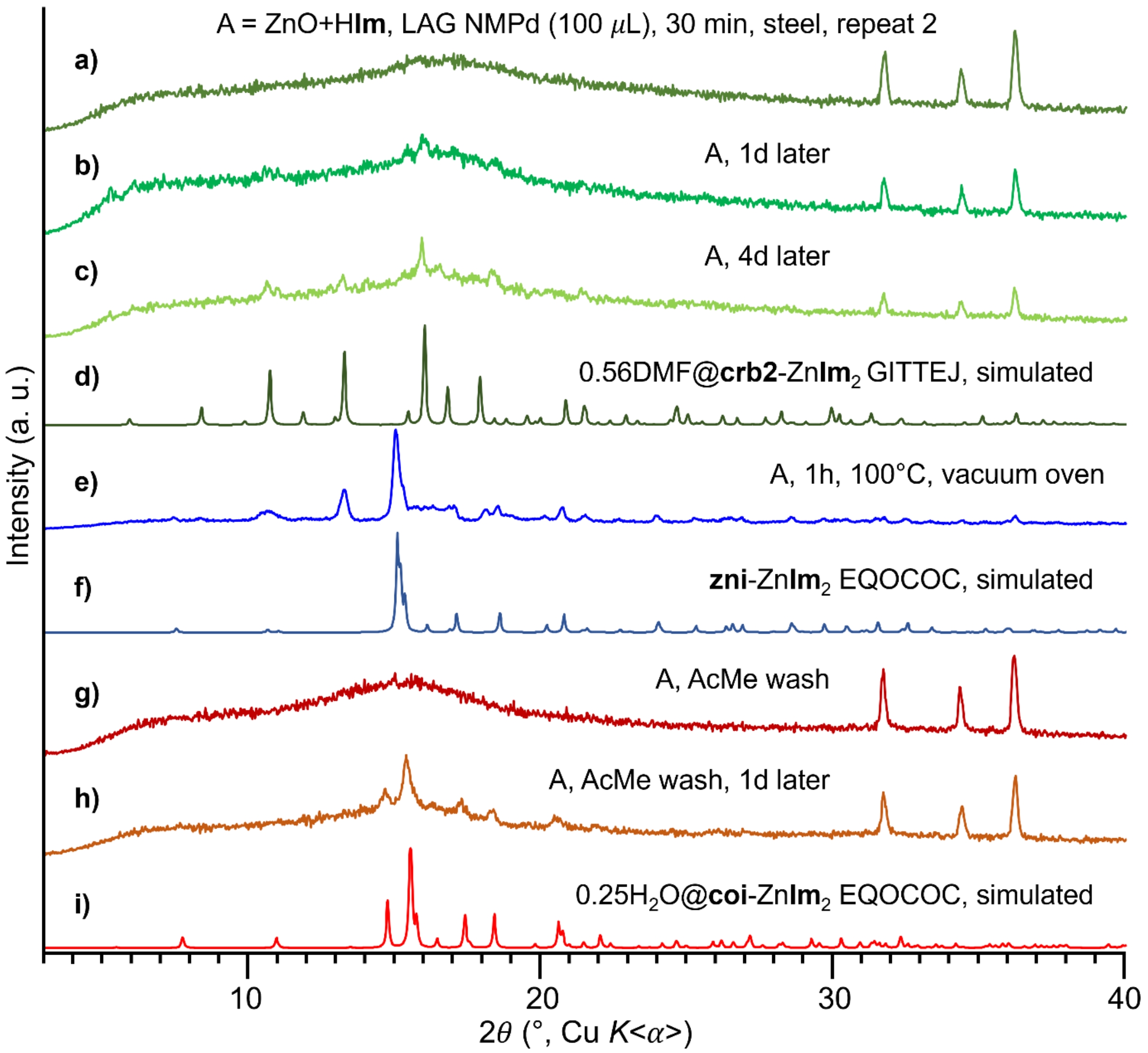

Figure S36. PXRD patterns of a) A= the product of milling ZnO and H**Im** with NMP in a steel jar for 30 min, second repeated experiment; b) A after 1 day; c) A after 4 days; e) A after 1h heating in a vacuum oven at 100°C; g) A washed with acetone; h) product of g, 1 day later. Simulated PXRD pattern of d) 0.56DMF@**crb2**-Zn**Im**$_2$ (CSD code GITTEJ); f) **zni**-Zn**Im**$_2$ (CSD code IMIDZB02) and i) 0.25H2O@**coi**-Zn**Im**$_2$ (CSD code EQOCOC). Black stars denote peaks of leftover ZnO reagent.

2.1.34. N-Methylpyrrolidine (NMPl)

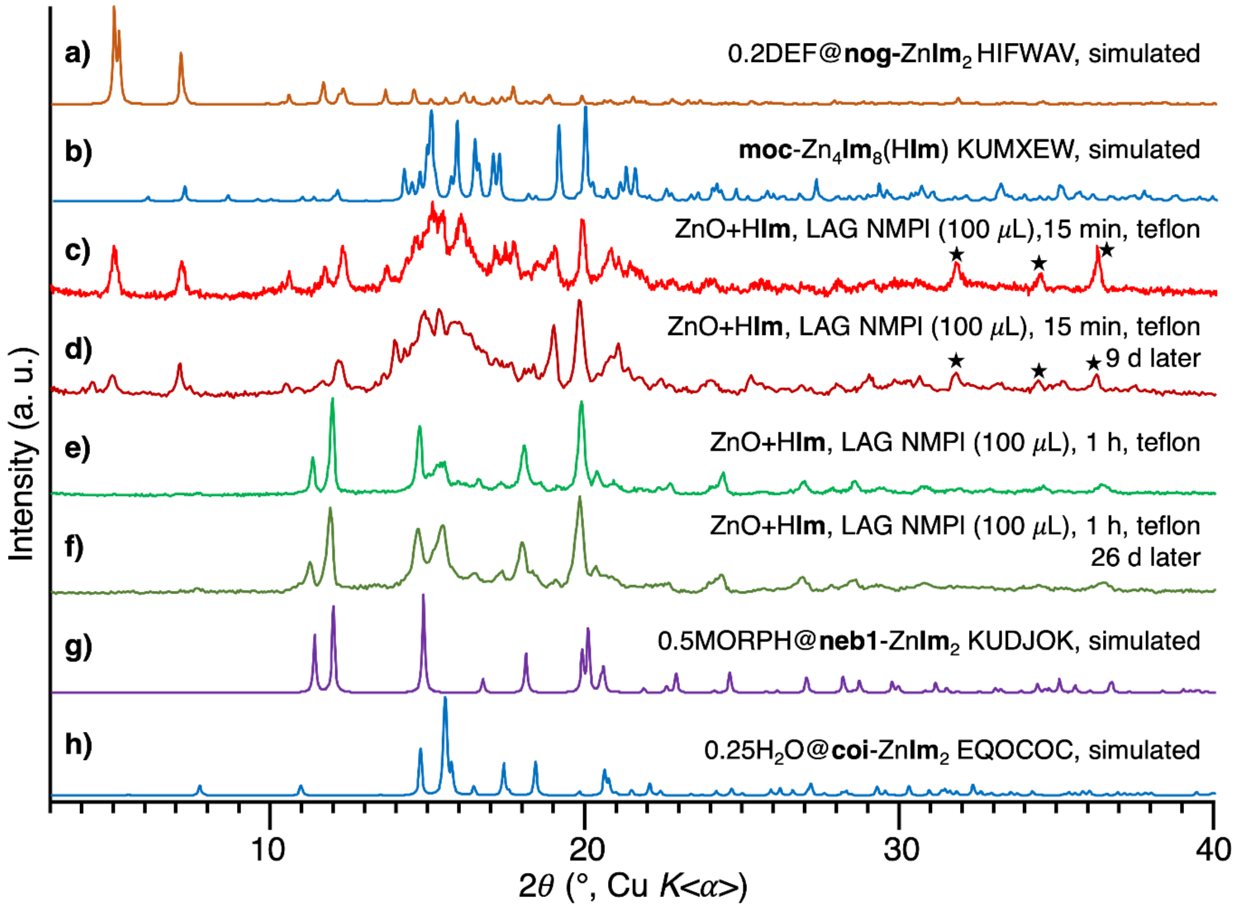

Figure S37. Simulated PXRD patterns of a) 0.2DEF@**nog**-Zn**Im**$_2$ (CSD code HIFWAV) b) **moc**-Zn$_4$**Im**$_8$H**Im** (CSD code KUMXEW), g) 0.5MORPH@**neb1**-Zn**Im**$_2$ (CSD code KUDJOK), and h) 0.25H$_2$O@**coi**-Zn**Im**$_2$ (CSD code EQOCOC). PXRD patterns of the products of milling ZnO and H**Im** with NMPl c) in a Teflon™ jar for 15 min; d) product of a after 9 days; e) in a Teflon™ jar for 60 min, and f) product of c after 26 days. Black stars denote peaks of leftover ZnO reagent.

2.1.35. N-methylpiperidine (NMPP)

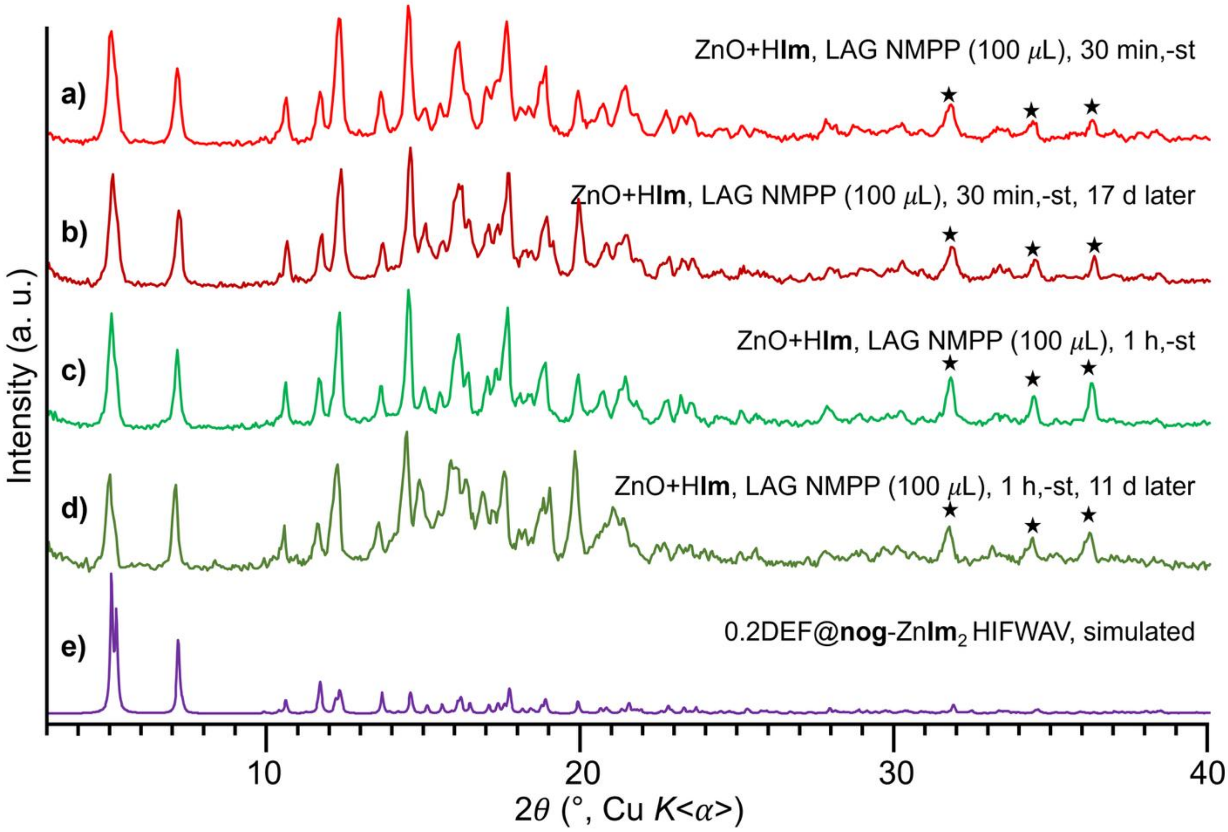

Figure S38. PXRD patterns of the products of milling ZnO and H**Im** with NMPP a) in a steel jar for 30 min; b) product of a after 17 days; c) in a steel jar for 60 min; d) product of c after 11 days. e) Simulated PXRD pattern of 0.2DEF@**nog**-Zn**Im**$_2$ (CSD code HIFWAV). Black stars denote peaks of leftover ZnO reagent.

2.1.36. Propan-1-ol (nPrOH)

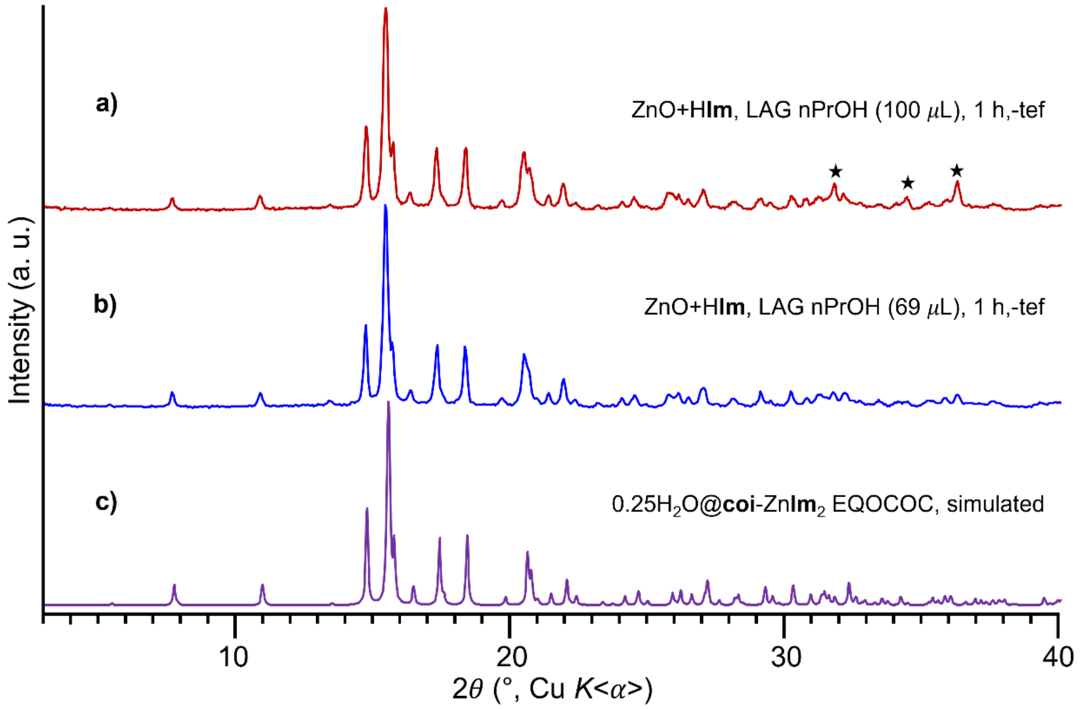

Figure S39. PXRD patterns of the products of milling ZnO and H**Im** with nPrOH a) in a Teflon™ jar for 60 min; b) in a Teflon™ jar for 60 min, using an equimolar amount of nPrOH (69 µL). c) Simulated PXRD pattern of $0.25H_2O$@**coi**-Zn**Im**$_2$ (CSD code EQOCOC). Black stars denote peaks of leftover ZnO reagent.

2.1.37. Oxetane (OXT)

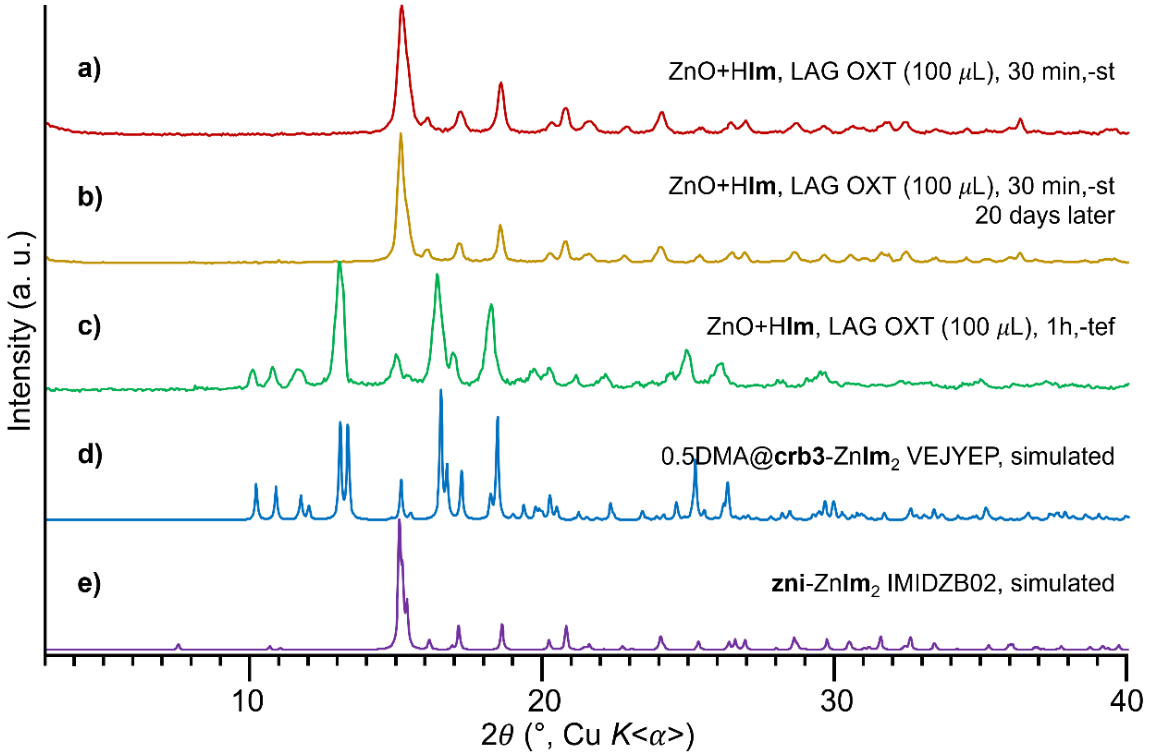

Figure S40. PXRD patterns of the products of milling ZnO and H**Im** with NMP a) in a steel jar for 30 min; b) product of a after 20 days; c) in a Teflon™ jar for 60 min. Simulated PXRD patterns of d) 0.5DMA@**crb3**-Zn**Im**$_2$ (CSD code VEJYEP) and e) **zni**-Zn**Im**$_2$ IMIDZB02.

2.1.38. Toluene (PhMe)

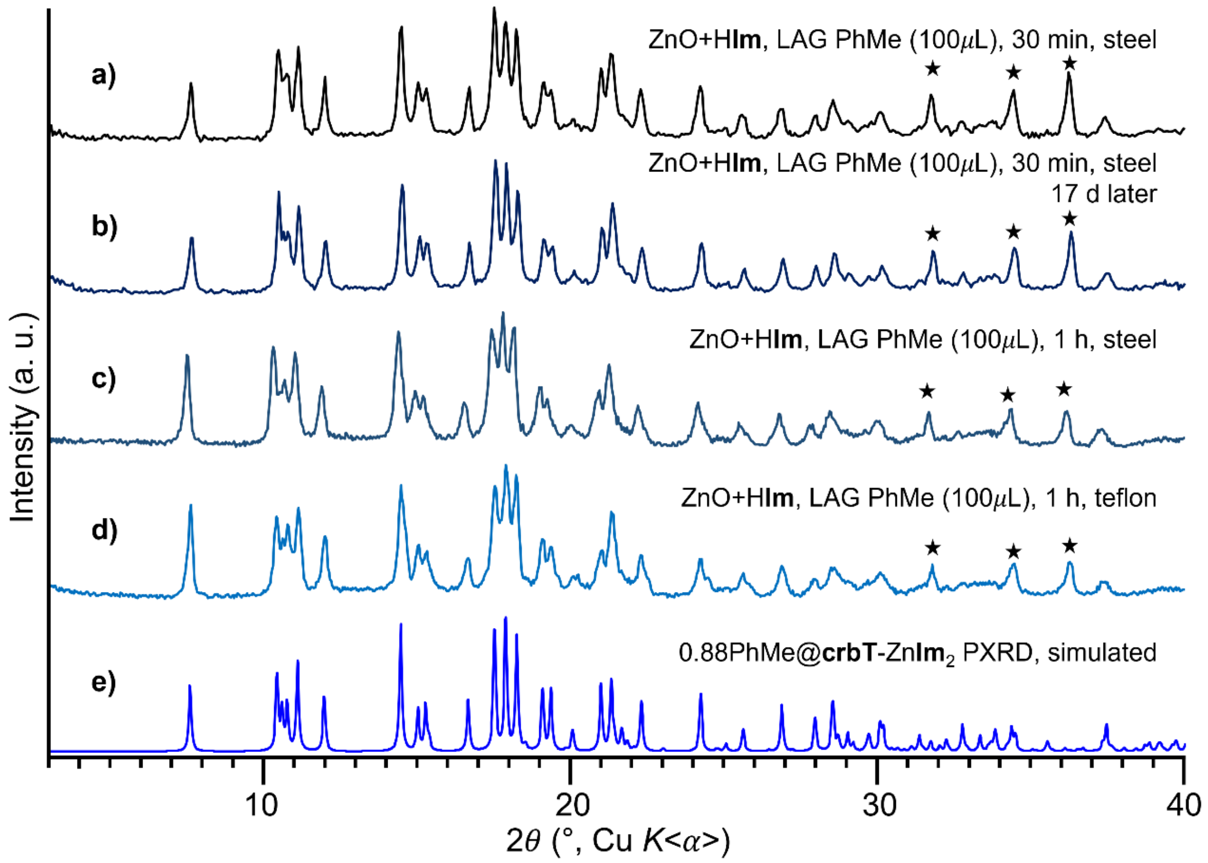

Figure S41. PXRD patterns of the products of milling ZnO and H**Im** with PhMe a) in a steel jar for 30 min; b) in a steel jar for 30 min, 17 days later; c) in a steel jar for 60 min; d) in a Teflon™ jar for 60 min. Simulated PXRD pattern of e) 0.88PhMe@**crbT**-Zn**Im**$_2$ (solved from PXRD data). Black stars denote peaks of leftover ZnO reagent.

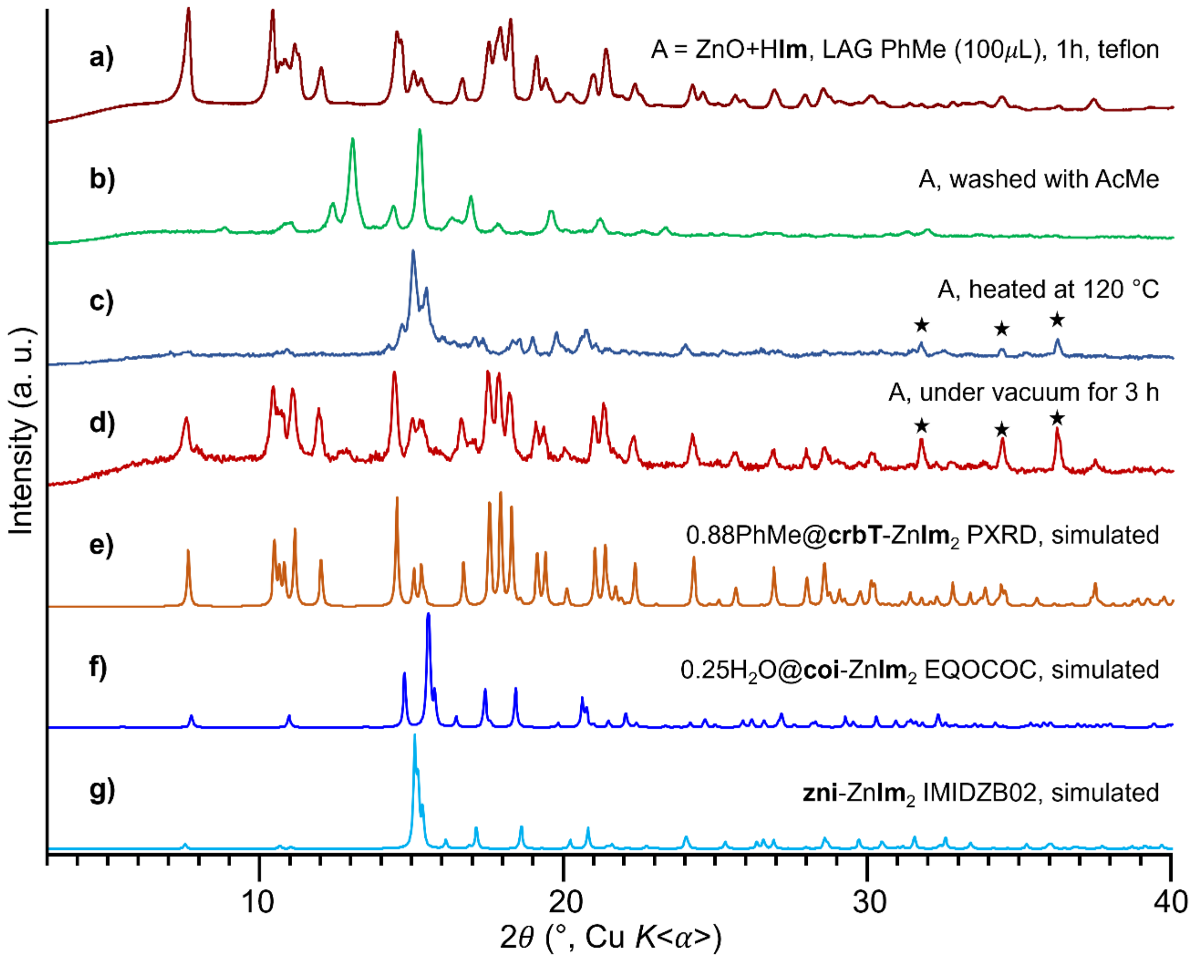

Figure S42. PXRD patterns of a) the product of milling ZnO and H**Im** with PhMe in a Teflon™ jar for 1 h; b) product of a, washed with acetone; c) product of a, heated at 120 °C for 3 h; d) product of a, under vacuum for 3 h. Simulated PXRD patterns of e) 0.88PhMe@**crbT**-Zn**Im**$_2$ (solved from PXRD data), f) 0.25H_2O@**coi**-Zn**Im**$_2$ (CSD code EQOCOC), and g) **zni**-Zn**Im**$_2$ (CSD code IMIDZB02). Black stars denote peaks of leftover ZnO reagent.

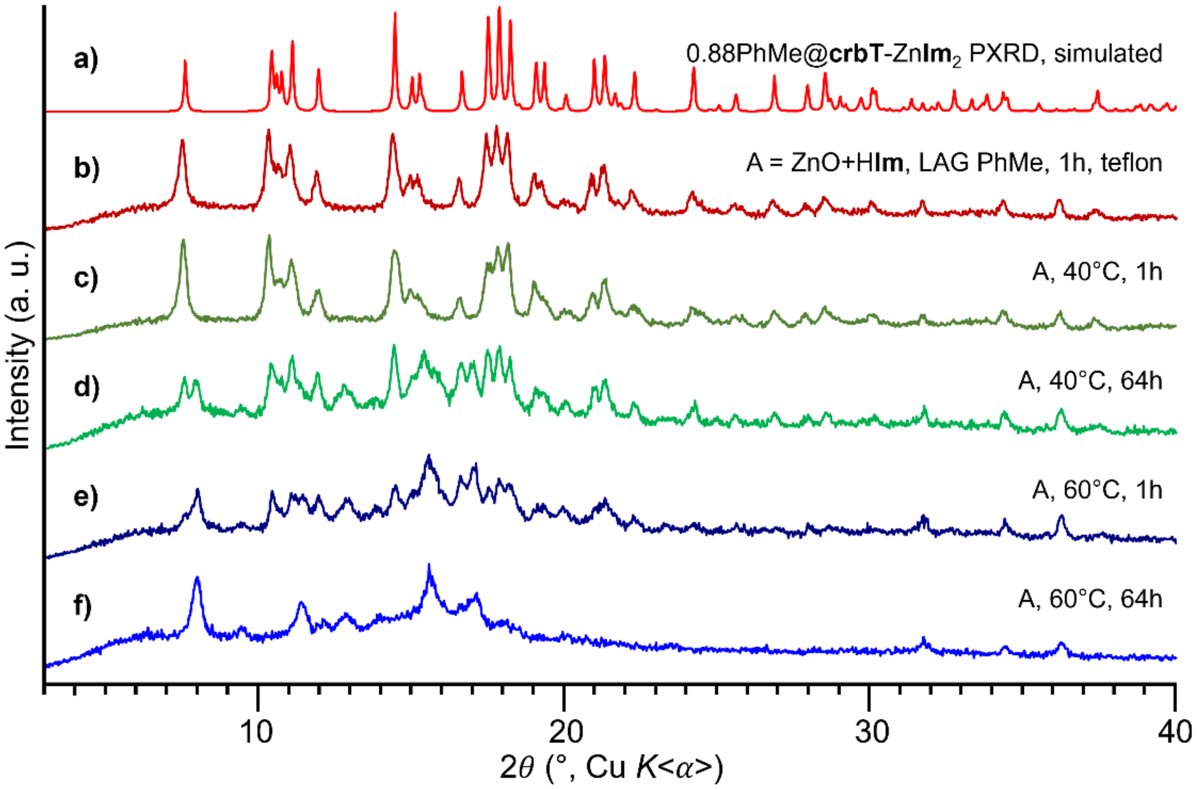

Figure S43. a) Simulated PXRD pattern of 0.88PhMe@**crbT**-Zn**Im**$_2$ (solved from PXRD data). PXRD patterns of b) A = the product of milling ZnO and H**Im** with PhMe in a Teflon™ jar for 1 h; c) A, heated at 40 °C for 1 h; d) A, heated at 40 °C for 64 h; e) A, heated at 60 °C for 1 h; f) A, heated at 60 °C for 64 h.

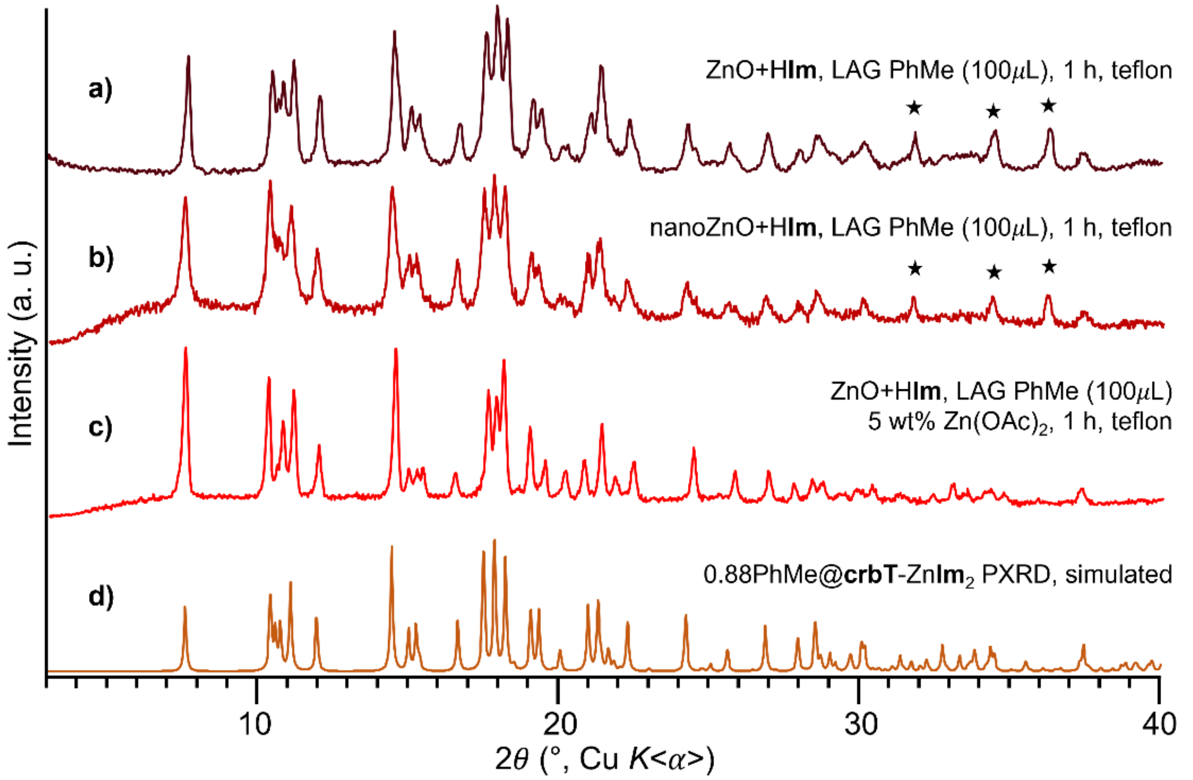

Figure S44. PXRD patterns of a) the product of milling ZnO and H**Im** with PhMe in a Teflon™ jar for 1 h; b) the product of milling nanoZnO and H**Im** with PhMe in a Teflon™ jar for 1 h; c) the product of milling ZnO and H**Im** with PhMe in a Teflon™ jar for 1 h, with the addition of 5 wt% of zinc acetate (compared to ZnO). Simulated PXRD pattern of d) 0.88PhMe@**crbT**-Zn**Im**$_2$ (solved from PXRD data). Black stars denote peaks of leftover ZnO reagent.

2.1.39. Anisole (PhOMe)

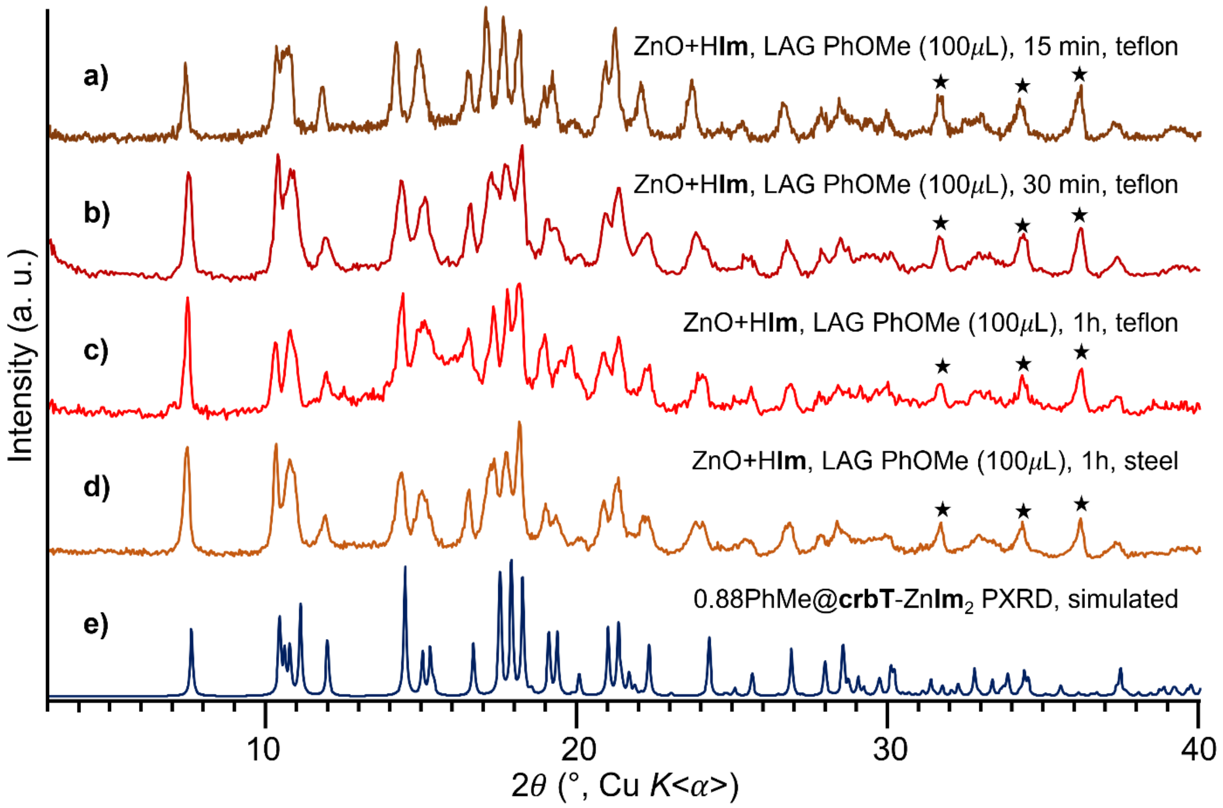

Figure S45. PXRD patterns of the product of milling ZnO and H**Im** with PhOMe a) in a Teflon™ jar for 15 min; b) in a Teflon™ jar for 30 min; c) in a Teflon™ jar for 1 h; d) in a steel jar for 1 h. e) Simulated PXRD pattern of 0.88PhMe@**crbT**-Zn**Im**$_2$ (solved from PXRD data). Black stars denote peaks of leftover ZnO reagent.

2.1.40. Piperidine (PP)

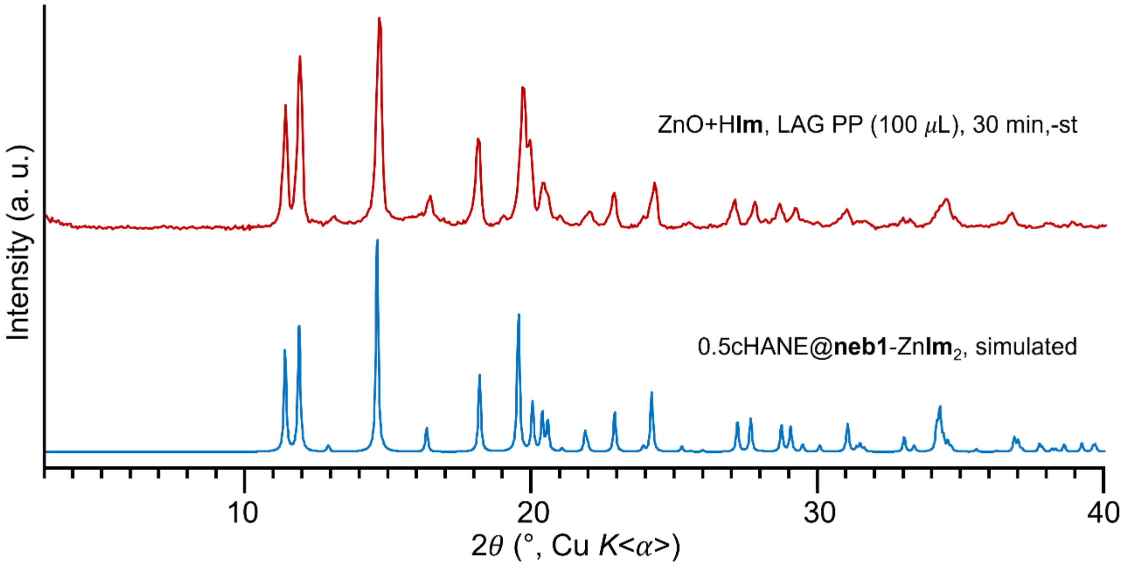

Figure S46. PXRD patterns of the products of milling ZnO and H**Im** with PP in a steel jar for 30 min (red, top) and the simulated PXRD pattern of 0.5cHANE@**neb1**-Zn**Im**$_2$ (blue, bottom).

2.1.41. Piperazine (PPZ)

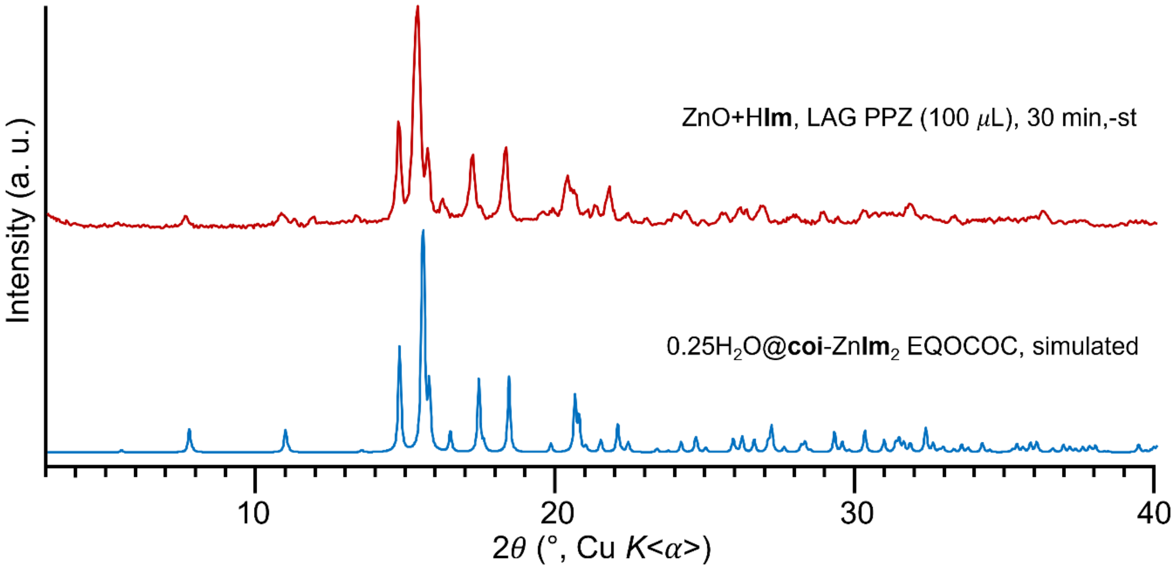

Figure S47. PXRD patterns of the products of milling ZnO and H**Im** with PPZ in a steel jar for 30 min (red, top) and the simulated PXRD pattern of 0.25H_2O@**coi**-Zn**Im**$_2$ EQOCOC (blue, bottom).

2.1.42. Pyridine (PYR)

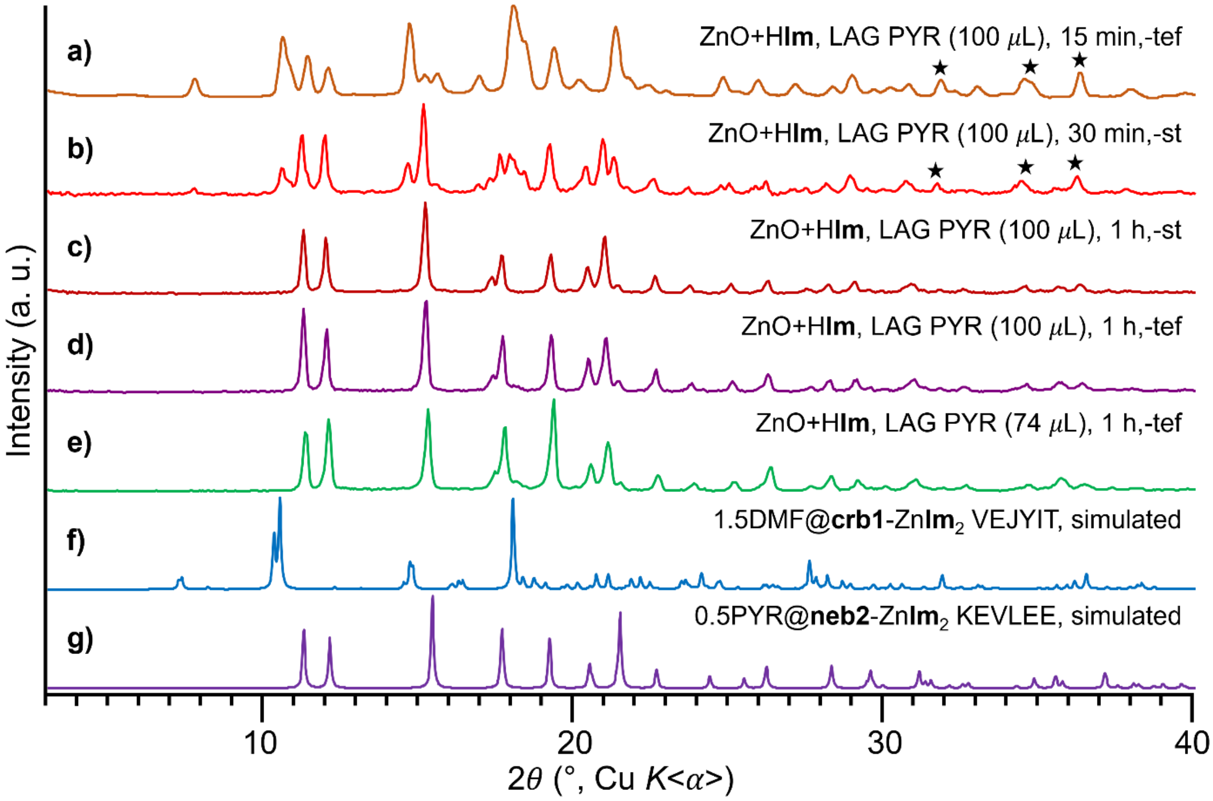

Figure S48. PXRD patterns of the products of milling ZnO and H**Im** with PYR a) in a Teflon™ jar for 15 min; b) in a steel jar for 30 min; c) in a steel jar for 60 min; d) in a Teflon™ jar for 60 min; e) in a Teflon™ jar for 60 min, using an equimolar amount of PYR (74 µL). Simulated PXRD patterns of f) 1.5DMF@**crb1**-Zn**Im**$_2$ (CSD code VEJYIT) and g) 0.5PYR@**neb2**-Zn**Im**$_2$ (CSD code KEVLEE). Black stars denote peaks of leftover ZnO reagent.

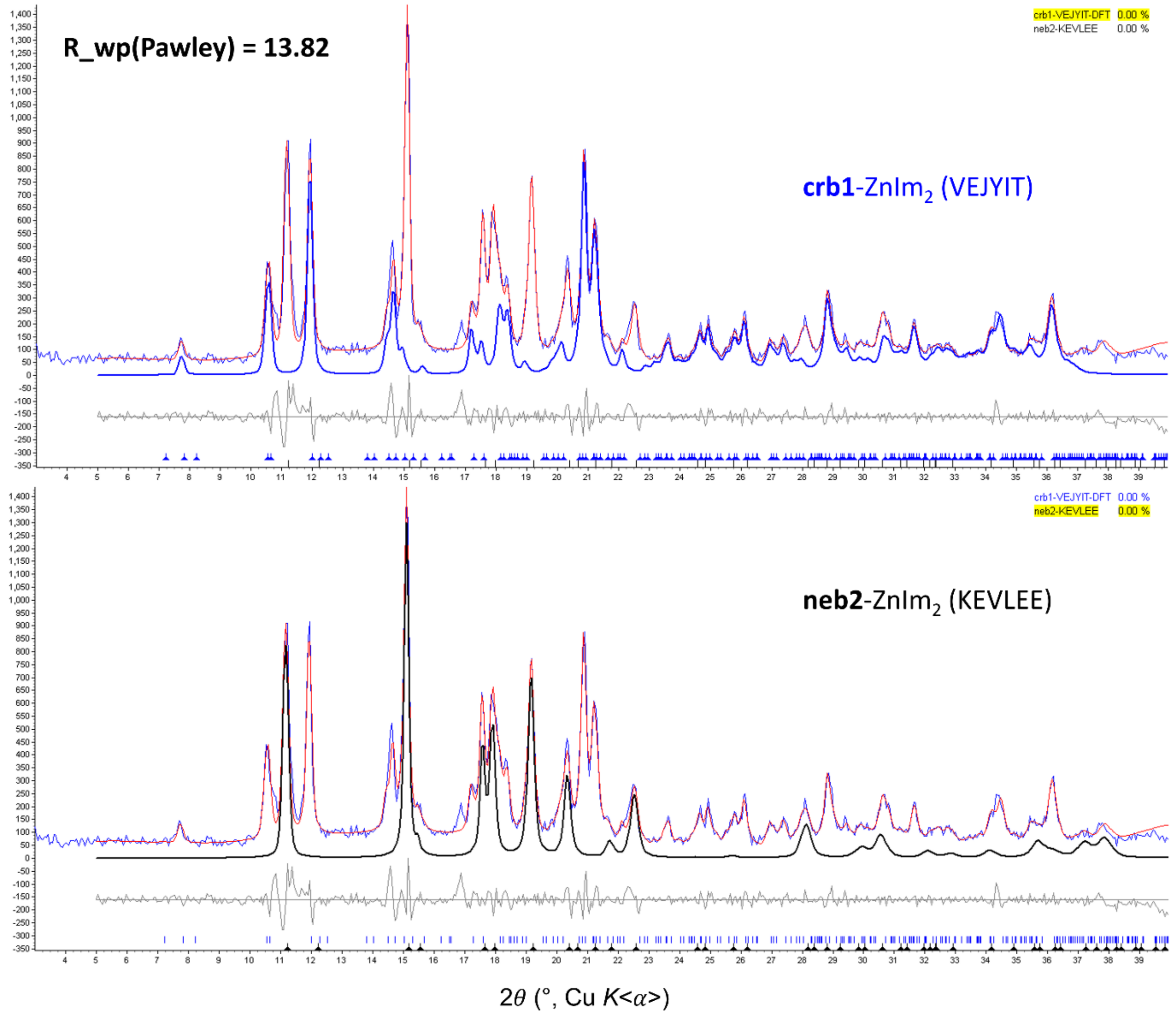

Figure S49. Pawley fitting of **crb1**-Zn**Im**$_2$ (CSD code GITTEJ, DFT optimized) and 0.5PYR@**neb2**-Zn**Im**$_2$ (CSD code KEVLEE) to the experimental PXRD pattern obtained by milling ZnO and H**Im** with PYR for 30 min in a steel jar. The experimental pattern is in light blue, the Pawley fit in red, and the difference curve in grey. Top panel displays the **crb1** contribution (blue line), and the bottom panel shows the **neb2** contribution (black line) to the overall fit.

2.1.43. *Tetrahydrofuran (THF)*

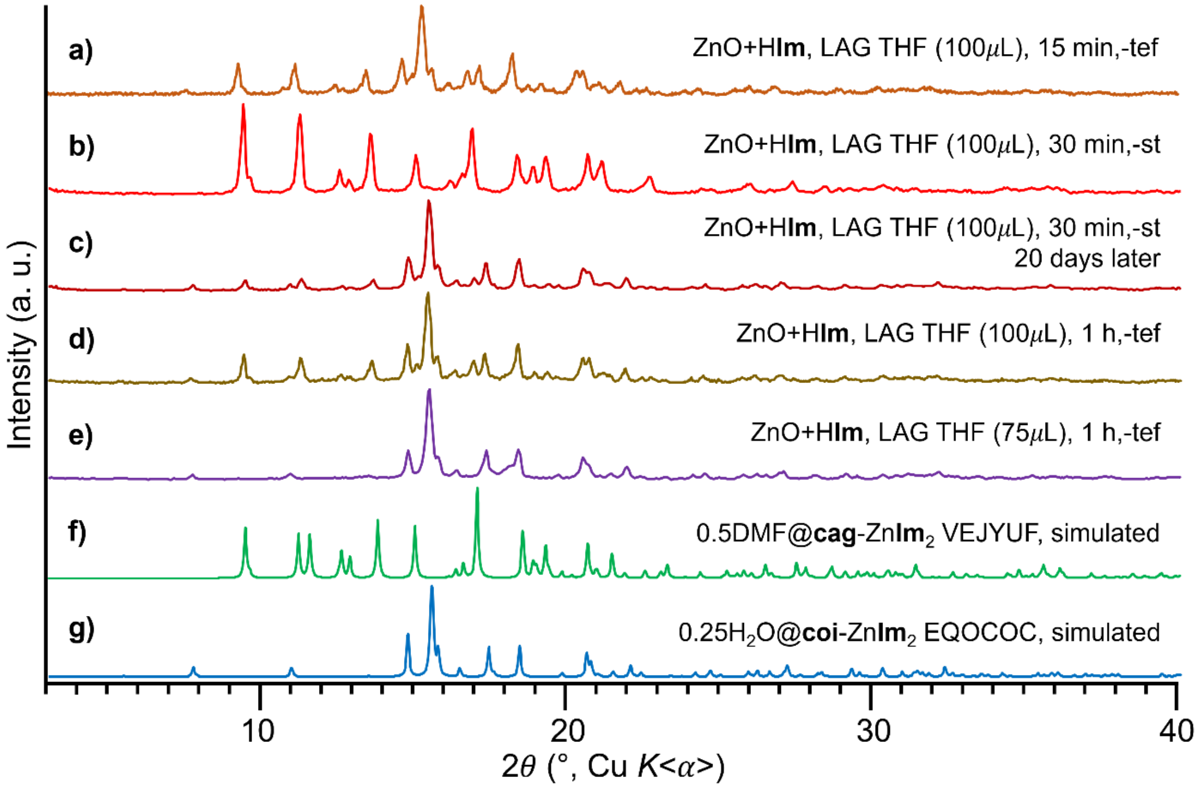

Figure S50. PXRD patterns of the products of milling ZnO and H**Im** with THF a) in a Teflon™ jar for 15 min; b) in a steel jar for 30 min; c) product of b, 20 days later; d) in a Teflon™ jar for 60 min; e) in a Teflon™ jar for 60 min, using an equimolar amount of THF (75 µL). Simulated PXRD patterns of f) 0.5DMF@**cag**-Zn**Im**$_2$ (CSD code VEJYUF) and g) 0.25H$_2$O@**coi**-Zn**Im**$_2$ (CSD code EQOCOC).

2.1.44. *Tetrahydropyran (THP)*

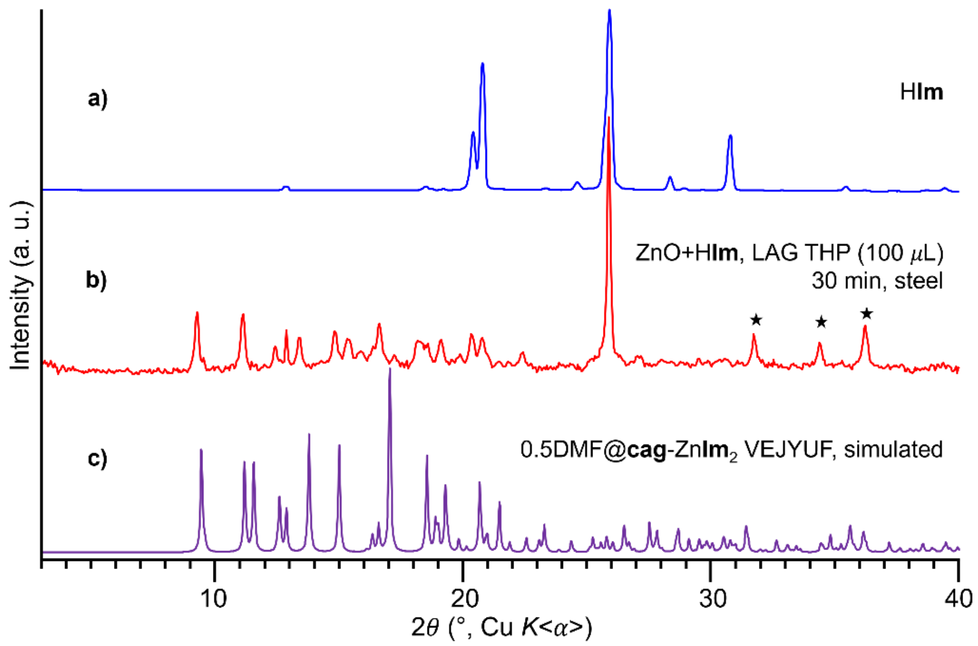

Figure S51. PXRD patterns of a) imidazole; b) the product of milling ZnO and H**Im** with THP in a steel jar for 30 min. c) Simulated PXRD pattern of 0.5DMF@**cag**-Zn**Im**$_2$ (CSD code VEJYUF). Black stars denote peaks of leftover ZnO reagent. PXRD pattern b) is shown on a square root *y* axis due to the very large imidazole impurity.

2.1.45. Thiophene (TPH)

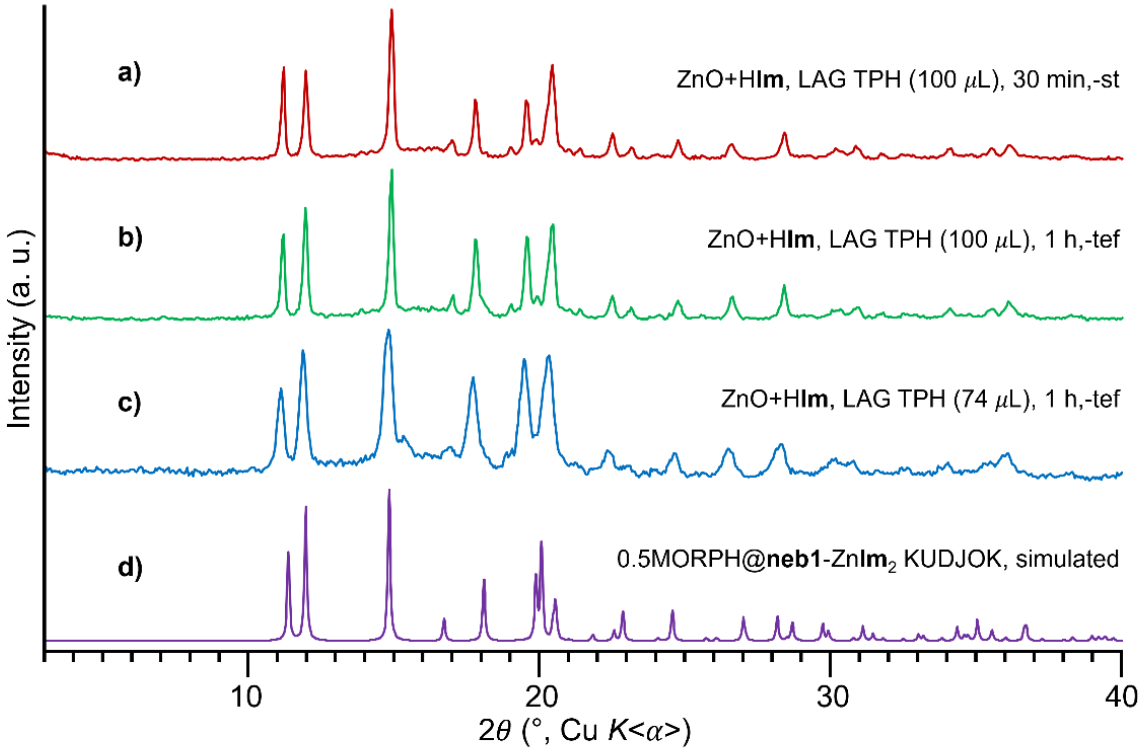

Figure S52. PXRD patterns of the products of milling ZnO and H**Im** with TPH a) in a steel jar for 30 min; b) in a Teflon™ jar for 60 min; c) in a Teflon™ jar for 60 min, using an equimolar amount of TPH (74 µL). d) Simulated PXRD pattern of 0.5MORPH@**neb1**-Zn**Im**$_2$ (CSD code KUDJOK).

2.1.46. Valerolactone (VLAC)

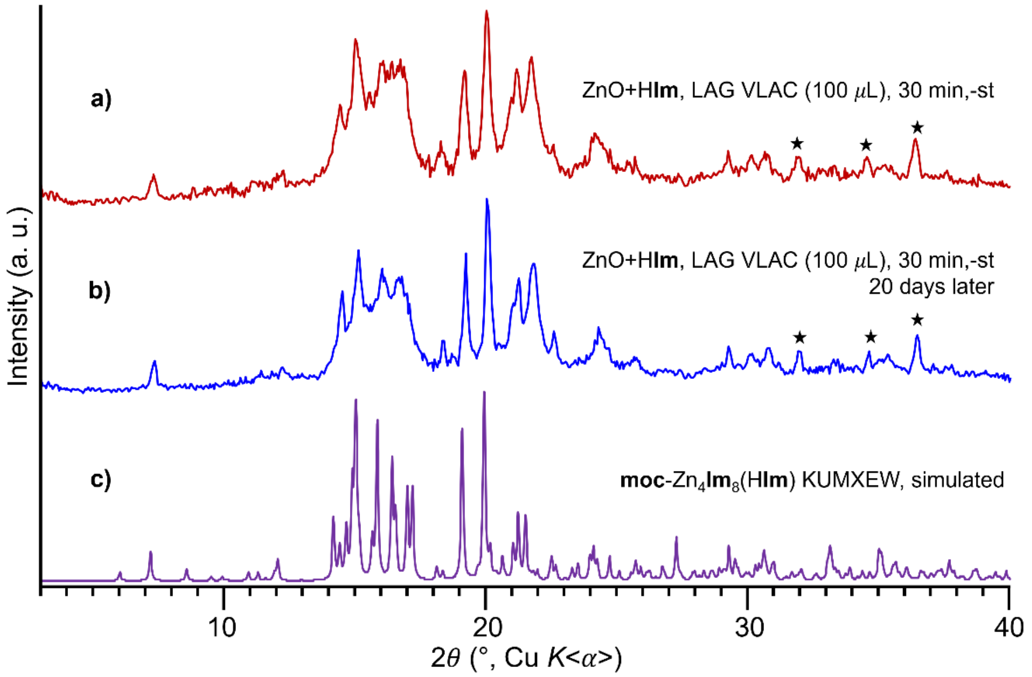

Figure S53. PXRD patterns of the products of milling ZnO and H**Im** with VLAC a) in a steel jar for 30 min; b) Product of a after 20 days. c) Simulated PXRD pattern of **moc**-Zn$_4$**Im**$_8$H**Im** (CSD code KUMXEW). Black stars denote peaks of leftover ZnO reagent.

2.1.47. Summary of PXRD experimental results and previous literature

Table S2. Summary of previous synthesis of the prepared topological forms.

Topologic form	CSD code	Previous work
**moc**	KUMXEW[20]	1-ethyl-3-methylimidazolium bis[(trifluoro-methyl)sulfonyl]imide (ionic liquid), 130 °C, 2d[20]<br>dry, 100°C, 2d[26]
**zni**	IMIDZB02[27]	DMF/butanol, 150 °C, 5d[27]<br>dry, 160°C, 2d[26]<br>from coi, heating above 360 °C[28]<br>DEF, propylamine, 10 °C, 120 h[29]
**coi**	IMIDZB07[27]<br>IMIDZB09[20]<br>ZAVBIL[30]<br>EQOCOC[19]	1-ethyl-3-methylimidazolium bis[(trifluoro-methyl)sulfonyl]imide (ionic liquid), 150 °C, 2d[20]<br>From neb2, in EtOH/PYR, 140°C[28]<br>From zni, 0.543(5)−0.847(5) GPa pressure range[27]<br>DMSO, propylimidazole, 100°C, 3d[30]<br>pyridine/ethanol, 140°C, 24 h[19]
**crb1**	VEJYIT[10]<br>(ZIF-2)	DMF, 85 °C, 3d[10,31 †]
**crb2**	GITTEJ[31]<br>(ZIF-64)	DMF, 100°C, 3d[31 †]
**crb3**	VEJYEP[10]<br>(ZIF-1)	DMF, 85 °C, 1d[10], DMA found in crystal structure
**crbT**	new	/
**crbA**	new	/
**cag**	VEJYUF01[32]	DMF/propylamine, RT, several days or weeks[32]
**neb1**	KUDJOK[33]	EtOH/MORPH, 140 °C, 4h[33]
**neb2**	KEVLEE[28]	EtOH/PYR, RT, 1d[28]
**nog**	HIFWAV[32]	DEF/propylamine/pyrazine, RT, 40 min to several days[32]
**10mr**	GOQSIQ[34]	DBF, 50°C, 3d[34]

[†] found only as single crystals in high-throughput experiments

Table S3. Summary of mechanochemical conditions resulting in different topological forms, either as a single phase product (column 2), or as a mixture including other phases (column 3).

	clean (single phase)	in mixture
**moc**	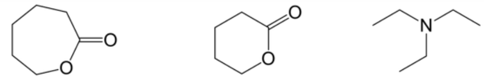	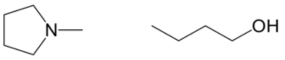
**zni**	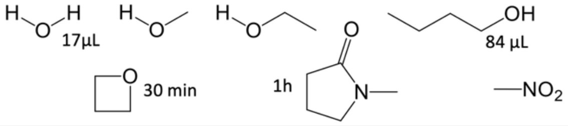 17μL; 84 μL; 30 min; 1h	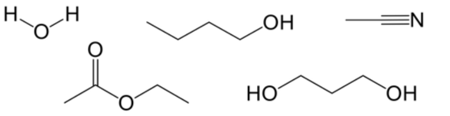
**coi**	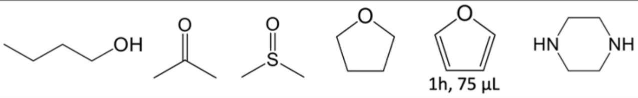 1h, 75 μL	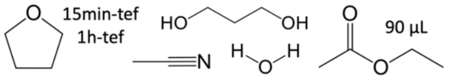 15min-tef 1h-tef; 90 μL
**crb1**		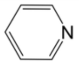 15 min-tef 30 min-tef
**crb2**		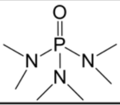
**crb3**	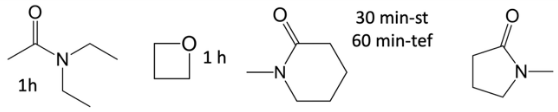 1h; 1 h; 30 min-st 60 min-tef; 15 min 30 min 89 μL	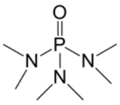
**crbT**	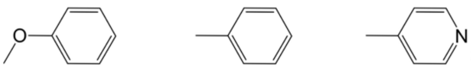 89 μL	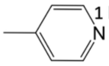 1 h
**crbA**	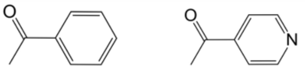	
**cag**	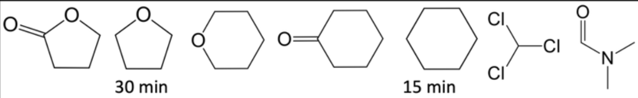 30 min; 15 min	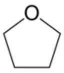 15 min-tef 90 min-tef
**neb1**	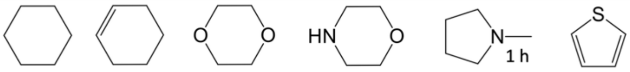 1 h	
**neb2**	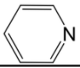 1h-st	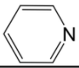 15 min-tef 30 min-tef 30 min-st
**nog**	1h+12d 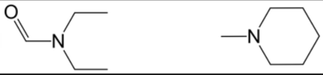	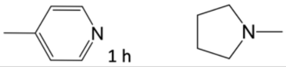 1 h
**10mr**	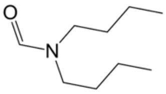	
**afi**	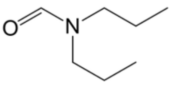	
**amorph**	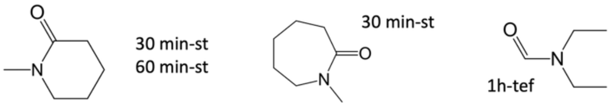 30 min-st 60 min-st; 30 min-st; 1h-tef	

Table S4. Summary of topological outcomes of mechanochemical LAG reactions for each liquid. Numbers 15, 30, 60 or 90 indicate minutes of milling, the tag -st indicates reaction in steel, the tag -tef indicates reactions in Teflon™, the tag -*xx*d indicates the result after *xx* days. Unless otherwise noted (marked with -xxµL), all reactions used 100 µL liquid. μL

Liquid	Molecular diagram	Mechanochemical result
**AcMe**	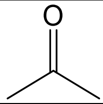	**coi** (30-st, 60-tef)
**AcPhe**	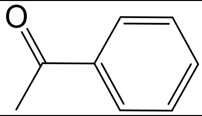	**crbA** (30-tef, 60-tef, 60-tef-wash, 60-tef-wash-vac), **crbA**+**crb3**(60-tef-wash-80C), **crb3** (60-tef-wash-105C) **unknown1** (60-tef-wash-sorption150C, 60-tef-wash-150C)
**4AcPyr**	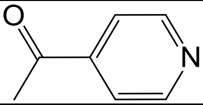	**crbA** (60-tef)
**BLAC**	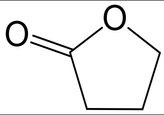	**cag** (15-tef, 30-st, 30-tef, 60-tef, 60-tef-61µL)
**cHANE**	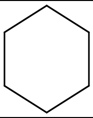	**cag** (15-tef), **neb1** (30-st, 30-st-14d, 30-tef, 60-tef, 90-tef)
**$CHCl_3$**	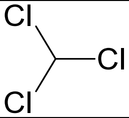	**cag** (60-tef)
**cHENE**	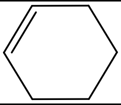	**neb1** (15-tef, 60-tef, 60-tef-11d, 60-st, 60-tef-96µL)
**cHONE**	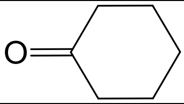	**cag** (15-tef, 30-st, 30-st-14d, 60-tef)
**CLAC**	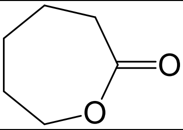	**moc** (30-st, 30-st-20d)
**DBF**	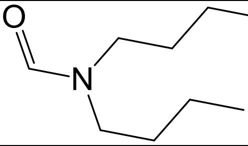	**10-mr** (60-tef-200µL, 60-tef-167µL), **10mr**+**moc** (15-tef-200µL, 60-tef-100µL), **moc** (60-tef-200µL-repeat2)
**DEF**	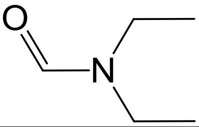	**amorphous** (60-tef), **nog** (60-tef-12d)
**DPF**	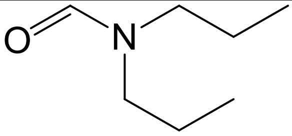	**afi** (60-tef-300)
**DIOX**	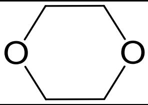	**neb1** (15-tef, 30-tef, 30-st, 60-tef), **neb1**+**moc** (30-st-20d)
**DMA**	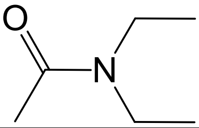	**crb3** (60-tef), **crb3**+**moc** (30-st)

**DMF**	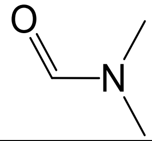	**cag** (60-tef)
**DMSO**	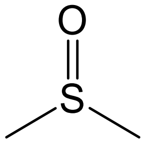	**coi** (60-tef, 50-tef-65μL)
**Et_3N**	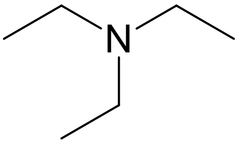	**moc** (60-tef, 60-tef-128μL)
**EtGly**	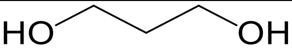	**coi**+**zni** (60-tef)
**EtOAc**	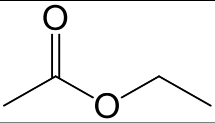	**coi**+**zni+unknown2** (60-tef, 60-tef-90μL)
**EtOH**	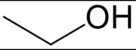	**zni** (60-tef, 60-tef-54μL)
**FUR**	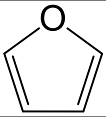	**coi** (30-st, 60-tef)
**H_2O**	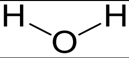	**coi**+**zni** (60-tef, 60-tef-17μL)
**HMPA**	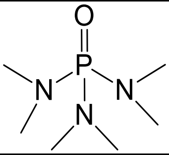	**crb2**+**crb3** (30-st)
**MeCN**	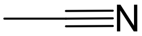	**coi**+**zni** (30-st, 60-tef, 60-tef-42μL)
**$MeNO_2$**	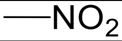	**zni** (60-tef)
**MeOH**	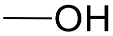	**zni** (60-tef, 60-tef-37μL)
**4-MePyr**	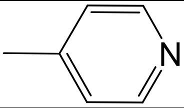	**crbT** (60-tef-89μL), **crbT**+**nog** (60-tef)
**MORPH**	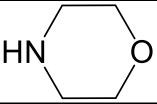	**neb1** (30-tef, 60-tef, 90-tef, 60-tef-79μL, acetone wash)
**nBuOH**	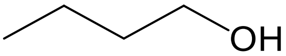	**zni** (60-tef-84μL), **zni**+**moc** (60-tef)
**NMC**	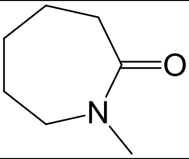	**amorphous** (30-st, 30-st-repeat, 30-st-repeat-8d), **crb1** (30-st-20d), **zni+unknown3** (30-st-1year)
**NMP**	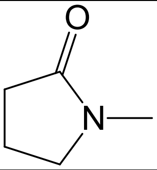	**crb3** (15-tef, 15-tef-9d, 30-st, 30-st-20d, 60-tef-89μL, 60-tef-89μL-repeat), **zni** (60-st, 60-st-29d)

**NMPd**	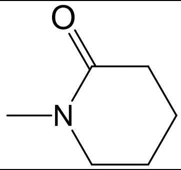	**amorphous** (30-st, 30-st-20d, 60-st, 60-st-11d, 30-st-repeat2), **crb3** (30-st-repeat, 30-st-repeat-8d, 60-tef)
**NMPl**	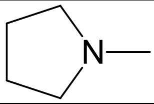	**nog**+**moc** (15-tef, 15-tef-9d), **neb1+coi** (60-tef, 60-tef-26d)
**NMPP**	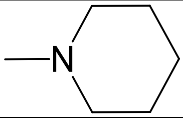	**nog** (30-st, 30-st-17d, 60-st, 60-st-11d)
**nPrOH**	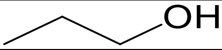	**coi** (60-tef, 60-tef-69µL)
**OXT**	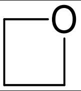	**zni** (30-st, 30-st-20d), **crb3** (60-tef)
**PhMe**	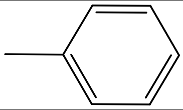	**crbT** (30-st, 30-st-17d, 60-st, 60-tef, 60-tef-vac), **zni**+**coi** (60-tef-120C), **unknown4** (60-tef-wash), **unknown5** (60-tef-60C)
**PhOMe**	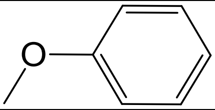	**crbT** (15-tef, 30-tef, 60-tef, 60-st)
**PP**	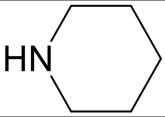	**neb1** (30-st)
**PPZ**	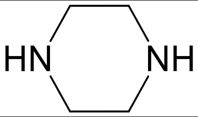	**coi** (30-st)
**PYR**	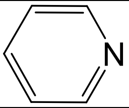	**crb1**+**neb2** (15-tef, 30-st), **neb2** (60-st, 60-tef, 60-tef-74)
**THF**	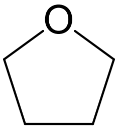	**cag** (30-st), **cag**+**coi** (15-tef, 60-tef, 30-st-20d), **coi** (60-tef-75)
**THP**	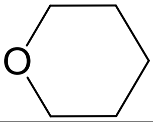	**cag+reagents** (30-st)
**TPH**	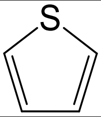	**neb1** (30-st, 60-tef, 60-tef-74µL)
**VLAC**	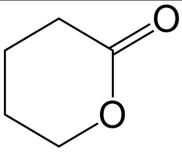	**moc** (30-st, 30-st-20d)

Scheme 1. Flowchart displaying the topologies and corresponding structure directing liquids that belong to case 1 – topological outcome depends only on the liquid used.

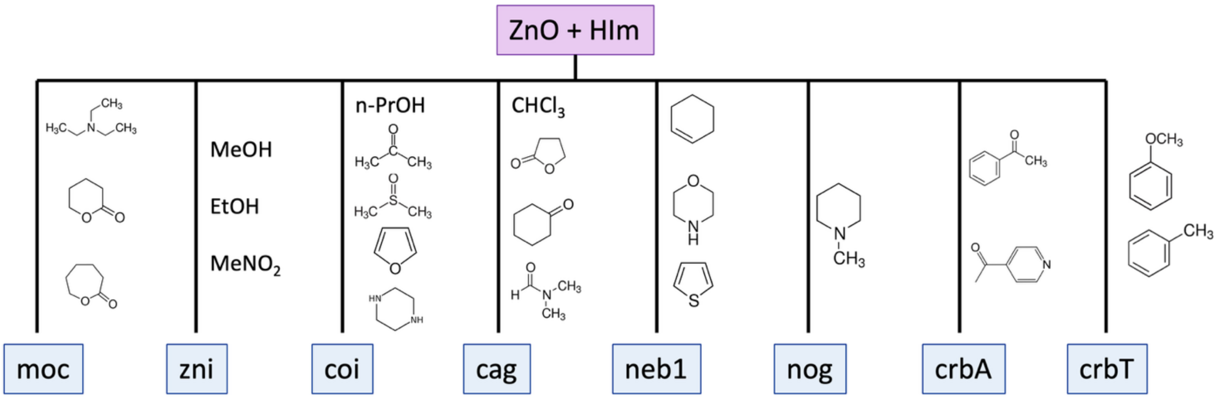

Scheme 2. Flowchart displaying the changes in solid form outcome of mechanochemical syntheses depending on milling time, or sample aging time.

<table>
<tr><th></th><th>15 min</th><th>30 min</th><th>60 min</th><th>Standing at RT</th></tr>
<tr><td>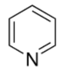</td><td colspan="2">crb1 + neb2</td><td>neb2</td><td></td></tr>
<tr><td>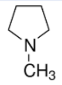</td><td colspan="2">nog + moc</td><td>neb1 + coi</td><td></td></tr>
<tr><td>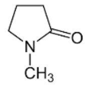</td><td colspan="2">crb3</td><td>zni</td><td></td></tr>
<tr><td>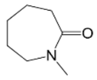</td><td></td><td>amorphous</td><td></td><td>crb3</td></tr>
<tr><td>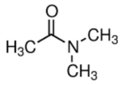</td><td></td><td>crb3 + moc</td><td>crb3</td><td></td></tr>
<tr><td>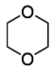</td><td colspan="3">neb1</td><td>neb1 + moc</td></tr>
<tr><td>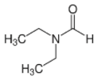</td><td></td><td></td><td>amorphous</td><td>nog</td></tr>
<tr><td>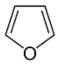</td><td colspan="2">cag</td><td>cag + coi</td><td></td></tr>
<tr><td>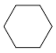</td><td>cag</td><td colspan="2">neb1</td><td></td></tr>
</table>

Scheme 3. Flowchart displaying the changes in solid form outcome of mechanochemical syntheses depending on amount of added LAG liquid.

	less added liquid	more added liquid
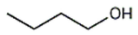	**zni** (84 μL)	**zni** + **moc** (100 μL)
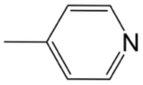	**crbT** (89 μL)	**crbT** + **nog** (100 μL)
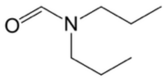	**moc** (100 μL)	**afi** (>200 μL)
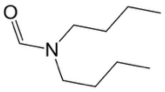	**moc** (100 μL)	**10mr** (>167 μL)

2.2. Crystal structures solved from Single Crystal X-Ray Diffraction (SCXRD)

The structures of the chloroform, tetrahydrofuran and N,N-diethylformamide solvates of the **cag** topology zinc imidazolate were solved from SCXRD experiments. All three compounds crystallize in the *Pbca* space group with slightly different unit cell parameters ($CHCl_3$: a=15.572(5) Å, b=14.634(5) Å, c=18.288(6) Å; THF: a=15.2629(7) Å, b=15.2037(7) Å, c=18.2768(9) Å; DEF: a=15.521(3) Å, b=14.903(3) Å, c=18.455(3) Å). This results in an overall unit cell volume expansion of 1.8% and 2.4% when replacing chloroform with tetrahydrofuran and N,N-diethylformamide, respectively, while keeping the zinc imidazolate framework nearly identical.

The unit cell of all three compounds consists of one guest molecule, two zinc cations and four imidazolate anions tetrahedrally coordinated to Zn (Figure S54 a, c, e). The THF structure contains well-resolved disorder in two of the four imidazolate anions, as well as positional disorder of the THF guest. The DEF structure contains well-resolved disorder of the DEF guest molecule, where the terminal methyl groups of both ethyl fragments are doubly disordered.

Each guest is contained in a zero dimensional pore, with no leftover solvent-accessible space in the structure (Figure S54 b, d, f).

Crystallographic parameters of all three structures can be found in Table S5.

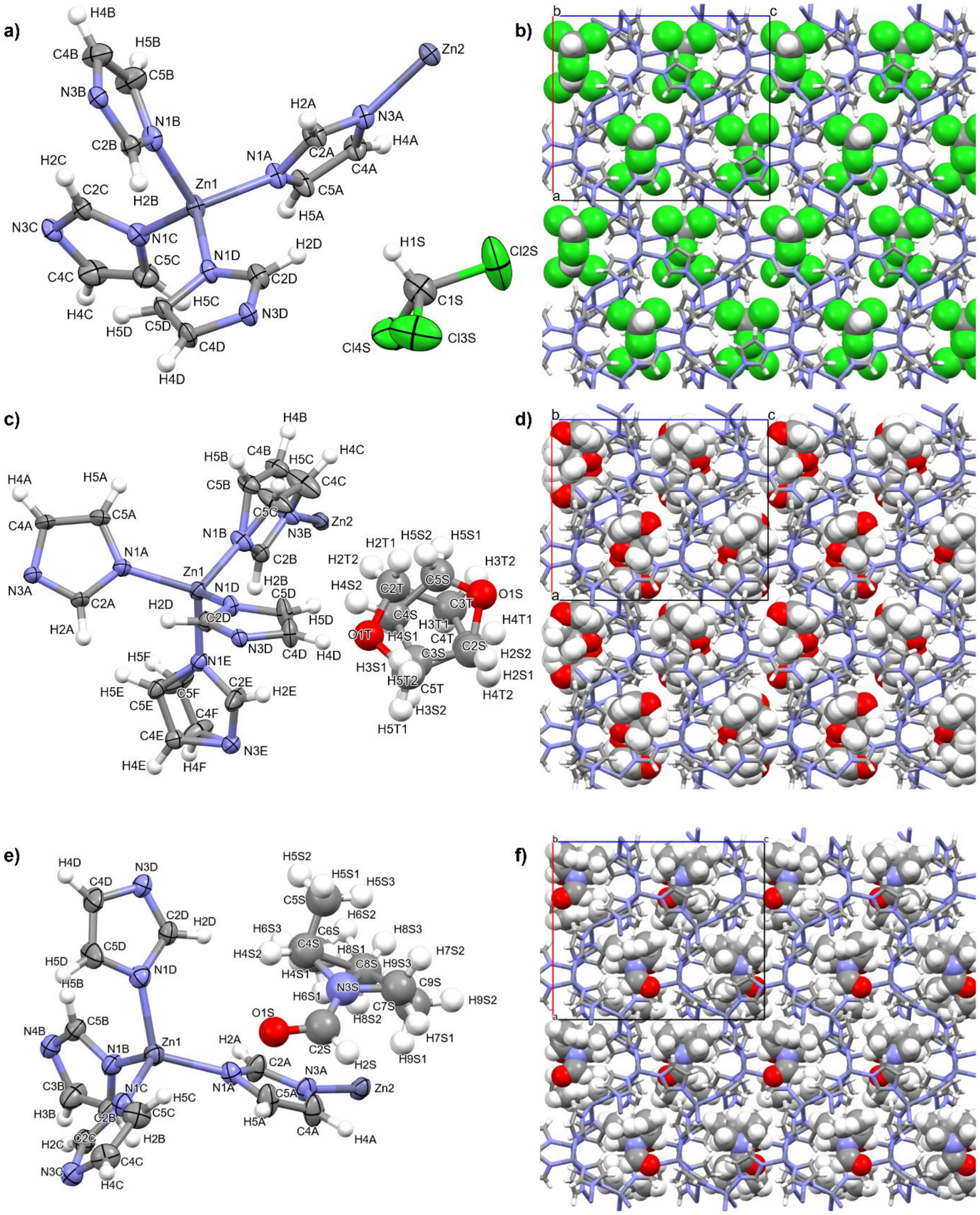

Figure S54. Crystal structures of $0.5CHCl_3$@**cag**-Zn**Im**$_2$ (a,b), 0.5THF@**cag**-Zn**Im**$_2$ (c,d) and 0.5DEF@**cag**-Zn**Im**$_2$ (e,f). (a, c, e) show the asymetric unit of the $CHCl_3$, THF and DEF solvates, respectively, with labels for all unique atoms. The THF and DEF molecules are shown in ball-and-stick representation due to heavy disorder. (b,d) show the packing of the three **cag** structures as viewed down the *b* crystallographic axis. The Zn**Im**$_2$ framework is shown in capped sticks (d = 0.2 Å), while the guests are shown in spacefill (r_{atom} = 0.75 r_{VdW}).

Table S5. Summary of crystallographic data for **cag**-Zn**Im**$_2$ solvate structures determined by diffraction on single crystals.

Crystal Parameters	0.5CHCl$_3$@cag-ZnIm$_2$	0.5THF@cag-ZnIm$_2$	0.5DEF@cag-ZnIm$_2$
**Empirical formula**	$C_{13} H_{13} Cl_3 N_8 Zn_2$	$C_{16} H_{20} N_8 O Zn_2$	$C_{16.04} H_{21.43} N_{8.68} O_{0.68} Zn_2$
**Formula weight**	518.40	471.14	477.47
**Temperature**	100(2) K	100(2) K	100(2) K
**Crystal system**	Orthorhombic	Orthorhombic	Orthorhombic
**Space group**	*Pbca*	*Pbca*	*Pbca*
***a*, Å**	15.572(5)	15.2629(7)	15.521(3)
***b*, Å**	14.634(5)	15.2037(7)	14.903(3)
***c*, Å**	18.288(6)	18.2768(9)	18.455(3)
***α*, °**	90	90	90
***β*, °**	90	90	90
***γ*, °**	90	90	90
**Volume, Å^3**	4167(2)	4241.2(3)	4268.9(13)
**Z**	8	8	8
**Density (calculated), g/cm^3**	1.652	1.476	1.486
**Absorption coefficient, mm^{-1}**	2.701	2.283	2.269
**F(000)**	2064	1920	1951
**Crystal size, mm x mm x mm**	0.210 x 0.190 x 0.090	0.260 x 0.250 x 0.160	0.118 x 0.114 x 0.111
**Theta range for data collection**	2.211 to 24.995°.	2.195 to 27.996°.	2.192 to 27.998°.
**Index ranges**	-18<=h<=18<br>-17<=k<=17<br>-21<=l<=21	-20<=h<=20<br>-20<=k<=20<br>-24<=l<=24	-20<=h<=20<br>-19<=k<=19<br>-24<=l<=24
**Reflections collected**	28962	56036	56627
**Independent reflections**	3674 [R(int) = 0.0677]	5115 [R(int) = 0.0379]	5161 [R(int) = 0.0543]
**Completeness to theta = 25.000°**	100.0 %	100.0 %	100.0 %
**Absorption correction**	Semi-empirical from equivalents	Semi-empirical from equivalents	Semi-empirical from equivalents
**Max. and min. transmission**	0.7457 and 0.5817	0.7462 and 0.6161	0.7462 and 0.6216
**Refinement method**	Full-matrix least-squares on F^2	Full-matrix least-squares on F^2	Full-matrix least-squares on F^2
**Data / restraints / parameters**	3674 / 0 / 235	5115 / 5 / 278	5161 / 18 / 274
**Goodness-of-fit on F^2**	1.062	1.198	1.094
**Final R indices [I>2sigma(I)]**	R1 = 0.0433<br>wR2 = 0.1091	R1 = 0.0406<br>wR2 = 0.0849	R1 = 0.0442<br>wR2 = 0.1212
**R indices (all data)**	R1 = 0.0623<br>wR2 = 0.1157	R1 = 0.0478<br>wR2 = 0.0866	R1 = 0.0589<br>wR2 = 0.1278
**Extinction coefficient**	n/a	n/a	n/a
**Largest diff. peak and hole, eÅ^{-3}**	0.982<br>-0.744	0.735<br>-0.694	0.814<br>-0.741
**CCDC number**	2381140	2381141	2423969

2.3. Crystal structures solved from Powder X-Ray Diffraction (PXRD)

*2.3.1. Crystal structure of 0.5cHANE@**neb1**-Zn**Im**$_2$*

The powder pattern of 0.5cHANE@**neb1**-Zn**Im**$_2$ was visually identified to match the isostructural cyclohexanol solvate of Co**Im**$_2$, CSD code EQOCES[19], which crystallizes in an orthorhombic unit cell with *Fdd2* space group, and contains one metal atom and two imidazolates in its asymmetric unit, as well as the solvent guest molecule, located on the crystallographic inversion center. The initial structure model was created by replacing the framework Co atoms with Zn and replacing the OH group of cyclohexanol with a hydrogen atom. The resulting structural model was then subjected to Rietveld refinement in TOPAS Academic v. 7.[15]

The initial step of the Rietveld procedure involved refining the zero point, unit cell parameter, Chebyshev polynomial background function and pseudo-Voigt peak shape function. In the next step, the position of the Zn atom was refined, while positions and orientations of imidazolate and cyclohexane fragments were refined with rigid body constraints. Finally, atomic thermal motion was described with a single isotropic Debye-Waller factor. During the refinement it was noted that the pattern contained impurity peaks corresponding to the ZnO (COD code 1011258[16]) and **moc**-Zn$_4$**Im**$_8$(H**Im**) (CSD code KUMXEW[20]) phases. Mixed phase refinement with the addition of these two impurity structures was performed.

The resulting structural model was then subjected to periodic DFT geometry optimization with unit cell parameters fixed at their experimental values (for details of the periodic DFT procedure see SI-1.6.). The DFT-optimized structure was used to define the rigid bodies for the final refinement cycle. Crystallographic parameters of the 0.5cHANE@**neb1**-Zn**Im**$_2$ structure can be found in Table S8, the Rietveld plot of the simulated and experimental diffraction profiles is shown in Figure S55.

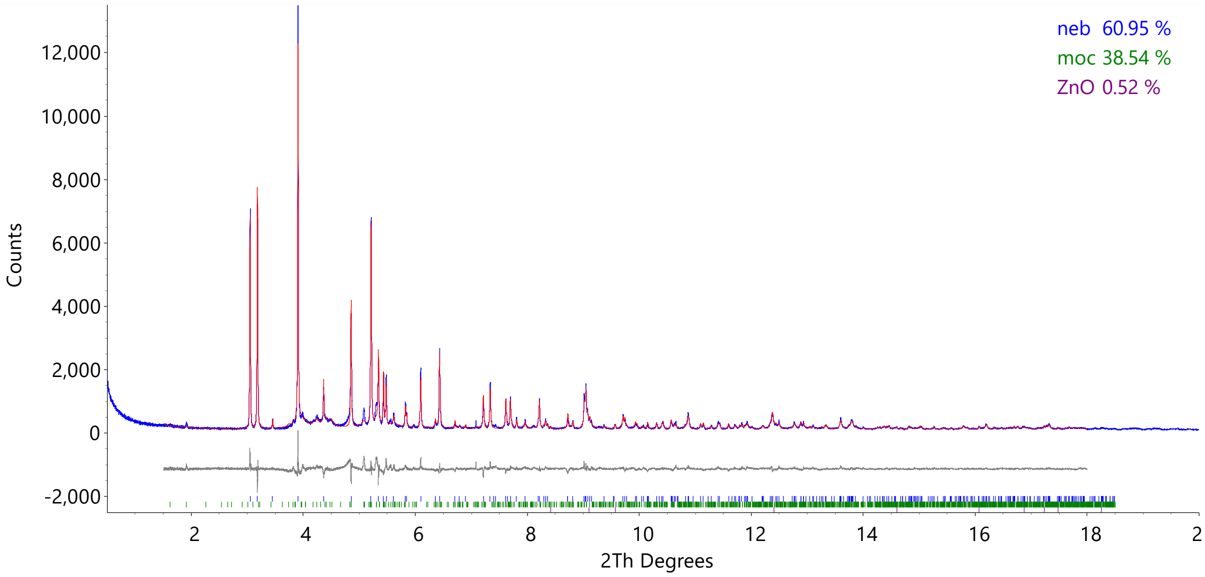

Figure S55. Rietveld refinement plot for 0.5cHANE@**neb1**-Zn**Im**$_2$ structure. The experimental profile is shown in blue, calculated profile in red, and the difference curve is shown in grey.

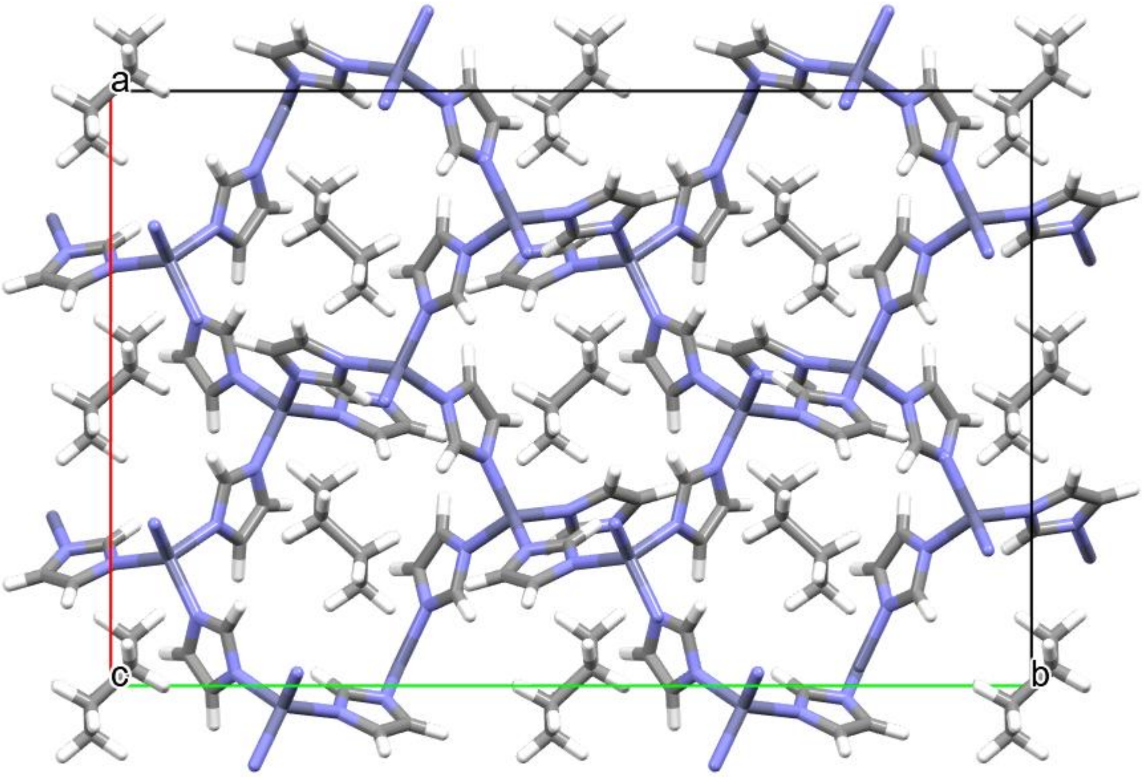

Figure S56. A capped sticks image of the crystal structure of 0.5cHANE@**neb1**-Zn**Im**$_2$ looking down the *c* axis.

*2.3.2. Crystal structures of 0.88PhMe@**crbT**-Zn**Im**$_2$ and 0.49AcPhe@**crbA**-Zn**Im**$_2$*

Comparison of the PXRD patterns of 0.88PhMe@**crbT**-Zn**Im**$_2$ and 0.49AcPhe@**crbA**-Zn**Im**$_2$ materials[*] against the simulated patterns of ZIF materials found in CSD has not revealed any closely-matching structures, therefore full *ab initio* structure determination had to be performed.

The powder patterns of both materials were indexed using DICVOL06[11] and N-TREOR[12] algorithms, *via* the interface of EXPO2014[13] program, followed by Le Bail pattern decomposition[14] and space group determination. The pattern of 0.88PhMe@**crbT**-Zn**Im**$_2$ was indexed with an orthorhombic cell of the volume of 2570 Å^3 and *Pnnm* space group symmetry, while 0.49AcPhe@**crbA**-Zn**Im**$_2$ was indexed with a monoclinic cell of 4503 Å^3 and assigned the $P2_1/c$ space group.

Structure solution was performed using the direct methods procedure implemented in EXPO2014, including the COVMAP[35] procedure for locating additional atoms by analyzing the covariance matrix. Peaks of the ZnO impurity were subtracted from the experimental PXRD pattern with the help of Pawley peak intensity refinement performed in TOPAS Academic 7. The impurity peaks had to be removed so as not to interfere with direct methods structure solution. The found structural model of 0.88PhMe@**crbT**-Zn**Im**$_2$ contained Zn atoms at distances of ~6 Å, appropriate for a ZIF structure. Moreover, molecular fragments in the shape of distorted 5-membered rings were identified between the Zn atoms, strongly suggesting the positions of **Im**$^-$ linkers. This initial model suggested that the asymmetric unit contains one Zn atom, one full **Im**$^-$ fragment, and two half **Im**$^-$ fragments located on

[*] Amounts of guest reported in this crystallography section are determined from Rietveld refinement; in all other sections of the text, an average of NMR and TGA quantification is reported instead

special crystallographic positions. Positions of toluene guest molecules could not be identified from the initial structural model.

In the case of 0.49AcPhe@**crbA**-Zn**Im**$_2$ direct methods structure solution revealed a far more complex model with four Zn atoms in the asymmetric unit. Gratifyingly, all these Zn atoms were arranged at positions and distances consistent with ZIF connectivity, however peaks of electron density found between the metal centers could not be resolved into chemically-sensible molecular fragments. Instead, **Im**$^-$ linkers were manually inserted between the Zn atoms in chemically-sensible positions using the program XSeed.[9]

The structural models described above were further processed on TOPAS Academic v7.[15] First, the positions of toluene and acetophenone guests were identified using the Simulated Annealing (SA) algorithm. During the SA procedure, the unit cell parameters, atom positions, pseudo-Voigt peak shapes and Chebyshev background polynomials were kept fixed, only the positions, orientations and occupancies of guest molecule fragments were allowed to vary. The occupancies were constrained to remain within the limits of 0 to 1. In both structures, the final solution was obtained by defining two symmetry-independent molecular fragments representing solvent guests.

After this initial SA procedure, both structures were subjected to Rietveld refinement, where positions of all Zn atoms, as well as positions and orientations of imidazolate and solvent guest molecules were refined. Restraints were applied to the Zn-N bond distances and N-Zn-N bond angles. Even after this refinement step, the orientation of imidazolate fragments remained somewhat ambiguous, as 180° rotation of each fragment around the Zn-Zn axis does not lead to significant changes in the calculated PXRD pattern. In order to accurately assign the orientations of all the linker fragments we analyzed the structures with periodic DFT (see section SI-1.6 for the general methodology). The structure of 0.88PhMe@**crbT**-Zn**Im**$_2$ contained three symmetry-independent imidazolate fragments, which allowed us to consider all possible arrangements resulting from rotating each of the linkers with respect to the Zn-Zn axis. Each of these configurations was then optimized with periodic DFT, after removal of the disordered solvent guest molecules, resulting in the overall energy ranking of structures with different linker orientations. The DFT-calculated energies of all structure rearrangements are shown in Table S6.

The structure of 0.49AcPhe@**crbA**-Zn**Im**$_2$ contained eight symmetry-independent imidazolate linkers, making the number of distinct linker orientations far larger than in the previous case. Rather than exploring all possible combinations of linker orientations, we first performed single permutations, where only one linker would be rotated around the Zn-Zn axis, while the other ones would remain in their original positions. In the next step, we performed double rotations for all distinct linker pairs. This resulted in a total of 36 configurations, all of which were geometry-optimized with periodic DFT, and their energies are shown in Table S7.

The lowest energy configurations found in DFT calculations were used in the final step of Rietveld refinement of both ZIF structures. Rigid body geometries of imidazolate fragments were adjusted to the

geometries extracted from DFT-optimized structures. The final set of refined parameters included the background polynomial function, zero point, peak shape parameters, positions of Zn, positions and orientations of imidazolate rigid bodies (subject to Zn-N bond length and N-Zn-N angle restraints), positions, orientations and occupancies of guest molecules, as well as a single isotropic Debye-Waller factor. Rietveld refinement gave the guest composition of 0.49AcPhe@**crbA**-Zn**Im**$_2$ and 0.88PhMe@**crbT**-Zn**Im**$_2$, however, especially due to the high disorder of toluene guest, we report guest incorporations from the average of TGA and NMR measurements throughout this work, as we believe they are more accurate (0.51AcPhe@**crbA**-Zn**Im**$_2$ and 0.65PhMe@**crbT**-Zn**Im**$_2$). The final Rietveld plots of 0.49AcPhe@**crbA**-Zn**Im**$_2$ and 0.88PhMe@**crbT**-Zn**Im**$_2$ are shown in Figures S57 and S58, respectively. The crystal structures of both materials are shown in Figure S59.

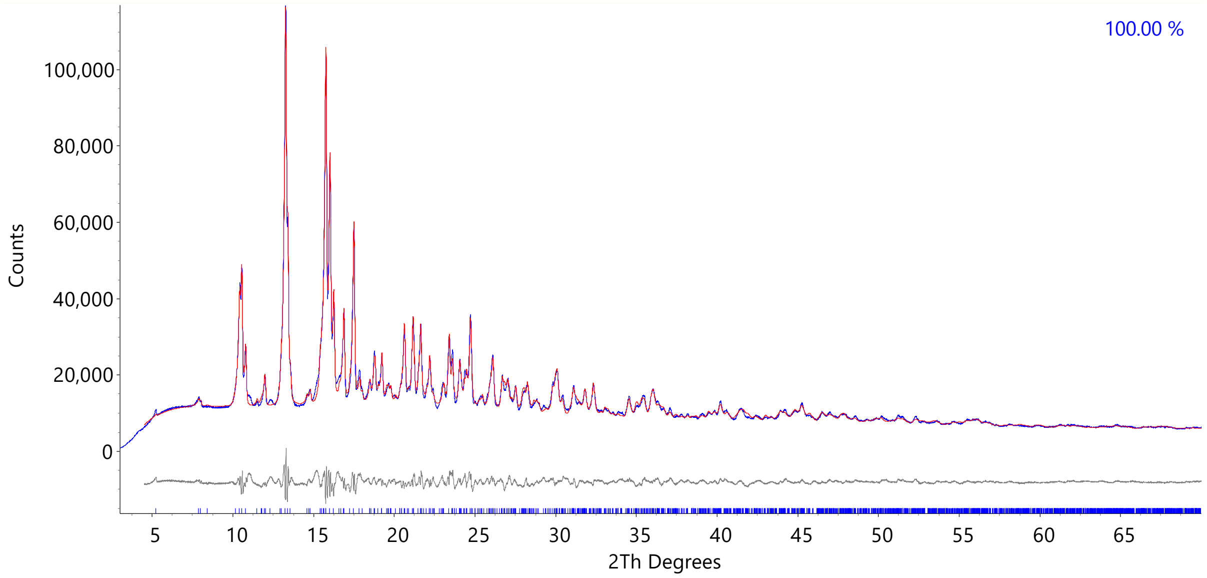

Figure S57. Rietveld refinement plot for 0.49AcPhe@**crbA**-Zn**Im**$_2$ structure. The experimental profile is shown in blue, calculated profile in red, and the difference curve is shown in grey.

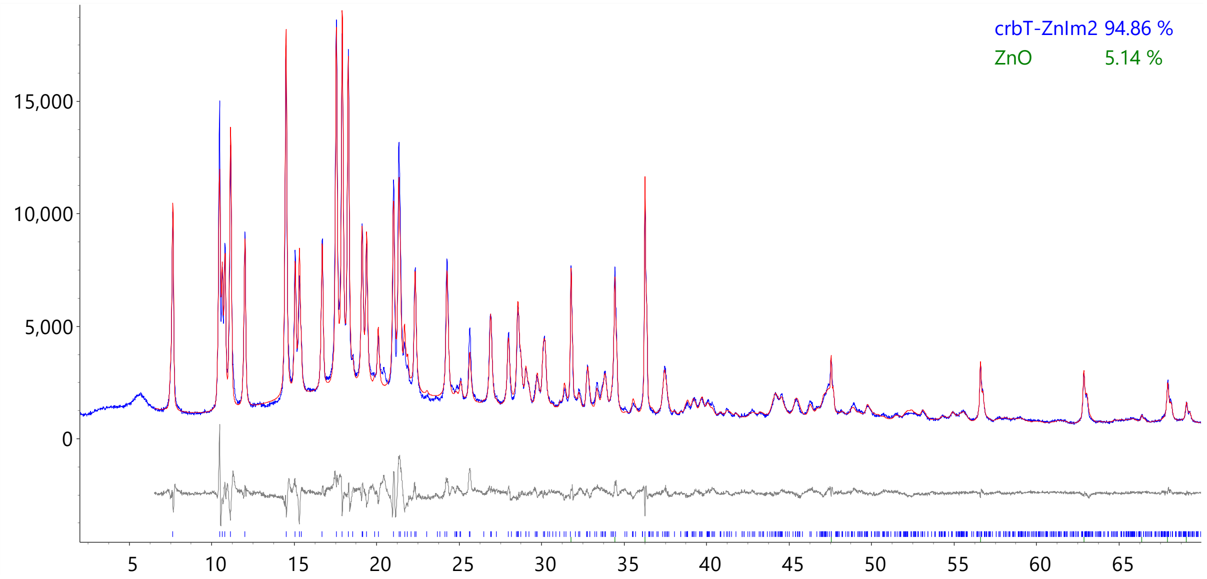

Figure S58. Rietveld refinement plot for 0.88PhMe@**crbT**-Zn**Im**$_2$ structure. The experimental profile is shown in blue, calculated profile in red, and the difference curve is shown in grey.

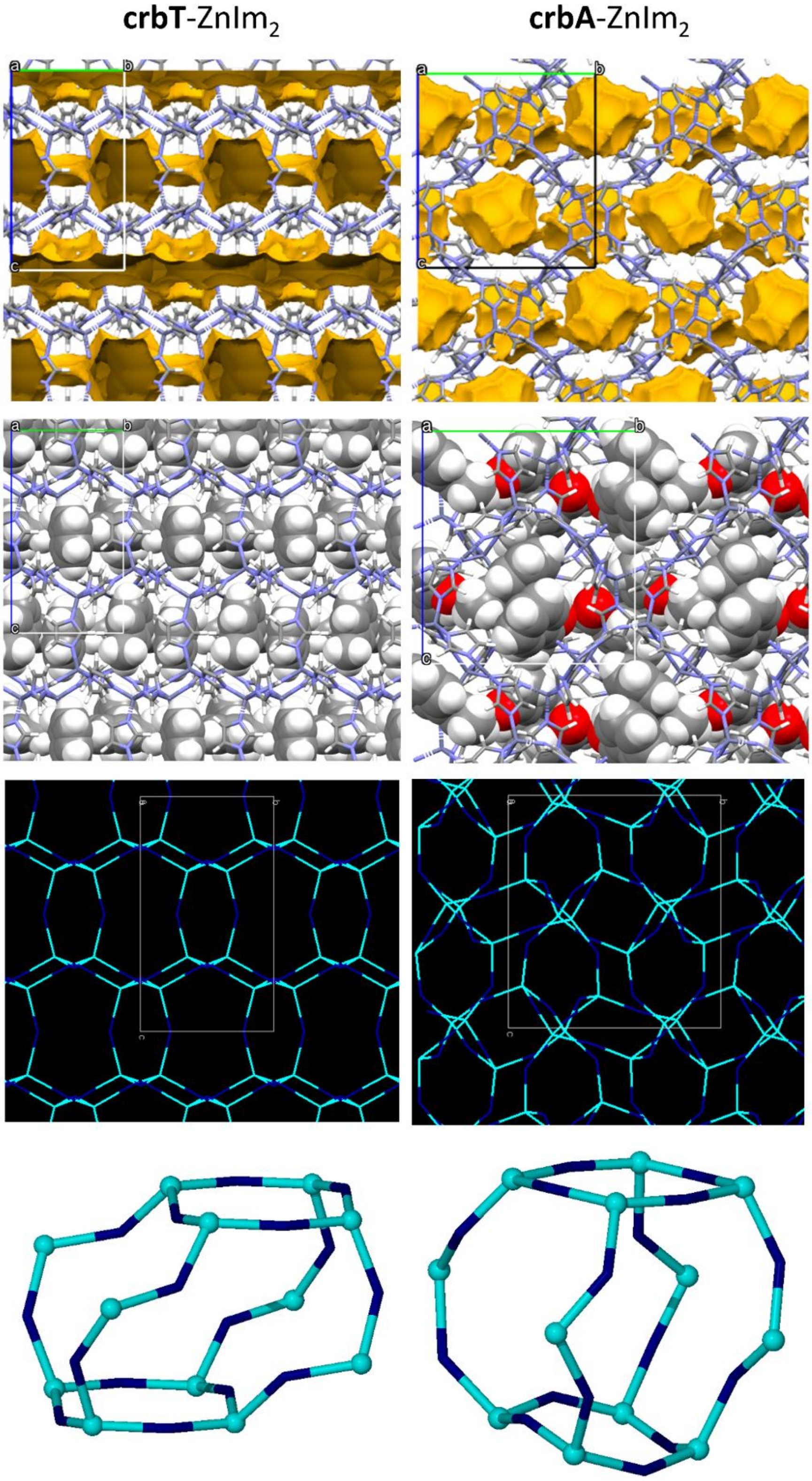

Figure S59. Top two sets of panels: Graphical representations of the 0.88PhMe@**crbT**-Zn**Im**$_2$ (left) and 0.51AcPhe@**crbA**-Zn**Im**$_2$ (right) materials. The crystal structures are viewed along the *a* axis and presented through the capped sticks representation, while the guests are displayed in the spacefill representation. The guests are removed from the top two panels and solvent accessible void surface is plotted in yellow. Third set of panels: The crystal packing of 0.88PhMe@**crbT**-Zn**Im**$_2$ (left) and 0.51AcPhe@**crbA**-Zn**Im**$_2$ represented in a reduced node-and-linker image where zinc ions are depicted in light blue, whereas the centroids of imidazolate ions are depicted in dark blue. Bottom set of panels: The **crb** building cages of **crbT**-Zn**Im**$_2$ (left) and **crbA**-Zn**Im**$_2$ depicted through the reduced node-and-linker image.

Table S6. Structure rearrangements of **crbT-**Zn**Im**$_2$ and their relative energies. The structure contains three symmetry-independent imidazole molecules. The "+" sign next to an imidazolate linker means no reorientation, while a "-" sign means 180° rotation around the N-N axis. The lowest energy configuration was used for the final Rietveld refinement step.

Imidazolate orientation			DFT energy per unit cell / eV	DFT energy per ZIF formula unit / eV	Relative energy / kJ mol^{-1}
**+**	**+**	**-**	**-31500.89**	**-3937.61**	**0.00**
-	-	-	-31500.88	-3937.61	0.14
-	+	-	-31500.54	-3937.57	4.27
+	-	-	-31500.48	-3937.56	4.99
-	+	+	-31498.82	-3937.35	25.08
+	-	+	-31498.80	-3937.35	25.25
+	+	+	-31498.75	-3937.34	25.91
-	-	+	-31497.07	-3937.13	46.10

Table S7. Structure rearrangements of **crbA**-Zn**Im**$_2$ and their energies relative to the initial configuration of imidazolate linkers. The lowest energy rearrangement 2+7 (highlighted in grey) was used for the final Rietveld refinement step.

Structure rearrangement		DFT energy per unit cell / eV	DFT energy per ZIF formula unit / eV	Energy relative to initial structure / kJ mol^{-1}
Initial structure		-63001.98	-3937.62	0.00
Single imidazolate rearrangement	1	-63001.72	-3937.61	1.56
	2	-63001.20	-3937.58	4.67
	3	-63001.55	-3937.60	2.58
	4	-63001.32	-3937.58	3.96
	5	-63002.30	-3937.64	-1.94
	6	-63002.08	-3937.63	-0.62
	7	-63002.41	-3937.65	-2.63
	8	-63001.74	-3937.61	1.47
Double imidazolate rearrangement	1+3	-63001.72	-3937.61	1.56
	1+6	-63001.93	-3937.62	0.29
	1+5	-63001.92	-3937.62	0.37
	3+6	-63001.38	-3937.59	3.64
	3+5	-62994.37	-3937.15	45.90
	5+6	-63001.60	-3937.60	2.26
	2+4	-63000.16	-3937.51	10.95
	**2+7**	**-63002.77**	**-3937.67**	**-4.80**
	2+8	-63002.05	-3937.63	-0.45
	4+7	-63000.45	-3937.53	9.22
	4+8	-63001.91	-3937.62	0.44
	7+8	-63001.45	-3937.59	3.18
	1+2	-63000.62	-3937.54	8.16
	1+4	-63000.42	-3937.53	9.37
	1+7	-63001.85	-3937.62	0.80
	3+8	-63001.33	-3937.58	3.93
	6+8	-63001.85	-3937.62	0.80
	5+8	-63002.08	-3937.63	-0.59
	5+7	-63001.69	-3937.61	1.76
	6+7	-63001.15	-3937.57	4.98
	1+8	-63001.88	-3937.62	0.57
	2+3	-63000.56	-3937.54	8.54
	2+5	-63001.71	-3937.61	1.64
	2+6	-63001.22	-3937.58	4.60
	3+4	-63001.36	-3937.58	3.75
	3+7	-63001.80	-3937.61	1.11
	4+5	-63001.91	-3937.62	0.41
	4+6	-63001.68	-3937.61	1.77

Table S8. General and crystallographic data for the structures solved from PXRD data

**Crystal Parameters**	**0.5cHANE@neb2-ZnIm$_2$**	**0.88PhMe@crbT-ZnIm$_2$**	**0.49AcPhe@crbA-ZnIm$_2$**
**Molecular formula**	$Zn(C_3H_3N_2)_2\,(C_6H_{12})_{1/2}$	$Zn(C_3H_3N_2)_2\,(C_7H_5)_{0.88}$	$Zn_4(C_3H_3N_2)_8\,(C_8H_8O)_{1.95}$
$\boldsymbol{M_r}$	241.58	277.53	1031.77
**Crystal system**	Orthorhombic	Orthorhombic	Monoclinic
**Space group**	*Fdd*2	*Pnnm*	$P2_1/c$
**Crystal data:**			
***a*, Å**	17.7406(3)	15.8922(7)	18.2394(6)
***b*, Å**	27.4599(5)	9.5664(4)	15.0107(6)
***c*, Å**	9.10804(16)	16.9136(7)	17.6170(6)
$\boldsymbol{\alpha}$**, °**	90	90	90
$\boldsymbol{\beta}$**, °**	90	90	110.9756(17)
$\boldsymbol{\gamma}$**, °**	90	90	90
***V*, Å^3**	4437.04(13)	2571.38(19)	4503.7(3)
Z	16	8	4
$\boldsymbol{D_{calc}}$**, g cm^{-3}**	1.447	1.434	1.520
Radiation type	synchrotron / 0.41260 Å	CuK_α / 1.54184 Å	CuK_α / 1.54184 Å
***T*, K**	298	298	298
**Sample mounting**	capillary	capillary	flat plate
***F*(*000*)**	1984	1128.6	2097.92
$\boldsymbol{R_{wp}}$	0.081	0.069	0.044
$\boldsymbol{R_p}$	0.103	0.053	0.034
$\boldsymbol{R_{Bragg}}$	0.039	0.028	0.016
$\boldsymbol{X^2}$	1.699	3.170	3.450
**CCDC number**	2381143	2381139	2381142

Table S9. Comparison of different forms of **crb** and **neb** topology zinc imidazolate. Porosity parameters calculated with the Pore Analyzer function in Mercury.

	CSD code	space group	Unit cell volume, Å^3	Unit cell volume per Zn, Å^3	T/V, nm^{-3}	surface area at 77 K (calc for N_2), m^2/g	maximum pore diameter (calc), Å	pore limiting diameter (calc), Å	void fraction, %
**crb1**	VEJYIT	*Pbca*	5706.62	356.66	2.80	1494.84	6.01	5.04	23.4
**crb2**	GITTEJ	*P2/n*	4414.43	275.90	3.62	465.39	6.80	1.70	11.9
**crb3**	VEJYEP	$P2_1/n$	2195.87	274.48	3.64	427.50	6.12	1.87	11.0
**crbA**	this work	$P2_1/c$	4503.67	281.48	3.55	511.10	6.40	1.96	12.0
**crbT**	this work	*Pnnm*	2571.39	321.42	3.11	321.36	4.75	3.66	14.7
**neb1**	KUDJOK	*Fdd2*	4310.48	269.41	3.71	236.61	4.97	2.15	8.1
**neb2**	KEVLEE	*Fdd2*	4115.42	257.21	3.89	1.03	4.05	2.15	6.0

2.4. Thermogravimetric studies

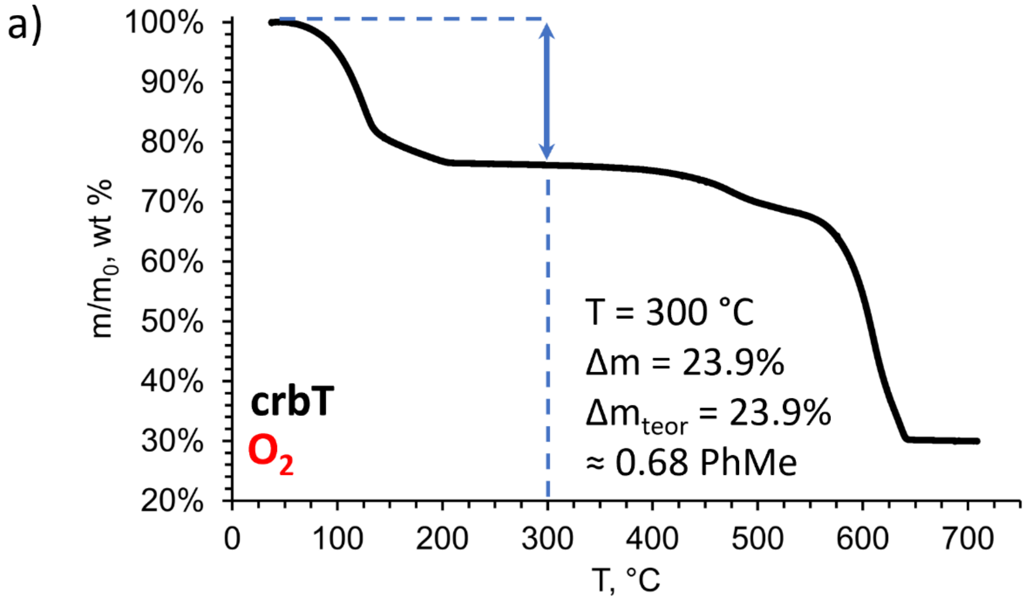

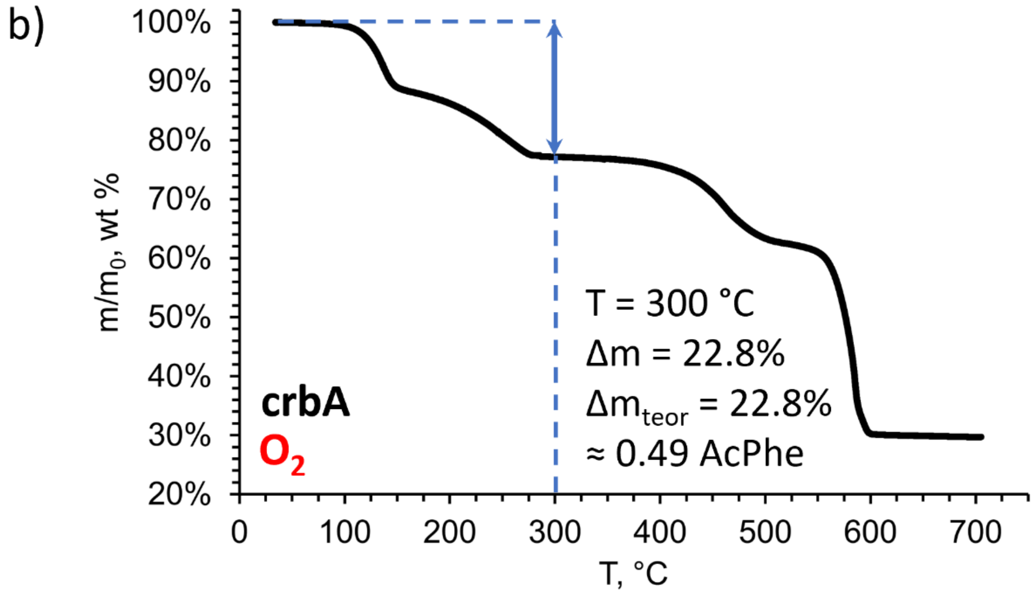

Figure S60. TGA curves collected under 30 mL/min flow of oxygen, with 7 °C/min heating for: a) 0.65PhMe@**crbT**-Zn**Im**$_2$ and b) 0.51AcPhe@**crbA**-Zn**Im**$_2$.

2.5. NMR results

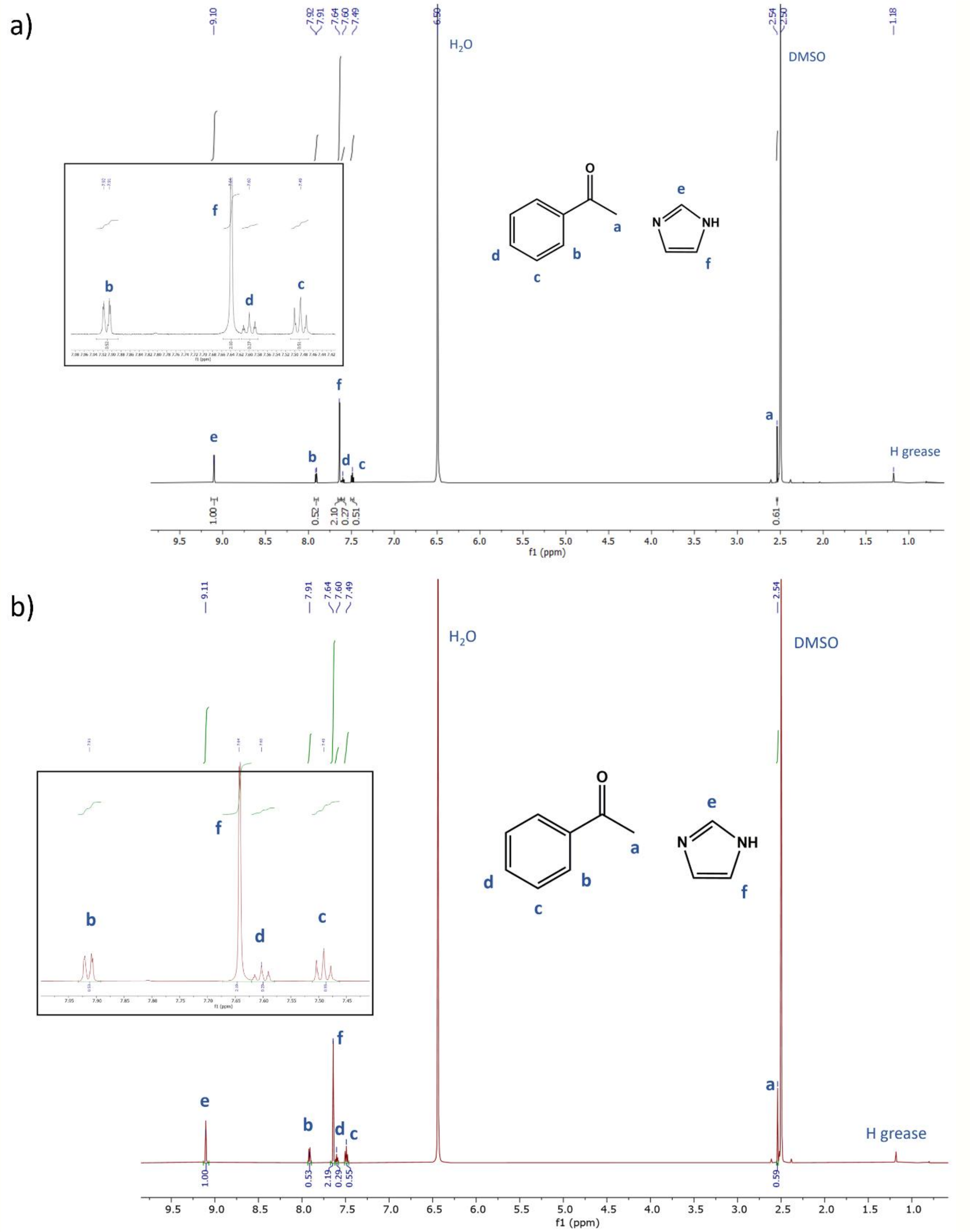

Figure S61. Assigned NMR spectrum of a) as synthesized 0.51AcPhe@**crbA**-Zn**Im**$_2$ (black trace) and b) 0.51AcPhe@**crbA**-Zn**Im**$_2$ washed with acetone and dried (red trace).

^{1}H NMR (600 MHz, DMSO, DCl) δ 9.10 (s, 1H), δ 7.91 (d, J = 7.1 Hz, 0.5H), δ 7.64 (s, 2H), δ 7.60 (t, J = 7.4 Hz, 0.3H), 7.49 (t, J = 7.9 Hz, 0.5H), δ 2.54 (s, 0.6H).

The amount of enclathrated AcPhe in the as synthesized product was calculated from the NMR spectrum (Figure S61.a) to be 0.26 AcPhe per imidazole molecule, leading to a formula of 0.52AcPhe@**crbA**-Zn**Im**$_2$. Washing with acetone didn't change the amount of enclathrated AcPhe, calculated at 0.27 AcPhe per imidazole molecule (Figure S61.b)

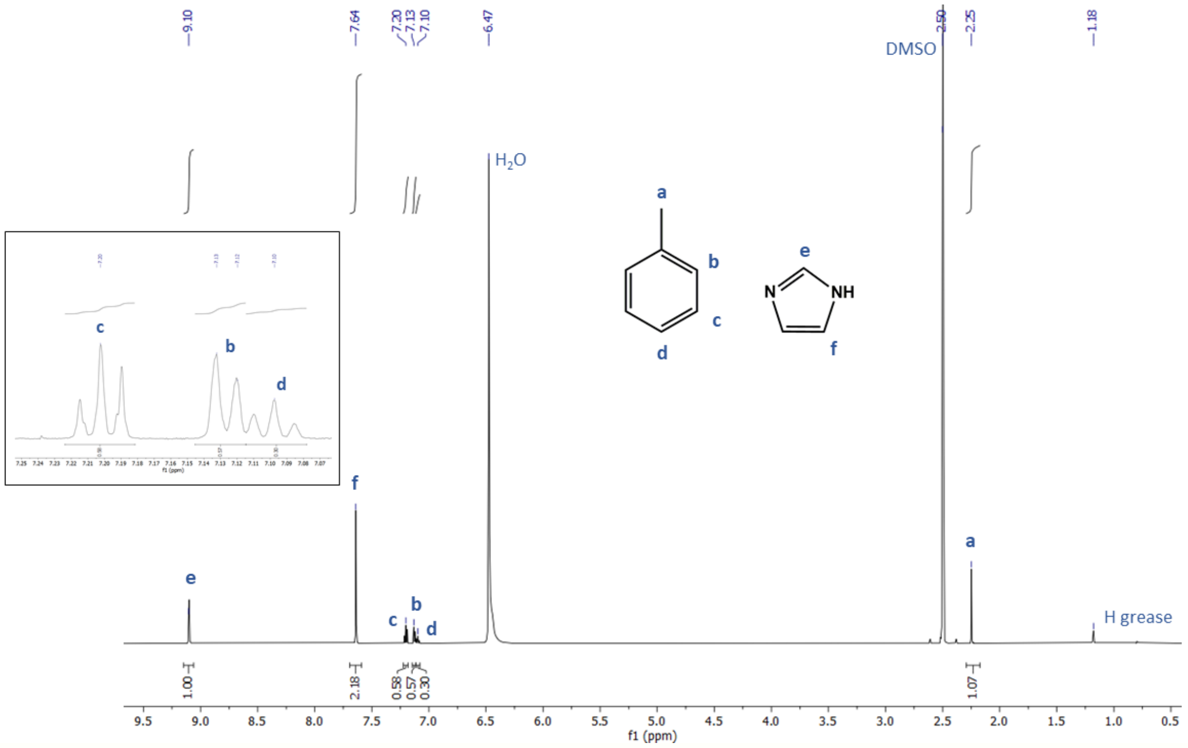

Figure S62. Assigned NMR spectrum of as synthesized 0.65PhMe@**crbT**-Zn**Im**$_2$.

^{1}H NMR (600 MHz, DMSO, DCl) δ 9.10 (s, 1H), δ 7.64 (s, 2H), δ 7.20 (t, J = 7.5 Hz, 0.6H), 7.13 (d, J = 7.5 Hz, 0.6H), 7.10 (t, J = 7.0 Hz, 0.3H), δ 2.25 (s, 1H).

The amount of enclathrated PhMe in the as synthesized product was calculated from the NMR spectrum (Figure S62) to be 0.31 PhMe per imidazole molecule, leading to a formula of 0.62PhMe@**crbA**-Zn**Im**$_2$.

Below is a table containing the results of three different techniques used to quantify the amount of guest in the newly prepared **crb** materials. Rietveld refinement is less accurate in this case, particularly due to the heavy disorder of toluene in the **crbT** material, so the average of NMR and TGA quantifications was taken for the final compositions: 0.65(3)PhMe@crbT-Zn**Im**2 and 0.51(2)AcPhe@**crbA**-Zn**Im**$_2$

Table S10. Comparison of TGA, Rietveld and NMR obtained occupancies for the **crbT** and **crbA** phases:

compound	*x*(Rietveld)	*x*(TGA)	*x*(NMR)	*x*(average, NMR and TGA)
*x*PhMe@**crbT**-Zn**Im**$_2$	0.88	0.68	0.62	**0.65(3)**
*x*AcPhe@**crbA**-Zn**Im**$_2$	0.49	0.49	0.52	**0.51(2)**

2.6. IR results

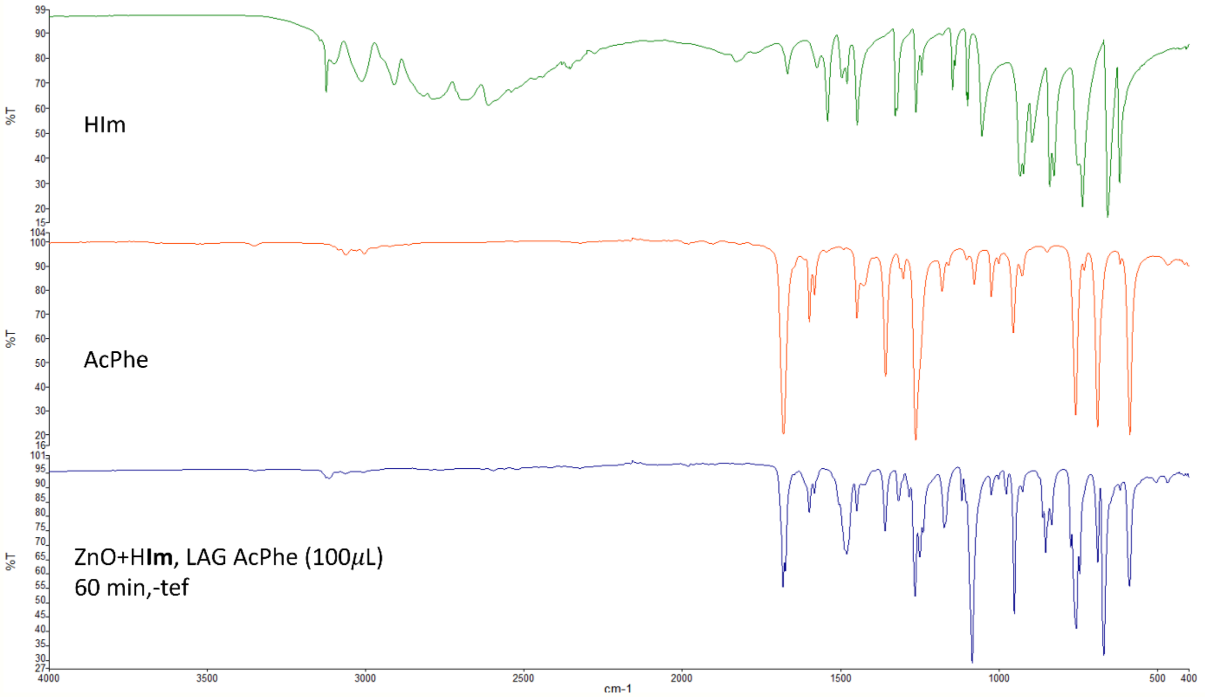

Figure S63. ATR-FTIR spectra of imidazole (top, green), acetophenone (middle, red) and the product of milling ZnO and H**Im** with AcPhe in a Teflon™ jar for 1 h (bottom, blue).

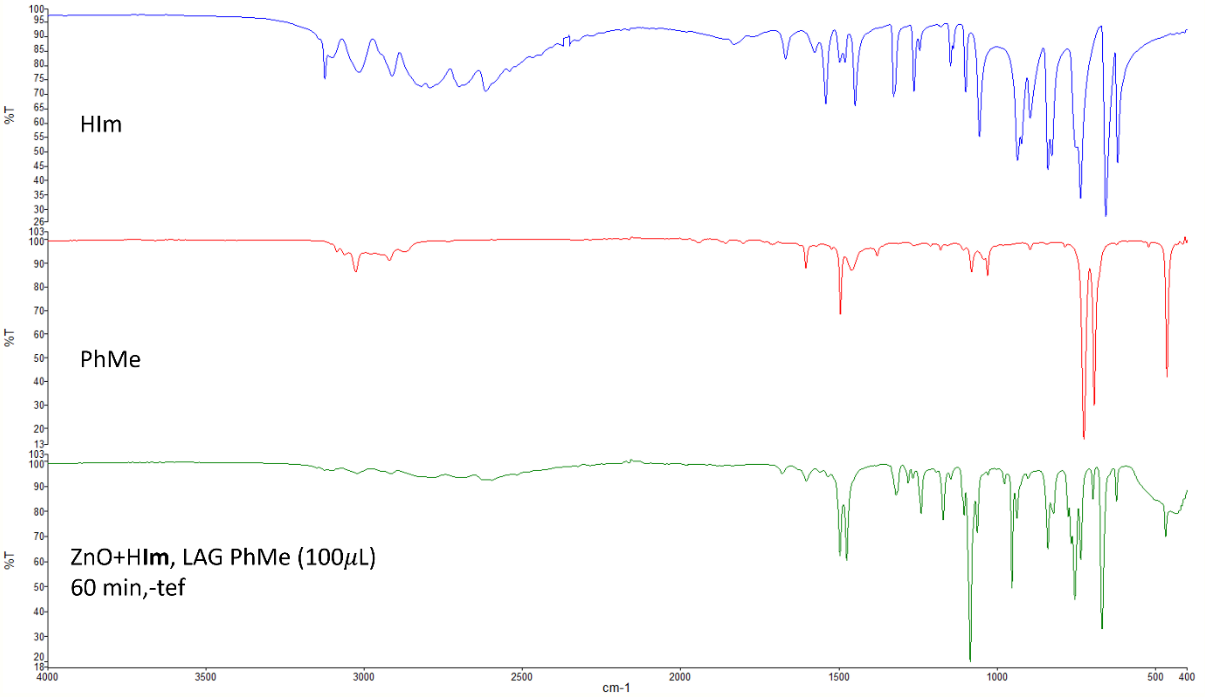

Figure S64. ATR-FTIR spectra of imidazole (top, blue), toluene (middle, red) and the product of milling ZnO and H**Im** with PhMe in a Teflon™ jar for 1 h (bottom, green).

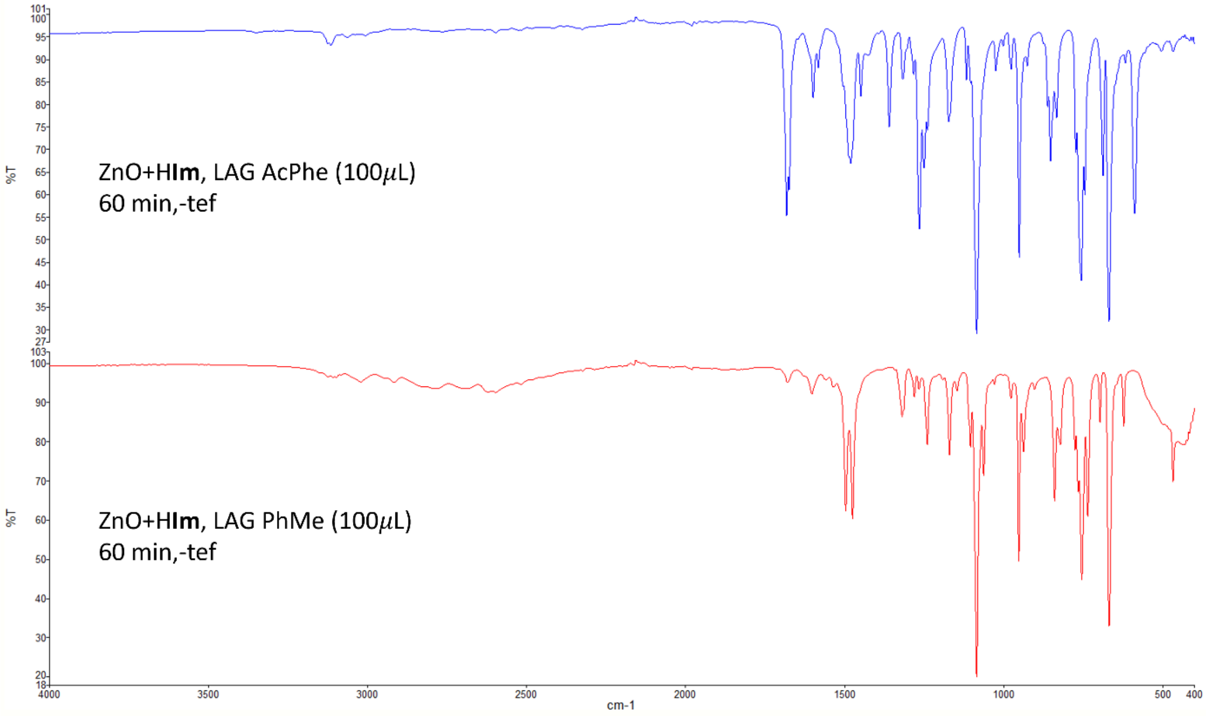

Figure S65. ATR-FTIR spectra of the products of milling ZnO and H**Im** in a Teflon™ jar for 1 h with AcPhe (top, blue) with PhMe (bottom, red).

2.7. DSC results

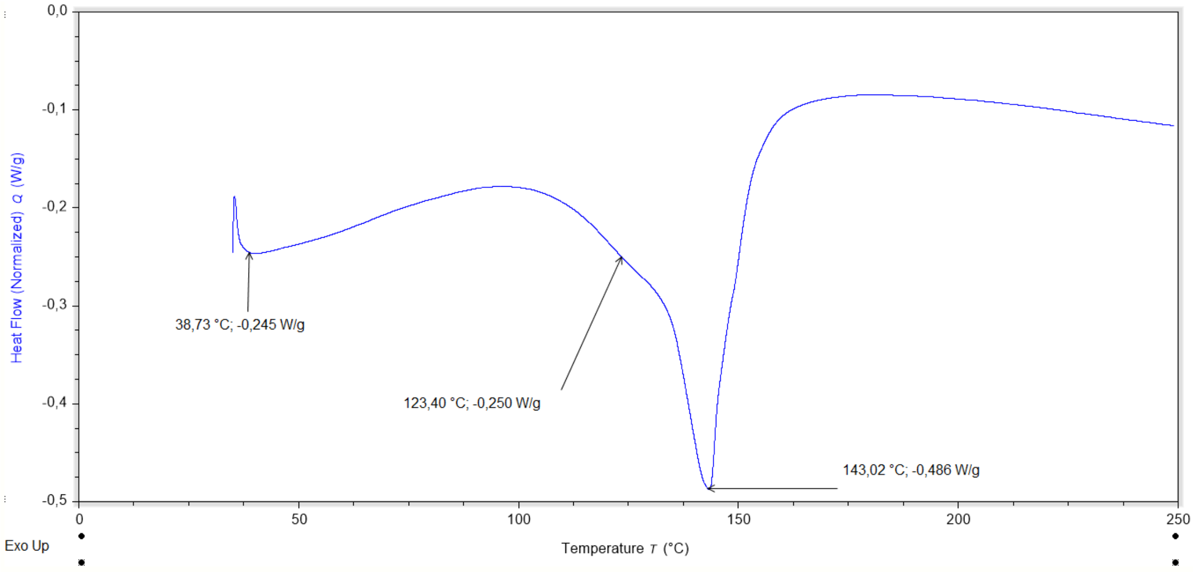

Figure S66. DSC curve of the product of milling ZnO and H**Im** in a Teflon™ jar for 1 h with AcPhe, washed with acetone.

2.8. N_2 adsorption results

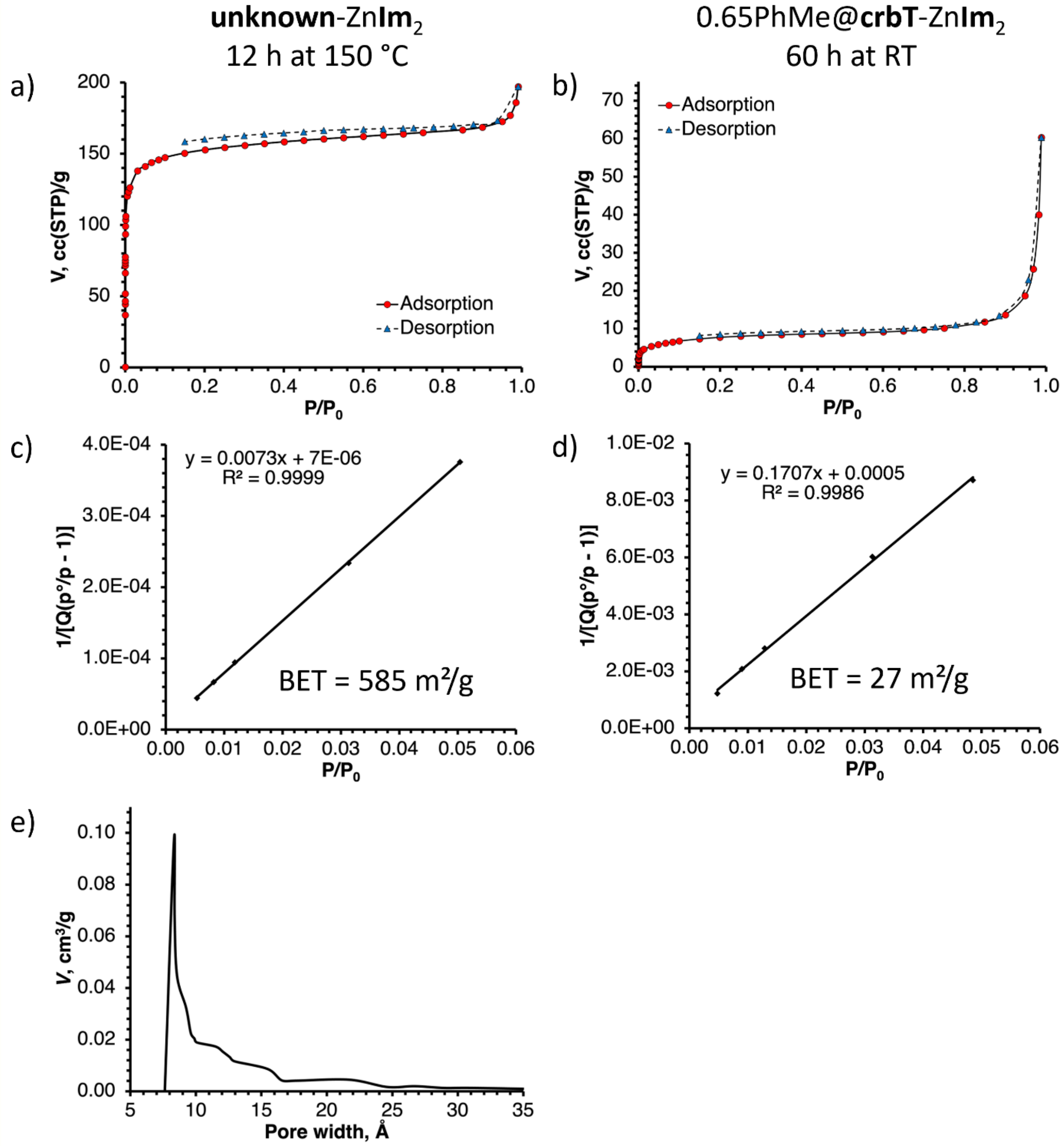

Figure S67. N_2 sorption isotherms at 77 K for a) 0.51AcPhe@**crbA**-Zn**Im**$_2$ activated for 12 h at 150 °C (PXRD post adsorption in Fig. S68.f), b) 0.65PhMe@**crbT**-Zn**Im**$_2$ activated for 60 h under vacuum at room temperature (PXRD post adsorption in Fig. S68.c). BET plots with the calculated BET surface areas for c) 0.51AcPhe@**crbA**-Zn**Im**$_2$ activated for 12 h at 150 °C and d) 0.65PhMe@**crbT**-Zn**Im**$_2$ activated for 60 h under vacuum at room temperature. e) Pore size distribution for 0.51AcPhe@**crbA**-Zn**Im**$_2$ activated for 12 h at 150 °C, calculated using the SF method.

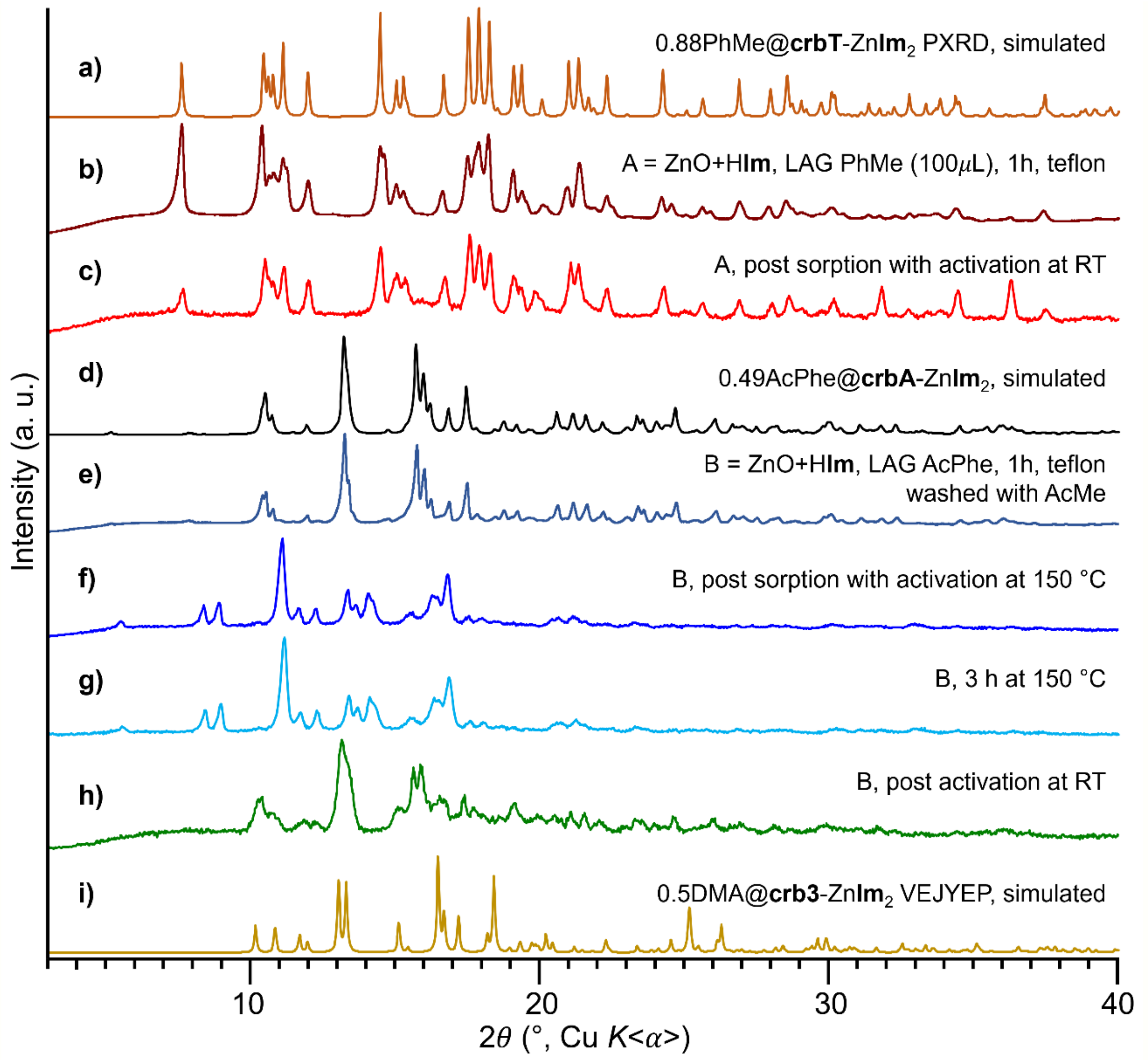

Figure S68. Simulated PXRD patterns of a) 0.88PhMe@**crbT**-Zn**Im**$_2$ (solved from PXRD data); d) 0.49AcPhe@**crbA**-Zn**Im**$_2$ (solved from PXRD data); and i) 0.5DMA@**crb3**-Zn**Im**$_2$ (CSD code VEJYEP). PXRD patterns of b) A = the product of milling ZnO and H**Im** with PhMe in a Teflon™ jar for 1 h; c) A, post sorption, with activation under high vacuum at RT for 60 h; e) B = the product of milling ZnO and H**Im** with AcPhe in a Teflon™ jar for 1 h, washed with acetone; f) B, post sorption with activation under high vacuum at 150 °C for 12 h; g) B, heated at 150 °C in air for 3 h; and h) B, post activation under high vacuum at RT (18 h).

2.9. DFT results

2.9.1. DFT parameter optimization

To obtain the optimal parameters for the periodic DFT optimizations of ZIF compounds, a series of geometry optimizations were performed on the **moc**-Zn_4**Im**$_8$(H**Im**) structure (CSD code KUMXEW). First, the plane wave basis set cutoff was systematically varied from 300 to 1000 eV (Δ = 50 eV) using a 0.11 Å^{-1} k-point spacing (Figure S69). The resulting final energies corrected for finite basis set converge at a 800 eV cutoff ($dE_{tot}/dlog(E_{cut})<1$ eV, Table S11).

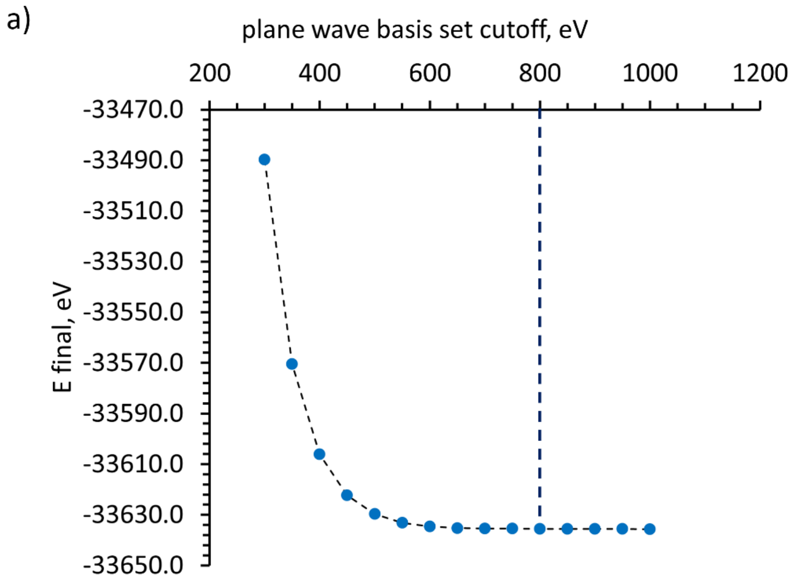

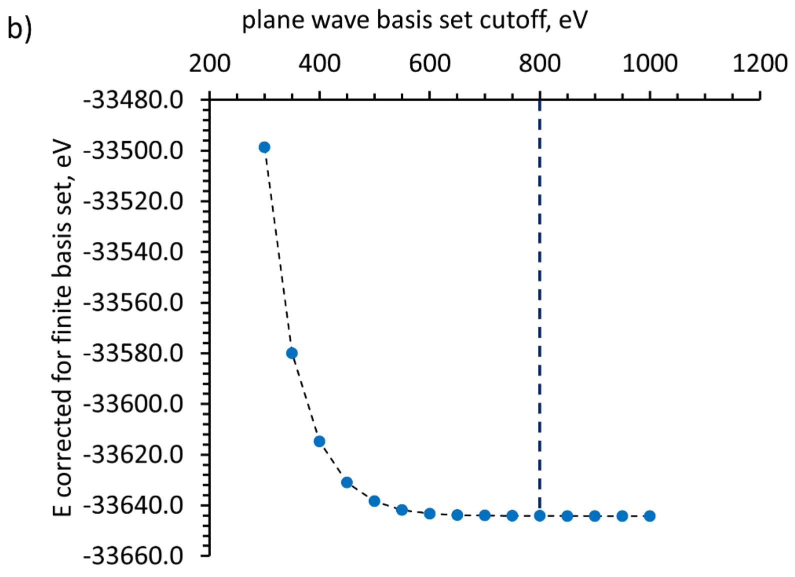

Figure S69. The dependance of a) final energies, and b) final energies corrected for finite basis set obtained by geometry optimization of the **moc**-Zn_4**Im**$_8$(H**Im**) structure (CSD code KUMXEW) in CASTEP on the plane wave basis set cutoff energies (blue circles). The blue dashed line indicates the optimal cutoff energy for these systems.

The Brillouin zone k-point spacing was then systematically varied from 0.03 to 0.11 Å^{-1} (Δ = 0.01 Å^{-1}), while keeping the plane wave basis set cutoff of 800 eV (Figure S70, Table S12). The resulting

final energies corrected for finite basis set reach a minimum and then level off for k-point spacings smaller than 0.06 Å^{-1}, so this spacing was taken as optimal.

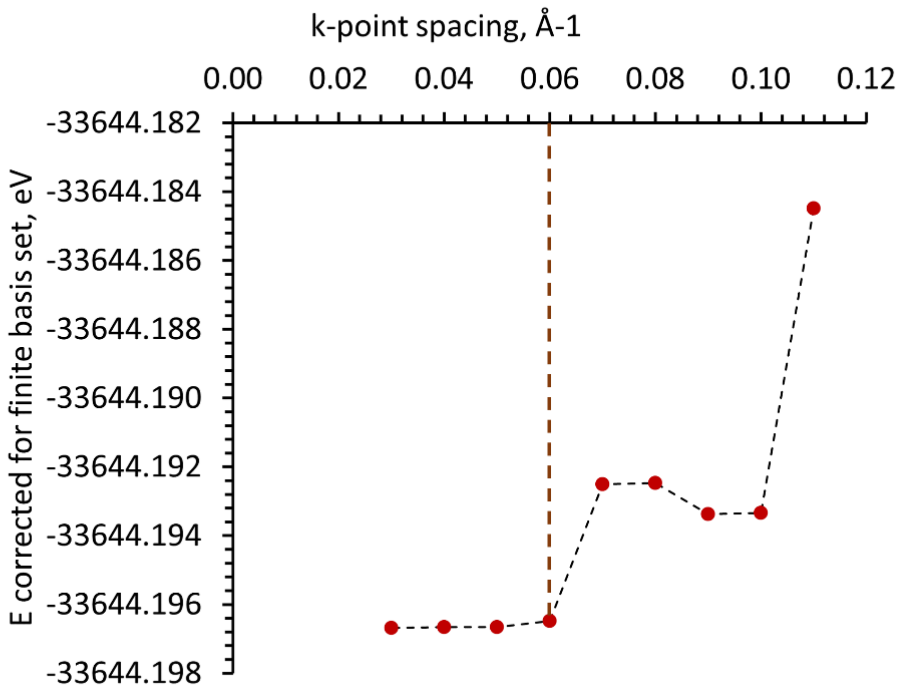

Figure S70. The dependance of final energies corrected for finite basis set obtained by geometry optimization of on the **moc**-Zn_4**Im**$_8$(H**Im**) structure (CSD code KUMXEW) in CASTEP on k-point spacings (red circles). The red dashed line indicates the optimal k-point spacing for these systems.

Table S11. The plane wave basis set cutoff and the corresponding final energies corrected for finite basis set and convergence criteria obtained by geometry optimization of the **moc**-Zn_4**Im**$_8$(H**Im**) structure (CSD code KUMXEW) in CASTEP.

Cutoff, eV	E_{basis}, eV	$dE_{tot}/dlog(E_{cut})$
300	-33498.693	-765.756
350	-33579.965	-399.501
400	-33614.852	-201.213
450	-33631.027	-104.374
500	-33638.421	-56.222
550	-33641.852	-28.395
600	-33643.28	-12.927
650	-33643.878	-5.109
700	-33644.066	-2.347
750	-33644.133	-1.158
800	-33644.184	-0.700
850	-33644.214	-0.655
900	-33644.248	-0.590
950	-33644.276	-0.489
1000	-33644.297	-0.356

Table S12. The k-point spacings and the corresponding final energies corrected for finite basis set and convergence criteria obtained by geometry optimization of the **moc**-Zn_4**Im**$_8$(H**Im**) structure (CSD code KUMXEW) in CASTEP.

k-point spacing, $Å^{-1}$	E_{basis}, eV	$dE_{tot}/dlog(E_{cut})$
0.11	-33644.184	-0.700
0.10	-33644.193	-0.702
0.09	-33644.193	-0.721
0.08	-33644.192	-0.699
0.07	-33644.193	-0.759
0.06	-33644.196	-0.705
0.05	-33644.197	-0.707
0.04	-33644.197	-0.683
0.03	-33644.197	-0.704

2.9.2. Periodic DFT optimization of experimentally obtained zinc imidazolate forms

The starting crystal structures for DFT optimization were taken from the CSD, or solved from PXRD data (**crbT** and **crbA** materials). In all cases guest molecules were deleted from the structure, the C-H bond lengths were normalized to a value of 1.088 Å in Mercury, and disorder was resolved as follows. The **moc** structure was prepared from the KUMXEW CSD entry. The asymmetric unit contains four zinc atoms and nine imidazol(at)e moieties, one of which must be protonated for charge neutrality, but it can not be determined from the CSD entry which one. Since both N10 and N11 are non-bridging, the choice narrows to them, so both protonation options were tested and the lower energy one (N11-H version) was chosen.

The **crb2** structure was prepared from the GITTEJ CSD entry. One of the imidazolate ligands is disordered such that either atoms C7 and H7, or atoms C6? And H6? Could be part of the asymmetric unit. Both options were tested, and while the "C7-version" was lower in energy, the "C6?-version" was a better match to the CSD database **crb2** structure (as seen by PXRD comparison in Figure S82), and was thus chosen.

Table S13. Results of periodic DFT optimization of experimentally obtained zinc imidazolate forms

**topology**	**CSD code**	**Enthalpy; eV**	**Formula units in cell**	**Compositio n**	**Enthalpy, adjusted; eV**	**dE, kJ/mol**	**Density , g/cm^3**
imidazole	IMAZOL17	-4363.881	4	H**Im**	N/A	N/A	1.30
**moc**	KUMXEW	-33685.123	8	Zn**Im**$_2$ (H**Im**)$_{0.25}$	-3937.898	**0.00**	**1.60**
**coi**	IMIDZB07	-63005.929	16	Zn**Im**$_2$	-3937.871	**2.66**	**1.57**
**zni**	IMIDZB02	-63005.746	16	Zn**Im**$_2$	-3937.859	**3.76**	**1.53**
**nog**	HIFWAV	-78753.797	20	Zn**Im**$_2$	-3937.690	**20.09**	**1.13**
**crb3**	VEJYEP	-31501.468	8	Zn**Im**$_2$	-3937.683	**20.71**	**1.20**
**crbA**	this work	-63002.786	16	Zn**Im**$_2$	-3937.674	**21.61**	**1.18**
**cag**	VEJYUF01	-63002.725	16	Zn**Im**$_2$	-3937.670	**21.98**	**1.19**
**neb2**	KEVLEE	-15750.646	4	Zn**Im**$_2$	-3937.661	**22.84**	**1.28**
**crb2**	GITTEJ	-63002.492	16	Zn**Im**$_2$	-3937.656	**23.38**	**1.20**
**neb1**	KUDJOK	-15750.565	4	Zn**Im**$_2$	-3937.641	**24.79**	**1.19**
**crbT**	this work	-31500.894	8	Zn**Im**$_2$	-3937.612	**27.63**	**1.03**
**10mr**	GOQSIQ	-157504.144	40	Zn**Im**$_2$	-3937.604	**28.42**	**0.98**
**crb1**	VEJYIT	-63000.106	16	Zn**Im**$_2$	-3937.507	**37.77**	**0.95**
**afi**	IMIDZB13	-94493.0935	24	Zn**Im**$_2$	-3937.212	**66.18**	**0.74**

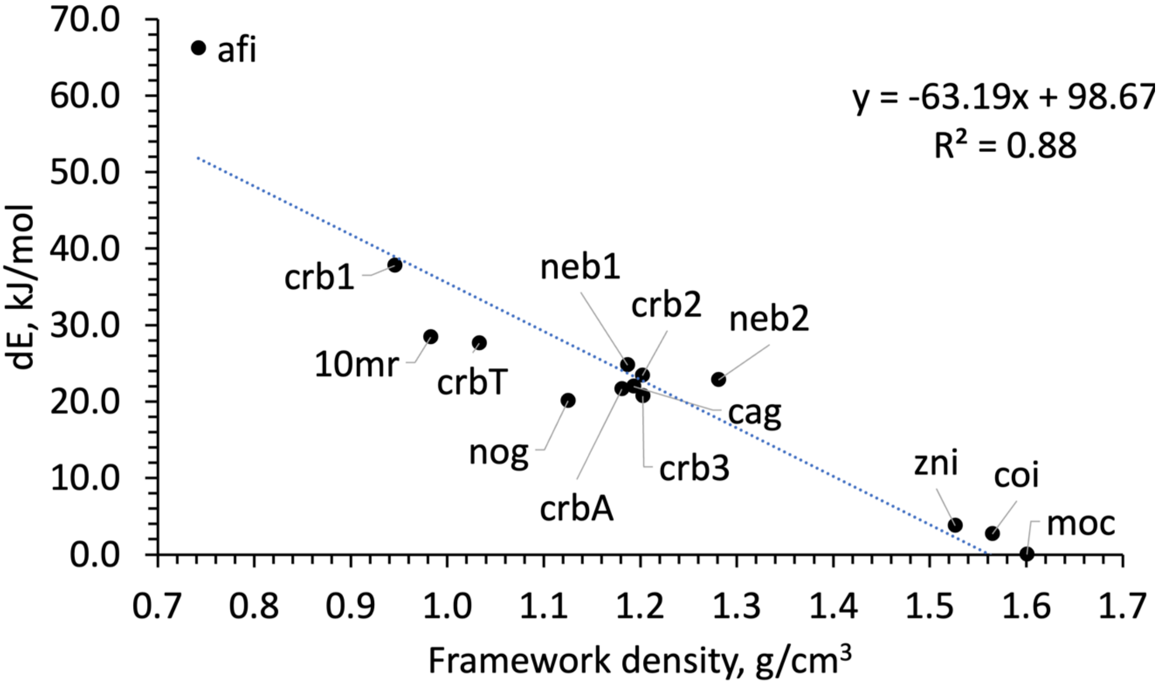

Figure S71. Dependance of the optimized relative enthalpy of different topology empty Zn**Im**$_2$ frameworks on their density. The reference point is the adjusted energy of **moc** topology framework, all enthalpies are scaled per zinc atom.

A representative input .cell file, on the example of the empty **moc**-Zn_4**Im**$_8$(H**Im**) structure:

```
#******************************************************************************
#*              Generated by cif2cell 1.2.10 2021-09-17 12:31                  *
#*  T. Bjorkman, Comp. Phys. Commun. 182, 1183-1186 (2011). Please cite generously.   *
#******************************************************************************

%BLOCK LATTICE_CART
ang
   9.816000000000001   0.000000000000000   0.000000000000000
  -4.123348931763962  12.467855372393482   0.000000000000000
  -0.161230369712944  -2.500241266699935  14.687845259811699
%ENDBLOCK LATTICE_CART

%BLOCK POSITIONS_FRAC
Zn   0.202270000000000   0.560370000000000   0.370480000000000
Zn   0.797730000000000   0.439630000000000   0.629520000000000
Zn   0.803100000000000   0.142280000000000   0.995070000000000
Zn   0.196900000000000   0.857720000000000   0.004930000000000
Zn   0.692540000000000   0.547910000000000   0.145520000000000
Zn   0.307460000000000   0.452090000000000   0.854480000000000
Zn   0.685920000000000   0.855620000000000   0.623690000000000
Zn   0.314080000000000   0.144380000000000   0.376310000000000
N    0.308300000000000   0.639300000000000   0.486700000000000
N    0.691700000000000   0.360700000000000   0.513300000000000
N    0.638600000000000   0.390200000000000   0.099200000000000
N    0.361400000000000   0.609800000000000   0.900800000000000
N    0.826400000000000   0.580800000000000   0.255800000000000
N    0.173600000000000   0.419200000000000   0.744200000000000
N    0.157000000000000   0.402200000000000   0.368700000000000
N    0.843000000000000   0.597800000000000   0.631300000000000
N    0.022500000000000   0.591700000000000   0.341500000000000
N    0.977500000000000   0.408300000000000   0.658500000000000
N    0.487800000000000   0.753100000000000   0.582200000000000
N    0.512200000000000   0.246900000000000   0.417800000000000
N    0.802900000000000   0.046900000000000   0.082800000000000
N    0.197100000000000   0.953100000000000   0.917200000000000
N    0.681800000000000   0.236200000000000   0.044600000000000
N    0.318200000000000   0.763800000000000   0.955400000000000
N    0.198600000000000   0.244900000000000   0.371400000000000
N    0.801400000000000   0.755100000000000   0.628600000000000
N    0.791500000000000   0.993100000000000   0.218800000000000
N    0.208500000000000   0.006900000000000   0.781200000000000
N    0.773700000000000   0.005800000000000   0.397500000000000
N    0.226300000000000   0.994200000000000   0.602500000000000
N    0.337900000000000   0.596200000000000   0.271000000000000
N    0.662100000000000   0.403800000000000   0.729000000000000
N    0.521700000000000   0.591300000000000   0.184300000000000
N    0.478300000000000   0.408700000000000   0.815700000000000
N    0.001900000000000   0.750100000000000   0.007500000000000
N    0.998100000000000   0.250000000000000   0.992500000000000
N    0.813900000000000   0.628400000000000   0.057100000000000
N    0.186100000000000   0.371600000000000   0.942900000000000
N    0.721600000000000   0.061500000000000   0.873400000000000
N    0.278400000000000   0.938500000000000   0.126600000000000
```

N	0.745900000000000	0.954300000000000	0.532100000000000
N	0.254100000000000	0.045700000000000	0.467900000000000
N	0.676200000000000	0.948900000000000	0.738300000000000
N	0.323800000000000	0.051100000000000	0.261700000000000
C	0.731500000000000	0.343600000000000	0.058700000000000
C	0.268500000000000	0.656400000000000	0.941300000000000
C	0.433200000000000	0.719900000000000	0.494400000000000
C	0.566800000000000	0.280100000000000	0.505600000000000
C	0.938700000000000	0.705900000000000	0.077900000000000
C	0.061300000000000	0.294100000000000	0.922100000000000
C	0.946500000000000	0.552900000000000	0.263000000000000
C	0.053500000000000	0.447100000000000	0.737000000000000
C	0.792900000000000	0.619700000000000	0.965400000000000
C	0.207100000000000	0.380300000000000	0.034600000000000
C	0.830500000000000	0.645900000000000	0.338700000000000
C	0.169500000000000	0.354100000000000	0.661300000000000
C	0.948000000000000	0.651500000000000	0.390000000000000
C	0.052000000000000	0.348500000000000	0.610000000000000
C	0.250600000000000	0.352100000000000	0.391800000000000
C	0.749400000000000	0.647900000000000	0.608200000000000
C	0.346900000000000	0.664300000000000	0.208900000000000
C	0.653100000000000	0.335700000000000	0.791100000000000
C	0.445700000000000	0.553800000000000	0.253600000000000
C	0.554300000000000	0.446200000000000	0.746400000000000
C	0.460500000000000	0.661800000000000	0.157700000000000
C	0.539500000000000	0.338200000000000	0.842300000000000
C	0.562900000000000	0.992000000000000	0.753900000000000
C	0.437100000000000	0.008000000000000	0.246100000000000
C	0.764400000000000	0.995500000000000	0.813000000000000
C	0.235600000000000	0.004500000000000	0.187000000000000
C	0.904700000000000	0.693900000000000	0.937200000000000
C	0.095300000000000	0.306100000000000	0.062800000000000
C	0.590400000000000	0.057100000000000	0.834600000000000
C	0.409600000000000	0.942900000000000	0.165400000000000
C	0.283400000000000	0.619800000000000	0.575100000000000
C	0.716600000000000	0.380200000000000	0.424900000000000
C	0.388900000000000	0.690100000000000	0.631800000000000
C	0.611100000000000	0.309900000000000	0.368200000000000
C	0.041400000000000	0.320400000000000	0.332100000000000
C	0.958600000000000	0.679600000000000	0.667900000000000
C	0.522200000000000	0.305500000000000	0.110100000000000
C	0.477800000000000	0.694500000000000	0.889900000000000
C	0.735400000000000	0.056900000000000	0.538000000000000
C	0.264600000000000	0.943100000000000	0.462000000000000
C	0.769000000000000	0.929800000000000	0.446700000000000
C	0.231000000000000	0.070200000000000	0.553300000000000
C	0.786600000000000	0.071500000000000	0.173800000000000
C	0.213400000000000	0.928500000000000	0.826200000000000
C	0.812800000000000	0.945000000000000	0.076800000000000
C	0.187200000000000	0.055000000000000	0.923200000000000
C	0.064600000000000	0.226700000000000	0.331600000000000
C	0.935400000000000	0.773300000000000	0.668400000000000
C	0.547200000000000	0.209600000000000	0.076900000000000
C	0.452800000000000	0.790400000000000	0.923100000000000
C	0.806100000000000	0.914400000000000	0.158000000000000

C	0.193900000000000	0.085600000000000	0.842000000000000
C	0.748900000000000	0.088200000000000	0.458900000000000
C	0.251100000000000	0.911800000000000	0.541100000000000
H	0.836885000000000	0.389735000000000	0.039496500000000
H	0.163115000000000	0.610265000000000	0.960503500000000
H	0.485328000000000	0.754925000000000	0.437118000000000
H	0.514672000000000	0.245075000000000	0.562882000000000
H	0.986776800000000	0.732102000000000	0.147266000000000
H	0.013223200000000	0.267898000000000	0.852734000000000
H	0.978584700000000	0.501963000000000	0.207964000000000
H	0.021415300000000	0.498037000000000	0.792036000000000
H	0.701639000000000	0.562765000000000	0.922639900000000
H	0.298361000000000	0.437235000000000	0.077360100000000
H	0.751656000000000	0.686201000000000	0.359436000000000
H	0.248344000000000	0.313799000000000	0.640564000000000
H	0.978312600000000	0.696442000000000	0.458935000000000
H	0.021687400000000	0.303558000000000	0.541065000000000
H	0.357626000000000	0.395660000000000	0.423885000000000
H	0.642374000000000	0.604340000000000	0.576115000000000
H	0.275761000000000	0.712000000000000	0.201634000000000
H	0.724239000000000	0.288000000000000	0.798366000000000
H	0.468512000000000	0.495776000000000	0.291386000000000
H	0.531488000000000	0.504224000000000	0.708614000000000
H	0.496547000000000	0.709199000000000	0.103630000000000
H	0.503453000000000	0.290801000000000	0.896370000000000
H	0.468646000000000	0.975257000000000	0.707885000000000
H	0.531354000000000	0.024743000000000	0.292115000000000
H	0.865588000000000	0.979807000000000	0.822955000000000
H	0.134412000000000	0.020193000000000	0.177045000000000
H	0.916064500000000	0.707491000000000	0.867021000000000
H	0.083935500000000	0.292509000000000	0.132979000000000
H	0.519999000000000	0.100440000000000	0.865525000000000
H	0.480001000000000	0.899560000000000	0.134475000000000
H	0.192187000000000	0.557040000000000	0.594888000000000
H	0.807813000000000	0.442960000000000	0.405112000000000
H	0.395340000000000	0.696305000000000	0.705563000000000
H	0.604660000000000	0.303695000000000	0.294437000000000
H	0.942337500000000	0.329544000000000	0.306426000000000
H	0.057662500000000	0.670456000000000	0.693574000000000
H	0.424189000000000	0.312175000000000	0.140311000000000
H	0.575811000000000	0.687825000000000	0.859689000000000
H	0.718192000000000	0.105480000000000	0.600394000000000
H	0.281808000000000	0.894520000000000	0.399606000000000
H	0.782246000000000	0.852201000000000	0.418099000000000
H	0.217754000000000	0.147799000000000	0.581901000000000
H	0.771139000000000	0.147163000000000	0.205775000000000
H	0.228861000000000	0.852837000000000	0.794225000000000
H	0.824623000000000	0.895015000000000	0.013704700000000
H	0.175377000000000	0.104985000000000	0.986295300000000
H	0.989005800000000	0.147127000000000	0.303750000000000
H	0.010994200000000	0.852873000000000	0.696250000000000
H	0.473707000000000	0.127798000000000	0.076548900000000
H	0.526293000000000	0.872202000000000	0.923451100000000
H	0.811840000000000	0.836733000000000	0.171706000000000
H	0.188160000000000	0.163267000000000	0.828294000000000

```
H    0.742000000000000   0.164920000000000   0.443580000000000
H    0.258000000000000   0.835080000000000   0.556420000000000
H    0.791329000000000   0.004450000000000   0.330392000000000
H    0.208671000000000   0.995550000000000   0.669608000000000
%ENDBLOCK POSITIONS_FRAC

#%BLOCK SPECIES_POT
# H   H_00.usp
# Zn  Zn_00.usp
# C   C_00.usp
# N   N_00.usp
#%ENDBLOCK SPECIES_POT

%BLOCK SYMMETRY_OPS
# Symm. op. 1
  1.000000000000000   0.000000000000000   0.000000000000000
  0.000000000000000   1.000000000000000   0.000000000000000
  0.000000000000000  -0.000000000000000   1.000000000000000
  0.000000000000000   0.000000000000000   0.000000000000000
# Symm. op. 2
 -1.000000000000000   0.000000000000000   0.000000000000000
  0.000000000000000  -1.000000000000000   0.000000000000000
 -0.000000000000000   0.000000000000000  -1.000000000000000
  0.000000000000000   0.000000000000000   0.000000000000000
%ENDBLOCK SYMMETRY_OPS
kpoint_mp_spacing : 0.06
snap_to_symmetry
```

A representative input .param file, on the example of the empty **moc**-Zn_4**Im**$_8$(H**Im**) structure:

```
TASK                    : geometryoptimization
XC_FUNCTIONAL           : PBE
SEDC_APPLY              : true
SEDC_SCHEME             : D3

CUT_OFF_ENERGY          : 800 eV
MAX_SCF_CYCLES          : 200
CALCULATE_STRESS        : true
GEOM_METHOD             : bfgs
MIXING_SCHEME           : pulay
MIX_CHARGE_AMP          : 0.4
ELEC_ENERGY_TOL         : 0.0000000001

FIX_OCCUPANCY           : false
WRITE_CELL_STRUCTURE    : true
GEOM_MAX_ITER           : 600
WRITE_CHECKPOINT        : all
WRITE_BIB               : false
OPT_STRATEGY            : speed
PAGE_WVFNS              : 0
NUM_DUMP_CYCLES         : 0
BACKUP_INTERVAL         : 1800
FINITE_BASIS_CORR       : 2
GEOM_FORCE_TOL          : 0.05
GEOM_STRESS_TOL         : 0.05
WRITE_CIF_STRUCTURE     : true
CONTINUATION            : default
```

2.9.3. Periodic DFT optimization of selected guest-filled zinc imidazolate forms

DFT calculations of framework template stabilization were performed in the following way:

1) Perform periodic DFT optimization on empty ZIF framework (Section SI-1.6)
2) Insert guest inside ZIF framework, optimize, and calculate the total enthalpy of ZIF + guest complex
3) Calculate the enthalpy of the gas phase guest (modeled as a molecule in a large box)
4) Subtract the guest energy (proportional to the number of guest molecules inside the framework) to get the stabilized enthalpy of pure Zn**Im**$_2$ (equation (1)).
5) Steps 1-4 give the stabilization enthalpy of inserting the guest in from **gas phase**. To approximate the enthalpy of liquid guest insertion, experimental (literature) evaporation enthalpies were added (equation (2)).

$E_{\text{stabil from gas}} = [E(x\text{guest@}y\text{Zn}\mathbf{Im}_2) - xE(\text{guest}) - yE(\text{empty Zn}\mathbf{Im}_2)]/y$ (1)

$E_{\text{stabil from liquid}} = E_{\text{stabil from gas}} + x/y\ E_{\text{vap}}(\text{guest})$. (2)

Enthalpy values obtained through equations (1) and (2) were compared to:

a) **moc**-Zn**Im**$_2$, the global minimum of the empty structures. This gives the overall stabilization enthalpy of inserting the guest from gas phase (Δ_{moc}(g)) and from the liquid phase (Δ_{moc}(l)), and

b) to the corresponding empty Zn**Im**$_2$ topologies. This gives the stabilization enthalpy of inserting the guest into the empty framework from the gas phase and the liquid phase (Δ_{empty}(g) and Δ_{empty}(l)).

Table S14. Results of periodic DFT optimization of single guest molecules in a large box – gas phase simulation

guest	abbreviation	Enthalpy; eV	E_{vap} (kJ/mol)
cyclohexane	cHANE	-1140.377	32.2
N,N-dibutylformamide	DBF	-2442.409	48.4
N,N-diethylformamide	DEF	-1682.042	41.4
N,N-dimethylacetamide	DMA	-1491.979	67.9
N,N-dimethylformamide	DMF	-1301.783	43.6
pyridine	PYR	-1144.693	35.1
tetrahydrofuran	THF	-1198.567	32.2
chloroform	$CHCl_3$	-1441.229	29.4
toluene	PhMe	-1232.465	38.0
acetophenone	AcPhe	-1828.986	52.6

The input crystal structures of the guest-filled zinc imidazolate topologies were either taken directly from experiment (CSD database or in-house) or modified from experimental structures.

5 structures were taken from the CSD (DBF@**10mr** – GOQSIQ, PYR@**neb2** – KEVLEE, DMA@**crb3** – VEJYEP, DMF@**cag** – VEJYUF01, DMF@**nog** – HIFWAV), and 6 structures were taken from

collected SCXRD (THF@**cag**, DEF@**cag** and $CHCl_3$@**cag**), and PXRD (PhMe@**crbT**, AcPhe@**crbA**, cHANE@**neb1**) data.

DMF@**crb3** and DEF@**crb3** were modified from VEJYEP. cHANE@**cag** was modified from the SCXRD structure of THF@**cag**. DMF@**nog** was modified from HIFWAV. PhH@**neb1** was modified from the PXRD structure of cHANE@**neb1**. PhH@**neb2** was modified from KEVLEE.

Table S15. Results of periodic DFT optimization of guest-filled zinc imidazolate topologies

	# guests	# Zn	enthalpy	E/Zn	density	Δ_{moc}(g)	Δ_{moc}(**l**)	Δ_{empty}(g)	Δ_{empty}(l)
	in unit cell		eV	eV	g/cm^3	kJ/mol			
DEF@**cag**	8	16	-76470.1365	-3938.3625	1.550	-44.83	**-24.13**	-66.78	-46.08
DBF@**10mr**	20	40	-206383.8940	-3938.3930	1.368	-47.78	**-23.58**	-76.16	-51.96
PhMe@**crbT**	6	8	-38901.9621	-3938.3968	1.413	-48.14	**-19.64**	-75.74	-47.24
PYR@**neb2**	2	4	-18042.3775	-3938.2478	1.535	-33.77	**-16.23**	-56.58	-39.03
DEF@**crb3**	4	8	-38234.1948	-3938.2533	1.472	-34.30	**-13.60**	-54.98	-34.28
cHANE@**cag**	8	16	-72134.2004	-3938.1988	1.453	-29.04	**-12.94**	-51.00	-34.90
HPh@**neb2**	4	2	-17837.2732	-3938.1814	1.512	-27.37	**-11.95**	-50.17	-34.75
DMF@**cag**	8	16	-73426.1228	-3938.2412	1.499	-33.13	**-11.33**	-55.08	-33.28
cHANE@**neb1**	2	4	-18033.4441	-3938.1723	1.448	-26.49	**-10.39**	-51.25	-35.15
THF@**cag**	8	16	-72599.3205	-3938.1740	1.471	-26.65	**-10.57**	-48.60	-32.52
DMF@**crb3**	4	8	-36712.7894	-3938.2072	1.469	-29.85	**-8.05**	-50.35	-28.73
HPh@**neb1**	4	2	-17837.0902	-3938.1357	1.444	-22.95	**-7.53**	-47.72	-32.30
AcPhe@**crbA**	16	8	-77643.8156	-3938.2455	1.529	-33.54	**-7.24**	-54.22	-20.27
$CHCl_3$@**cag**	8	16	-74539.5929	-3938.1099	1.632	-20.46	**-5.76**	-42.41	-27.71
DMA@**crb3**	4	8	-37474.2317	-3938.2897	1.496	-37.81	**-3.86**	-58.49	-24.54
DEF@**nog**	4	20	-85487.3041	-3937.9568	1.249	-5.69	**2.59**	-25.75	-17.47
DMF@**nog**	4	20	-83964.9504	-3937.8909	1.220	0.66	**9.38**	-19.40	-10.68

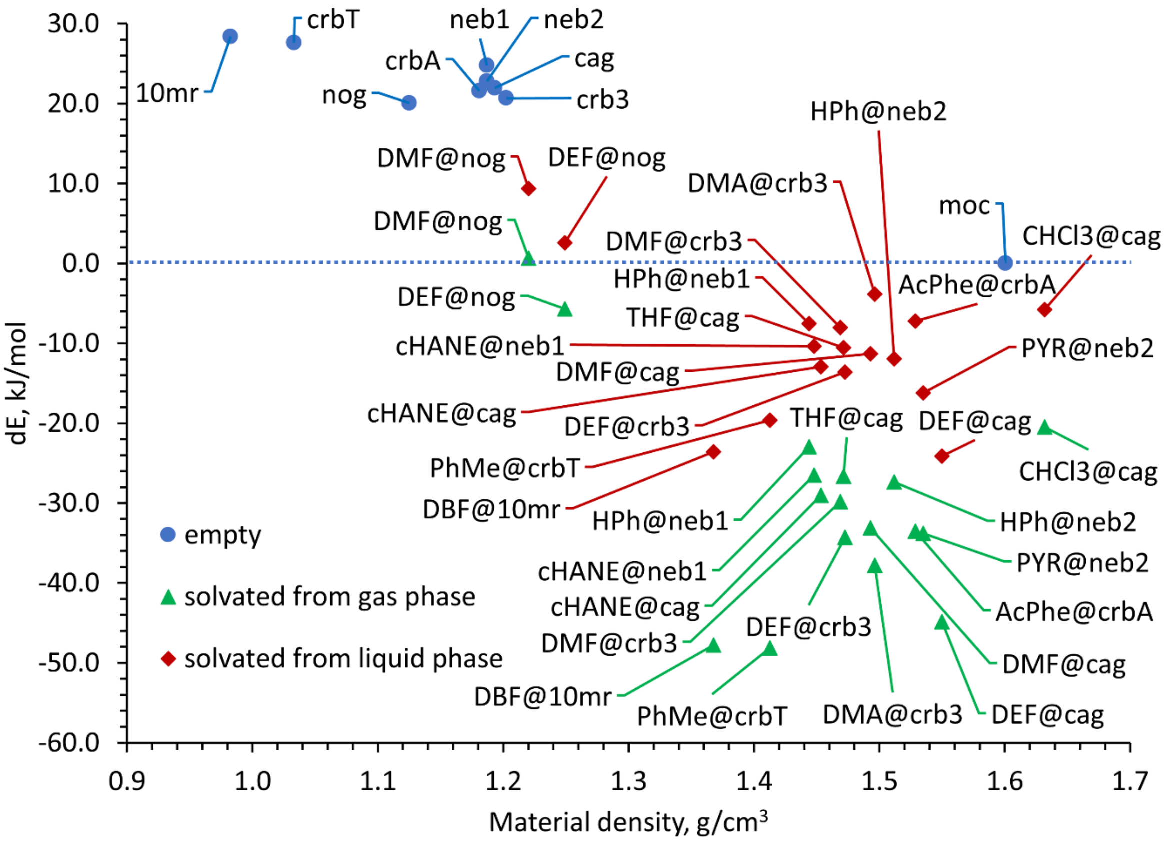

Figure S72. Dependance of the optimized relative enthalpy of different topology Zn**Im**$_2$ frameworks on their density. The reference point is the adjusted energy of **moc** topology framework (blue dotted line), and all enthalpies are scaled per zinc atom. The enthalpies of empty frameworks are plotted in blue circles and labeled by their topology. The guest-filled frameworks are labeled as "guest@topology". Enthalpies of frameworks with guests inserted from the gas phase are labeled in red rhombuses, while those with guests inserted from the liquid phase are labeled with green triangles.

*2.9.4. Comparison of DFT and experimental structures for different topologies of Zn**Im**$_2$*

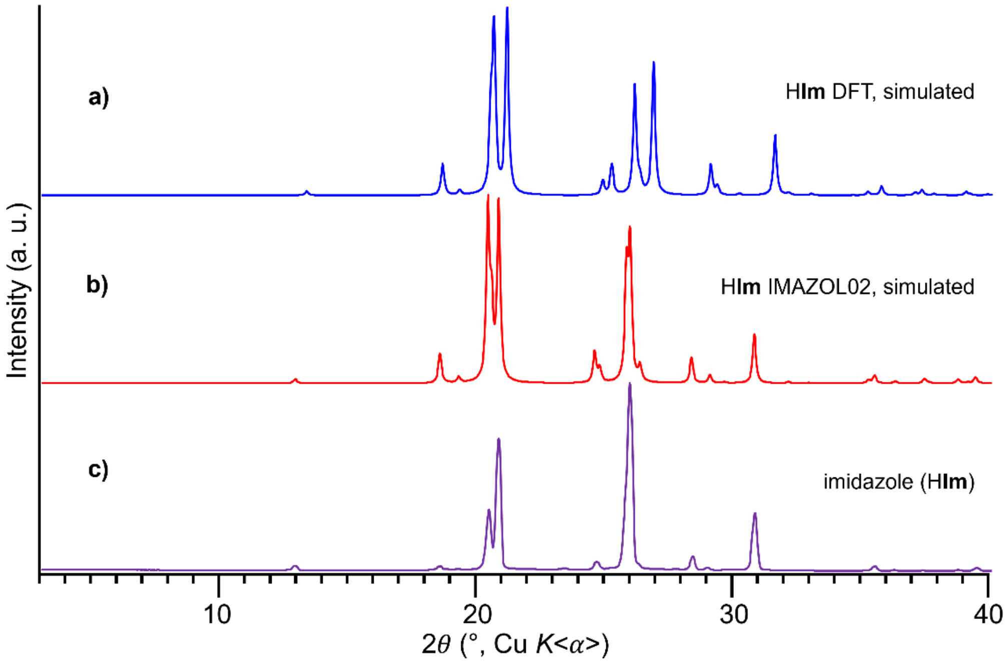

Figure S73. Simulated PXRD patterns of a) DFT optimized imidazole (H**Im**), b) experimental H**im** (CSD code IMAZOL02). c) PXRD pattern of H**Im** starting material.

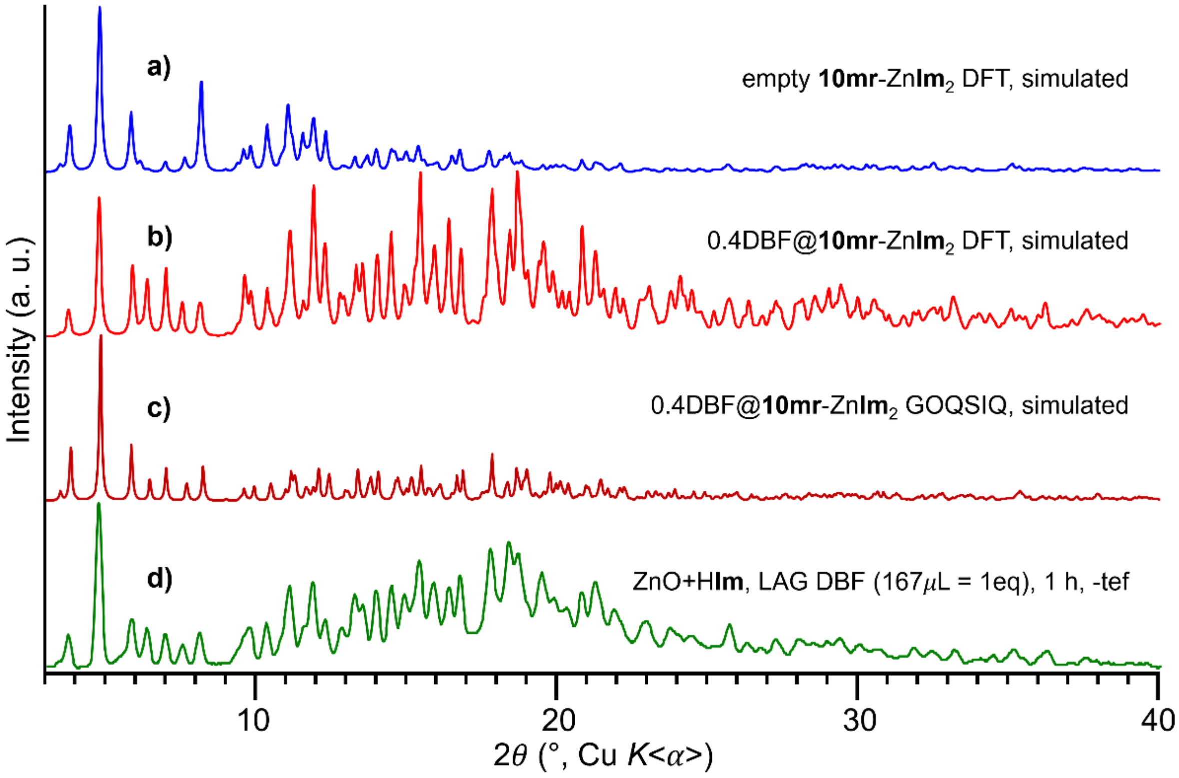

Figure S74. Simulated PXRD patterns of a) DFT optimized empty **10mr**-Zn**Im**$_2$, b) DFT optimized 0.4DBF@**10mr**-Zn**Im**$_2$ and c) experimental 0.4DBF@**10mr**-Zn**Im**$_2$ (CSD code GOQSIQ). d) PXRD pattern of the product of milling ZnO and H**Im** with 200 µL of DBF in a Teflon™ jar for 60 min.

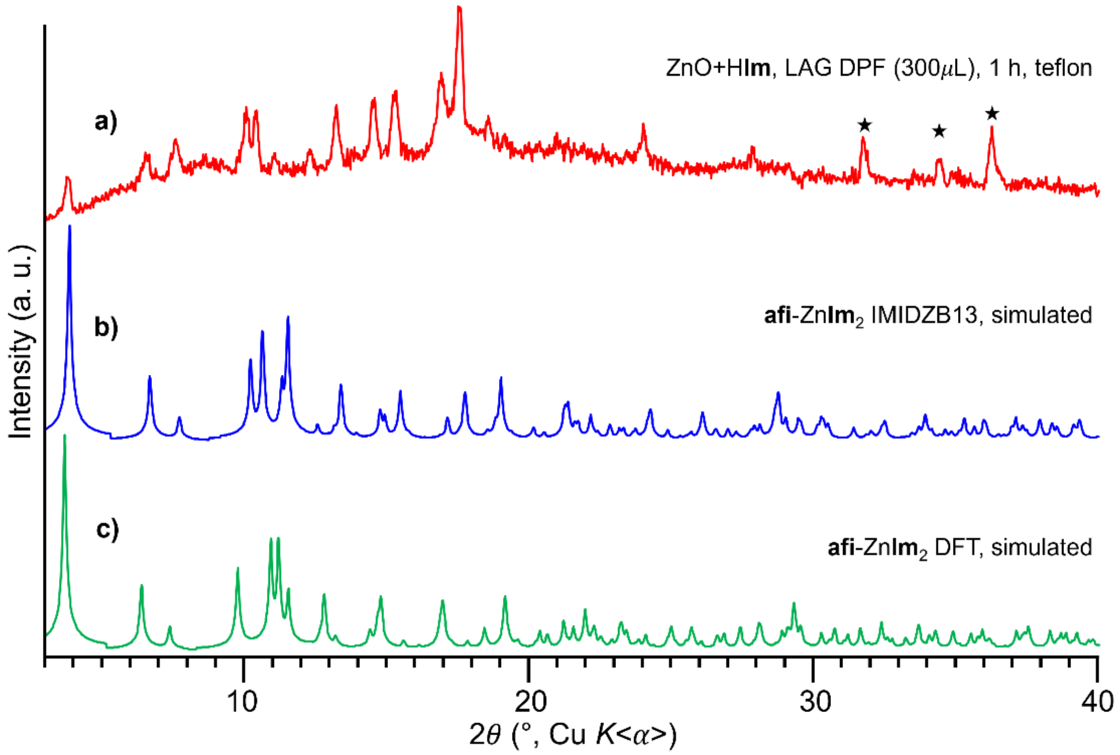

Figure S75. a) PXRD pattern of the product of milling ZnO and H**Im** with DPF in a Teflon™ jar for 60 min. Simulated PXRD patterns (plotted as the square root of intensity on the y axis, for easier peak identification) of b) **afi**-Zn**Im**$_2$ (CSD code IMIDZB13) and c) DFT optimized **afi**-Zn**Im**$_2$ (CSD code IMIDZB13). Black stars denote peaks of leftover ZnO reagent.

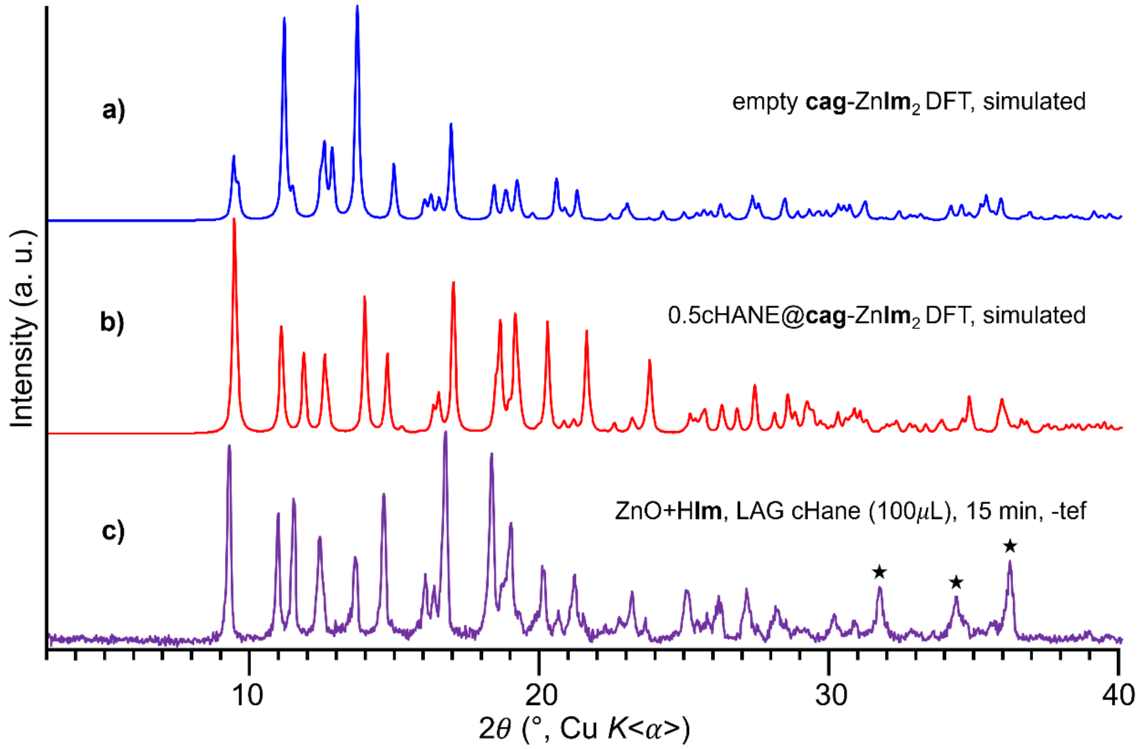

Figure S76. Simulated PXRD patterns of a) DFT optimized empty **cag**-Zn**Im**$_2$, b) DFT optimized 0.5cHANE@**cag**-Zn**Im**$_2$. c) PXRD pattern of the product of milling ZnO and H**Im** with 100 µL of cHANE in a Teflon™ jar for 15 min. Black stars denote peaks of leftover ZnO reagent.

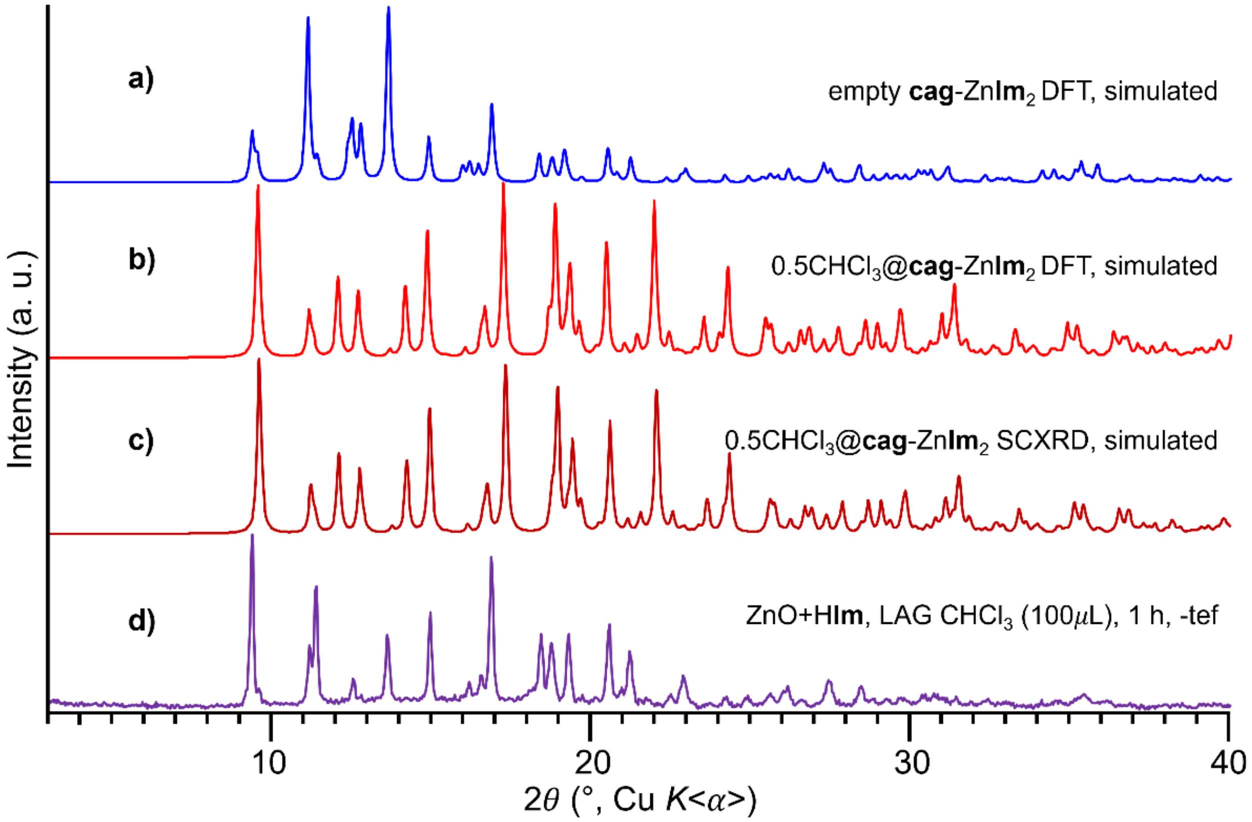

Figure S77. Simulated PXRD patterns of a) DFT optimized empty **cag**-Zn**Im**$_2$, b) DFT optimized 0.5$CHCl_3$@**cag**-Zn**Im**$_2$, c) experimental 0.5$CHCl_3$@**cag**-Zn**Im**$_2$ (solved from SCXRD). d) PXRD pattern of the product of milling ZnO and H**Im** with 100 µL of $CHCl_3$ in a Teflon™ jar for 60 min.

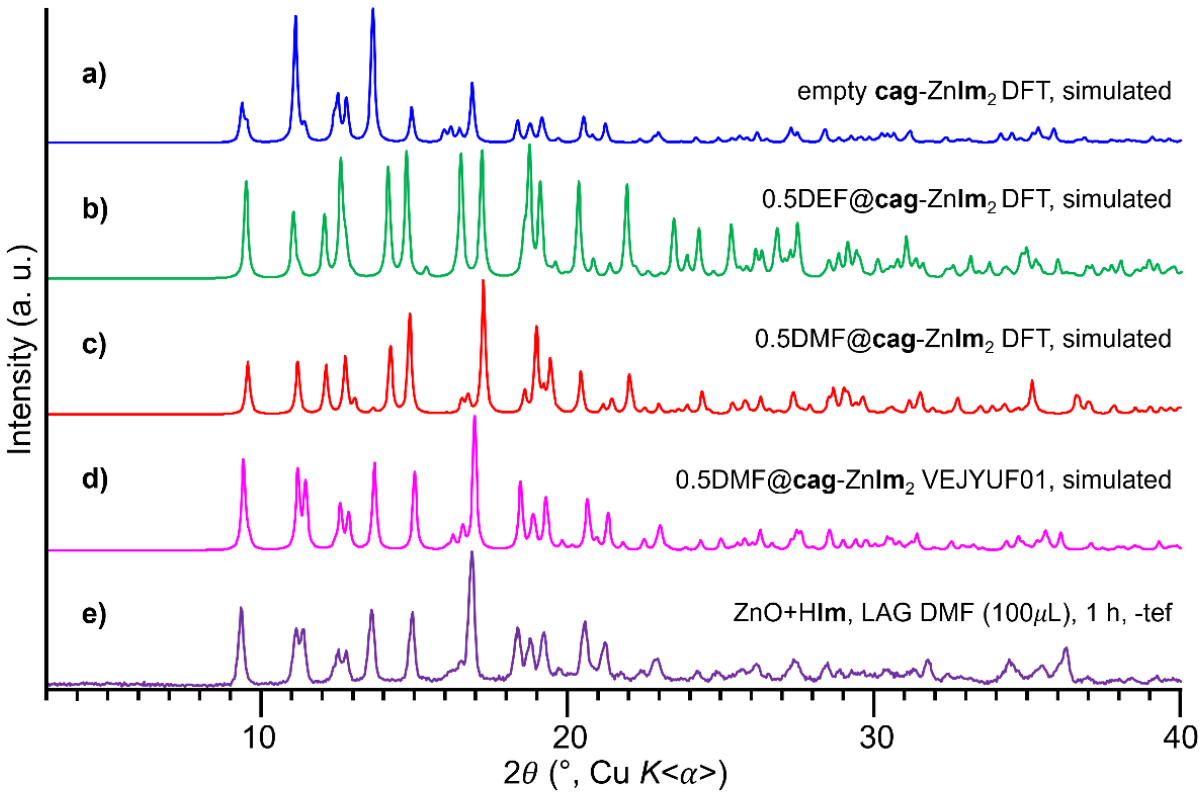

Figure S78. Simulated PXRD patterns of a) DFT optimized empty **cag**-Zn**Im**$_2$, b) DFT optimized 0.5DEF@**cag**-Zn**Im**$_2$, c) DFT optimized 0.5DMF@**cag**-Zn**Im**$_2$, d) experimental 0.5DMF@**cag**-Zn**Im**$_2$ (CSD code VEJYUF01). e) PXRD pattern of the product of milling ZnO and H**Im** with 100 μL of DMF in a Teflon™ jar for 60 min.

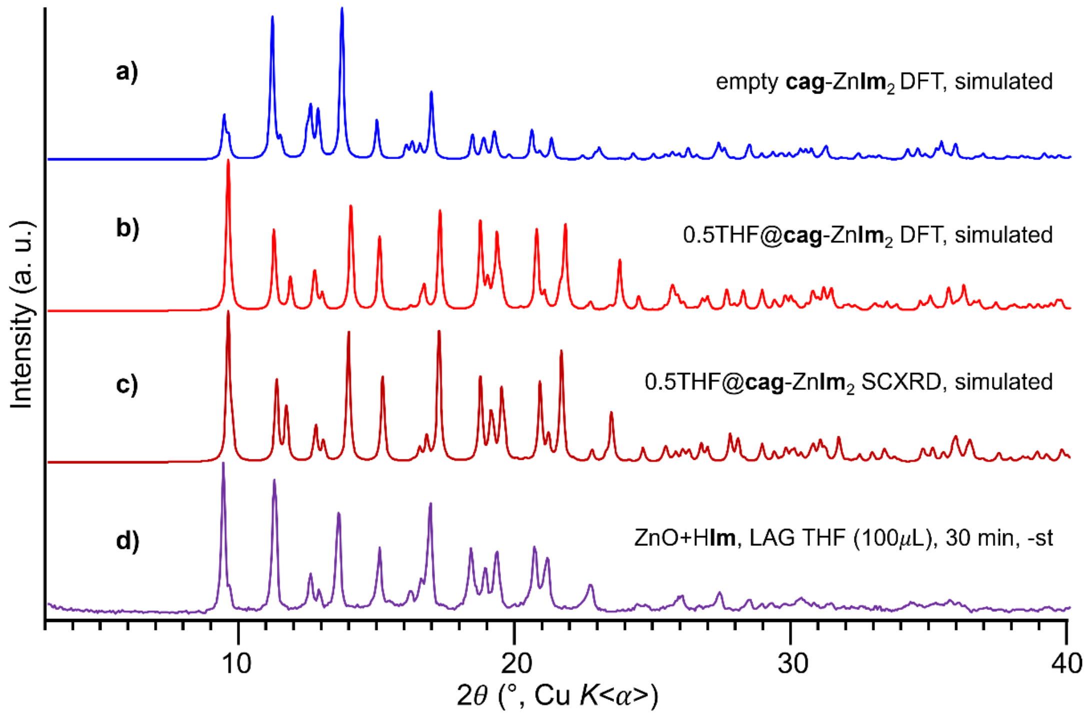

Figure S79. Simulated PXRD patterns of a) DFT optimized empty **cag**-Zn**Im**$_2$, b) DFT optimized 0.5THF@**cag**-Zn**Im**$_2$, c) experimental 0.5THF@**cag**-Zn**Im**$_2$ (solved from SCXRD). d) PXRD pattern of the product of milling ZnO and H**Im** with 100 μL of THF in a steel jar for 30 min.

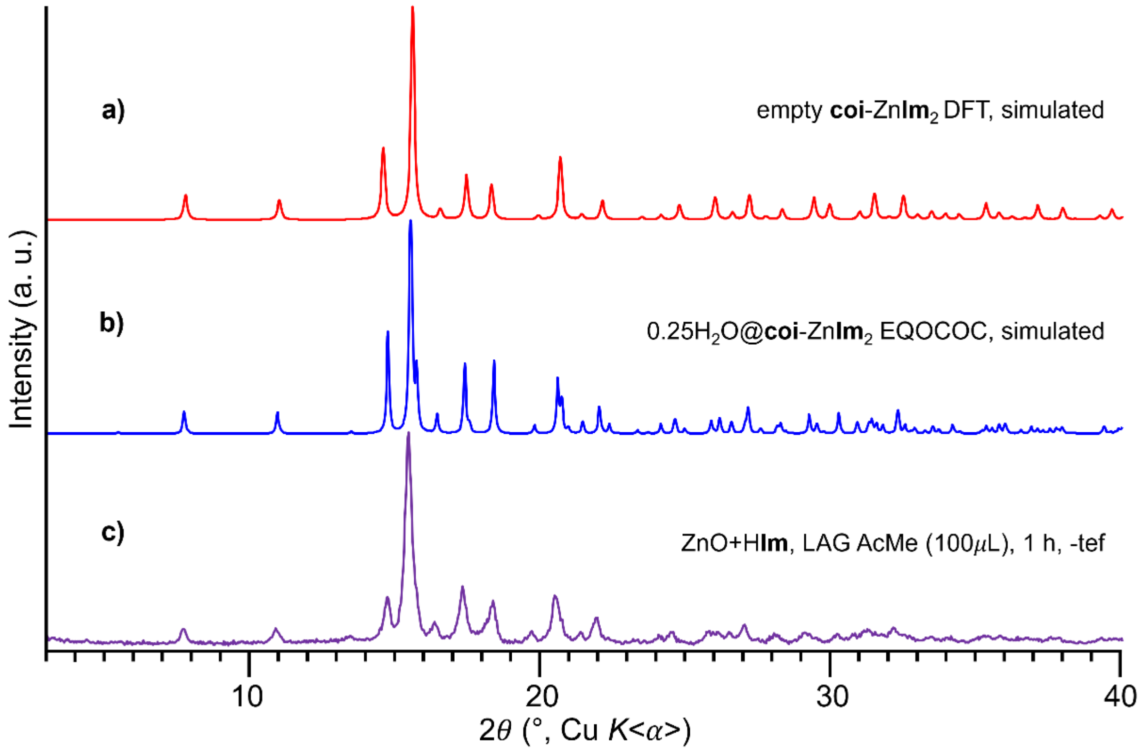

Figure S80. Simulated PXRD patterns of a) DFT optimized empty **coi**-Zn**Im**$_2$, b) experimental 0.25H_2O@**coi**-Zn**Im**$_2$ (CSD code EQOCOC). c) PXRD pattern of the product of milling ZnO and H**Im** with 100 µL of AcMe in a Teflon™ jar for 60 min.

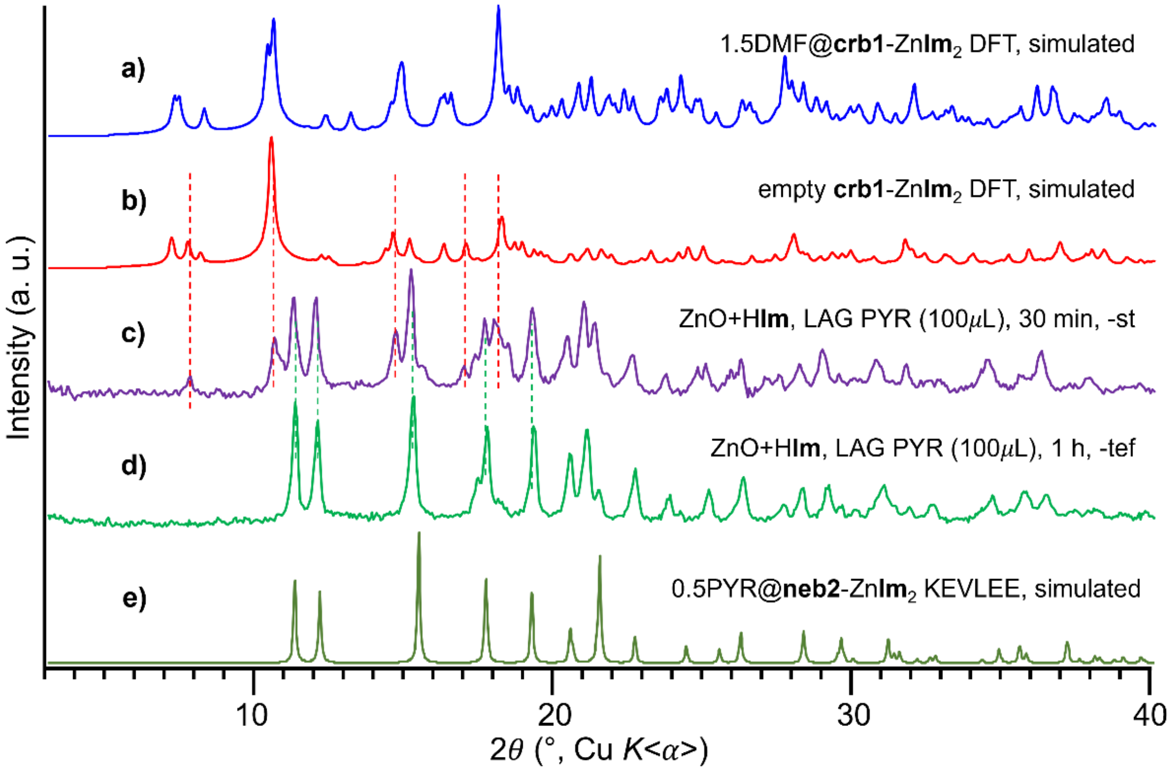

Figure S81. Simulated PXRD patterns of a) DFT optimized 1.5DMF@**crb1**-Zn**Im**$_2$, b) DFT optimized empty **crb1**-Zn**Im**$_2$, and e) experimental 0.5PYR@**neb2**-Zn**Im**$_2$ (CSD code KEVLEE). PXRD patterns of c) the product of milling ZnO and H**Im** with 100 µL of PYR in a steel jar for 30 min and d) PXRD pattern of the product of milling ZnO and H**Im** with 100 µL of PYR in a Teflon™ jar for 60 min. Red dashed lines indicate **crb1** phase peaks present in pattern c), while green dashed lines indicate **neb2** phase peaks present in pattern c).

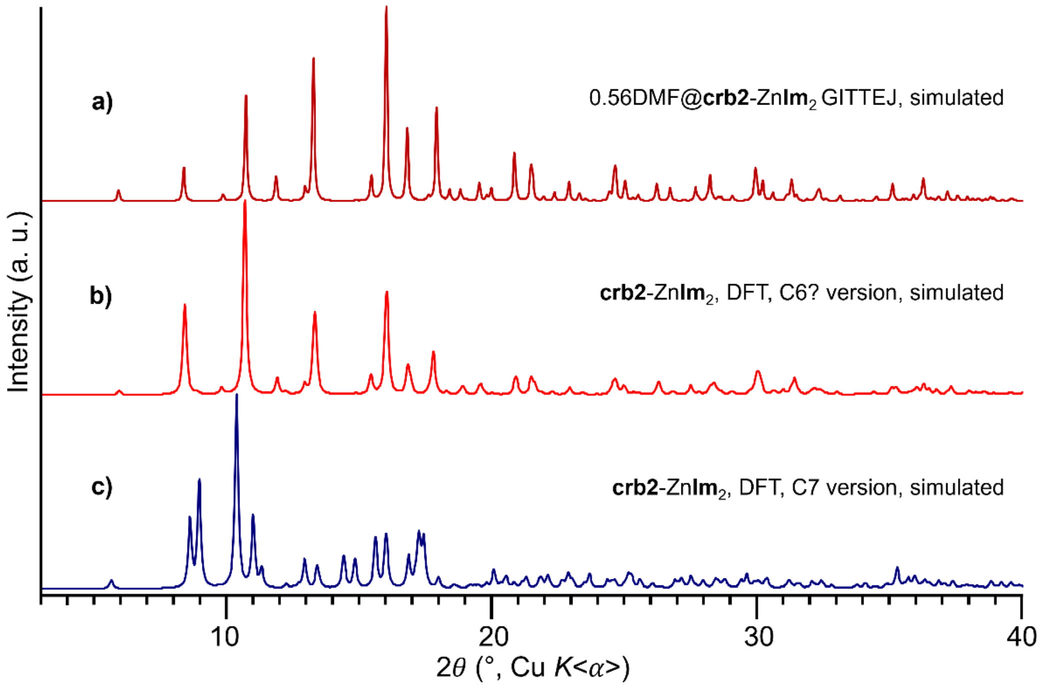

Figure S82. Simulated PXRD patterns of a) experimental 0.56DMF@**crb2**-Zn**Im**$_2$ (CSD code GITTEJ); b) DFT optimized empty **crb2**-Zn**Im**$_2$, C6? version; and c) DFT optimized empty **crb2**-Zn**Im**$_2$, C7 version.

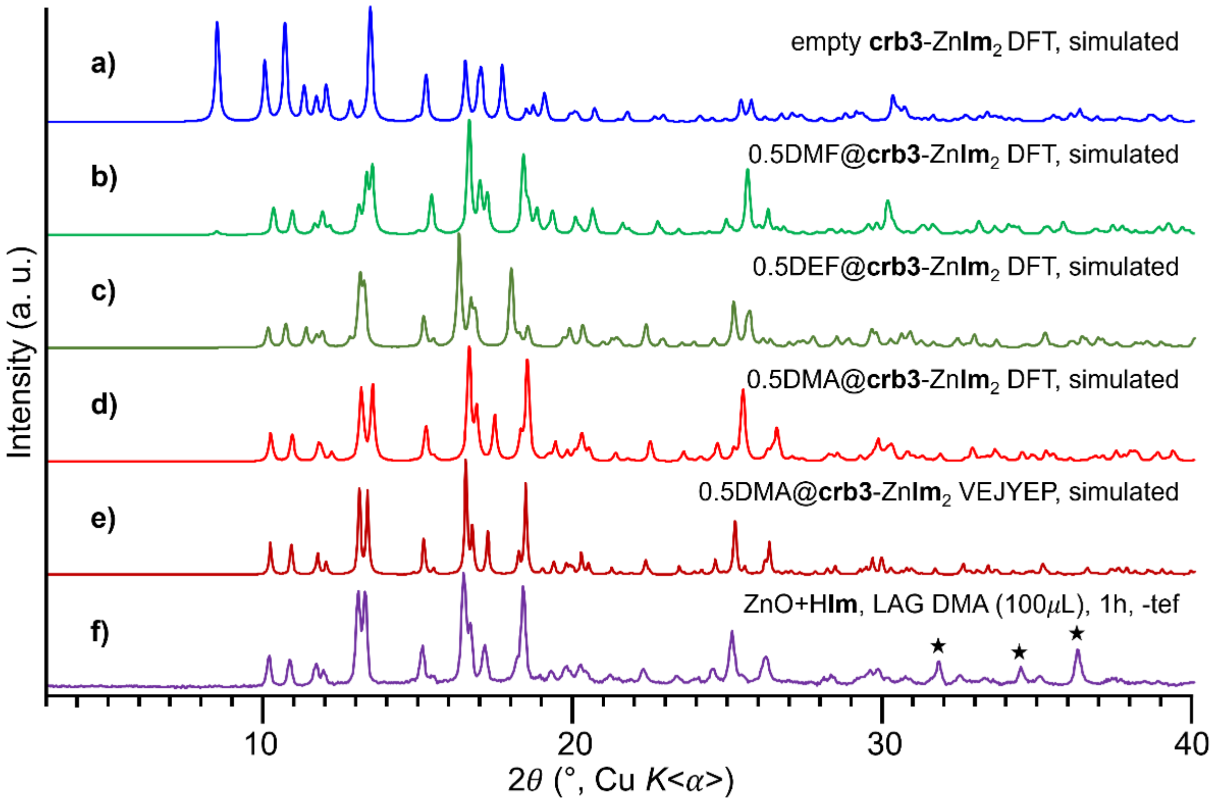

Figure S83. Simulated PXRD patterns of a) DFT optimized empty **crb3**-Zn**Im**$_2$, b) DFT optimized 0.5DMF@**crb3**-Zn**Im**$_2$, c) DFT optimized 0.5DEF@**crb3**-Zn**Im**$_2$, d) DFT optimized 0.5DMA@**crb3**-Zn**Im**$_2$, and e) experimental 0.5DMA@**crb3**-Zn**Im**$_2$ (CSD code VEJYEP). f) PXRD pattern of the product of milling ZnO and H**Im** with 100 µL of DMA in a Teflon™ jar for 60 min. Black stars denote peaks of leftover ZnO reagent.

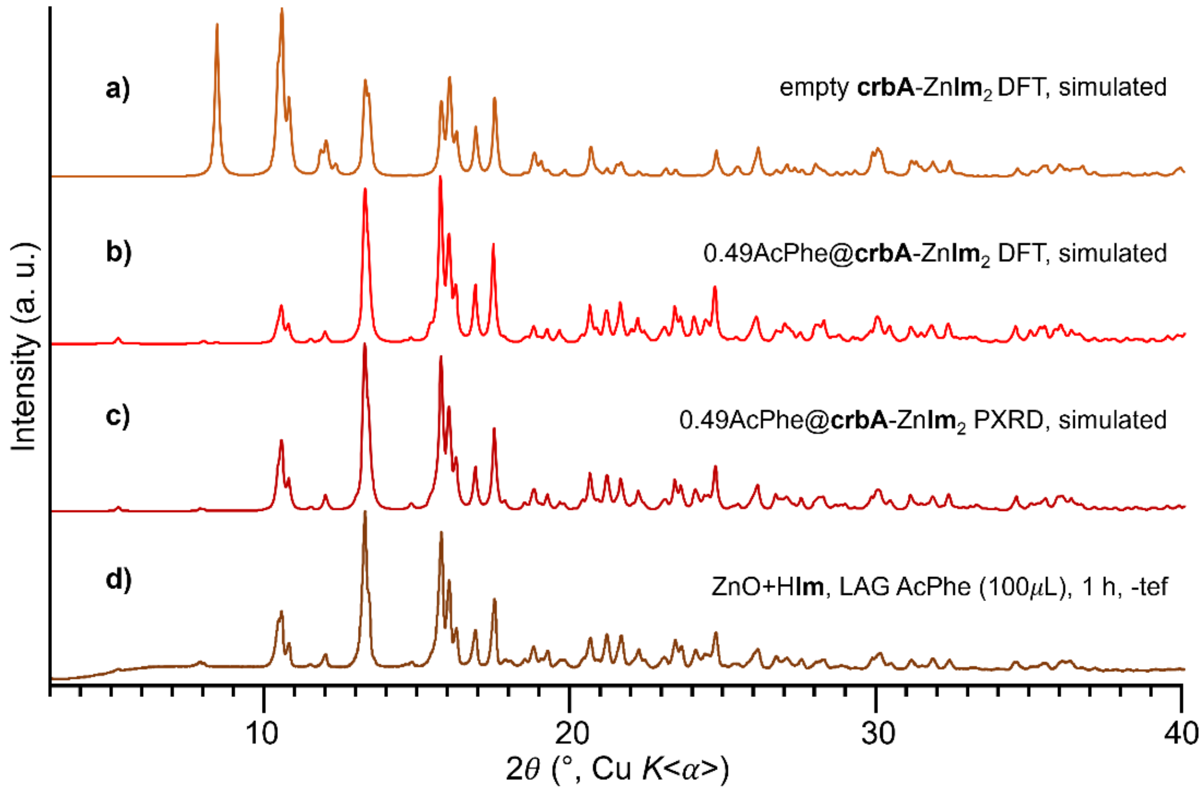

Figure S84. Simulated PXRD patterns of a) DFT optimized empty **crbA**-Zn**Im**$_2$, b) DFT optimized 0.49AcPhe@**crbA**-Zn**Im**$_2$, and c) experimental 0.49AcPhe@**crbA**-Zn**Im**$_2$ (solved from PXRD). d) PXRD pattern of the product of milling ZnO and H**Im** with 100 µL of AcPhe in a Teflon™ jar for 60 min.

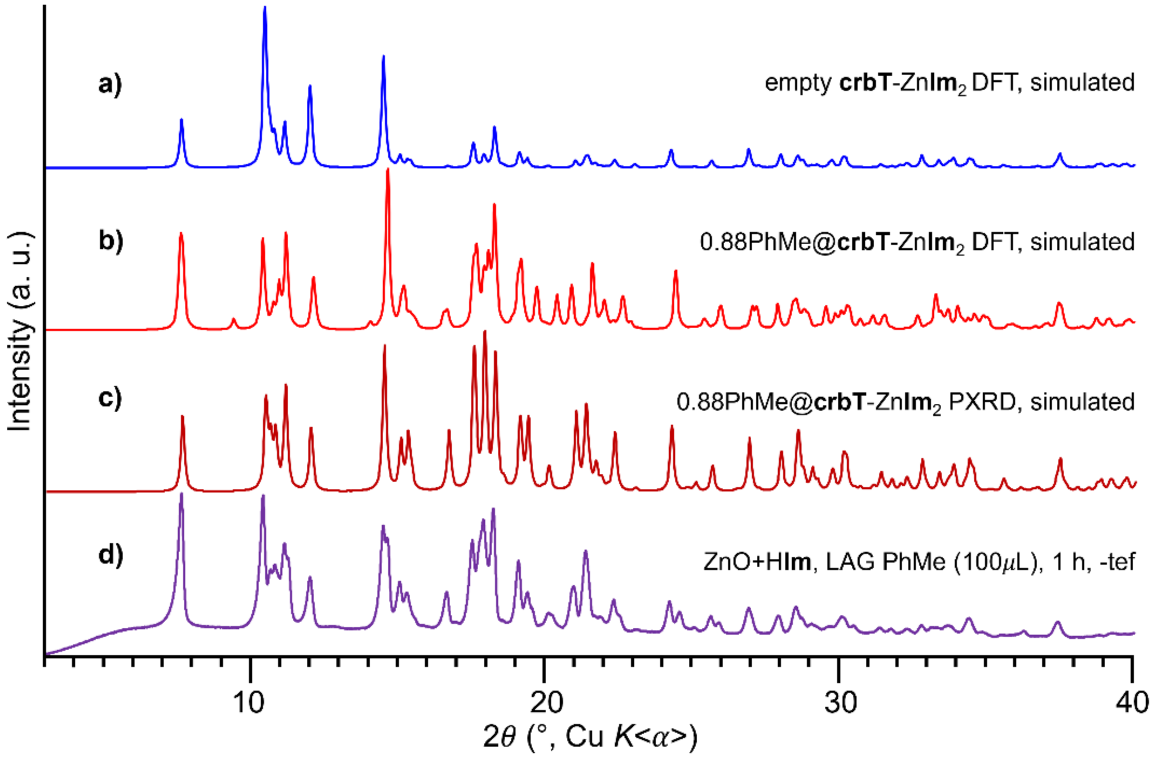

Figure S85. Simulated PXRD patterns of a) DFT optimized empty **crbT**-Zn**Im**$_2$, b) DFT optimized 0.88PhMe@**crbT**-Zn**Im**$_2$, and c) experimental 0.88PhMe@**crbT**-Zn**Im**$_2$ (solved from PXRD). d) PXRD pattern of the product of milling ZnO and H**Im** with 100 µL of PhMe in a Teflon™ jar for 60 min.

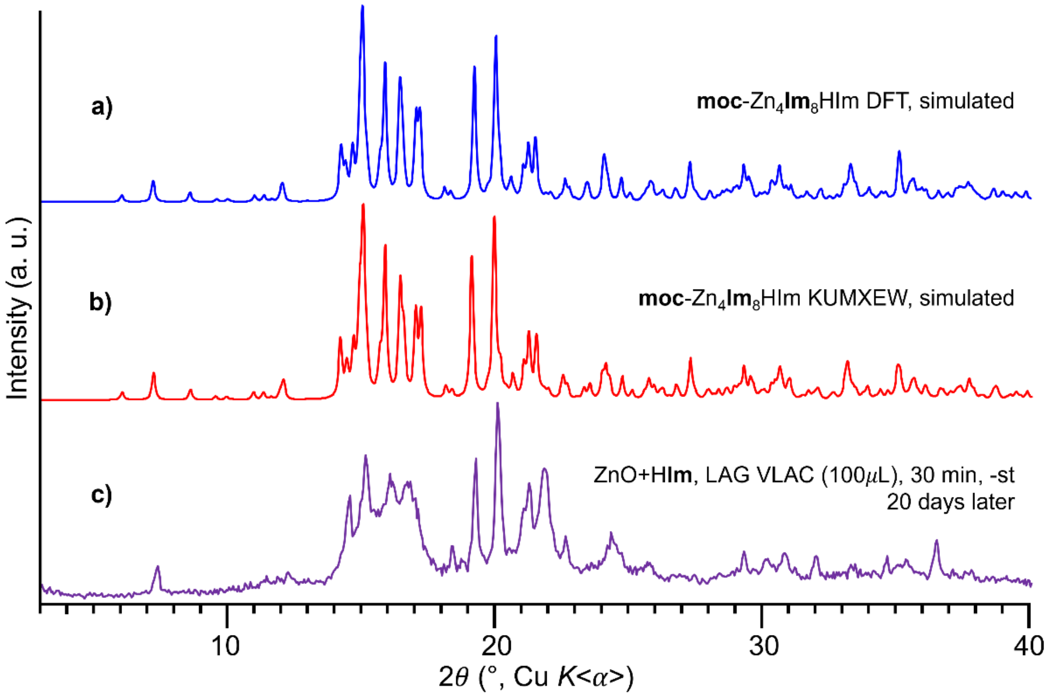

Figure S86. Simulated PXRD patterns of a) DFT optimized **moc**-Zn_4**Im**$_8$H**Im**, b) experimental **moc**-Zn_4**Im**$_8$H**Im** (CSD code KUMXEW). c) PXRD pattern of the product of milling ZnO and H**Im** with 100 µL of VLAC in a steel jar for 30 min, after 20 days standing at ambient conditions.

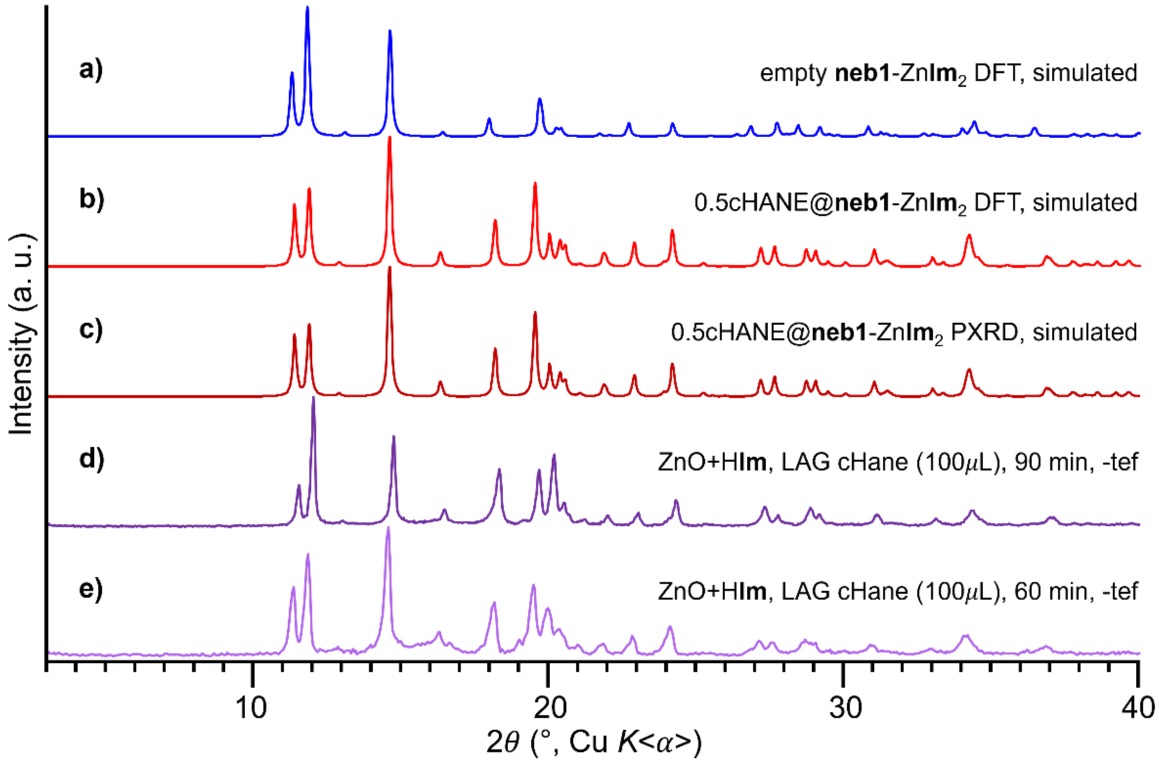

Figure S87. Simulated PXRD patterns of a) DFT optimized empty **neb1**-Zn**Im**$_2$, b) DFT optimized 0.5cHANE@**neb1**-Zn**Im**$_2$, c) experimental 0.5cHANE@**neb1**-Zn**Im**$_2$ (CSD code KEVLEE). PXRD patterns of the products of milling ZnO and H**Im** with 100 µL of cHANE in a Teflon™ jar for d) 90 min, and e) 60 min.

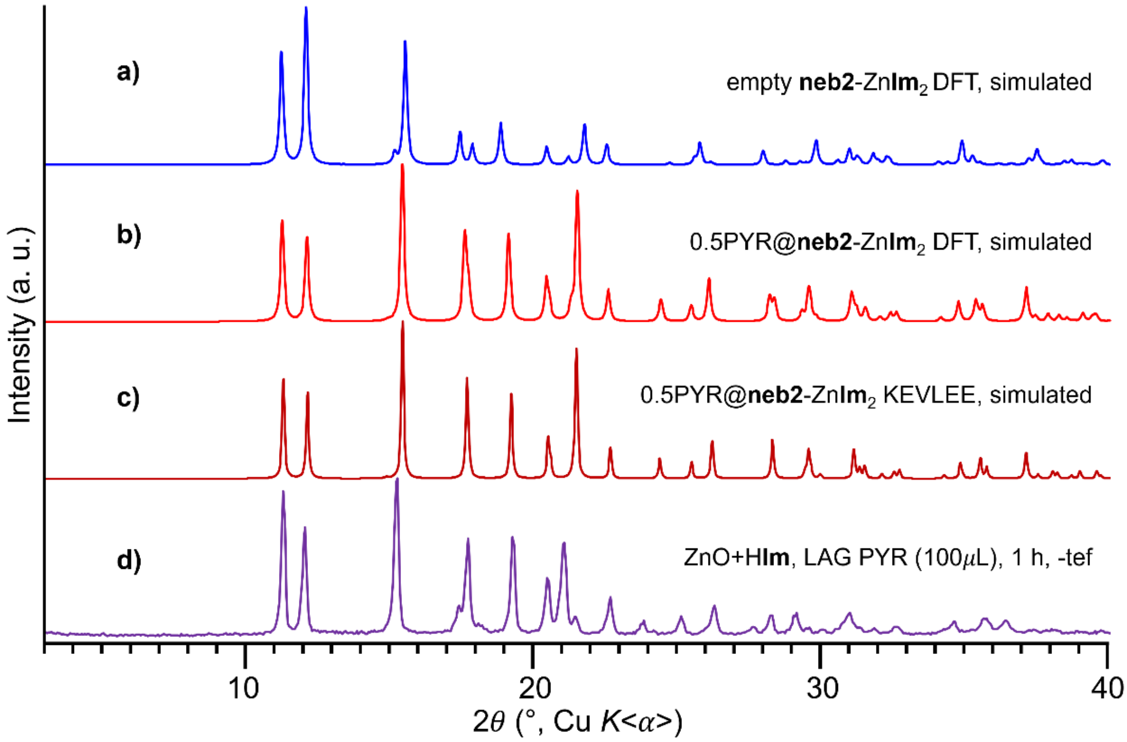

Figure S88. Simulated PXRD patterns of a) DFT optimized empty **neb2**-Zn**Im**$_2$, b) DFT optimized 0.5PYR@**neb2**-Zn**Im**$_2$, c) experimental 0.5PYR@**neb2**-Zn**Im**$_2$ (CSD code KEVLEE). d) PXRD pattern of the product of milling ZnO and H**Im** with 100 µL of PYR in a Teflon™ jar for 60 min.

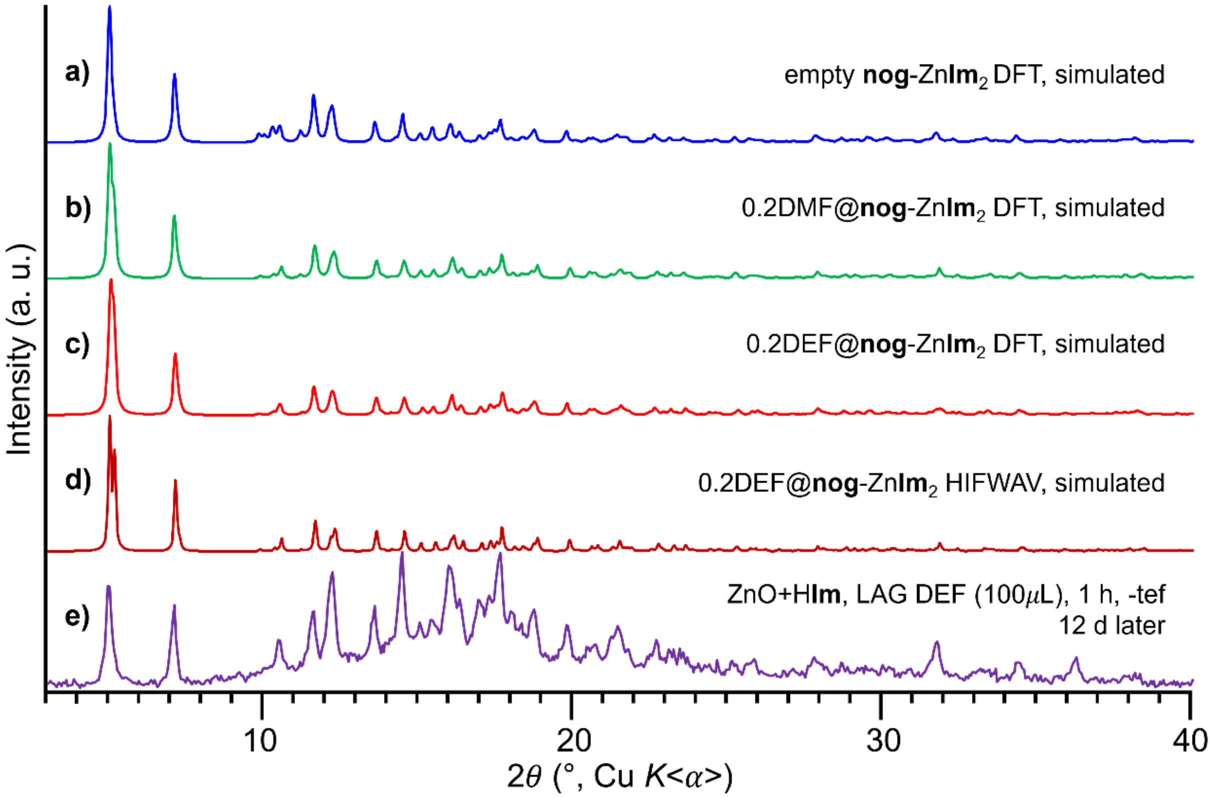

Figure S89. Simulated PXRD patterns of a) DFT optimized empty **nog**-Zn**Im**$_2$, b) DFT optimized 0.2DMF@**neb1**-Zn**Im**$_2$, b) DFT optimized 0.2DEF@**neb1**-Zn**Im**$_2$, and d) experimental 0.5DEF@**nog**-Zn**Im**$_2$ (CSD code HIFWAV). PXRD pattern of the product of milling ZnO and H**Im** with 100 µL of DEF in a Teflon™ jar for 60 min, after 12 days standing at ambient conditions.

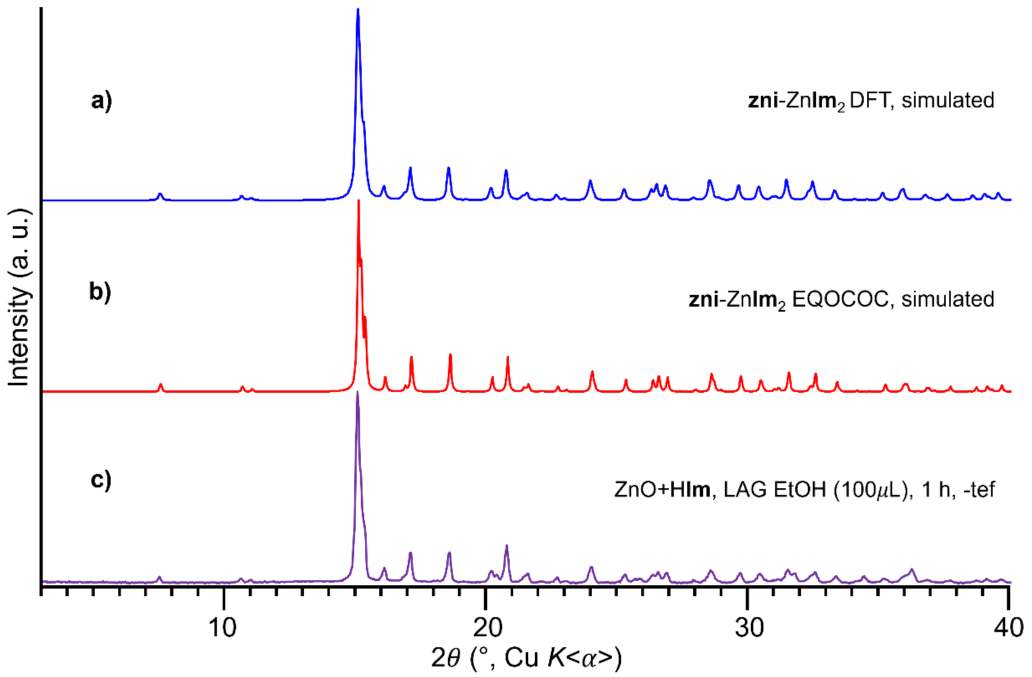

Figure S90. Simulated PXRD patterns of a) DFT optimized **zni**-Zn**Im**$_2$, b) experimental **zni**-Zn**Im**$_2$ (CSD code EQOCOC). c) PXRD pattern of the product of milling ZnO and H**Im** with 100 µL of EtOH in a Teflon™ jar for 60 min.

2.9.5. Images of selected geometry optimized crystal structures

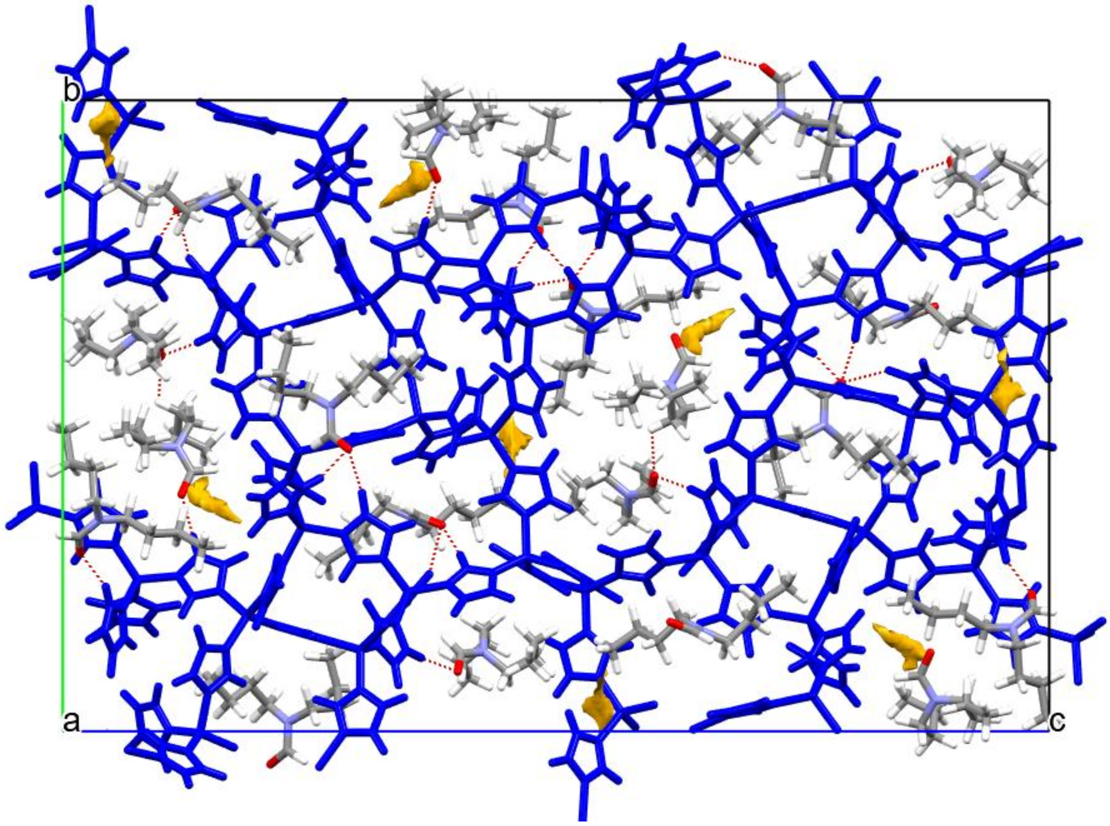

Figure S91. Geometry optimized structure of 0.4DBF@**10mr**-Zn**Im**$_2$. The Zn**Im**$_2$ parent framework is displayed in blue, and the DBF guests in CPK. Leftover voids in the structure are shown in yellow (solvent accessible surface), while the C-H···O bonds are shown dashed in red.

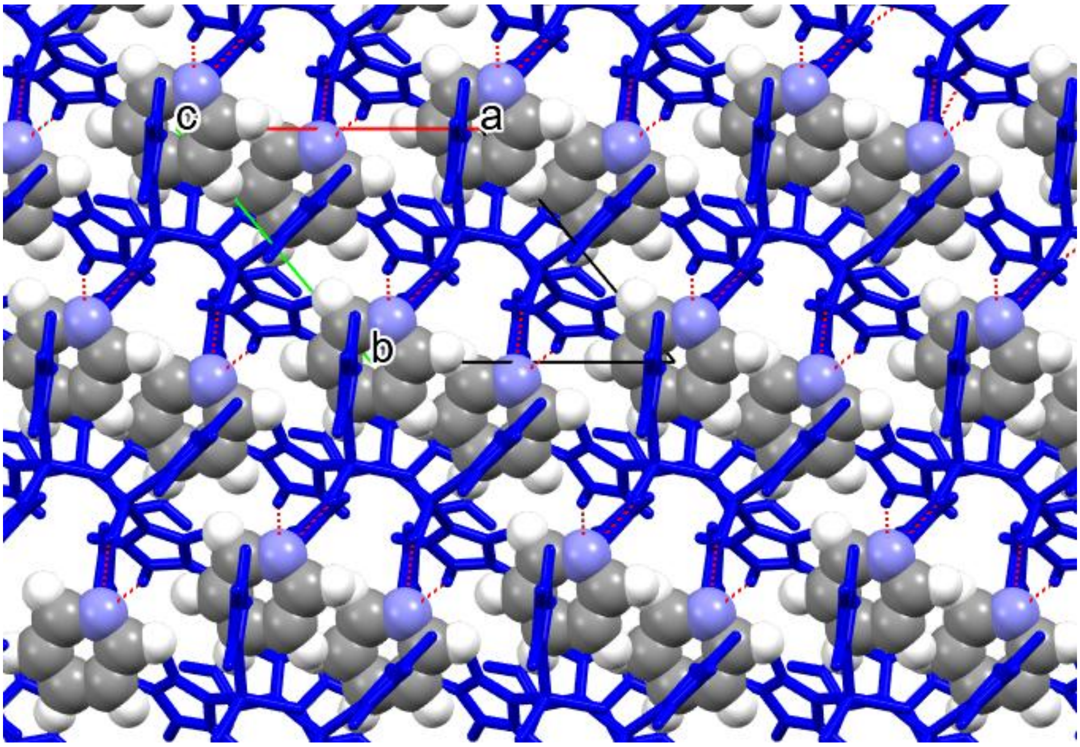

Figure S92. Geometry optimized structure of 0.5PYR@**neb2**-Zn**Im**$_2$. The Zn**Im**$_2$ parent framework is displayed in blue capped sticks, and the DBF guests in CPK spacefill. The C-H···N bonds are shown dashed in red.

2.10. Quantitative Rietveld refinement of nano-ZnO experiments

The amount of leftover ZnO in the syntheses of 0.65PhMe@**crbT**-Zn**Im**$_2$ was quantified by Rietveld refinement for two cases: the reaction starting from regular ZnO and the reaction starting from nanoparticulate ZnO. The amounts of residual ZnO were found to be 3.8(3)% and 1.7(2)% for regular and nanoparticulate ZnO, respectively. The rigid bodies and toluene occupancies were refined in both cases, with the determined toluene composition remaining consistent: $x_{regular} = 0.85$ and $x_{nano} = 0.83$.

3. REFERENCES

(1) Degen, T. ; Sadki, M. ; Bron, E. ; König, U. ;; Nénert, G. The HighScore Suite. *Powder Diffr.* **2014**, *29*, S13–S18.

(2) Kourkoumelis, N.; O'Neill, L. ICDD Annual Spring Meetings: PowDLL, a Reusable .NET Component for Interconverting Powder Diffraction Data: Recent Developments. In *Powder Diffr.*; 2013; Vol. 28, pp 137–148.

(3) Macrae, C. F.; Sovago, I.; Cottrell, S. J.; Galek, P. T. A.; McCabe, P.; Pidcock, E.; Platings, M.; Shields, G. P.; Stevens, J. S.; Towler, M.; Wood, P. A. Mercury 4.0: From Visualization to Analysis, Design and Prediction. *J Appl Crystallogr* **2020**, *53* (1), 226–235. https://doi.org/10.1107/S1600576719014092.

(4) Yvon, K.; Jeitschko, W.; Parthé, E. LAZY PULVERIX, a Computer Program, for Calculating X-Ray and Neutron Diffraction Powder Patterns. *J. Appl. Cryst.* **1977**, *10*, 73–74.

(5) Groom, C. R.; Bruno, I. J.; Lightfoot, M. P.; Ward, S. C. The Cambridge Structural Database. *Acta Crystallogr B Struct Sci Cryst Eng Mater* **2016**, *72* (2), 171–179. https://doi.org/10.1107/S2052520616003954.

(6) Gražulis, S.; Daškevič, A.; Merkys, A.; Chateigner, D.; Lutterotti, L.; Quirós, M.; Serebryanaya, N. R.; Moeck, P.; Downs, R. T.; Le Bail, A. Crystallography Open Database

(COD): An Open-Access Collection of Crystal Structures and Platform for World-Wide Collaboration. *Nucleic Acids Res* **2012**, *40* (D1). https://doi.org/10.1093/nar/gkr900.
(7) Sheldrick, G. M. A Short History of SHELX. *Acta Crystallogr A* **2008**, *64* (1), 112–122. https://doi.org/10.1107/S0108767307043930.
(8) Sheldrick, G. M. Crystal Structure Refinement with SHELXL. *Acta Crystallogr C Struct Chem* **2015**, *71* (1), 3–8. https://doi.org/10.1107/S2053229614024218.
(9) Barbour, L. J. X-Seed — A Software Tool for Supramolecular Crystallography. *Journal of Supramolecular Chemistry* **2001**, *1* (4–6), 189–191. https://doi.org/10.1016/S1472-7862(02)00030-8.
(10) Park, K. S.; Ni, Z.; Côté, A. P.; Choi, J. Y.; Huang, R.; Uribe-Romo, F. J.; Chae, H. K.; O'Keeffe, M.; Yaghi, O. M. Exceptional Chemical and Thermal Stability of Zeolitic Imidazolate Frameworks. *Proceedings of the National Academy of Sciences* **2006**, *103* (27), 10186–10191. https://doi.org/10.1073/pnas.0602439103.
(11) Boultif, A.; Louër, D. Powder Pattern Indexing with the Dichotomy Method. *J Appl Crystallogr* **2004**, *37* (5), 724–731. https://doi.org/10.1107/S0021889804014876.
(12) Altomare, A.; Giacovazzo, C.; Guagliardi, A.; Moliterni, A. G. G.; Rizzi, R.; Werner, P.-E. New Techniques for Indexing: N-TREOR in EXPO. *J Appl Crystallogr* **2000**, *33* (4), 1180–1186. https://doi.org/10.1107/S0021889800006427.
(13) Altomare, A.; Cuocci, C.; Giacovazzo, C.; Moliterni, A.; Rizzi, R.; Corriero, N.; Falcicchio, A. EXPO2013: A Kit of Tools for Phasing Crystal Structures from Powder Data. *J Appl Crystallogr* **2013**, *46* (4), 1231–1235. https://doi.org/10.1107/S0021889813013113.
(14) Le Bail, A.; Duroy, H.; Fourquet, J. L. Ab-Initio Structure Determination of LiSbWO6 by X-Ray Powder Diffraction. *Mater Res Bull* **1988**, *23* (3), 447–452. https://doi.org/10.1016/0025-5408(88)90019-0.
(15) Coelho, A. A. TOPAS and TOPAS-Academic: An Optimization Program Integrating Computer Algebra and Crystallographic Objects Written in C++. *J Appl Crystallogr* **2018**, *51* (1), 210–218. https://doi.org/10.1107/S1600576718000183.
(16) Aminoff, G. XXIV. Über Lauephotogramme Und Struktur von Zinkit. *Z Kristallogr Cryst Mater* **1921**, *56* (1–6), 495–505. https://doi.org/10.1524/zkri.1921.56.1.495.
(17) Blatov, V. A.; Shevchenko, A. P.; Proserpio, D. M. Applied Topological Analysis of Crystal Structures with the Program Package ToposPro. *Cryst Growth Des* **2014**, *14* (7), 3576–3586. https://doi.org/10.1021/cg500498k.
(18) Shevchenko, A. P.; Shabalin, A. A.; Karpukhin, I. Yu.; Blatov, V. A. Topological Representations of Crystal Structures: Generation, Analysis and Implementation in the *TopCryst* System. *Science and Technology of Advanced Materials: Methods* **2022**, *2* (1), 250–265. https://doi.org/10.1080/27660400.2022.2088041.
(19) Tian, Y.; Cai, C.; Ren, X.; Duan, C.; Xu, Y.; Gao, S.; You, X. The Silica-Like Extended Polymorphism of Cobalt(II) Imidazolate Three-Dimensional Frameworks: X-ray Single-Crystal Structures and Magnetic Properties. *Chemistry – A European Journal* **2003**, *9* (22), 5673–5685. https://doi.org/10.1002/chem.200304957.
(20) Martins, G. A. V.; Byrne, P. J.; Allan, P.; Teat, S. J.; Slawin, A. M. Z.; Li, Y.; Morris, R. E. The Use of Ionic Liquids in the Synthesis of Zinc Imidazolate Frameworks. *Dalton Transactions* **2010**, *39* (7), 1758–1762. https://doi.org/10.1039/b917348j.
(21) Clark, S. J.; Segall, M. D.; Pickard, C. J.; Hasnip, P. J.; Probert, M. I. J.; Refson, K.; Payne, M. C. First Principles Methods Using CASTEP. *Z Kristallogr Cryst Mater* **2005**, *220* (5–6), 567–570. https://doi.org/10.1524/zkri.220.5.567.65075.
(22) Björkman, T. CIF2Cell: Generating Geometries for Electronic Structure Programs. *Comput Phys Commun* **2011**, *182* (5), 1183–1186. https://doi.org/10.1016/j.cpc.2011.01.013.
(23) Perdew, J. P.; Burke, K.; Ernzerhof, M. Generalized Gradient Approximation Made Simple. *Phys Rev Lett* **1996**, *77* (18), 3865–3868. https://doi.org/10.1103/PhysRevLett.77.3865.
(24) Grimme, S.; Antony, J.; Ehrlich, S.; Krieg, H. A Consistent and Accurate Ab Initio Parametrization of Density Functional Dispersion Correction (DFT-D) for the 94 Elements H-Pu. *J Chem Phys* **2010**, *132* (15). https://doi.org/10.1063/1.3382344.
(25) Monkhorst, H. J.; Pack, J. D. Special Points for Brillouin-Zone Integrations. *Phys Rev B* **1976**, *13* (12), 5188–5192. https://doi.org/10.1103/PhysRevB.13.5188.

(26) Lanchas, M.; Vallejo-Sánchez, D.; Beobide, G.; Castillo, O.; Aguayo, A. T.; Luque, A.; Román, P. A Direct Reaction Approach for the Synthesis of Zeolitic Imidazolate Frameworks: Template and Temperature Mediated Control on Network Topology and Crystal Size. *Chemical Communications* **2012**, *48* (79), 9930–9932. https://doi.org/10.1039/c2cc34787c.
(27) Spencer, E. C.; Angel, R. J.; Ross, N. L.; Hanson, B. E.; Howard, J. A. K. Pressure-Induced Cooperative Bond Rearrangement in a Zinc Imidazolate Framework: A High-Pressure Single-Crystal X-Ray Diffraction Study. *J Am Chem Soc* **2009**, *131* (11), 4022–4026. https://doi.org/10.1021/ja808531m.
(28) Schröder, C. A.; Baburin, I. A.; van Wüllen, L.; Wiebcke, M.; Leoni, S. Subtle Polymorphism of Zinc Imidazolate Frameworks: Temperature-Dependent Ground States in the Energy Landscape Revealed by Experiment and Theory. *CrystEngComm* **2013**, *15* (20), 4036–4040. https://doi.org/10.1039/C2CE26045J.
(29) Zhou, C.; Stepniewska, M.; Sørensen, J. M.; Scarpa, L.; Magnacca, G.; Boffa, V.; Bennett, T. D.; Yue, Y. Polymorph Formation for a Zeolitic Imidazolate Framework Composition - Zn(Im)2. *Microporous and Mesoporous Materials* **2018**, *265*, 57–62. https://doi.org/10.1016/j.micromeso.2018.01.038.
(30) Guo, S.; Li, H.-Z.; Wang, Z.-W.; Zhu, Z.-Y.; Zhang, S.-H.; Wang, F.; Zhang, J. Syntheses of New Zeolitic Imidazolate Frameworks in Dimethyl Sulfoxide. *Inorg Chem Front* **2022**, *9* (9), 2011–2015. https://doi.org/10.1039/D1QI01538A.
(31) Banerjee, R.; Phan, A.; Wang, B.; Knobler, C.; Furukawa, H.; O'Keeffe, M.; Yaghi, O. M. High-Throughput Synthesis of Zeolitic Imidazolate Frameworks and Application to CO_2 Capture. *Science (1979)* **2008**, *319* (5865), 939–943. https://doi.org/10.1126/science.1152516.
(32) Tian, Y.; Zhao, Y.; Chen, Z.; Zhang, G.; Weng, L.; Zhao, D. Design and Generation of Extended Zeolitic Metal–Organic Frameworks (ZMOFs): Synthesis and Crystal Structures of Zinc(II) Imidazolate Polymers with Zeolitic Topologies. *Chemistry – A European Journal* **2007**, *13* (15), 4146–4154. https://doi.org/10.1002/chem.200700181.
(33) Schröder, C. A.; Saha, S.; Huber, K.; Leoni, S.; Wiebcke, M. Metastable Metal Imidazolates: Development of Targeted Syntheses by Combining Experimental and Theoretical Investigations of the Formation Mechanisms. *Z Kristallogr Cryst Mater* **2014**, *229* (12), 807–822. https://doi.org/10.1515/zkri-2014-1788.
(34) Shi, Q.; Kang, X.; Shi, F.-N.; Dong, J. $Zn_{10}(Im)_{20}\cdot$4DBF: An Unprecedented 10-Nodal Zeolitic Topology with a 10-MR Channel and 10 Crystallographically Independent Zn Atoms. *Chemical Communications* **2015**, *51* (6), 1131–1134. https://doi.org/10.1039/C4CC07105K.
(35) Altomare, A.; Cuocci, C.; Giacovazzo, C.; Moliterni, A.; Rizzi, R. COVMAP: A New Algorithm for Structure Model Optimization in the EXPO Package. *J Appl Crystallogr* **2012**, *45* (4), 789–797. https://doi.org/10.1107/S002188981201953X.
